# Automatic Classification of Artifactual ICA-Components for Artifact Removal in EEG Signals

**DOI:** 10.1186/1744-9081-7-30

**Published:** 2011-08-02

**Authors:** Irene Winkler, Stefan Haufe, Michael Tangermann

**Affiliations:** 1 Machine Learning Laboratory, Berlin Institute of Technology, Franklinstr. 28/29, 10587 Berlin, Germany

## Abstract

**Background:**

Artifacts contained in EEG recordings hamper both, the visual interpretation by experts as well as the algorithmic processing and analysis (e.g. for Brain-Computer Interfaces (BCI) or for Mental State Monitoring). While hand-optimized selection of source components derived from Independent Component Analysis (ICA) to clean EEG data is widespread, the field could greatly profit from automated solutions based on Machine Learning methods. Existing ICA-based removal strategies depend on explicit recordings of an individual's artifacts or have not been shown to reliably identify muscle artifacts.

**Methods:**

We propose an automatic method for the classification of general artifactual source components. They are estimated by TDSEP, an ICA method that takes temporal correlations into account. The linear classifier is based on an optimized feature subset determined by a Linear Programming Machine (LPM). The subset is composed of features from the frequency-, the spatial- and temporal domain. A subject independent classifier was trained on 640 TDSEP components (reaction time (RT) study, n = 12) that were hand labeled by experts as artifactual or brain sources and tested on 1080 new components of RT data of the same study. Generalization was tested on new data from two studies (auditory Event Related Potential (ERP) paradigm, n = 18; motor imagery BCI paradigm, n = 80) that used data with different channel setups and from new subjects.

**Results:**

Based on six features only, the optimized linear classifier performed on level with the inter-expert disagreement (*<*10% Mean Squared Error (MSE)) on the RT data. On data of the auditory ERP study, the same pre-calculated classifier generalized well and achieved 15% MSE. On data of the motor imagery paradigm, we demonstrate that the discriminant information used for BCI is preserved when removing up to 60% of the most artifactual source components.

**Conclusions:**

We propose a universal and efficient classifier of ICA components for the subject independent removal of artifacts from EEG data. Based on linear methods, it is applicable for different electrode placements and supports the introspection of results. Trained on expert ratings of large data sets, it is not restricted to the detection of eye- and muscle artifacts. Its performance and generalization ability is demonstrated on data of different EEG studies.

## Background

Signals of the electroencephalogram (EEG) can reflect the electrical background activity of the brain as well as the activity which is specific for a cognitive task during an experiment. As the electrical field generated by neural activity is very small, it can only be recognized by EEG if large assemblies of neurons show a similar behavior. Resulting neural EEG signals are in the range of micro volts only and can easily be masked by artifactual sources. Typical artifacts of the EEG are caused either by the non-neural physiological activities of the subject or by external technical sources. Eye blinks, eye movements, muscle activity in the vicinity of the head (e.g. face muscles, jaws, tongue, neck), heart beat, pulse and Mayer waves are examples for physiological artifact sources, while swaying cables in the magnetic field of the earth, line humming, power supplies or transformers can be the cause of technical artifacts.

Brain-Computer Interfaces (BCI) are based on the single trial classification of the ongoing EEG signal and can improve the life quality of disabled individuals especially in combination with other assitive technology [1]. The exclusion of artifacts is of special interest for BCI applications, as the intended or unconscious use of artifacts for BCI control are usually not desirable when the BCI system is tested on healthy subjects. Furthermore, as averaging methods have to be avoided, these real-time systems BCIs rely on relatively clean EEG signals. The same holds true for other Mental State Monitoring applications, that monitor a subject's mental state continuously and on a fine granular time resolution to detect changes e.g. of wakefulness, responsiveness or mental workload as early as possible [2].

The two physiological artifacts most problematic for BCI applications are ocular (EOG) and muscle (EMG) artifacts. EOG activity is either caused by rolling of the eyes or by eye blinks which occur approx. 20 times per minute [3]. Both result in a low-frequency activity most prominent over the anterior head regions, with maximal frequencies below 4 Hz. In contrast, EMG activity (caused by chewing, swallowing, head or tongue movements) is usually a high-frequency activity (*>*20 Hz) which ranges from rather small to very large amplitudes [4].

For an extensive review of artifact reduction techniques in the context of BCI-systems, the reader can refer to Fatourechi et al. [5]. Since the rejection of artifactual trials amounts to a considerable loss of data, a method that removes the artifacts while preserving the underlying neural activity is needed. For example, linear filtering is a simple and effective method if artifactual and neural activity are located in non-overlapping frequency bands. Unfortunately, artifacts and the brain signal of interest do usually overlap. Nevertheless, ocular activity can be partially removed by regression-based methods, which subtract a part of the activity measured at additional electrooculogram (EOG) channels from the EEG (see [6] for a review). Regression-based methods require the reliable recording of additional EOG channels and are limited by the fact that the EOG is contaminated by brain activity which is removed as well. Furthermore, they cannot eliminate non-eye activity.

**Figure 1:**
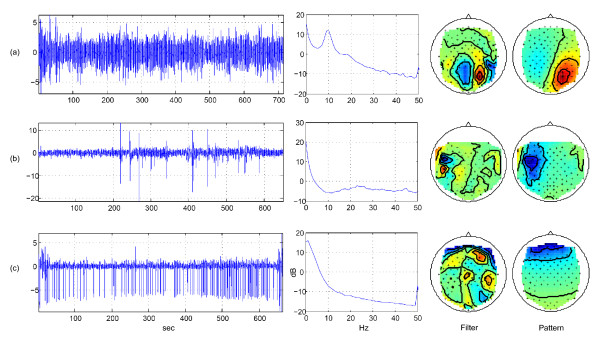
**Three example independent source components**. Time series (first column), spectrum (second column), filter (third column) and pattern (fourth column) of three components. The first row (a) shows an alpha generator in the occipital lobe. The second row (b) shows a rare muscle artifact component with an increased spectrum in higher frequencies. The third line (c) shows an eye artifact component that appears regularly, has an increases spectrum in lower frequencies and a typical front-back distribution in the pattern.

If artifactual signal components and neural activity of interest are not systematically co-activated due to a disadvantageous experimental design, methods of Blind Source Separation (BSS) like Independent Component Analysis (ICA) are promising approaches for their separation [7,8]. A common approach is the transformation of the EEG signals into a space of independent source components, the hand-selection of non-artifactual neural sources and the reconstruction of the EEG without the artifactual components (for an example of independent source components, see Figure 1). While assumptions for the application of ICA methods are only approximately met in practice (linear mixture of independent components, stationarity of the sources and the mixture, and prior knowledge about the number of components), their application usually leads to a good separation, with only a small number of hybrid components that contain both, artifacts and neural signals [9-12].

Existing methods for artifact rejection can be separated into hand-optimized, semi-automatic and fully automatic approaches. Semi-automatic approaches require user interaction for ambiguous or outlier components [13,14]. While fully automated methods were proposed for the classification of eye artifacts [15,16], these methods do not easily generalize to non-eye artifacts or even require the additional recording of the EOG [17,18]. Viola et al. and Mognon et al. [19,20] both developed an EEGLAB plug-in which finds artifactual independent components. Both plug-ins have a fully automatic mode that has been shown to recognize and reject major artifacts like eye blinks, eye movements and heart beats, while the detection of muscular or more subtle artifacts has not been reported. The plug-in developed by Viola et al. relies on a user-defined template, while Mognon's approach does not require user interaction.

Existing more flexible approaches for the general classification of different artifact types were reported for EEG data of epileptic patients [21], where the authors report a Mean Squared Error (MSE) of approx. 20% for their system based on a Bayesian classifier. Halder et al. [22] report a classification error below 10% for their Support Vector Machine (SVM) based system for a fixed number of electrodes if dedicated artifact recordings are available for the classifier training. But even if such optimized conditions are present, difficulties of separating muscle artifact components from neural components are common [22].

The review of the existing literature did not reveal a systematic screening of potentially discriminant features for the general task of artifact detection/removal. Moreover, most approaches restrict themselves to part of the available information, e.g. rely on spatial patterns only [19], or spatial patterns and spectral features [22], or spatial pattern and temporal features [20].

Our proposed solution for a general artifact detection method is motivated by the needs of EEG practitioners. First, it is desirable that a method efficiently and reliably detects all classes of artifacts, e.g. is not restricted to eye-, heart beat-, or muscle artifacts. Second, a practical method must be applicable post-hoc, i.e. without the need of dedicated artifact recordings at the time of the experiment. Third, it is difficult to convince EEG practitioners to use a method of artifact rejection if it is a black box and refuses introspection. As the goal must be to develop a method, that delivers interpretable and easy to understand results, we decided for a linear classification method. Luckily, linear methods have proven a high performance for a number of classification tasks in the field of EEG-based BCI systems. However, to be able to estimate the performance loss compared to a potentially better, but difficult to interpret, non-linear classification method, the results of a Gaussian SVM are reported in parallel.

We decided to use a sparse approach (sparsity in the features) although it is a mixed blessing. It leads to a trade-off between efficiency and interpretability, as redundant but slightly less discriminative features are removed with high probability from the overall set of features. This has to be kept in mind during the analysis of results. To reach the goal of a sparse method that delivers physiologically interpretable results, we decided to incorporate a thorough feature selection procedure in combination with a linear classification method that is based on features of all three available information domains of EEG data: the spatial domain (e.g. patterns of independent components), the frequency domain and the temporal domain. 

The paper is organized as follows: In the methods section, a reaction time (RT) paradigm is introduced, as data from this study forms the basis for the construction of the proposed artifact detection method. After the signal pre-processing methods (including a temporal variant of ICA) are introduced, we describe 38 features that are candidates for the artifact discrimination task. Based on labels provided by EEG experts, a thorough feature selection procedure is described, that is used to condense the 38 features to a small subset. Furthermore, classification methods are introduced. The methods section ends with a description of two other EEG paradigms (auditory Event Related Potential (ERP) and motor imagery for BCI), that will be used to validate the generalization approach of the proposed artifact classifier. In the results section, the outcome of the feature selection procedure is given, together with the artifact classification performance on unseen data of the RT paradigm, data of a unseen auditory ERP paradigm. Finally the method is applied in the context of a motor imagery BCI setup, before the paper closes with a discussion.

## Methods

In the following subsections, we will describe how the proposed new artifact classification method is set up. Then we will introduce two further studies that are utilized to test the classifier's generalizability.

Participants of the studies described below provided verbal and written informed consent and were free to stop their participation at any time. All collected data was anonymized before any subsequent analysis or presentation took place.

### Classifier Construction using a RT study

The artifact classifier is set up based on labeled independent components gained from a reaction time (RT) study.

#### Experimental Setup

Data from 12 healthy right-handed male subjects were used to train and to test the proposed automated component classification method. Every subject participated in one EEG recording session of approx. 5 hours duration. EEG was recorded from 121 approx. equidistant sensors and high pass filtered at 2 Hz. During this session, 4 repeated blocks of 3 different conditions (C0, C1, C2) were performed. Each block lasted approx. 45 minutes. During all three conditions, subjects performed a forced-choice left or right key press reaction time task upon two auditory stimuli in an oddball paradigm. The key press actions were performed with micro switches attached to the index fingers. During condition C0 subjects had to gaze at a fixation cross without any further visual task. Condition C1 introduced an additional distraction, as a video of a driving scene had to be watched passively on a screen. Condition C2 introduced an additional second task: subjects infrequently had to follow simple lane change instructions and control a steering wheel. By design, EEG recordings under condition C2 were inevitably more prone to muscle and eye artifacts, while C1 possibly stimulated eye movement artifacts, but not muscle artifacts. However, all subjects had been instructed during all conditions to avoid producing artifacts.

#### Unmixing and data split

To avoid the artificial split of signal components due to the high dimensionality of the data, the separation of the EEG signals by an ICA method was preceded by a dimensionality reduction by Principal Component Analysis (PCA) from 121 EEG channels in the sensor space into *k *= 30 PCA components. This choice of *k *was based on previous experience, but was probably not the optimal choice. The TDSEP algorithm (Temporal Decorrelation source SEParation) [23] was used to transform the 30 PCA components into 30 independent source components. PCA and TDSEP were applied in a subject specific way, i.e. PCA and TDSEP matrices were calculated seperately for each subject.

TDSEP is a BSS algorithm to estimate a linear demixing(1)

of a given multivariate time series *X *= (*x*_1_,..., *x_k_*)*^T ^*into unknown, assumed mutually independent source components *S *= (*s*_1_,..., *s_k_*)*^T^*. Note that both the demixing *W *and the source components *S *are unknown, and that BSS algorithms differ in the definition of independence between components. While ICA algorithms exploit higher order statistics, TDSEP relies on second-order statistics by taking the temporal structure of the time series into account. TDSEP amounts to finding a demixing *W *which leads to minimal cross-covariances over several time-lags between all pairs of components of *S*.

For a mathematical discussion, let  be the cross-covariance matrix of the whitened data *X*_w _at time-lag *τ*, where the whitening transformation linearly decorrellates and scales the data such that Σ(0) = *I*. Consider now that (1) Whitening reduces the BSS problem to finding an orthogonal demixing matrix ; (2)  equals the cross-covariance matrix of the source components *S *at time-lag *τ*; and (3) The independence assumption yields that the cross-covariance matrix of the source components *S *at time-lag *τ *is a diagonal matrix. TDSEP thus computes  as the matrix that jointly diagonalizes a set of whitened cross-covariances Σ(*τ*). Here we use *τ *= 1,..., 99.

In the context of EEG signals, TDSEP finds *k *independent components contributing to the scalp EEG. They are now characterized by their time course, a spatial pattern given by the respective column of the mixing matrix *A *:= *W*^-1^, and a spatial filter given by the respective row of the demixing matrix *W*. The pattern contains the projection strengths of the respective component onto the scalp electrodes, whereas the filter gives the projection strength of the scalp sensors onto the source component (see, e.g [24]). All resulting source components were hand labeled into artifactual and non-artifactual components by two experts who each labeled one half of the ICA components based on four plots per component, namely the time series, the frequency spectrum and one scalp plot of the component's filter and one of its pattern. Not all components were unambigious but instead contained a mixture of neural and artifactual activity. Discarding all those components which contain traces of artifacts would remove too much of the relevant neural activity. Therefore, only those mixed components were labeled as artifacts, that revealed a relatively small amount of neural activity compared to the strength of the artifact contained.

For the training of the proposed automated classification method, 23 EEG recordings of 10 minutes duration were taken from the first experimental block only, leading to 690 labeled source components. Neural components and artifact components were approx. equally distributed (46% vs. 54%). Figure 1 shows typical examples of two artifacts and one neural component.

The trained classifier was tested on 36 unseen EEG recordings from the third experimental blocks. Among these 1080 source components were 47% neuronal components and 53% artifact components.

#### Feature Extraction

In order to provide substantial information to an automated classification method, we construct an initial feature set that contains 13 features from a component's time series, 9 features from its spectrum and 16 from its pattern. Based on this collection of 38 features a subset of the most discriminative features is determined in a feature selection procedure.

#### Features derived from a component's time series

1. ***Variance ***of a component's time series. It is not possible to determine the variances of the independent components, as both *S *and *A *:= *W*^-1 ^are unknown, and the solution is thus undetermined up to scaling. We estimate the impact one independent component *s_i _*has on the original EEG by calculating Var(std(*A_i_*) · *s_i_*) where *A_i_* denotes the respective pattern. The idea here is to calculate the standard deviation of one independent component when its corresponding pattern has unit variance.

2. ***Maximum Amplitude***

3. ***Range ***of the signal amplitude

4. ***Max First Derivative***, approximated for the discrete signal *s*(*t*) in *t_i _*by 

5. ***Kurtosis***

6. ***Shannon Entropy***

7. ***Deterministic Entropy***, a computationally tractable measure related to the Kolmogorov complexity of a signal [25]

8. ***Variance of Local Variance ***of time intervals of 1 s and of 15 s duration (2 separate features)

9. ***Mean Local Variance ***of time intervals of 1 s duration, and of 15 s duration (2 separate features)

10. ***Mean Local Skewness***, the mean absolute local skewness of time intervals of 1 s and 15 s duration (2 separate features)

The above 13 features were all logarithmized in a last step. With exception of the *Variance* feature all were calculated after standardization of the time series to variance 1. These features describe outliers in terms of unusual high amplitude values, as they are typically present in blinks and muscle artifacts. Furthermore, they are sensitive to non-stationarities and non-normal higher order moments in the time series signal, as they can be expected by muscle activity which typically is not present equally strong over the full duration of 10 min.

#### Features derived from a component's spectrum

1. ***k*_1_**, ***λ***, ***k*_2 _**and ***Fit Error ***describe the deviation of a component's spectrum from a prototypical 1/frequency curve and its shape. The parameters *k*_1_, *λ*, *k*_2 _*>*0 of the curve(2)

are determined by three points of the log spectrum: (1) value at 2 Hz, (2) local minimum in the band 5-13 Hz, (3) local minimum in the band 33-39 Hz. The logarithm of *k*_1_, *λ*, *k*_2 _and of the mean squared error of the approximation to the real spectrum are used as features.

The spectrum of muscle artifacts, characterized by unusual high values in the 20-50 Hz range, are thus approximated by a comparatively steep curve with high *λ *and low *k*_1_.

2. ***0-3 Hz, 4-7 Hz, 8-13 Hz, 14-30 Hz, 31-45 Hz***, the average log band power of the *δ *(0-3 Hz), *θ *(4-7 Hz), *α *(8-13 Hz), *β *(14-30 Hz) and *γ *(31-45 Hz) band.

#### Features derived from a component's pattern

**Figure 2:**
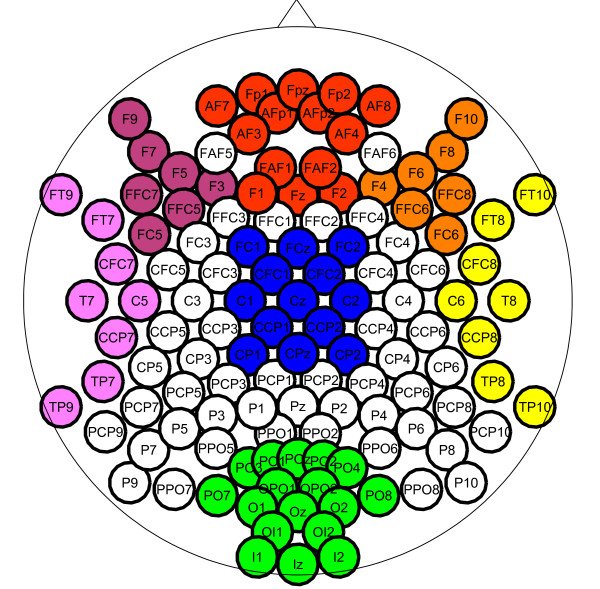
**Scalp electrode sets**. Mean activation in the 7 colored electrode groups are used as features.

**Figure 3:**
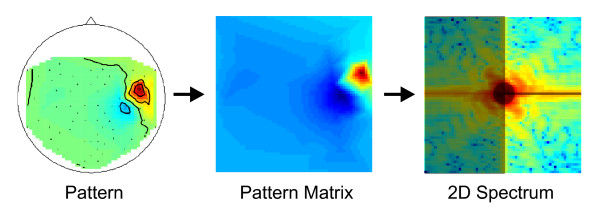
**The feature 2DDFT**. The feature 2DDFT is the average logarithmic band power of higher frequencies of the 1st and 4th quadrant of the 2D-Fourier spectrum of the pattern matrix.

**Figure 4:**
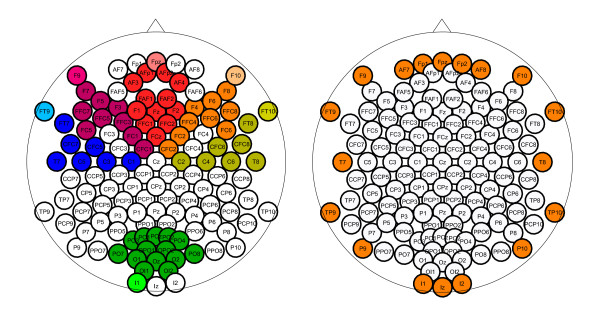
**The feature Border Activation**. Electrode groups (left) and electrodes (right) used to determine the feature Border Activation.

1. ***Range Within Pattern***, logarithm of the difference between the minimal and the maximal activation in a pattern

2. ***Spatial Distance of Extrema***, logarithm of the Euclidean norm of the 2D-coordinates of the minimal and maximal activation in a pattern

3. ***Spatial Mean Activation Left, Left Frontal, Frontal, Right Frontal, Right, Occipital, Central***, logarithm of the average activation in 7 groups of electrodes as depicted in Figure 2

4. ***2DDFT***. Pattern without a "smooth" activity distribution do not originate from an easily traceable psychological source and are thus artifacts or mixed components. The spatial frequency of a pattern can be described by means of a two-dimensional discrete Fourier transformation. As a first step, the pattern is linearly interpolated to a quadratic 64x64 pattern matrix. The feature 2DDFT is the average logarithmic band power of higher frequencies of the 1st and 4th quadrant (see Figure 3) of the 2D-Fourier spectrum of the pattern matrix.

5. ***Laplace-Filter***. Laplace-filtering leads a second way of finding spatially high frequent patterns, as these have more defined edges. Similar to the 2DDFT-Feature, the pattern is linearly interpolated to a quadratic 64 × 64 pattern matrix. Then, a 3 × 3 Laplace filter is applied. The feature is defined as the logarithm of the Frobenius norm of the resulting matrix.

6. ***Border Activation***. This binary feature captures the spatial distribution at the borders of a pattern. It is defined as 1 if either the global maximum of the pattern is located at one of the outmost electrodes of the setup in Figure 4 (right), or if the local maximum of an electrode group in Figure 4 (left) is located at the outmost electrode of the group and if that local maximum deviates at least 2 standard deviations from the group average. Otherwise the feature is defined as -1. The idea behind this feature is that a pattern with maximal activation at its border is unlikely to be generated by a source inside the brain - it thus indicates an artifact.

7. ***Current Density Norm ***of estimated source distribution and strongest source's position ***x, y, z***. ICA itself does not provide information about the locations of the sources *S*. However, ICA patterns can be interpreted as EEG potentials for which a physical model is given by *a *= *Fz*. Here, *z *∈ ℝ^3*m *^are current moment vectors of unknown sources at *m *locations in the brain and *F *∈ ℝ^*k*× 3 m ^describes the mapping from sources to *k *sensors, which is determined by the shape of the head and the conductivities of brain, skull and skin tissues. We consider *m *= 2142 sources which are arranged in a 1 cm grid.

Source estimation can only be done under additional constraints since *k *≪ *m*. Commonly, the source distribution with minimal *l*_2_-norm (i.e., the "simplest" solution) is sought [26]. This leads to estimates(3)

where Γ approximately equalizes the cost of dipoles at different depths [27] and *λ *defines a trade-off between the simplicity of the sources and the fidelity of the model.

**Figure 5:**
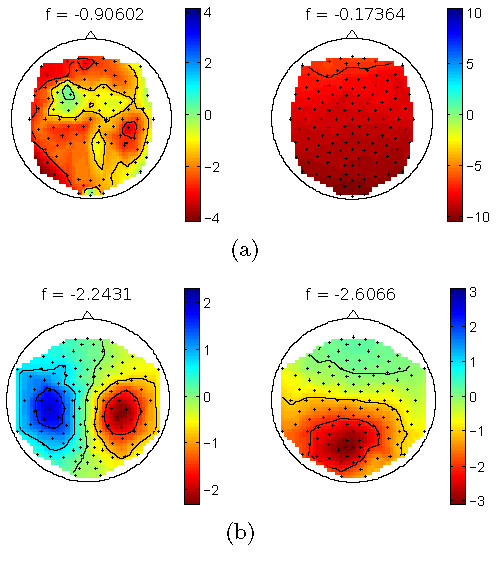
**The feature Current Density Norm**. (a) Two example patterns with high Current Density Norm *f *= log ||Γ*z*||. (b) Two example patterns with low Current Density Norm.

Since Eq. 3 models only cerebral sources, it is natural that noisy patterns and patterns originating outside the brain can only be described by rather complicated sources, which are characterized by a large *l*_2_-norm. For an example, see Figure 5. We propose to use  as a feature for discriminating physiological from noisy or artifactual patterns. Here  are normalized ICA patterns and *λ *= 100 was chosen from {0,1,10,100,1000} by cross-validation. To allow for a meaningful comparison of different f values over settings of varying numbers of electrodes, we pre-calculated Γ*J_λ _*on 115 electrodes and used only those rows that corresponded to the recorded electrodes. Note that while this approach is simple, it may not be the optimal choice when the set of electrodes varies.

Assuming a pattern is generated by only one source, we can estimate its 3D-coordinates *x*, *y*, *z *as the location of maximal current density. Note that this is only a very simple source localization method.

#### Feature Selection and Classification

We conduct an embedded feature selection by using the weight vector of a Linear Programming Machine (LPM) [28]. Like all binary linear classifiers it finds a separating hyperplane *H *: ℝ*^d ^*∋ **x** ↦ sign(**w***^T ^*· **x **+ *b*) ∈ {-1, 1} characterized by a weight vector **w **and a bias term *b*. If the features are zero-mean and have same variance, their importance for the classification task can be ranked by their respective absolute weights |*w_i_*|. The LPM is known to produce a sparse weight vector **w **by solving the following minimization problem:(4)

We thus apply a LPM to the training data in a 5 × 10 cross-validation procedure with the goal to obtain a ranking of the features according to |*E*(*w_i_*/||*w*||)|. Beforehand, the LPM-hyperparameter *C *was set to *C *= 0.1 by a 5 × 10 cross-validation heuristic, such that LPM yielded good classification results while using a sparse feature vector, i.e. we selected C with the minimal number of features essential for the classification task (defined by |*E*(*w_i_*/||*w_i_*||)| *>*0.1) while the cross-validation error deviates less than one standard error from the minimal cross-validation error.

Having obtained a ranking of the features, the additional information needed is how many of the best-ranked features are optimal for classification. With the goal in mind to find a good trade-off between feature size and error we proceed as follows: For every rank position, we compute the cross-validation error obtained by a classification based on the best-ranked features. Then the number of best ranked features is selected to be the minimum number of features yielding a cross-validation error which deviates less than one standard error from the minimal cross-validation error.

Obviously, the number of features depends on the classification method. We compare a LPM, a non-linear Support Vector Machine (SVM) with Gaussian kernel [29] and a regularized Linear Discriminant Analysis (RLDA) [24], where we use a recently developed method to analytically calculate the optimal shrinkage parameter for regularization of LDA [30,31]. Since a nested cross-validation is computational expensive, the hyperparameters of SVM and LPM are set by an outer cross-validation, i.e. they are estimated on the whole training set which leads to a slight overfitting on the training data.

As a last step, the final classifier was trained on the full training data (690 examples) on the selected features, and tested on unseen test data (1080 examples).

### Validation in an auditory ERP study

To evaluate the artifact detection performance beyond the training domain, data from 18 healthy subjects were used to test the proposed automated component classification method in a completely different setup of an auditory ERP study.

#### Experimental Setup

A group of 18 subjects of 20 to 57 years of age (mean = 34.1, SD = 11.4) underwent an EEG recording of approx. 30 min duration using 64 Ag/AgCl electrodes of approx. equidistant sensors. EEG was band-pass filtered between 0.1-40 Hz. Note that this setup differs from the RT experiment, where EEG was recorded from 121 electrodes and high-pass filtered at 2 Hz.

The subjects were situated in the center of a ring of six speakers (at ear height). During several short trials they listened to a rapid sequence (Stimulus Onset Asynchrony = 175 ms) of six auditory stimuli of 40 ms duration. The six stimuli varied in pitch and noise. Each stimulus type was presented from one speaker only, and each speaker emitted one stimulus type only such that *direction *was a discriminant cue in addition to the pitch/noise characteristics. Subjects had to count the number of appearances of a rare target tone, that was presented in a pseudo-random sequence together with 5 frequent non-target tones (ratio 1:5).

#### Unmixing and Classification

A PCA reduced the dimensionality of the EEG channels to 30 PCA components. Then, the TDSEP algorithm was used to transform the 30 PCA components into 30 independent source components. The resulting 540 source components were hand labeled by two experts into artifactual and non-artifactual source components. One of the experts had participated in the rating of the RT-study. Both experts rated all independent components. On average, the experts identified 28% neuronal components and 72% artifactual components (expert 1: 25% neuronal components, expert 2: 31% neuronal components). The labeled data was used to test how the artifact classifier generalizes to new data acquired in a different experimental setup by training the classifier solely on the training data from the RT experiment and applying it to this unseen data set.

### Application to Motor Imagery BCI

To investigate the possibility of removing relevant neural activity, we incorporated our automatic ICA-classification step in a motor-imagery BCI system. In this offine analysis we investigate how an ICA-artifact reduction step affects the classification performance of a motor imagery BCI system based on the Common Spatial Patterns (CSP) method. For a detailed discussion of CSP the reader is referred to [32].

#### Experimental Setup

Eighty healthy BCI-novices performed first motor imagery with the left hand, right hand and both feet in a calibration (i.e. without feedback) measurement. Every 8 s one of three different visual cues (arrows pointing left, right, down) indicated to the subject which type of motor imagery to perform. Three runs with 25 trials of each motor condition were recorded. A classifier was trained using the pair of classes that provided best discrimination: CSP filters were calculated on the band-pass filtered signals and the log-variance of the spatially filtered signals were used to train a LDA. In a feedback measurement subjects could control a 1D cursor application in three runs of 100 trials [33].

#### Motor Imagery BCI preceded by ICA-based artifact reduction

**Figure 6:**
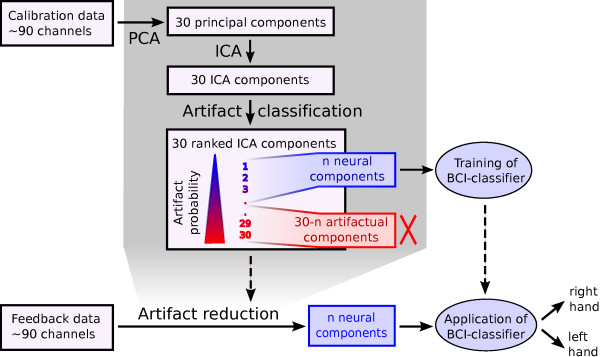
**Artifact reduction step included in the standard CSP-procedure**. The linear artifactreduction transformation of the original EEG into 6 - 30 signal components is calculated in the calibration phase. This transformation is applied to the feedback data.

The steps conducted to incorporate the artifact reduction are illustrated in Figure 6. The first step consists of a dimensionality reduction from about 90 EEG channels in the sensor space into k = 30 PCA components. As in the previous experiments, TDSEP was used to transform the 30 PCA components into 30 independent source components. Then, the component classifier trained on the RT experiment was applied. The components were ranked based on the classifiers output, which was used as a surrogate for the probability of being an artifact. Retaining a smaller or larger number of sources corresponds to an either very strict or soft policy for the removal of potential artifactual sources. We retained 6 to 30 source components of the most probable true neural sources, and removed the others. Further analysis was performed on the remaining sources, i.e. CSP filters were determined on the remaining independent source components and the log-variance of the spatially filtered signals were used to train an LDA.

Note that ICA artifact reduction methods usually reconstruct the EEG from the remaining neural sources. However, CSP solves an eigenvalue problem and requires the covariance matrix of the data to have full rank. Thus, CSP cannot be applied to the reconstructed EEG.

The application to the feedback measurement in a manner that allows for real-time BCI applications is straightforward: After un-mixing the original data according to the ICA filters determined on the calibration measurement, the previously determined 6 to 30 sources were selected for band-pass and CSP filtering and log-variance determination in order to form the test data features. To estimate the influence of the artifact reduction step on BCI performance, we compared the classification performance with artifact reduction (depending on the number of selected sources) with the standard CSP procedure using no artifact reduction.

## Results

In the following subsections, the results of the classifier model selection and its additional validation on new data sets is presented.

### Model Selection: RT study

**Figure 7:**
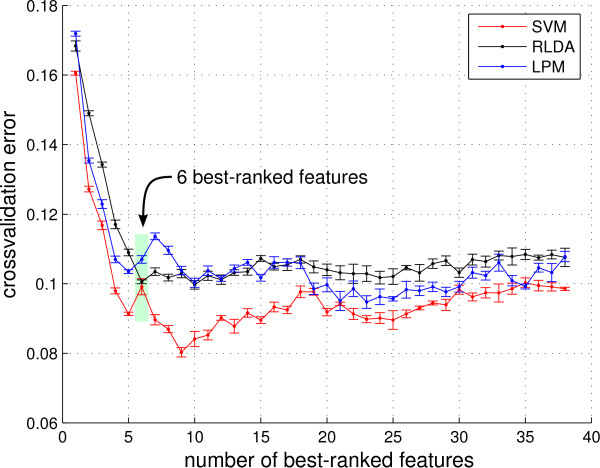
**Cross-validation error for SVM, RLDA and LPM against the number of best-ranked features**. A 10-fold cross-validation was repeated 5 times and standard errors are plotted. The SVM and LPM hyperparameters were selected by an outer cross validation. The number of 6 best-ranked features was determined for building the final classifier, as the estimated error of the RLDA starts to increase significantly for higher numbers of features.

The ranking of the features obtained by applying a LPM to the training data set of the RT study is shown in Table 1. Figure 7 shows the cross-validation errors for SVM, RLDA and LPM plotted against the size of the feature sub-set used for classification. The shape of the three curves reveals that at first, classification performance improves when adding features to the feature set. These features contain necessary but not redundant information. However, adding more than a certain number of features does not improve classification performance - these features only contain redundant information. Classification error slightly increases when more features are used for the classification task, which indicates that the classifier overfits on noisy and irrelevant features.

**Table 1:** Ranking of features obtained by LPM.

Feature	Weight
Current Density Norm	
Range Within Pattern	
Mean Local Skewness 15 s	
*λ*	
8-13 Hz	
FitError	

Border Activation	
2DDFT	
Spatial Mean Activation Central	
Max First Derivative	
Variance	
*k*_2_	
Spatial Mean Activation Left	
Spatial Mean Activation Left Frontal	
Laplace-Filter	
Mean Local Variance 15 s	
14-30 Hz	
4-7 Hz	
Mean Local Variance 1 s	
Spatial Distance of Extrema	
Spatial Mean Activation Occipital	
*k*_1_	
Maximum Amplitude	
*y*	
Spatial Mean Activation Right	
Kurtosis	
*x*	
0-3 Hz	
Deterministic Entropy	
Spatial Mean Activation Frontal	
*z*	
Variance of Local Variance 1 s	
Range	
Spatial Mean Activation Right Frontal	
Variance of Local Variance 15 s	
Mean Local Skewness 1 s	
31-45 Hz	
Shannon Entropy	

The diameter of the black circles visualizes the absolute LPM weights |*E*(*w_i_*/||*w*||)| per feature after learning on the training data set. (The LPM-hyperparameter C had been set to *C *= 0.1 based on the cross-validation performance.)

The fact that LPM performance is in the range of the RLDA classifier indicates that the feature ranking was suitable for our analysis (and not just for the LPM classifier). Given the ranking, the minimum number of features yielding a cross-validation error which deviates less than one standard error from the minimal cross-validation error is 9 for the SVM and only 6 for the RLDA. The SVM classifier slightly outperforms the RLDA classifier on the training data, but since our goal is to construct a simple linear classifier, we decided to use the RLDA classifier with the 6 best-ranked features. Notice that while SVM outperforms RLDA on the training data, this effect might be due to overfitting and disappears on the test data, as is shown in the next section.

The 6 best-ranked features are *Current Density Norm*, *Range Within Pattern*, *Mean Local Skewness 15 s*, *λ*, *8-13 Hz *and *FitError*. They incorporate information from the temporal, spatial and frequency domain.

### Validation 1: RT study

Testing the trained classifier on unseen data from the RT study (1080 examples from experimental block 3) leads to an mean-squared error (MSE) of 8.9% only, which corresponds to a high agreement with the expert's labeling. Interestingly, testing a trained SVM classifier (based on 9 selected features) leads to an error of 9.5%. Thus, after feature selection, the RLDA classifier performs as good as a SVM classifier on unseen test data.

Let's take a moment to interpret the obtained classifier: The weight vector **w **is given in Table 2. It shows that a high current density norm of a component indicates an artifactual component. Recall from the definition of the Current Density Norm feature that these components are in fact difficult to explain by a prominent source within the brain. Furthermore, components with a high range within the pattern (i.e. outliers in the pattern), a high local skewness (i.e. outliers in the time series), high *λ *(i.e. a steep spectrum typical for muscle artifacts) and low spectral power in the 8-13 Hz range (i.e. no prominent alpha peak) are rated as artifacts by the classifier. Interestingly, a low FitError, i.e. a low error when approximating the spectrum by a 1/f curve, indicates an artifact for the classifier. This is due to the fact that components which have no alpha peak in the spectrum are most probably artifacts. Notice that the FitError feature in itself is not very informative, because a high FitError cannot distinguish between components with a large alpha peak (which contain most probably neural activity) and components with an unusual high spectrum in higher frequency (which indicates muscle activity). However, in combination with the other five features, the FitError feature carries additional information which improve classification performance.

**Table 2:** Feature weight vector and test errors.

Feature	Feature weight	Test Error RT	Test Error ERP
Current Density Norm	0.342	0.141	0.488
Range Within Pattern	0.574	0.151	0.186
Mean Local Skewness 15 s	0.317	0.309	0.442
*λ*	0.569	0.177	0.144
8-13 Hz	-0.219	0.166	0.138
FitError	-0.286	0.424	0.640

Combined		0.089	0.147

Feature weights *w_i _*for each feature *x_i _*of the classifier *H *: ℝ^6 ^∋ **x **α sign(**w***^T ^*· **x **+ *b*) ∈ {-1, 1} with 1 ≙ Artifact and -1 ≙ Neuronal activity. Test error (MSE) for the 6 single features and for the combined classification for the RT experiment and the ERP experiment.

It is interesting to take a closer look at the performance of single features, which is also given in Table 2. The best one, Current Density Norm, leads to a MSE of 14.1% on the test data of the RT study. The combination of the six features from all three domains improves the error substantially compared to even the best single feature. This shows that features which are far from optimal in single classification have a positive contribution in combination with other features.

**Figure 8:**
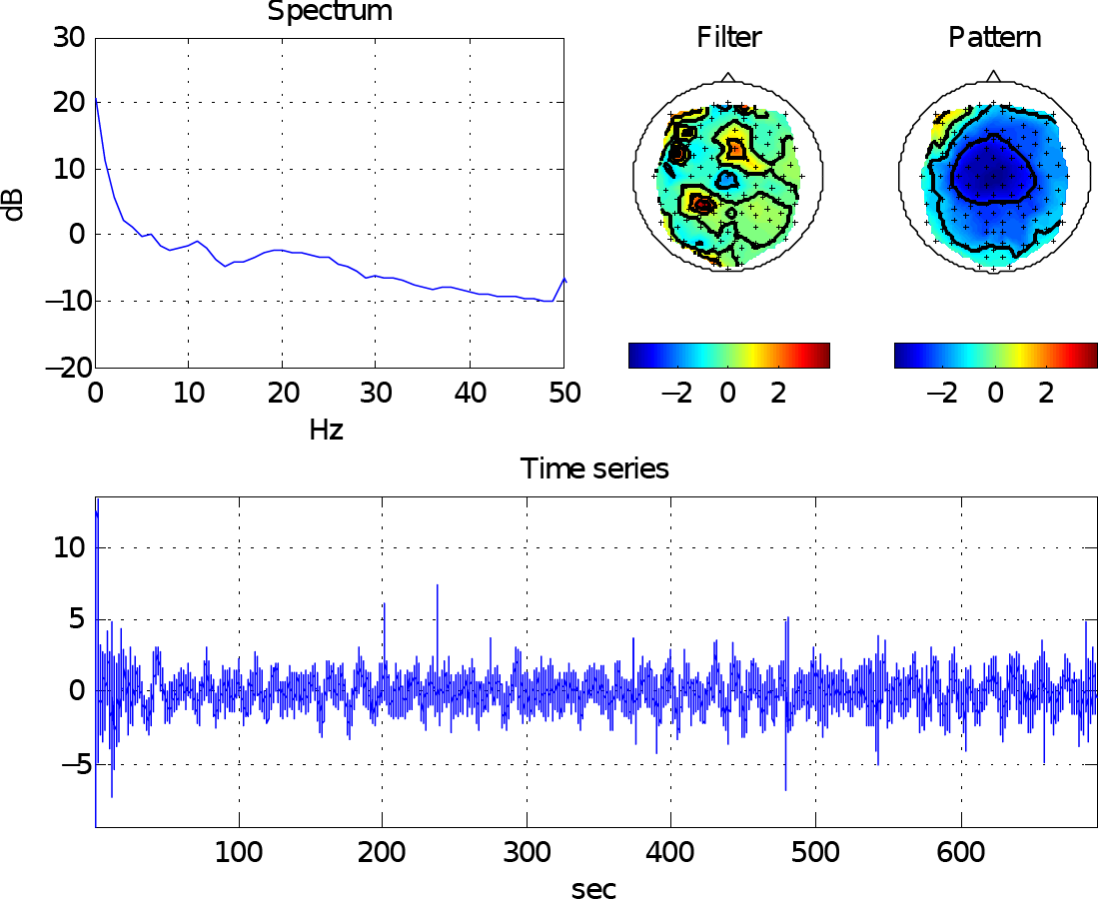
**Example for a misclassified component**. Mixed component that combines central alpha activity and slow Mayer Waves [35]. The human expert considered the mixed component a neural source, but the classifier labeld it as an artifact.

Looking at the complete test set of 1080 components, 75 of them were misclassified as artifacts and 21 components were misclassified as neural sources. A detailed visual analysis of these cases reveals, that most of them were mixed components that contained both, artifacts and brain activity. Out of the 21 components which were misclassified as neural activity only two were eye movements and none were blinks. In some rare cases, examples which had been mislabeled by the expert could be identified. Figure 8 shows an example of a misclassified mixed component.

To quantify the classification performance on muscle artifacts, we asked one expert to review the 574 artifactual components of the test set for muscle activity. The expert identified 388 components which contained muscle activity (which corresponds to 67.5% of the artifactual components and 17.2% of all the components). Out of the 21 artifactual components which were misclassified as neuronal components, only 12 contained muscle activity (57.1%). This indicates that muscle artifacts were handled equally well by the classifier as other types of artifacts.

The performance of a system on the classification task has to be judged in the light of the fact that inter-expert disagreements on EEG signals are often above 10% [34]. For our data, we asked one expert to re-label the 690 components of the training set, two years after the original labeling. The MSE between the new and the former rating was 13.2%. Thus, the prediction performance of our proposed classification method was comparable to the ranking of an human expert.

### Validation 2: Auditory ERP study

The classifier trained on RT data and applied to 540 components of the auditory ERP study leads to an average MSE of 14.7% only for the classification of artifacts (expert 1: 15.7%, expert 2: 13.7%). On average over both experts, 18 of the 540 components were misclassified as artifacts and 61.5 components were misclassifed as neural sources (expert 1: 12 - 73, expert 2: 24 - 50).

Table 2 also shows the classification results for every single feature and for the combined classification for the auditory ERP data. The classification performance of the three features *Range Within Pattern*, *λ *and *8-13 Hz *is comparable to those in the RT experiment. They generalize very well over different experimental setups. However, the single feature classification performance for the remaining three features, *Current Density Norm*, *Mean Local Skewness 15 s*, and *FitError*, was close to chance level. This does not imply, however, that these features are unimportant for the classification tasks in the combined feature set. To asses the relevance of each feature in the combined feature set, we trained a RLDA on the ERP data using the labels of expert 1 and report the feature weights of the weight vector - *Current Density Norm *0.139; *Range Within Pattern *0.355; *Mean Local Skewness 15 s *0.255; *λ *0.531; *8-13 Hz *-0.710; *FitError *0.059. We found that while the feature weight of *Current Density Norm *and *Mean Local Skewness 15 s *slightly decreased compared to the feature vector trained on the RT data, these were still far away from zero and thus carry information for the classification task.

### Validation 3: Application to Motor Imagery BCI

**Figure 9:**
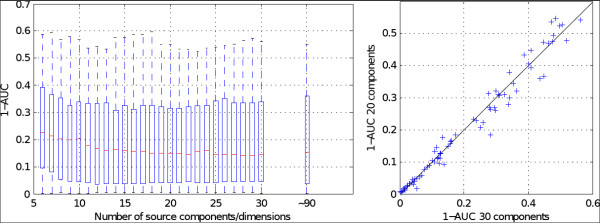
**Influence of ICA-based artifact reduction in a motor imagery BCI tested with 80 subjects**. Left: box plot of classifition errors (1-AUC) against the number of remaining independent sources compared with no artifact reduction. Right: scatter plot of classification errors (1-AUC) of each subject when removing 10 source components vs. using all 30 source components.

Figure 9 (left) plots the BCI classification error (1-AUC) against the number of remaining independent components, including one entry for the standard procedure without artifact reduction. Reducing the dimensionality of the data to 30 dimensions by PCA does not affect BCI performance. Moreover, consecutively removing components does not impair BCI performance at first, as these are artifactual components according to the classifier. Performance breaks down only when a strict removing policy is applied and less than about 12 sources (out of ~90 original channels) are retained, which have been ranked as neural sources by the classifier. The ranking of the classifier was confirmed by a visual analysis of the source components. Following the ranking of very probable artifacts to less probable artifacts, the inspection resulted in clear artifactual components to components that contained mixtures of neural and artifactual activity.

Figure 9 (right) shows a scatter plot of classification errors (1-AUC) for each subject when removing 10 source components vs. using all 30 source components. For this soft policy for removing artifactual components the variance between subjects is very small, especially for subjects with good classification rates.

## Discussion

To summarize, we have constructed a subject-independent, fast, efficient linear component classification method that automates the process of tedious hand-selection of e.g. artifactual independent components. The proposed method is applicable online and generalizes to new subjects without re-calibration. It delivers physiologically interpretable results, generalizes well over different experimental setups and is not limited to a specific type of artifact. In particular, muscle artifacts and eye artifacts (besides other types) are recognized.

The proposed artifact classifier is based on six carefully constructed features that incorporate information from the spatial, the temporal and the spectral domain of the components and have been selected out of 38 features by a thorough feature selection procedure. After its construction on data from a reaction time experiment, the classifier's performance was validated on two different data sets: (1) on unseen data of a second condition of the original reaction time study - here the classifier achieved a classification error of 8.9%, while disagreement between two ratings of experts was 13.2%. (2) on unseen data of an auditory oddball ERP study - here the classifier showed a classification error of 14.7% in comparison to 10.6% of disagreement between experts. The classification error is remarkable low given that the second study has been recorded with half the number of electrodes, under a completely different paradigm, and contained a significantly higher proportion of artifactual components.

We could show that the generalization over different EEG studies is possible, which is in line with the findings of Mognon et al. [20] who demonstrated the generalization of an artifact classifier to a different laboratory and to a different paradigm. Although their method is simple and efficient, it so far does not recognize muscle artifacts.

Compared to the classification results of Halder et al. [22], who reported 8% of error for muscle artifacts and 1% error for eye artifacts, the classification error of our solution is slightly higher. A major difference between the two approaches is the way the training data was generated. Halder et al. reported, that subjects had specifically been instructed to produce a number of artifacts under controlled conditions for the classifier training. It can be speculated that such a training set contains stronger artifacts and less erroneous labels. Nevertheless, the results of Halder et al. were generated based on EEG recordings of only 16 electrodes. Without adjustments, it can only be applied to EEG recordings with 16 electrodes. In contrast, our method is applicable to different EEG setups. However, we only tested the generalization ability over different EEG studies on electrode sets that covered the whole scalp with approx. equidistant sensors. Whether the classifier is applicable to deal with EEG data recorded with further reduced electrode sets remains an open question that could be analyzed in the future.

To assess the danger of false positives introduced by our artifact detection method, we evaluated the influence of a strong artifact reduction on the classification performance of a standard motor imagery BCI task. An offline analysis of data acquired from 80 healthy subjects demonstrated that removing up to 60% of the sources (that were ranked according to their artifact classifier rating) did not impair the overall BCI classification performance. Note that we discarded the same number of components per subject in order to analyse the effect of false positives. In a practical BCI-system, it would probably be beneficial to apply a threshold on the propability of being an artifact per component instead.

While the suitability of our approach to remove large artifactual subspaces of the data is a welcome result, an open question remains that addresses the potential performance increase by careful artifact removal. Why didn't the removal of few artifactual sources improve the average motor imagery BCI performance? It is known that CSP is rather prone to outliers if the training data set is small [36]. Strategies to overcome this problem include the use of regularization methods for CSP (such as invariant CSP [37] or robust CSP [38]), the explicit removal of outlier trials or channels and regularization in the following classification step. As the standard evaluation procedures for motor imagery data contained counter measures already (channel rejection and trial rejection based on variance), and the number of training data was considerably large, the overall influence of artifacts on the motor imagery data set probably was small. Furthermore, we observed, that for subjects with very good motor imagery classification rates, artifacts did not play any role at all. We conjecture that in the other subjects, artifacts either obstructed the relevant neural activity (cases where a slight improvement by artifact removal was obtained) or artifacts played some role in the control of the BCI system (cases where artifact removal slightly reduced the performance).

In addition to the construction of an efficient, sparse and interpretable classifier, our feature-selection methodology leads to valuable insights into the question of which features are best suited for the discrimination of artifactual and neuronal source components. However, it needs to be kept in mind that while the six identified features were arguably an exceptionally suitable feature set, these features were probably not the overall optimal choice. Furthermore, the question remains if the selected features generalize to other EEG data. Single feature classification performance drops on the ERP data for three of the six features (*Current Density Norm*, *Mean Local Skewness 15 s *and *Fit Error*). However, both *Current Density Norm *and *Mean Local Skewness 15 s *carry important information in the combined classification (when used together with the other four features). Still, the non-redundant information carried by the *Current Density Norm *feature drops substantially -- a problem that may be caused by the use of a fixed matrix Γ*J_λ _*which had been determined on the RT setup of 115 electrodes. We found no obvious explanation for the importance change for the *Fit Error *feature, however.

In any case, several insights can be gained concerning the construction of a suitable feature set for the classification of artifactual components in general. First, the spatial, the temporal and the spectral domain of the components contain non-redundant information. Second, features that quantify aspects of the pattern's activity distribution, not its single values, are discriminative. Features that were ranked high in our feature selection procedure were the range within the pattern, a feature based on the simplicity of a source separation, features that analyzed the spatial frequency and a binary feature which indicates if the maximal activation is on the border of the pattern. Third, features that model the shape of the power spectrum as a 1/*f*-curve as well as the absolute spectrum in the *α *range are discriminative. Fourth, features that quantify outliers in the time series such as kurtosis, entropy, and mean local skewness, seem to be important but redundant. We analyzed 12 such features and only one obtained a high ranking in the feature selection. Last but not least, a linear classification method seems to be sufficient when the feature set is carefully constructed.

The classification difficulties of expert raters and of proposed automatic classification methods reflect the fundamental fact that any ICA-based artifact reduction method depends crucially on the quality of the source separation into clear artifactual and neuronal source components. A good source separation method avoids mixed components that contain both, neural and artifactual activity as well as arbitrary splits of a single source into several components. In the following, both type of errors are briefly discussed.

Blind source separation is a difficult problem by itself, and various approaches have been proposed to solve it (see, e.g. [39] for a review). In the context of EEG signals, the goal is to find a source separation that minimizes the amount of mixed components. The choice of TDSEP for the pre-processing of the EEG data was motivated by the ability of the algorithm to utilize temporal structure in the data. Although this is not a unique feature of TDSEP, this approach seemed to be suitable for the processing of EEG data, which is composed of multidimensional time series signals with temporal dependencies. Moreover, research indicates that methods based on second-order statistics might outperform methods based on higher-order statistics in the removal of ocular artifacts [10,22]. Although, as Fitzgibbon stated, "the quality of the separation is highly dependent on the type of contamination, the degree of contamination, and the choice of BSS algorithm" [9], a thorough test of various ICA methods is out of the scope of this paper.

The second kind of error, the arbitrary split of sources into several components, can partially be compensated by combining the ICA with a preceding PCA step for the dimensionality reduction. This procedure has the additional advantage of removing noise in the data. We chose to project the original data into a 30 dimensional space. The value of 30 was based on rough experience and on a quick visual inspection of the data, and was probably not the optimal choice. An improvement of the quality of separation might be possible by optimizing the dimensionality reduction, but the effort was not undertaken here. Future work is needed to analyze the influence of dimensionality reduction on source separation. 

To conclude, we hope that the source component classification method presented in this study delivers a substantial contribution for the BCI community and the EEG community in general, as a reliable and practical tool for the removal of artifacts. To support the community, to encourage the reproduction of our results, or allow for re-labeling of data we provide the readily trained classifier, an implementation of the feature extraction routines together with example scripts, the extracted features of the RT data, a visualization of 1770 components together with the expert labels used for the classifier training, and a visualization of components misclassified by our method (see Additional File 1 - MatlabCode; Additional File 2 - TrainComponents; Additional File 3 - TestComponents; Additional File 4 - Misclassifications).

## Competing interests

The authors declare that they have no competing interests.

## Authors' contributions

The EEG studies were performed in cooperation with colleagues mentioned in the acknowledgments section. Authors MT and SH designed and carried out the RT EEG study. MT designed and carried out the ERP study. IW and MT designed the feature extraction and feature selection algorithms and the artifact classification method. SH provided the current density norm feature method and implementation. All other implementations were carried out by IW. IW and MT analyzed and evaluated the overall methodology and wrote the manuscript. All authors proof-read and approved the final manuscript.

## Supplementary Material

Additional file 1 **MatlabCode**. A Matlab implementation of the feature extraction routines together with example scripts, the readily trained classifier and the extracted features for all the components of the RT data set. Click here for file

Additional file 2 **TrainComponents**. Visualization of the 690 independent components in the training RT data, together with the expert's labels. Click here for file

Additional file 3 **TestComponents**. Visualization of the 1080 independent components in the RT test data, together with the expert's labels. Click here for file

Additional file 4 **Misclassifications**. Visualization of the 75 + 21 misclassified components of the RT test data Click here for file

## References

[B1] del R Millán J Rupp R Mueller-Putz G Murray-Smith R Giugliemma C Tangermann M Vidaurre C Cincotti F Kübler A Leeb R Neuper C Müller KR Mattia D Combining Brain-Computer Interfaces and Assistive Technologies: State-of-the-Art and Challenges Frontiers in Neuroprosthetics 2010 4 10.3389/fnins.2010.00161 PMC2944670 20877434

[B2] Müller KR Tangermann M Dornhege G Krauledat M Curio G Blankertz B Machine Learning for real-time single-trial EEG-analysis: From brain-computer interfacing to mental state monitoring Journal of neuroscience methods 2008 167 82 90 10.1016/j.jneumeth.2007.09.022 18031824

[B3] Iwasaki M Kellinghaus C Alexopoulos AV Burgess RC Kumar AN Han YH Lüders HO Leigh RJ Effects of eyelid closure, blinks, and eye movements on the electroencephalogram Clinical Neurophysiology 2005 116 4 878 885 10.1016/j.clinph.2004.11.001 15792897

[B4] Goncharova II McFarland DJ Vaughan TM Wolpaw JR EMG contamination of EEG: spectral and topographical characteristics Clinical Neurophysiology 2003 114 1580 1593 10.1016/S1388-2457(03)00093-2 12948787

[B5] Fatourechi M Bashashati A Ward RK EBirch G EMG and EOG artifacts in brain computer interface systems: A survey Clinical Neurophysiology 2007 118 480 494 10.1016/j.clinph.2006.10.019 17169606

[B6] Croft RJ Barry RJ Removal of ocular artifact from the EEG: a review Clinical Neurophysiology 2000 30 5 19 10.1016/S0987-7053(00)00055-1 10740792

[B7] Makeig S Bell AJ Jung TP Sejnowski TJ Independent Component Analysis of Electroencephalographic Data Advances in neural information processing systems 1996 8 145 151

[B8] Jung TP Makeig S Humphries C Lee TW Mckeown MJ Iragui V Sejnowski TJ Removing electroencephalographic artifacts by blind source separation Psychophysiology 2000 37 163 178 10.1016/S0167-8760(00)00088-X 10731767

[B9] Fitzgibbon SP Powers DMW Pope KJ Clark CR Removal of EEG Noise and Artifact Using Blind Source Separation Clinical Neurophysiology 2007 24 3 232 243 10.1097/WNP.0b013e3180556926 17545826

[B10] Romero S Mañanas MA Barbanoj MJ A comparative study of automatic techniques for ocular artifact reduction in spontaneous EEG signals based on clinical target variables: A simulation case Computers in Biology and Medicine 2008 38 348 360 10.1016/j.compbiomed.2007.12.001 18222418

[B11] Crespo-Garcia M Atienza M LCantero J Muscle artifact remval from human sleep EEG by using independent component analysis Ann Biomed Eng 2008 36 467 475 10.1007/s10439-008-9442-y 18228142

[B12] McMenamin BW Shackman AJ Maxwell JS Bachhuber DRW Koppenhaver AM Greischar LL Davidson RJ Validation of ICA-based myogenic artifact correction for scalp and source-localized EEG NeuroImage 2010 49 2416 2432 10.1016/j.neuroimage.2009.10.010 19833218 PMC2818255

[B13] Barbati G Porcaro C Zappasodi F Rossini PM Tecchio F Optimization of an independent component analysis approach for artifact identifaction and removal in magnetoencephalographic signals Clinical Neurophysiology 2004 115 1220 1232 10.1016/j.clinph.2003.12.015 15066548

[B14] Delorme A Makeig S Sejnowski T Automatic artifact rejection for EEG data using high-order statistics and independent component analysis Proceedings of third international independent component analysis and blind source decomposition conference, San Diego, CA 2001 457 462

[B15] Romero S Mañanas M Riba J Giménez S Clos S Barbanoj M Evaluation of an automatic ocular filtering method for awake spontaneos EEG signals based on Independt Component Analysis26th Annual International Conference of the Engineering in Medicine and Biology Society (EMBS) 2004 925 928 10.1109/IEMBS.2004.1403311 17271830

[B16] Shoker L Sanei S Chambers J Artifact Removal from Electroencephalograms using a hybrid BSS-SVM algorithm IEEE Signal Processing Letters 2005 12 10 721 724

[B17] James CJ Gibson OJ Temporally constrained ICA: an application to artifact rejection in electromagnetic brain signal analysis IEEE Trans Biomed Eng 2003 50 1108 1116 10.1109/TBME.2003.816076 12943278

[B18] Joyce CA Gorodnitsky IF Kutas M Automatic removal of eye movement and blink artifacts from EEG data using blind component separation Psychophysiology 2004 41 331 325 10.1111/j.1469-8986.2003.00141.x 15032997

[B19] Viola FC Thorne J Edmonds B Schneider T Eichele T Debener S Semi-automatic identification of independent components representing EEG artifact Clinical Neurophysiology 2009 120 868 877 10.1016/j.clinph.2009.01.015 19345611

[B20] Mognon A Jovicich J Bruzzone L Buiatti M ADJUST: An automatic EEG artifact detector based on the joint use of spatial and temporal features Psychophysiology 2010 1 12 10.1111/j.1469-8986.2010.01061.x 20636297

[B21] LeVan P Urrestarazu E Gotman J A system for automatic artifact removal in ictal scalp EEG based on independent component analysis and Bayesian classification Clinical Neurophysiology 2006 117 912 927 10.1016/j.clinph.2005.12.013 16458594

[B22] Halder S Bensch M Mellinger J Bogdan M Kübler A Birbaumer N Rosenstiel W Online Artifact Removal for Brain-Computer Interfaces Using Support Vector Machines and Blind Source Separation Computational Intelligence and Neuroscience 2007 7 3 1 10 10.1155/2007/82069 PMC2234090 18288259

[B23] Ziehe A Laskov P Nolte G Müller KR A Fast Algorithm for Joint Diagonalization with Non-orthogonal Transformations and its Application to Blind Source Separation Journal of Machine Learning Research 2004 5 801 818

[B24] Blankertz B Lemm S Treder MS Haufe S Müller KR Single-trial analysis and classification of ERP components - a tutorial NeuroImage 2011 in press 10.1016/j.neuroimage.2010.06.048 20600976

[B25] Titchener MR T-entropy of EEG/EOG Sensitive to Sleep State International Symposium on Nonlinear Theory and Applications (NOLTA) 2006 859 862

[B26] Hämäläinen MS Ilmoniemi R Interpreting magnetic fields of the brain: minimum-norm estimates Med Biol Eng Comput 1994 32 35 42 10.1007/BF02512476 8182960

[B27] Haufe S Nikulin VV Ziehe A Müller KR Nolte G Combining sparsity and rotational invariance in EEG/MEG source reconstruction NeuroImage 2008 42 726 738 10.1016/j.neuroimage.2008.04.246 18583157

[B28] Bennett KP Mangasarian OL Robust linear programming discrimination of two linearly inseparable sets Optimizaion Methods and Software 1994

[B29] Chang CC Lin CJ LIBSVM. a library for support vector machines 2001

[B30] Ledoit O Wolf M A well-conditioned estimator for large-dimensional covariance matrices Journal of Multivariate Analysis 2004 88 2 365 411 10.1016/S0047-259X(03)00096-4

[B31] Schäfer J Strimmer K A Shrinkage Approach to Large-Scale Covariance Matrix Estimation and Implications for Functional Genomics Statistical Applications in Genetics and Molecular Biology 2005 4 32 10.2202/1544-6115.1175 16646851

[B32] Blankertz B Tomioka R Lemm S Kawanabe M Müller KR Optimizing spatial filters for robust EEG single-trial analysis IEEE Signal Proc Magazine 2008 25 41 56

[B33] Blankertz B Sannelli C Halder S Hammer EM Kübler A Müller KR Curio G Dickhaus T Neurophysiological predictor of SMR-based BCI performance NeuroImage 2010 51 4 1303 1309 10.1016/j.neuroimage.2010.03.022 20303409

[B34] Klekowicz H Malinow U Niemcewi S Wakarow A Wolynczyk-Gmaj D Piotrowski T Durka P Automatic analysis of sleep EEG Frontiers in Neuroinformatics. Conference Abstract. Neuroinformatics 2008

[B35] Lugaresi E Coccagna G Mantovani M Lebrun R Some periodic phenomena arising during drowsiness and sleeping in humans Electroenceph clin Neurophysiol 1972 32 701 705 10.1016/0013-4694(72)90106-X 4121520

[B36] Krauledat M Dornhege G Blankertz B Müller KR Robustifying EEG data analysis by removing outliers Chaos and Complexity Letters 2007 2 3 259 274

[B37] Blankertz B Kawanabe M Tomioka R Hohlefeld F Nikulin V Müller KR Platt J, Koller D, Singer Y, Roweis S Invariant Common Spatial Patterns: Alleviating Nonstationarities in Brain-Computer Interfacing Advances in Neural Information Processing Systems 20 2008 Cambridge, MA: MIT Press 113 120

[B38] Kawanabe M Vidaurre C Scholler S Blankertz B Müller KR Robust Common Spatial Filters with a Maxmin Approach EMBS-Conference 2009 2470 2473 10.1109/IEMBS.2009.5334786 19964963

[B39] Choi S Cichocki A Park HM Lee SY Blind Source Separation and Independent Component Analysis: A Review Neural Information Processing - Letters and Reviews 2005 6 1 57

