# Supplementary figures and images for: Automatic Classification of Artifactual ICA-Components for Artifact Removal in EEG Signals (part 2 of 5)
Source: Behav Brain Funct. 2011 Aug 2;7:30. doi: 10.1186/1744-9081-7-30 (PMC3175453; doi:10.1186/1744-9081-7-30)

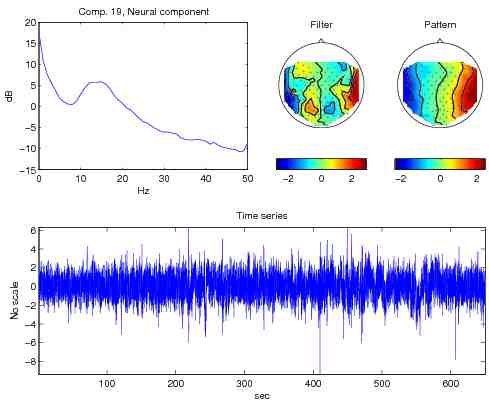

Supplement: Additional file 2 — TrainComponents. Visualization of the 690 independent components in the training RT data, together with the expert's labels. [file 1744-9081-7-30-S2.GZ › components_train/comp19.jpg]

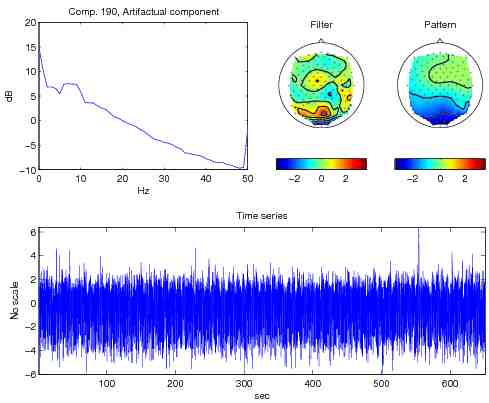

Supplement: Additional file 2 — TrainComponents. Visualization of the 690 independent components in the training RT data, together with the expert's labels. [file 1744-9081-7-30-S2.GZ › components_train/comp190.jpg]

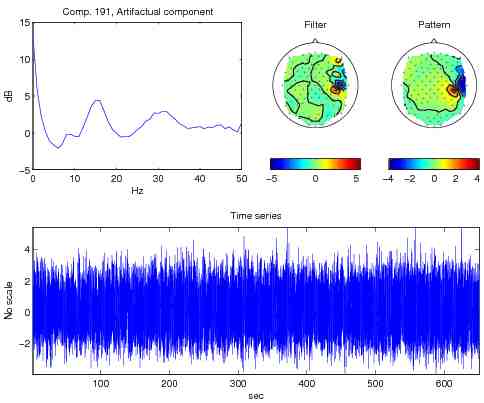

Supplement: Additional file 2 — TrainComponents. Visualization of the 690 independent components in the training RT data, together with the expert's labels. [file 1744-9081-7-30-S2.GZ › components_train/comp191.jpg]

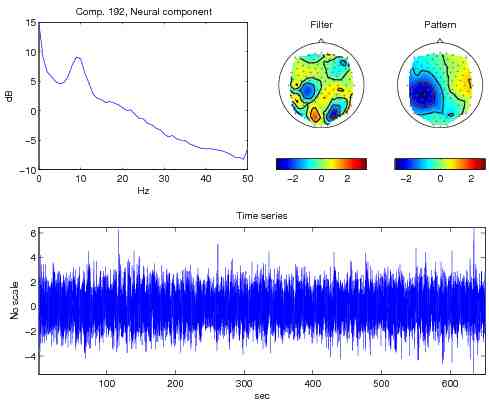

Supplement: Additional file 2 — TrainComponents. Visualization of the 690 independent components in the training RT data, together with the expert's labels. [file 1744-9081-7-30-S2.GZ › components_train/comp192.jpg]

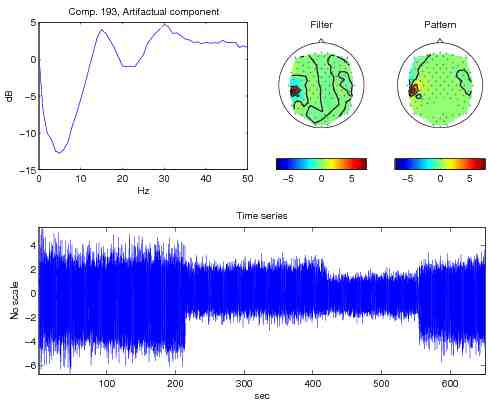

Supplement: Additional file 2 — TrainComponents. Visualization of the 690 independent components in the training RT data, together with the expert's labels. [file 1744-9081-7-30-S2.GZ › components_train/comp193.jpg]

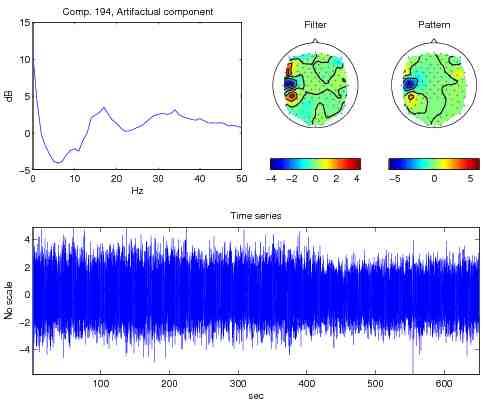

Supplement: Additional file 2 — TrainComponents. Visualization of the 690 independent components in the training RT data, together with the expert's labels. [file 1744-9081-7-30-S2.GZ › components_train/comp194.jpg]

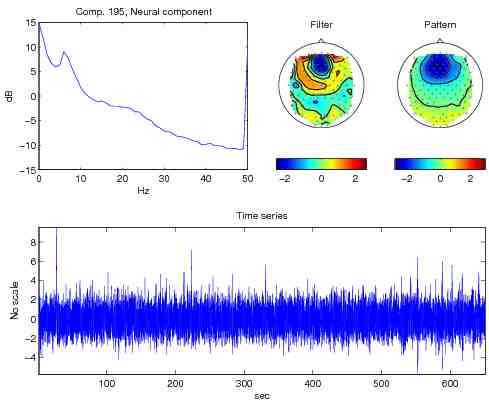

Supplement: Additional file 2 — TrainComponents. Visualization of the 690 independent components in the training RT data, together with the expert's labels. [file 1744-9081-7-30-S2.GZ › components_train/comp195.jpg]

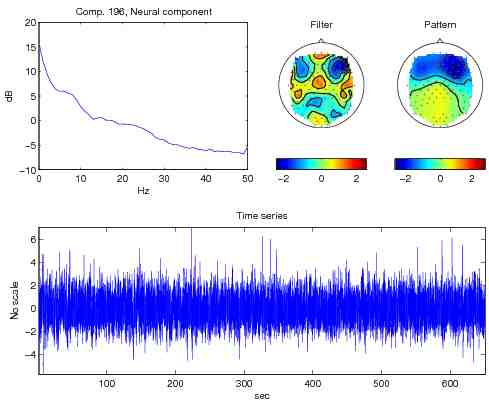

Supplement: Additional file 2 — TrainComponents. Visualization of the 690 independent components in the training RT data, together with the expert's labels. [file 1744-9081-7-30-S2.GZ › components_train/comp196.jpg]

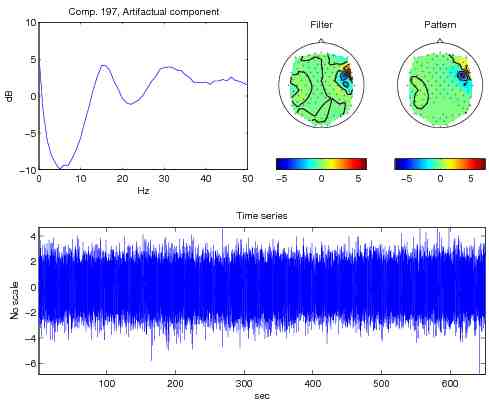

Supplement: Additional file 2 — TrainComponents. Visualization of the 690 independent components in the training RT data, together with the expert's labels. [file 1744-9081-7-30-S2.GZ › components_train/comp197.jpg]

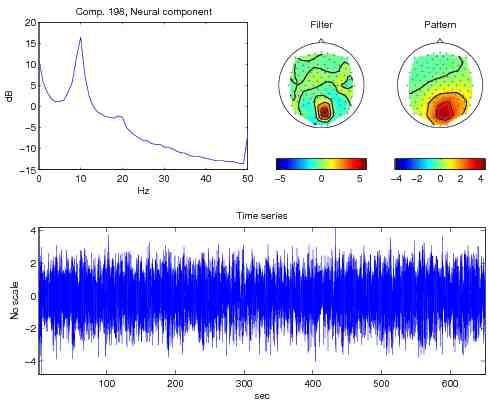

Supplement: Additional file 2 — TrainComponents. Visualization of the 690 independent components in the training RT data, together with the expert's labels. [file 1744-9081-7-30-S2.GZ › components_train/comp198.jpg]

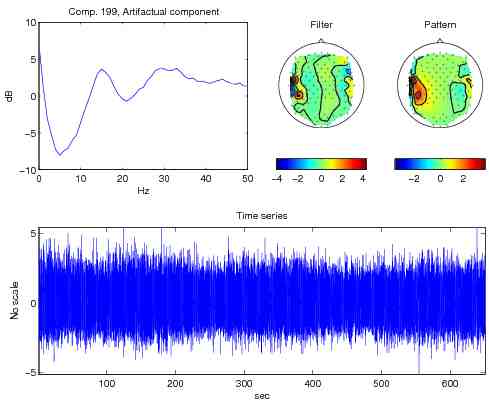

Supplement: Additional file 2 — TrainComponents. Visualization of the 690 independent components in the training RT data, together with the expert's labels. [file 1744-9081-7-30-S2.GZ › components_train/comp199.jpg]

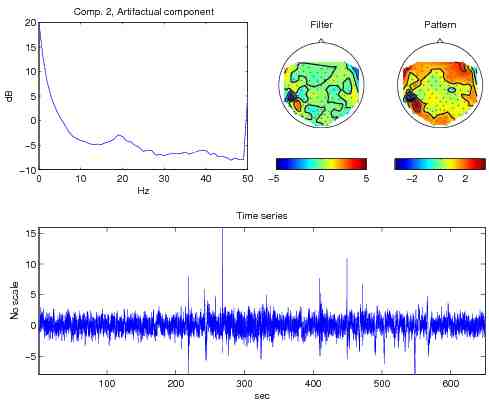

Supplement: Additional file 2 — TrainComponents. Visualization of the 690 independent components in the training RT data, together with the expert's labels. [file 1744-9081-7-30-S2.GZ › components_train/comp2.jpg]

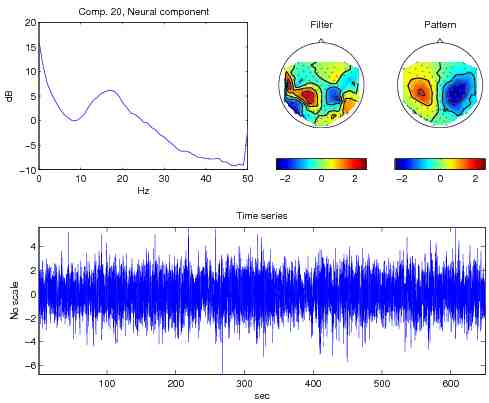

Supplement: Additional file 2 — TrainComponents. Visualization of the 690 independent components in the training RT data, together with the expert's labels. [file 1744-9081-7-30-S2.GZ › components_train/comp20.jpg]

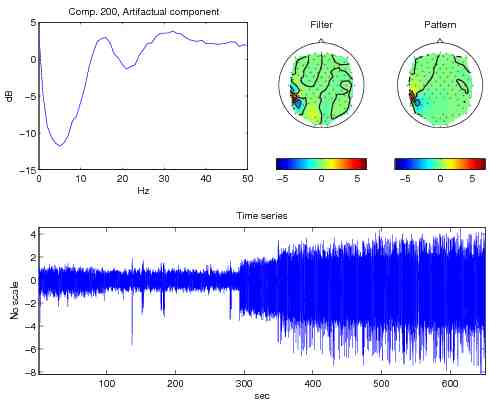

Supplement: Additional file 2 — TrainComponents. Visualization of the 690 independent components in the training RT data, together with the expert's labels. [file 1744-9081-7-30-S2.GZ › components_train/comp200.jpg]

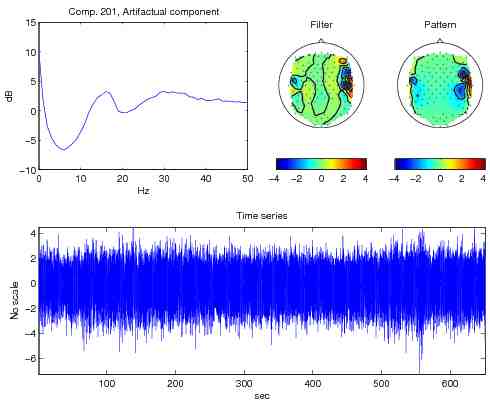

Supplement: Additional file 2 — TrainComponents. Visualization of the 690 independent components in the training RT data, together with the expert's labels. [file 1744-9081-7-30-S2.GZ › components_train/comp201.jpg]

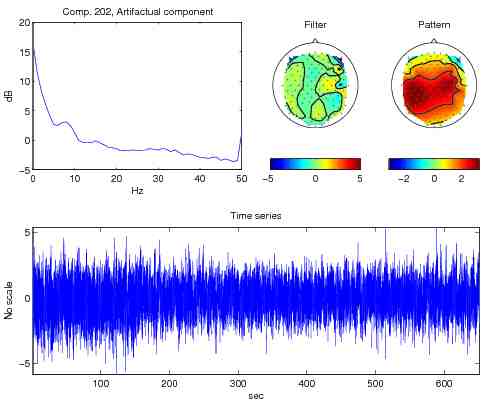

Supplement: Additional file 2 — TrainComponents. Visualization of the 690 independent components in the training RT data, together with the expert's labels. [file 1744-9081-7-30-S2.GZ › components_train/comp202.jpg]

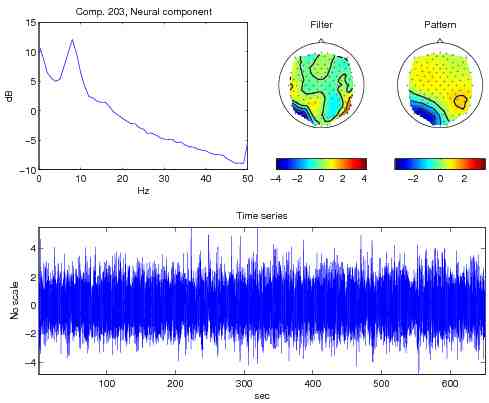

Supplement: Additional file 2 — TrainComponents. Visualization of the 690 independent components in the training RT data, together with the expert's labels. [file 1744-9081-7-30-S2.GZ › components_train/comp203.jpg]

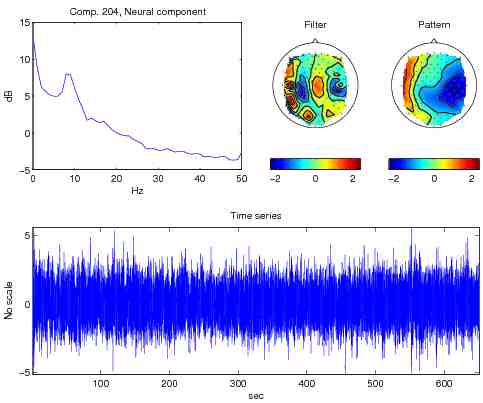

Supplement: Additional file 2 — TrainComponents. Visualization of the 690 independent components in the training RT data, together with the expert's labels. [file 1744-9081-7-30-S2.GZ › components_train/comp204.jpg]

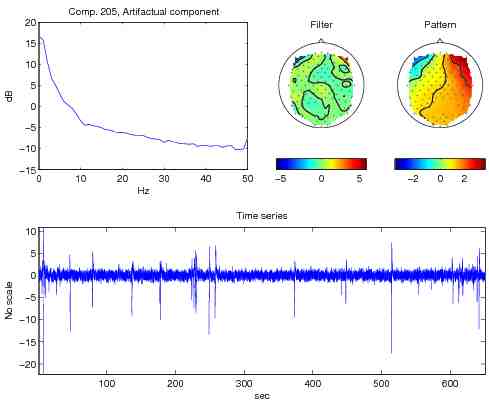

Supplement: Additional file 2 — TrainComponents. Visualization of the 690 independent components in the training RT data, together with the expert's labels. [file 1744-9081-7-30-S2.GZ › components_train/comp205.jpg]

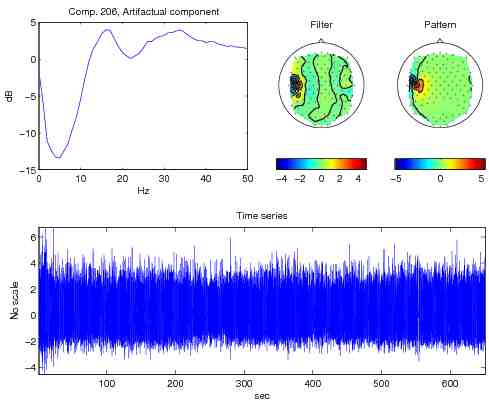

Supplement: Additional file 2 — TrainComponents. Visualization of the 690 independent components in the training RT data, together with the expert's labels. [file 1744-9081-7-30-S2.GZ › components_train/comp206.jpg]

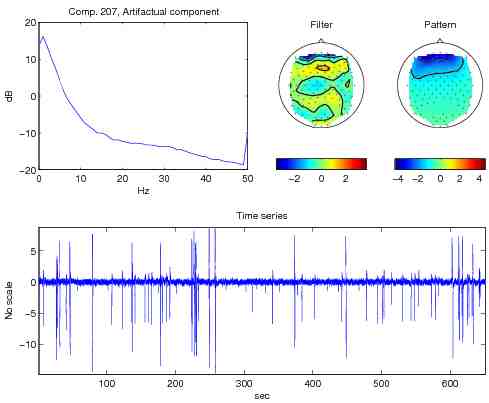

Supplement: Additional file 2 — TrainComponents. Visualization of the 690 independent components in the training RT data, together with the expert's labels. [file 1744-9081-7-30-S2.GZ › components_train/comp207.jpg]

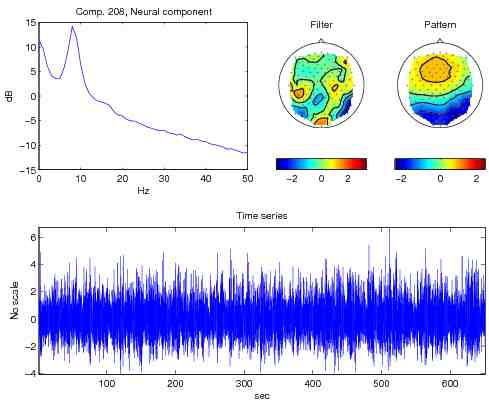

Supplement: Additional file 2 — TrainComponents. Visualization of the 690 independent components in the training RT data, together with the expert's labels. [file 1744-9081-7-30-S2.GZ › components_train/comp208.jpg]

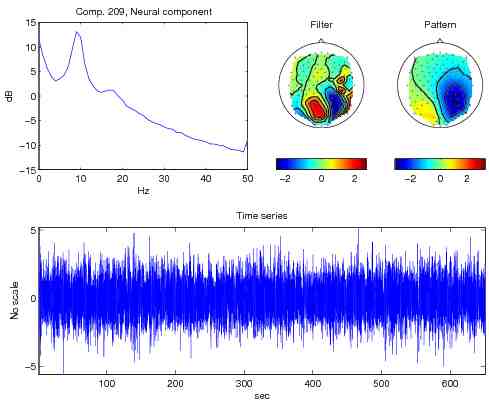

Supplement: Additional file 2 — TrainComponents. Visualization of the 690 independent components in the training RT data, together with the expert's labels. [file 1744-9081-7-30-S2.GZ › components_train/comp209.jpg]

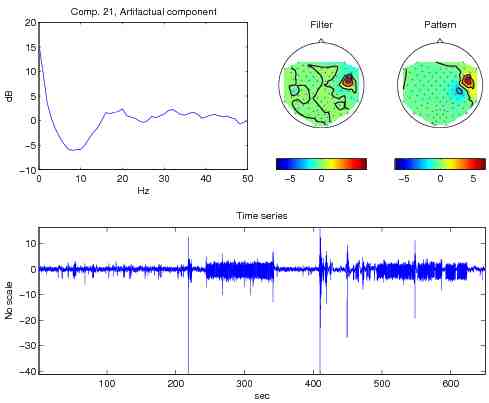

Supplement: Additional file 2 — TrainComponents. Visualization of the 690 independent components in the training RT data, together with the expert's labels. [file 1744-9081-7-30-S2.GZ › components_train/comp21.jpg]

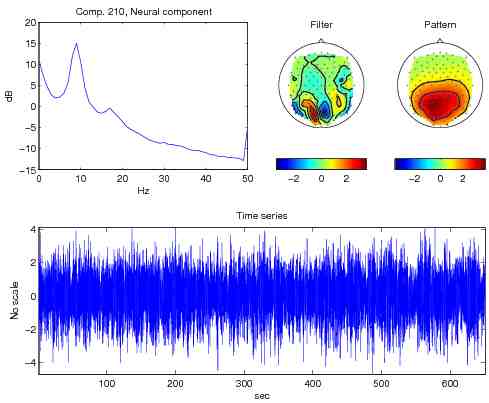

Supplement: Additional file 2 — TrainComponents. Visualization of the 690 independent components in the training RT data, together with the expert's labels. [file 1744-9081-7-30-S2.GZ › components_train/comp210.jpg]

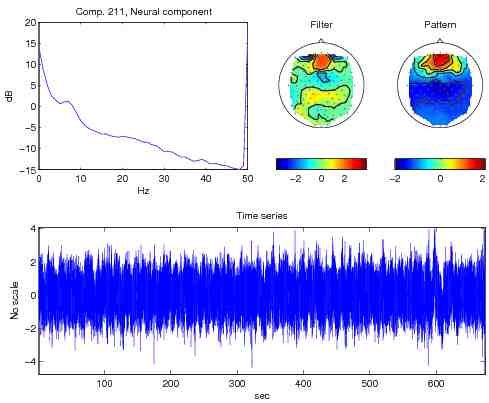

Supplement: Additional file 2 — TrainComponents. Visualization of the 690 independent components in the training RT data, together with the expert's labels. [file 1744-9081-7-30-S2.GZ › components_train/comp211.jpg]

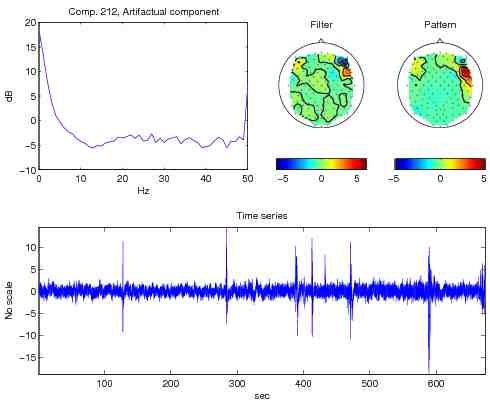

Supplement: Additional file 2 — TrainComponents. Visualization of the 690 independent components in the training RT data, together with the expert's labels. [file 1744-9081-7-30-S2.GZ › components_train/comp212.jpg]

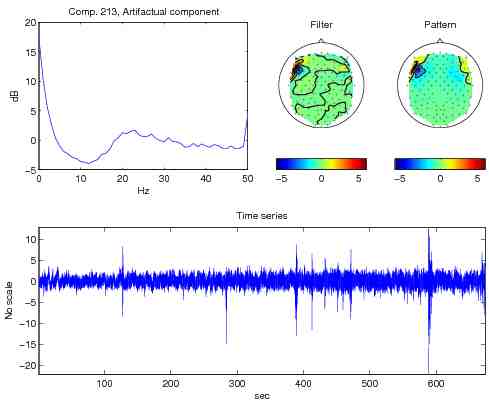

Supplement: Additional file 2 — TrainComponents. Visualization of the 690 independent components in the training RT data, together with the expert's labels. [file 1744-9081-7-30-S2.GZ › components_train/comp213.jpg]

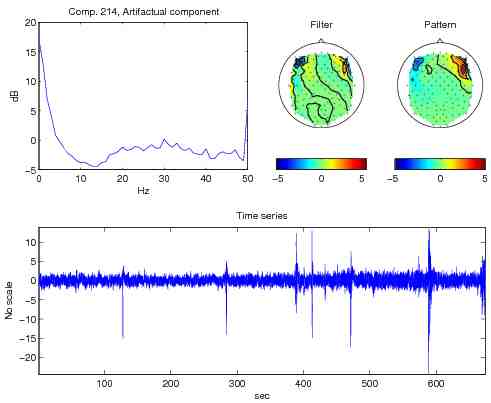

Supplement: Additional file 2 — TrainComponents. Visualization of the 690 independent components in the training RT data, together with the expert's labels. [file 1744-9081-7-30-S2.GZ › components_train/comp214.jpg]

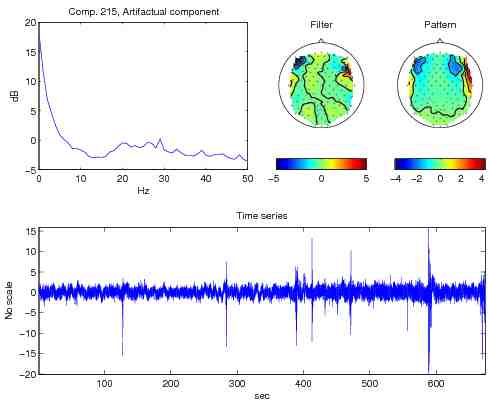

Supplement: Additional file 2 — TrainComponents. Visualization of the 690 independent components in the training RT data, together with the expert's labels. [file 1744-9081-7-30-S2.GZ › components_train/comp215.jpg]

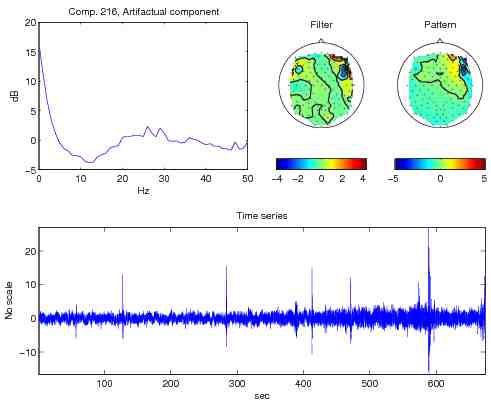

Supplement: Additional file 2 — TrainComponents. Visualization of the 690 independent components in the training RT data, together with the expert's labels. [file 1744-9081-7-30-S2.GZ › components_train/comp216.jpg]

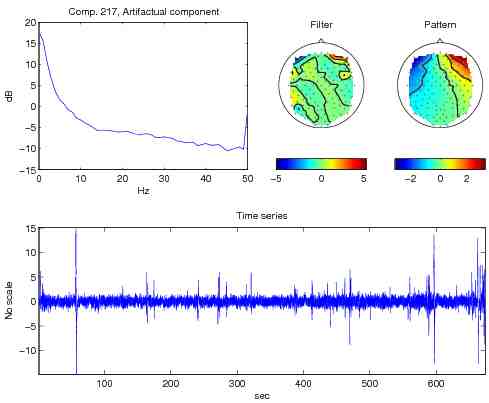

Supplement: Additional file 2 — TrainComponents. Visualization of the 690 independent components in the training RT data, together with the expert's labels. [file 1744-9081-7-30-S2.GZ › components_train/comp217.jpg]

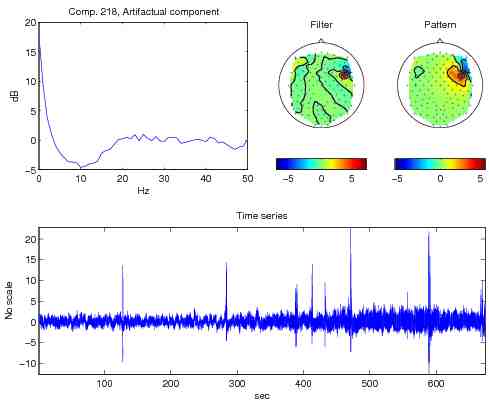

Supplement: Additional file 2 — TrainComponents. Visualization of the 690 independent components in the training RT data, together with the expert's labels. [file 1744-9081-7-30-S2.GZ › components_train/comp218.jpg]

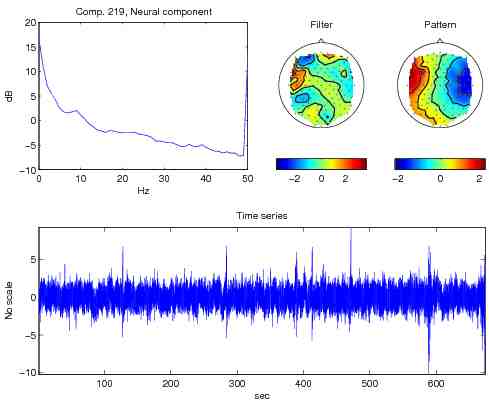

Supplement: Additional file 2 — TrainComponents. Visualization of the 690 independent components in the training RT data, together with the expert's labels. [file 1744-9081-7-30-S2.GZ › components_train/comp219.jpg]

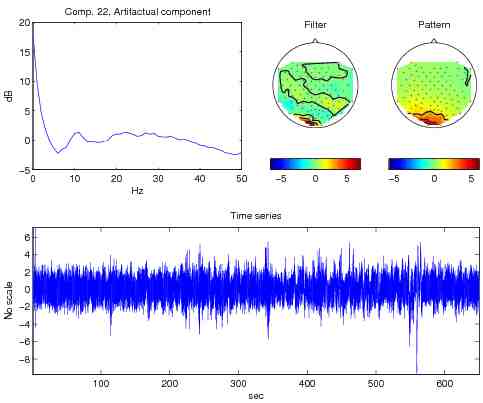

Supplement: Additional file 2 — TrainComponents. Visualization of the 690 independent components in the training RT data, together with the expert's labels. [file 1744-9081-7-30-S2.GZ › components_train/comp22.jpg]

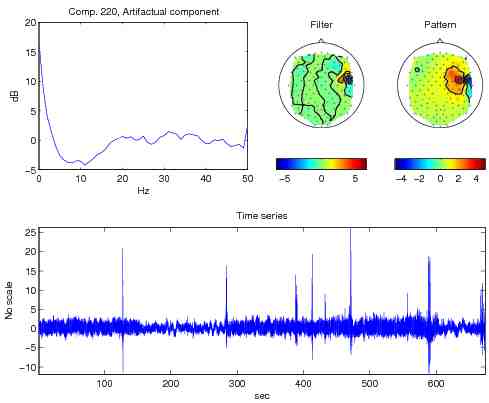

Supplement: Additional file 2 — TrainComponents. Visualization of the 690 independent components in the training RT data, together with the expert's labels. [file 1744-9081-7-30-S2.GZ › components_train/comp220.jpg]

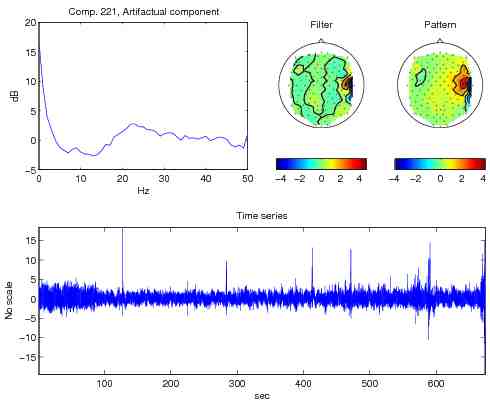

Supplement: Additional file 2 — TrainComponents. Visualization of the 690 independent components in the training RT data, together with the expert's labels. [file 1744-9081-7-30-S2.GZ › components_train/comp221.jpg]

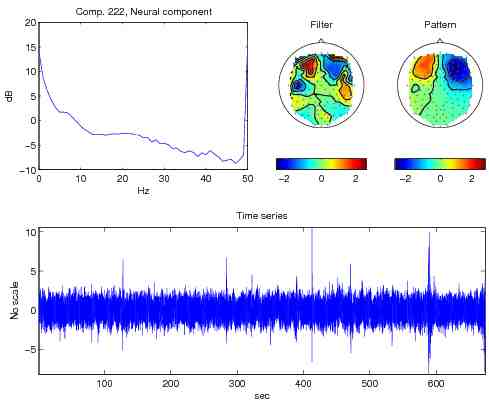

Supplement: Additional file 2 — TrainComponents. Visualization of the 690 independent components in the training RT data, together with the expert's labels. [file 1744-9081-7-30-S2.GZ › components_train/comp222.jpg]

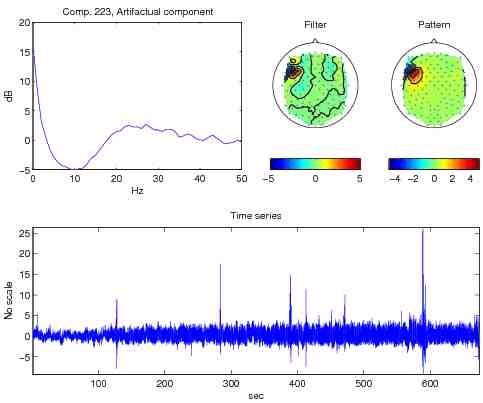

Supplement: Additional file 2 — TrainComponents. Visualization of the 690 independent components in the training RT data, together with the expert's labels. [file 1744-9081-7-30-S2.GZ › components_train/comp223.jpg]

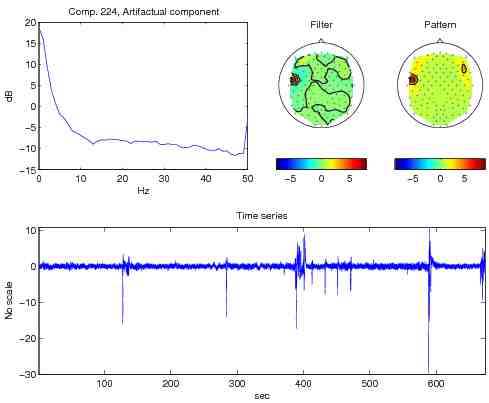

Supplement: Additional file 2 — TrainComponents. Visualization of the 690 independent components in the training RT data, together with the expert's labels. [file 1744-9081-7-30-S2.GZ › components_train/comp224.jpg]

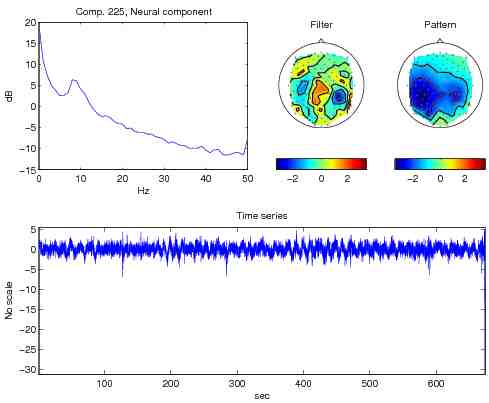

Supplement: Additional file 2 — TrainComponents. Visualization of the 690 independent components in the training RT data, together with the expert's labels. [file 1744-9081-7-30-S2.GZ › components_train/comp225.jpg]

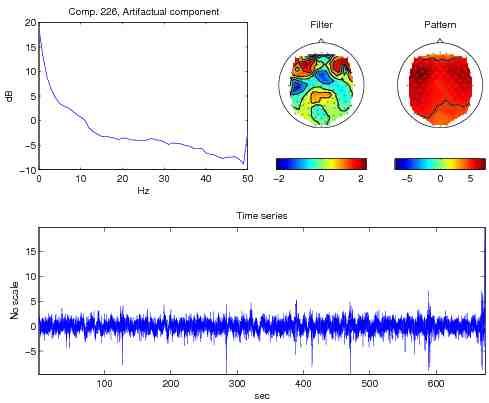

Supplement: Additional file 2 — TrainComponents. Visualization of the 690 independent components in the training RT data, together with the expert's labels. [file 1744-9081-7-30-S2.GZ › components_train/comp226.jpg]

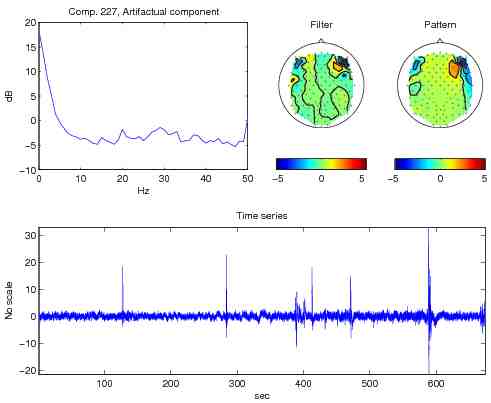

Supplement: Additional file 2 — TrainComponents. Visualization of the 690 independent components in the training RT data, together with the expert's labels. [file 1744-9081-7-30-S2.GZ › components_train/comp227.jpg]

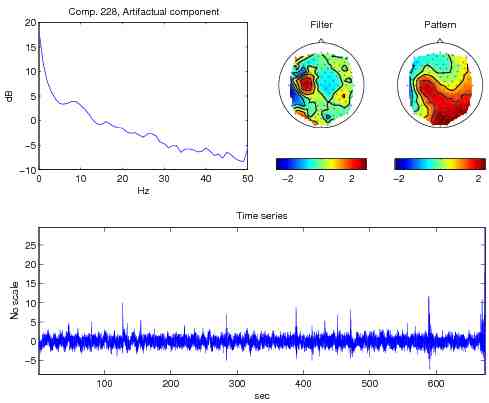

Supplement: Additional file 2 — TrainComponents. Visualization of the 690 independent components in the training RT data, together with the expert's labels. [file 1744-9081-7-30-S2.GZ › components_train/comp228.jpg]

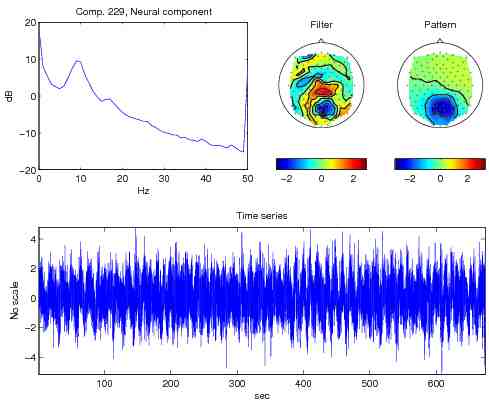

Supplement: Additional file 2 — TrainComponents. Visualization of the 690 independent components in the training RT data, together with the expert's labels. [file 1744-9081-7-30-S2.GZ › components_train/comp229.jpg]

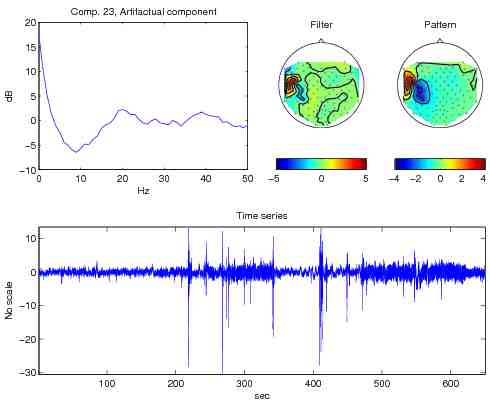

Supplement: Additional file 2 — TrainComponents. Visualization of the 690 independent components in the training RT data, together with the expert's labels. [file 1744-9081-7-30-S2.GZ › components_train/comp23.jpg]

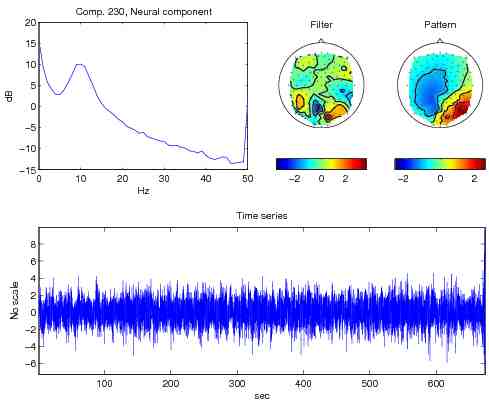

Supplement: Additional file 2 — TrainComponents. Visualization of the 690 independent components in the training RT data, together with the expert's labels. [file 1744-9081-7-30-S2.GZ › components_train/comp230.jpg]

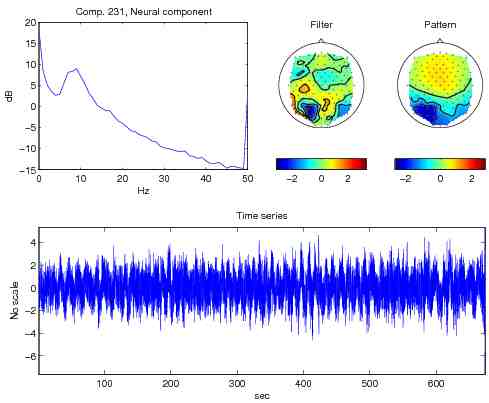

Supplement: Additional file 2 — TrainComponents. Visualization of the 690 independent components in the training RT data, together with the expert's labels. [file 1744-9081-7-30-S2.GZ › components_train/comp231.jpg]

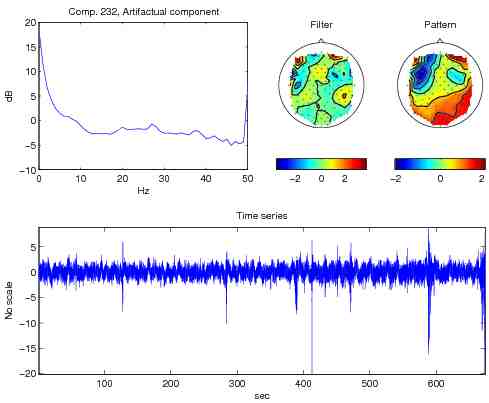

Supplement: Additional file 2 — TrainComponents. Visualization of the 690 independent components in the training RT data, together with the expert's labels. [file 1744-9081-7-30-S2.GZ › components_train/comp232.jpg]

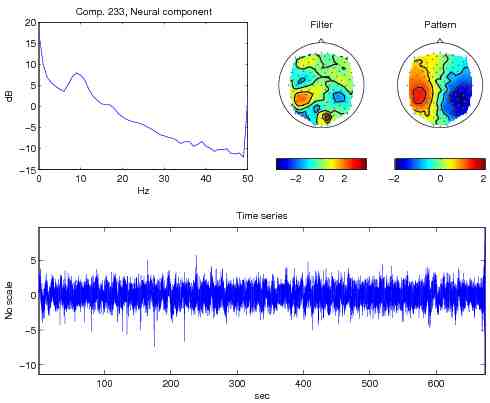

Supplement: Additional file 2 — TrainComponents. Visualization of the 690 independent components in the training RT data, together with the expert's labels. [file 1744-9081-7-30-S2.GZ › components_train/comp233.jpg]

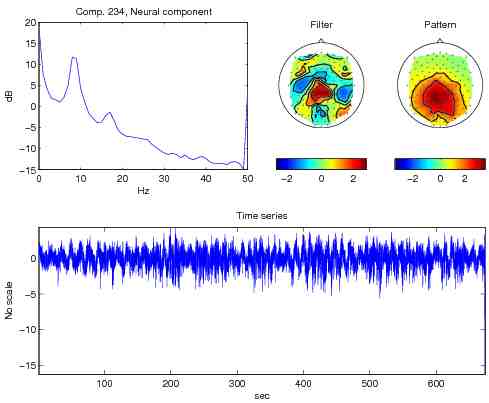

Supplement: Additional file 2 — TrainComponents. Visualization of the 690 independent components in the training RT data, together with the expert's labels. [file 1744-9081-7-30-S2.GZ › components_train/comp234.jpg]

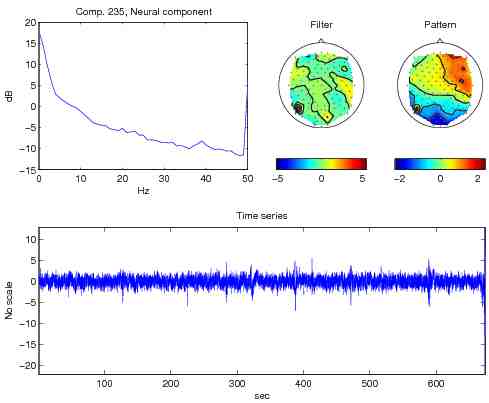

Supplement: Additional file 2 — TrainComponents. Visualization of the 690 independent components in the training RT data, together with the expert's labels. [file 1744-9081-7-30-S2.GZ › components_train/comp235.jpg]

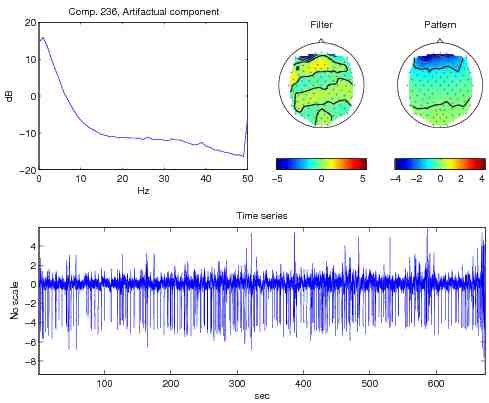

Supplement: Additional file 2 — TrainComponents. Visualization of the 690 independent components in the training RT data, together with the expert's labels. [file 1744-9081-7-30-S2.GZ › components_train/comp236.jpg]

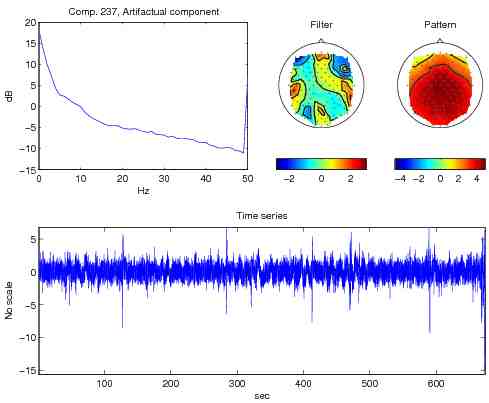

Supplement: Additional file 2 — TrainComponents. Visualization of the 690 independent components in the training RT data, together with the expert's labels. [file 1744-9081-7-30-S2.GZ › components_train/comp237.jpg]

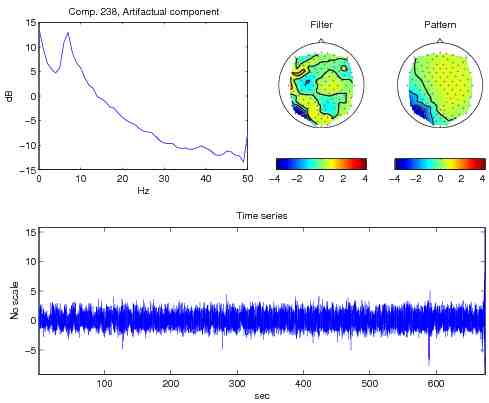

Supplement: Additional file 2 — TrainComponents. Visualization of the 690 independent components in the training RT data, together with the expert's labels. [file 1744-9081-7-30-S2.GZ › components_train/comp238.jpg]

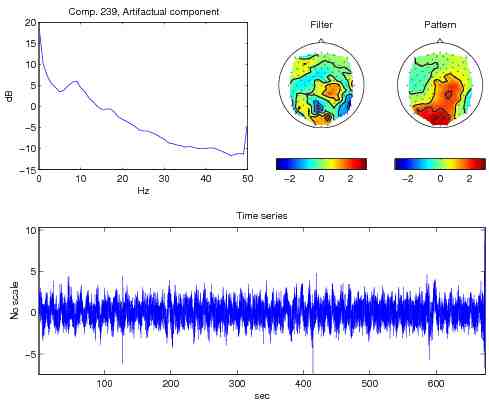

Supplement: Additional file 2 — TrainComponents. Visualization of the 690 independent components in the training RT data, together with the expert's labels. [file 1744-9081-7-30-S2.GZ › components_train/comp239.jpg]

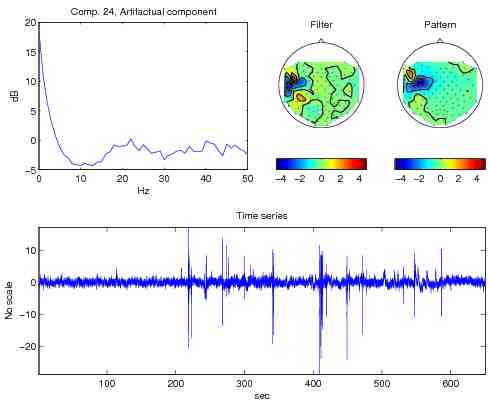

Supplement: Additional file 2 — TrainComponents. Visualization of the 690 independent components in the training RT data, together with the expert's labels. [file 1744-9081-7-30-S2.GZ › components_train/comp24.jpg]

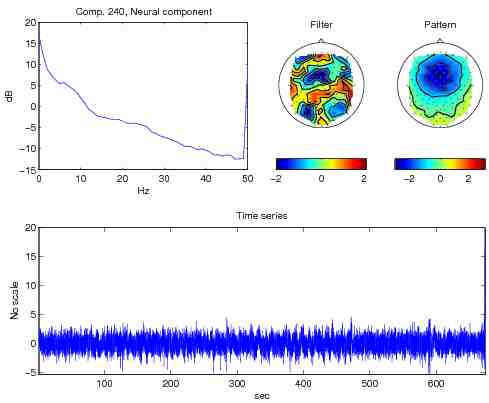

Supplement: Additional file 2 — TrainComponents. Visualization of the 690 independent components in the training RT data, together with the expert's labels. [file 1744-9081-7-30-S2.GZ › components_train/comp240.jpg]

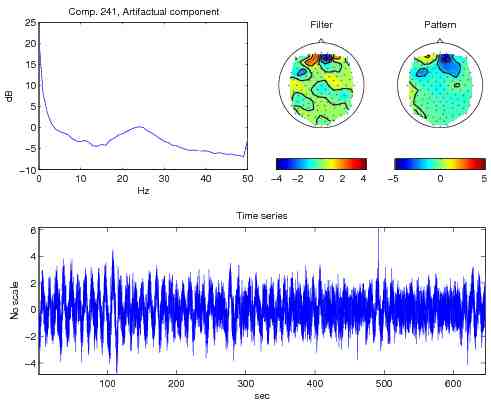

Supplement: Additional file 2 — TrainComponents. Visualization of the 690 independent components in the training RT data, together with the expert's labels. [file 1744-9081-7-30-S2.GZ › components_train/comp241.jpg]

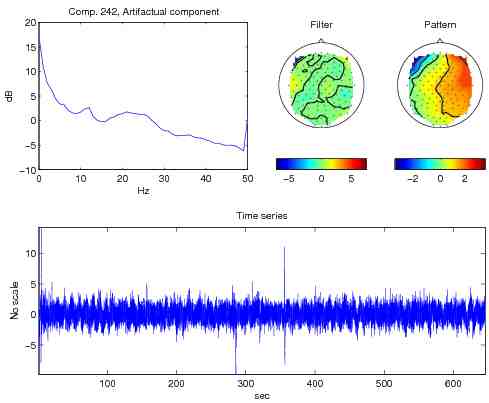

Supplement: Additional file 2 — TrainComponents. Visualization of the 690 independent components in the training RT data, together with the expert's labels. [file 1744-9081-7-30-S2.GZ › components_train/comp242.jpg]

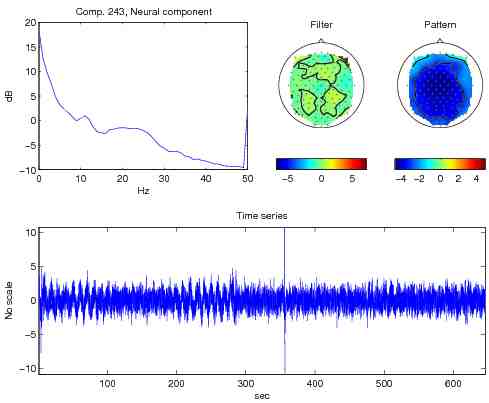

Supplement: Additional file 2 — TrainComponents. Visualization of the 690 independent components in the training RT data, together with the expert's labels. [file 1744-9081-7-30-S2.GZ › components_train/comp243.jpg]

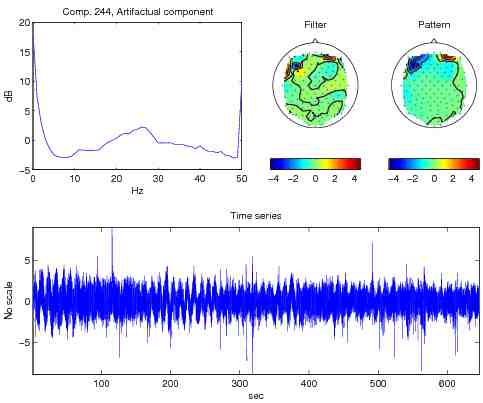

Supplement: Additional file 2 — TrainComponents. Visualization of the 690 independent components in the training RT data, together with the expert's labels. [file 1744-9081-7-30-S2.GZ › components_train/comp244.jpg]

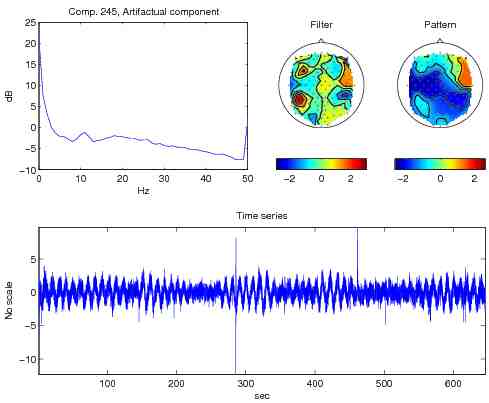

Supplement: Additional file 2 — TrainComponents. Visualization of the 690 independent components in the training RT data, together with the expert's labels. [file 1744-9081-7-30-S2.GZ › components_train/comp245.jpg]

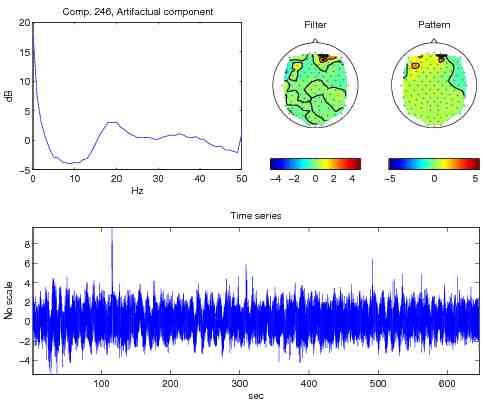

Supplement: Additional file 2 — TrainComponents. Visualization of the 690 independent components in the training RT data, together with the expert's labels. [file 1744-9081-7-30-S2.GZ › components_train/comp246.jpg]

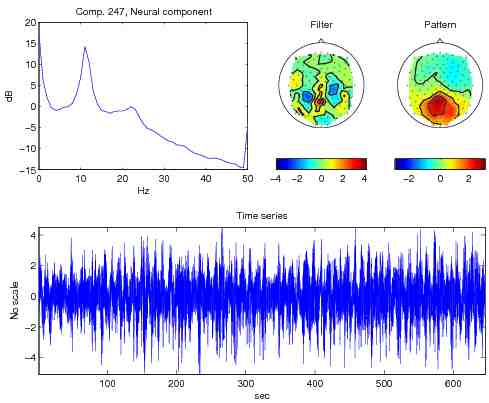

Supplement: Additional file 2 — TrainComponents. Visualization of the 690 independent components in the training RT data, together with the expert's labels. [file 1744-9081-7-30-S2.GZ › components_train/comp247.jpg]

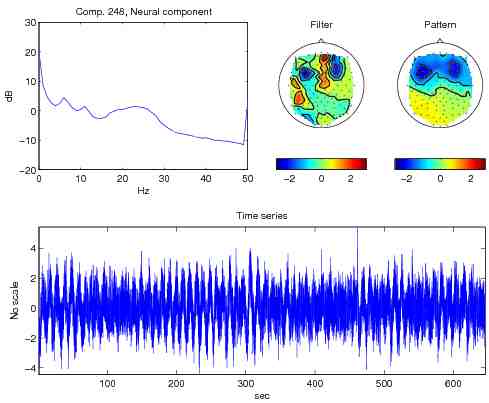

Supplement: Additional file 2 — TrainComponents. Visualization of the 690 independent components in the training RT data, together with the expert's labels. [file 1744-9081-7-30-S2.GZ › components_train/comp248.jpg]

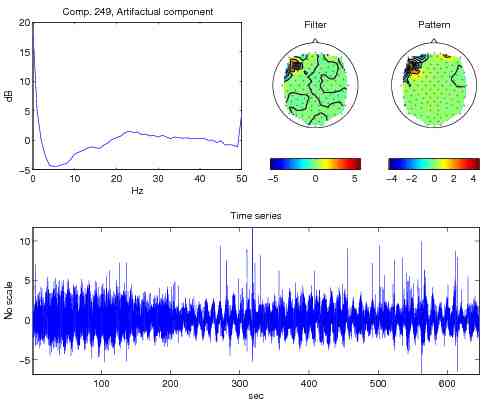

Supplement: Additional file 2 — TrainComponents. Visualization of the 690 independent components in the training RT data, together with the expert's labels. [file 1744-9081-7-30-S2.GZ › components_train/comp249.jpg]

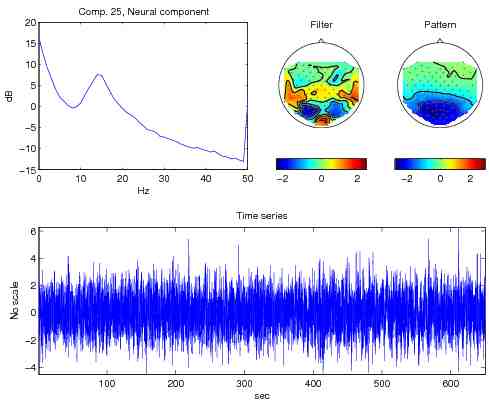

Supplement: Additional file 2 — TrainComponents. Visualization of the 690 independent components in the training RT data, together with the expert's labels. [file 1744-9081-7-30-S2.GZ › components_train/comp25.jpg]

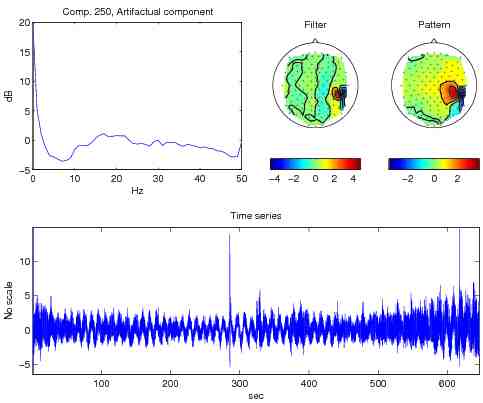

Supplement: Additional file 2 — TrainComponents. Visualization of the 690 independent components in the training RT data, together with the expert's labels. [file 1744-9081-7-30-S2.GZ › components_train/comp250.jpg]

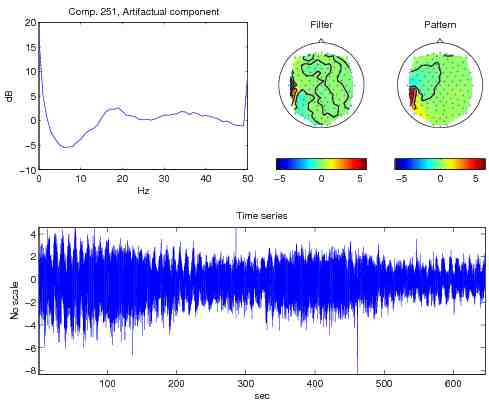

Supplement: Additional file 2 — TrainComponents. Visualization of the 690 independent components in the training RT data, together with the expert's labels. [file 1744-9081-7-30-S2.GZ › components_train/comp251.jpg]

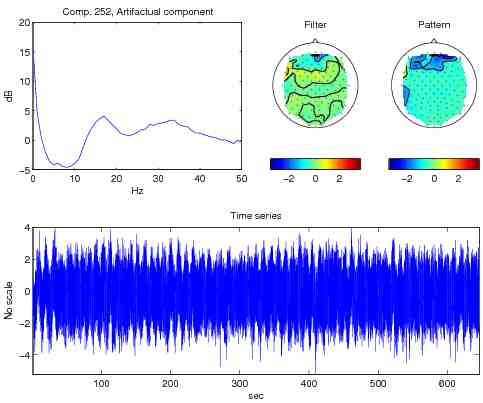

Supplement: Additional file 2 — TrainComponents. Visualization of the 690 independent components in the training RT data, together with the expert's labels. [file 1744-9081-7-30-S2.GZ › components_train/comp252.jpg]

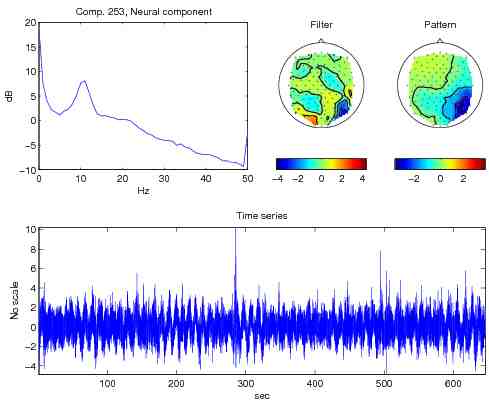

Supplement: Additional file 2 — TrainComponents. Visualization of the 690 independent components in the training RT data, together with the expert's labels. [file 1744-9081-7-30-S2.GZ › components_train/comp253.jpg]

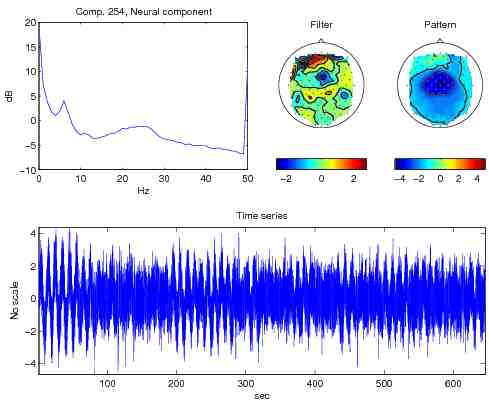

Supplement: Additional file 2 — TrainComponents. Visualization of the 690 independent components in the training RT data, together with the expert's labels. [file 1744-9081-7-30-S2.GZ › components_train/comp254.jpg]

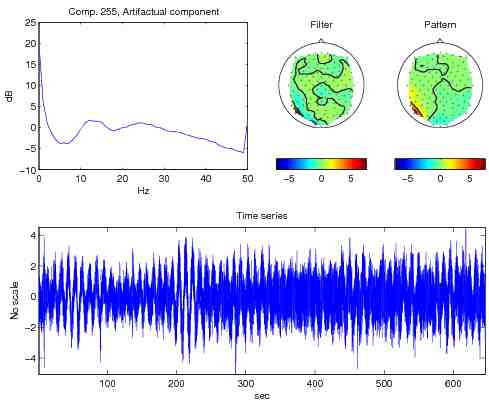

Supplement: Additional file 2 — TrainComponents. Visualization of the 690 independent components in the training RT data, together with the expert's labels. [file 1744-9081-7-30-S2.GZ › components_train/comp255.jpg]

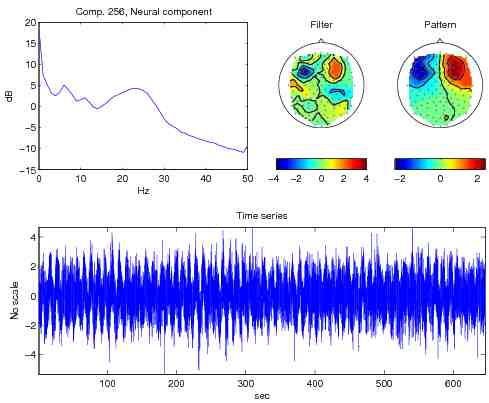

Supplement: Additional file 2 — TrainComponents. Visualization of the 690 independent components in the training RT data, together with the expert's labels. [file 1744-9081-7-30-S2.GZ › components_train/comp256.jpg]

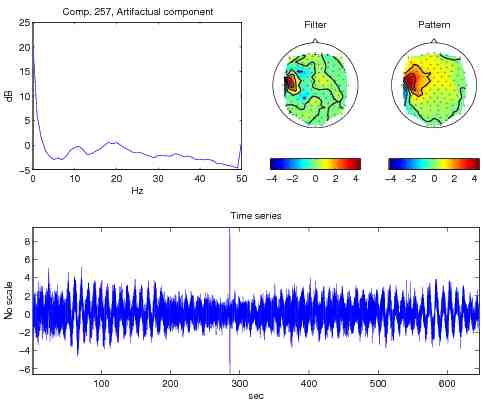

Supplement: Additional file 2 — TrainComponents. Visualization of the 690 independent components in the training RT data, together with the expert's labels. [file 1744-9081-7-30-S2.GZ › components_train/comp257.jpg]

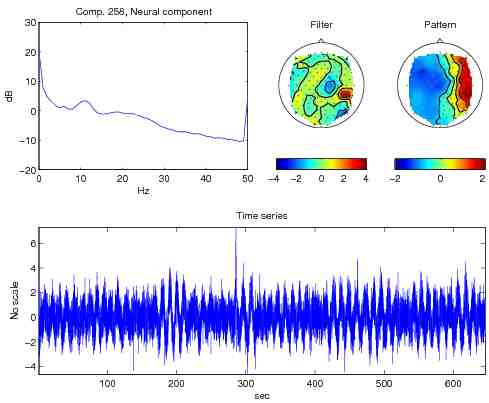

Supplement: Additional file 2 — TrainComponents. Visualization of the 690 independent components in the training RT data, together with the expert's labels. [file 1744-9081-7-30-S2.GZ › components_train/comp258.jpg]

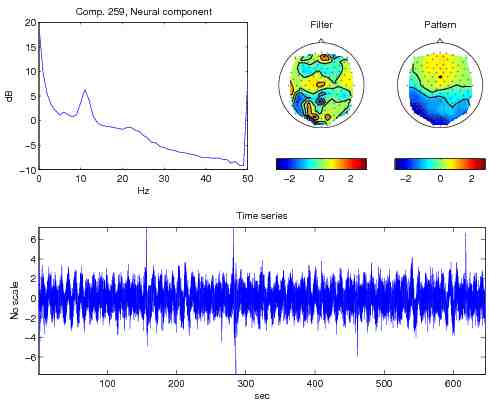

Supplement: Additional file 2 — TrainComponents. Visualization of the 690 independent components in the training RT data, together with the expert's labels. [file 1744-9081-7-30-S2.GZ › components_train/comp259.jpg]

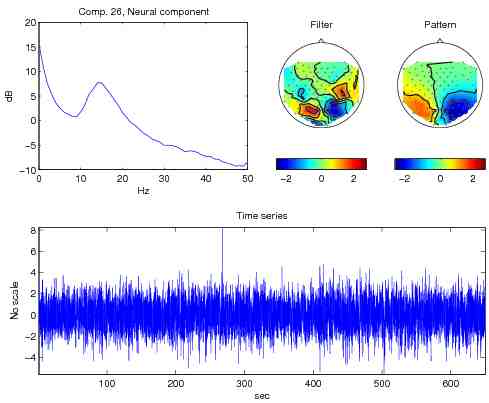

Supplement: Additional file 2 — TrainComponents. Visualization of the 690 independent components in the training RT data, together with the expert's labels. [file 1744-9081-7-30-S2.GZ › components_train/comp26.jpg]

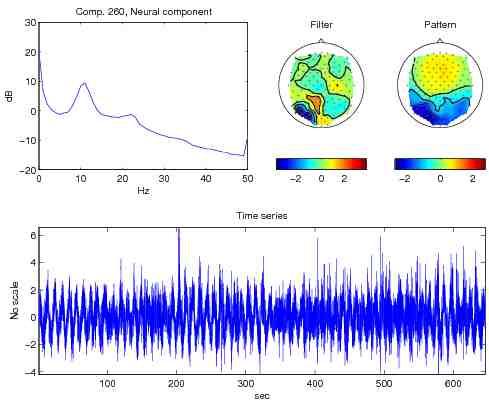

Supplement: Additional file 2 — TrainComponents. Visualization of the 690 independent components in the training RT data, together with the expert's labels. [file 1744-9081-7-30-S2.GZ › components_train/comp260.jpg]

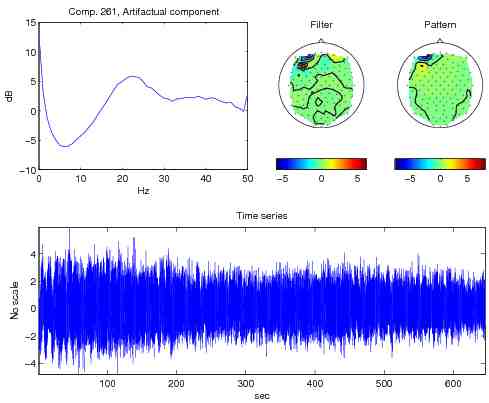

Supplement: Additional file 2 — TrainComponents. Visualization of the 690 independent components in the training RT data, together with the expert's labels. [file 1744-9081-7-30-S2.GZ › components_train/comp261.jpg]

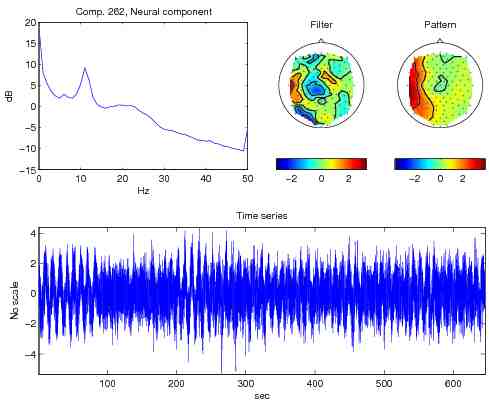

Supplement: Additional file 2 — TrainComponents. Visualization of the 690 independent components in the training RT data, together with the expert's labels. [file 1744-9081-7-30-S2.GZ › components_train/comp262.jpg]

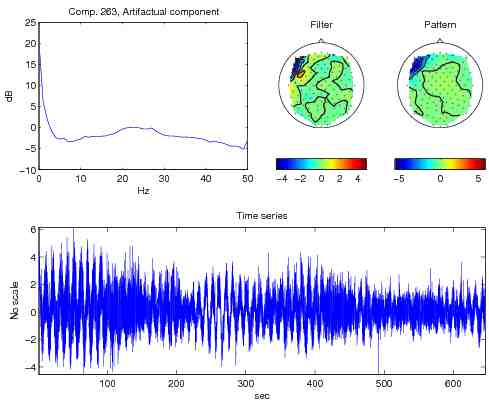

Supplement: Additional file 2 — TrainComponents. Visualization of the 690 independent components in the training RT data, together with the expert's labels. [file 1744-9081-7-30-S2.GZ › components_train/comp263.jpg]

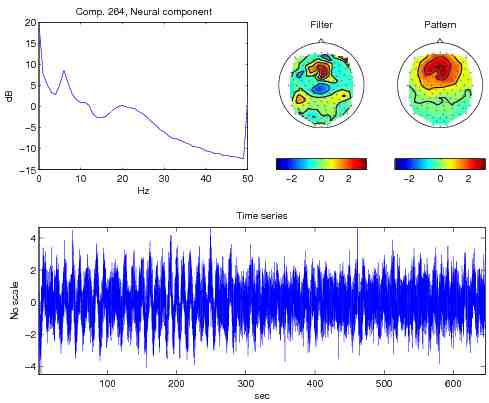

Supplement: Additional file 2 — TrainComponents. Visualization of the 690 independent components in the training RT data, together with the expert's labels. [file 1744-9081-7-30-S2.GZ › components_train/comp264.jpg]

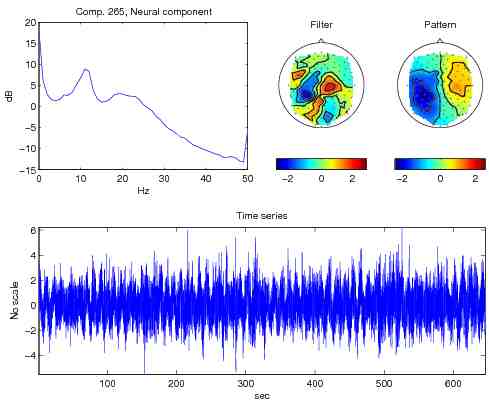

Supplement: Additional file 2 — TrainComponents. Visualization of the 690 independent components in the training RT data, together with the expert's labels. [file 1744-9081-7-30-S2.GZ › components_train/comp265.jpg]

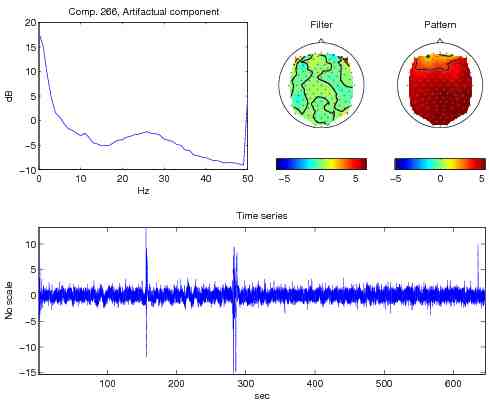

Supplement: Additional file 2 — TrainComponents. Visualization of the 690 independent components in the training RT data, together with the expert's labels. [file 1744-9081-7-30-S2.GZ › components_train/comp266.jpg]

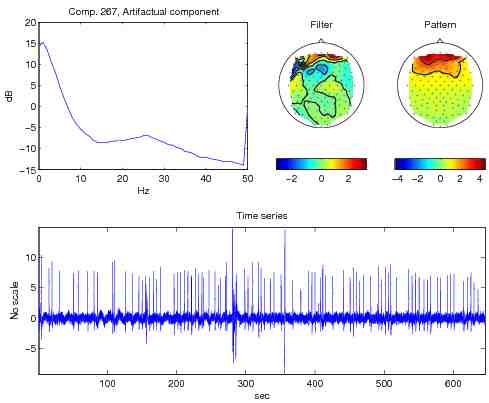

Supplement: Additional file 2 — TrainComponents. Visualization of the 690 independent components in the training RT data, together with the expert's labels. [file 1744-9081-7-30-S2.GZ › components_train/comp267.jpg]

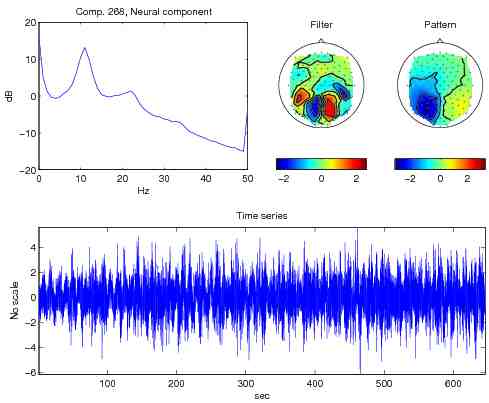

Supplement: Additional file 2 — TrainComponents. Visualization of the 690 independent components in the training RT data, together with the expert's labels. [file 1744-9081-7-30-S2.GZ › components_train/comp268.jpg]

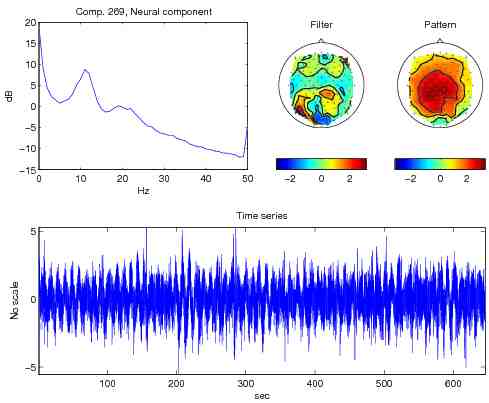

Supplement: Additional file 2 — TrainComponents. Visualization of the 690 independent components in the training RT data, together with the expert's labels. [file 1744-9081-7-30-S2.GZ › components_train/comp269.jpg]

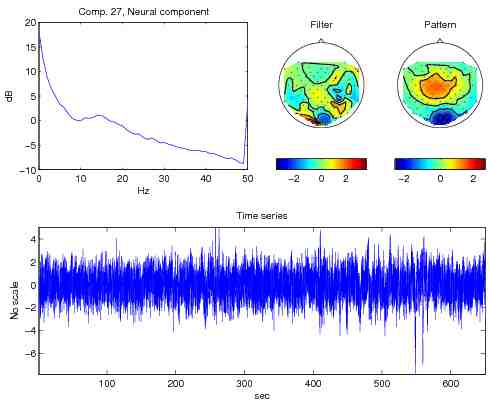

Supplement: Additional file 2 — TrainComponents. Visualization of the 690 independent components in the training RT data, together with the expert's labels. [file 1744-9081-7-30-S2.GZ › components_train/comp27.jpg]

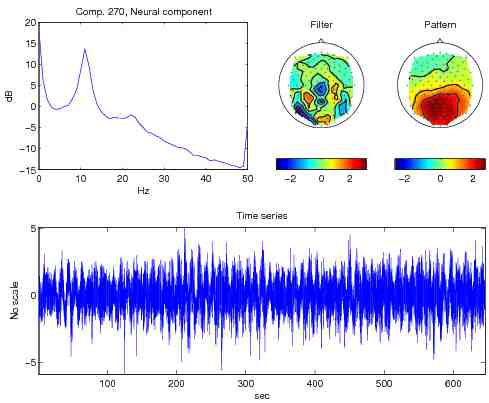

Supplement: Additional file 2 — TrainComponents. Visualization of the 690 independent components in the training RT data, together with the expert's labels. [file 1744-9081-7-30-S2.GZ › components_train/comp270.jpg]

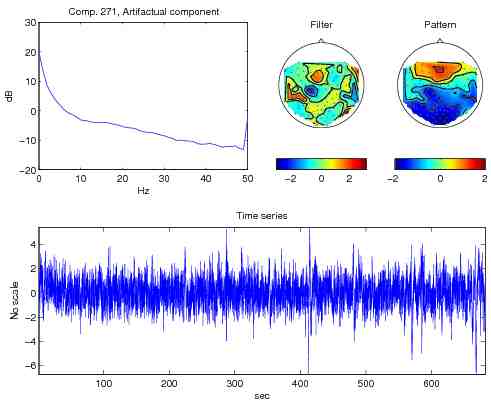

Supplement: Additional file 2 — TrainComponents. Visualization of the 690 independent components in the training RT data, together with the expert's labels. [file 1744-9081-7-30-S2.GZ › components_train/comp271.jpg]

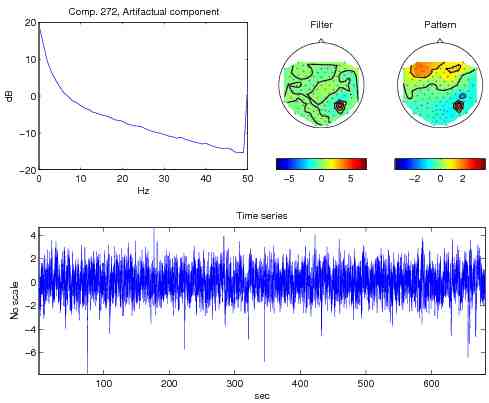

Supplement: Additional file 2 — TrainComponents. Visualization of the 690 independent components in the training RT data, together with the expert's labels. [file 1744-9081-7-30-S2.GZ › components_train/comp272.jpg]

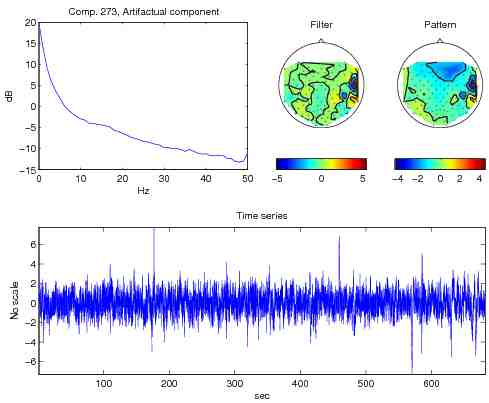

Supplement: Additional file 2 — TrainComponents. Visualization of the 690 independent components in the training RT data, together with the expert's labels. [file 1744-9081-7-30-S2.GZ › components_train/comp273.jpg]

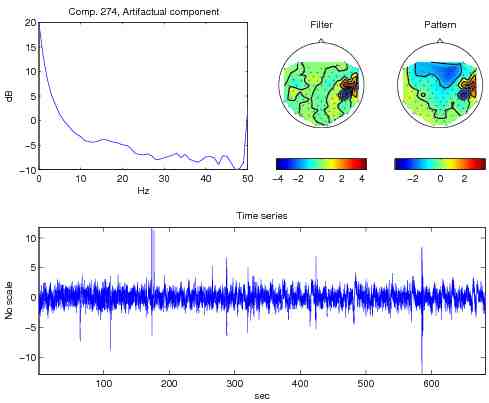

Supplement: Additional file 2 — TrainComponents. Visualization of the 690 independent components in the training RT data, together with the expert's labels. [file 1744-9081-7-30-S2.GZ › components_train/comp274.jpg]

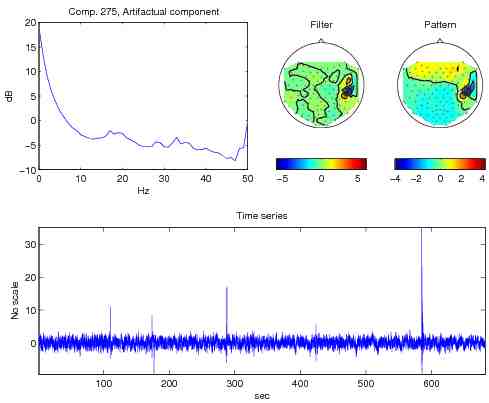

Supplement: Additional file 2 — TrainComponents. Visualization of the 690 independent components in the training RT data, together with the expert's labels. [file 1744-9081-7-30-S2.GZ › components_train/comp275.jpg]

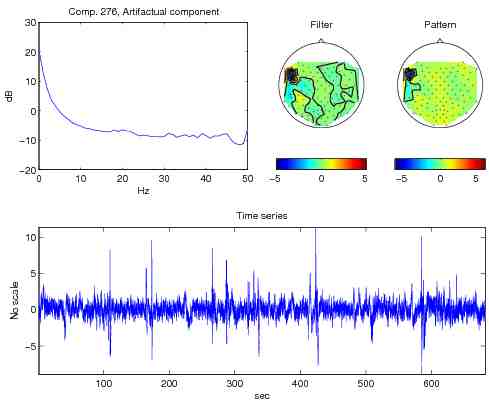

Supplement: Additional file 2 — TrainComponents. Visualization of the 690 independent components in the training RT data, together with the expert's labels. [file 1744-9081-7-30-S2.GZ › components_train/comp276.jpg]

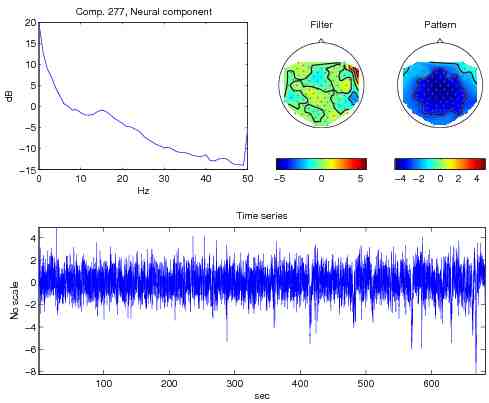

Supplement: Additional file 2 — TrainComponents. Visualization of the 690 independent components in the training RT data, together with the expert's labels. [file 1744-9081-7-30-S2.GZ › components_train/comp277.jpg]

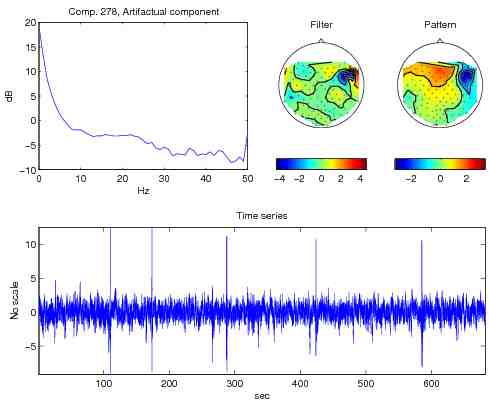

Supplement: Additional file 2 — TrainComponents. Visualization of the 690 independent components in the training RT data, together with the expert's labels. [file 1744-9081-7-30-S2.GZ › components_train/comp278.jpg]

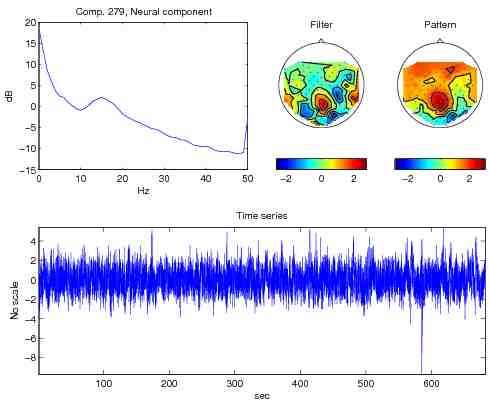

Supplement: Additional file 2 — TrainComponents. Visualization of the 690 independent components in the training RT data, together with the expert's labels. [file 1744-9081-7-30-S2.GZ › components_train/comp279.jpg]
