# Supplementary figures and images for: Automatic Classification of Artifactual ICA-Components for Artifact Removal in EEG Signals (part 1 of 5)
Source: Behav Brain Funct. 2011 Aug 2;7:30. doi: 10.1186/1744-9081-7-30 (PMC3175453; doi:10.1186/1744-9081-7-30)

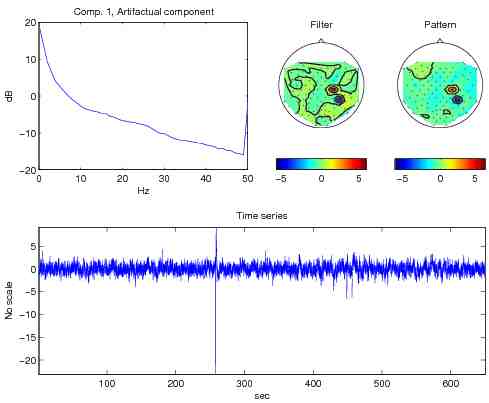

Supplement: Additional file 2 — TrainComponents. Visualization of the 690 independent components in the training RT data, together with the expert's labels. [file 1744-9081-7-30-S2.GZ › components_train/comp1.jpg]

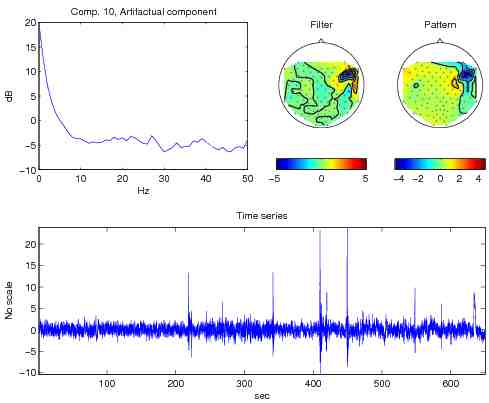

Supplement: Additional file 2 — TrainComponents. Visualization of the 690 independent components in the training RT data, together with the expert's labels. [file 1744-9081-7-30-S2.GZ › components_train/comp10.jpg]

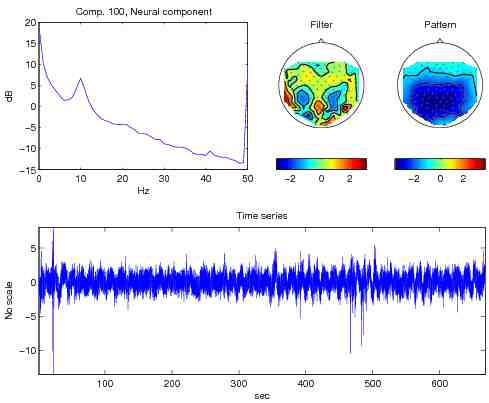

Supplement: Additional file 2 — TrainComponents. Visualization of the 690 independent components in the training RT data, together with the expert's labels. [file 1744-9081-7-30-S2.GZ › components_train/comp100.jpg]

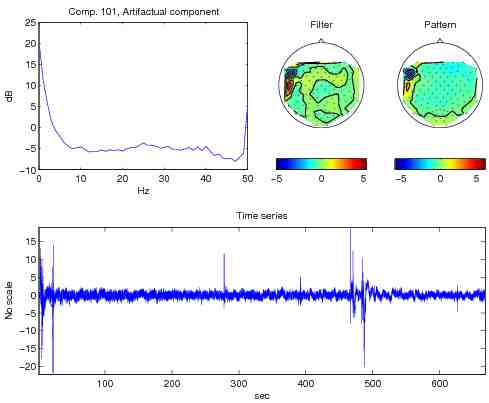

Supplement: Additional file 2 — TrainComponents. Visualization of the 690 independent components in the training RT data, together with the expert's labels. [file 1744-9081-7-30-S2.GZ › components_train/comp101.jpg]

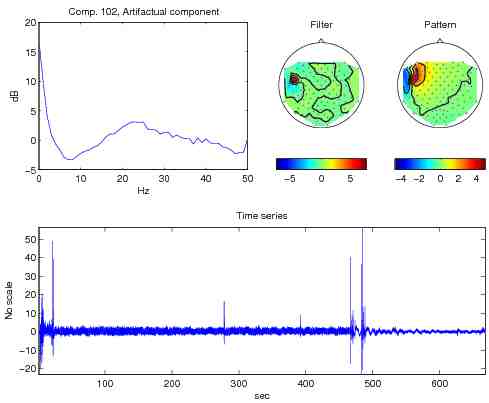

Supplement: Additional file 2 — TrainComponents. Visualization of the 690 independent components in the training RT data, together with the expert's labels. [file 1744-9081-7-30-S2.GZ › components_train/comp102.jpg]

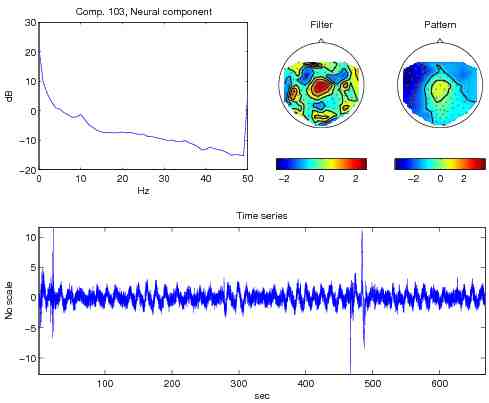

Supplement: Additional file 2 — TrainComponents. Visualization of the 690 independent components in the training RT data, together with the expert's labels. [file 1744-9081-7-30-S2.GZ › components_train/comp103.jpg]

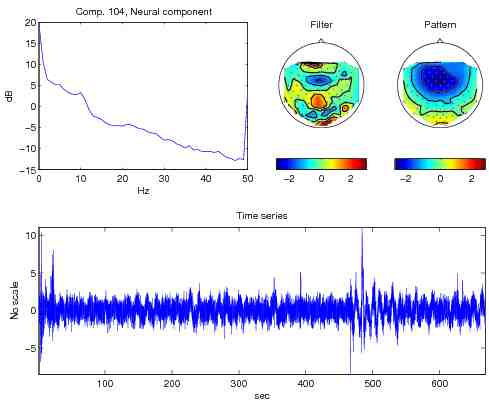

Supplement: Additional file 2 — TrainComponents. Visualization of the 690 independent components in the training RT data, together with the expert's labels. [file 1744-9081-7-30-S2.GZ › components_train/comp104.jpg]

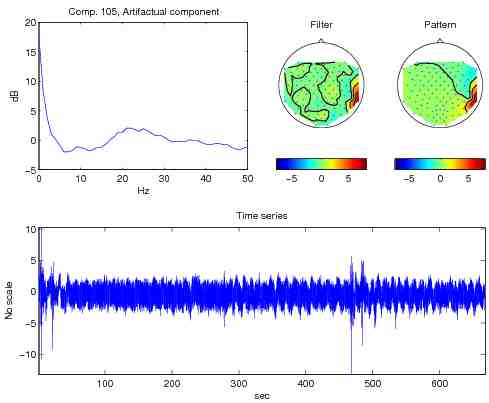

Supplement: Additional file 2 — TrainComponents. Visualization of the 690 independent components in the training RT data, together with the expert's labels. [file 1744-9081-7-30-S2.GZ › components_train/comp105.jpg]

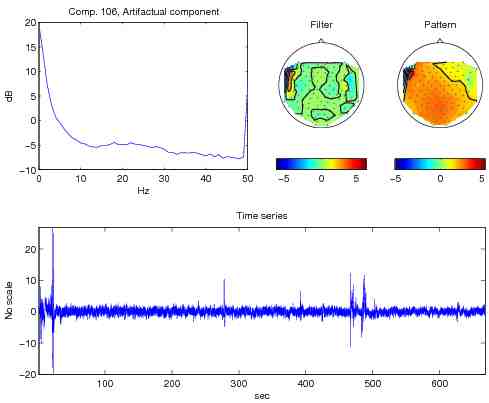

Supplement: Additional file 2 — TrainComponents. Visualization of the 690 independent components in the training RT data, together with the expert's labels. [file 1744-9081-7-30-S2.GZ › components_train/comp106.jpg]

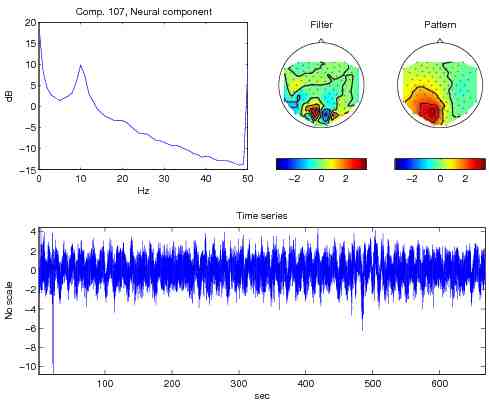

Supplement: Additional file 2 — TrainComponents. Visualization of the 690 independent components in the training RT data, together with the expert's labels. [file 1744-9081-7-30-S2.GZ › components_train/comp107.jpg]

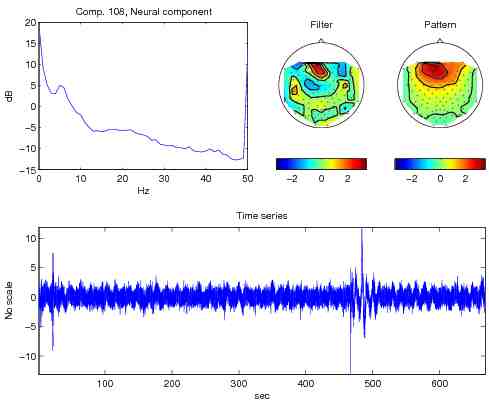

Supplement: Additional file 2 — TrainComponents. Visualization of the 690 independent components in the training RT data, together with the expert's labels. [file 1744-9081-7-30-S2.GZ › components_train/comp108.jpg]

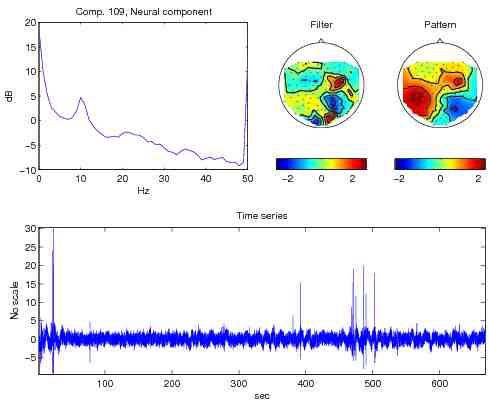

Supplement: Additional file 2 — TrainComponents. Visualization of the 690 independent components in the training RT data, together with the expert's labels. [file 1744-9081-7-30-S2.GZ › components_train/comp109.jpg]

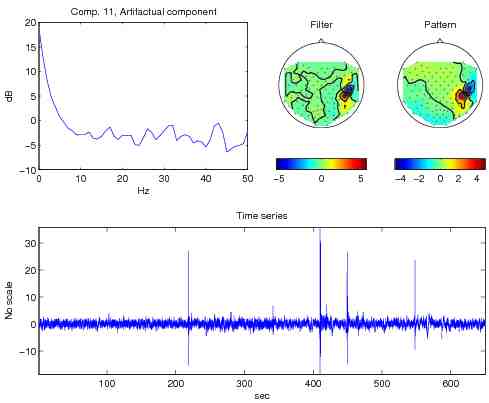

Supplement: Additional file 2 — TrainComponents. Visualization of the 690 independent components in the training RT data, together with the expert's labels. [file 1744-9081-7-30-S2.GZ › components_train/comp11.jpg]

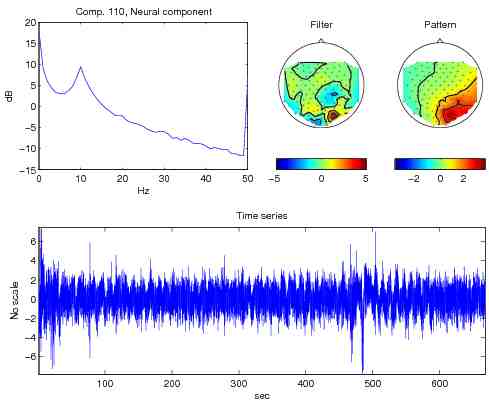

Supplement: Additional file 2 — TrainComponents. Visualization of the 690 independent components in the training RT data, together with the expert's labels. [file 1744-9081-7-30-S2.GZ › components_train/comp110.jpg]

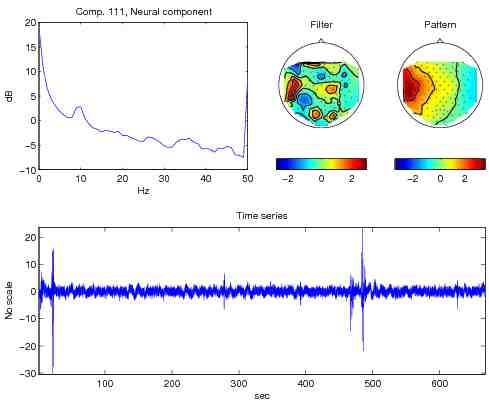

Supplement: Additional file 2 — TrainComponents. Visualization of the 690 independent components in the training RT data, together with the expert's labels. [file 1744-9081-7-30-S2.GZ › components_train/comp111.jpg]

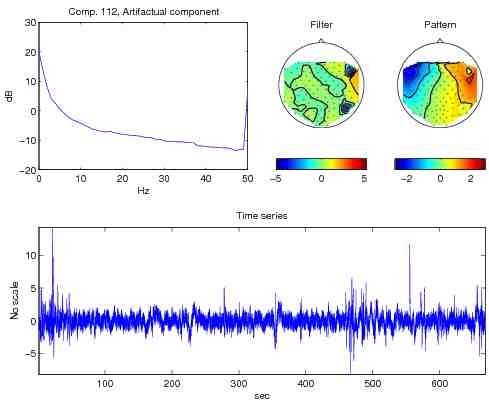

Supplement: Additional file 2 — TrainComponents. Visualization of the 690 independent components in the training RT data, together with the expert's labels. [file 1744-9081-7-30-S2.GZ › components_train/comp112.jpg]

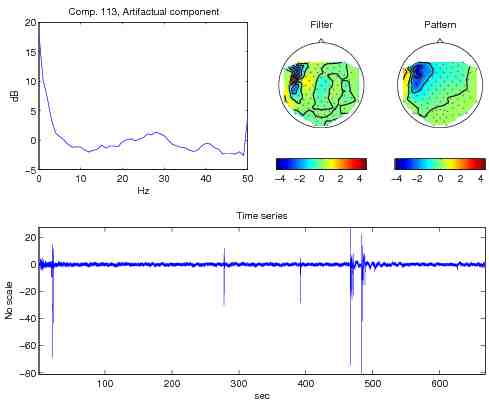

Supplement: Additional file 2 — TrainComponents. Visualization of the 690 independent components in the training RT data, together with the expert's labels. [file 1744-9081-7-30-S2.GZ › components_train/comp113.jpg]

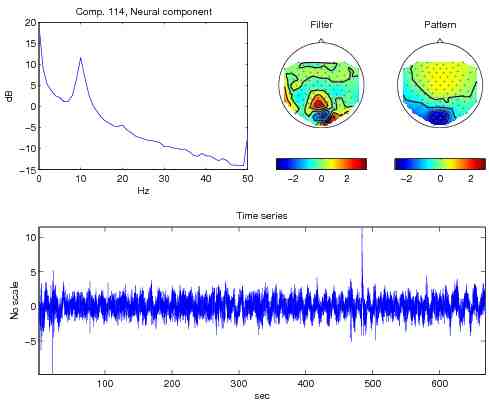

Supplement: Additional file 2 — TrainComponents. Visualization of the 690 independent components in the training RT data, together with the expert's labels. [file 1744-9081-7-30-S2.GZ › components_train/comp114.jpg]

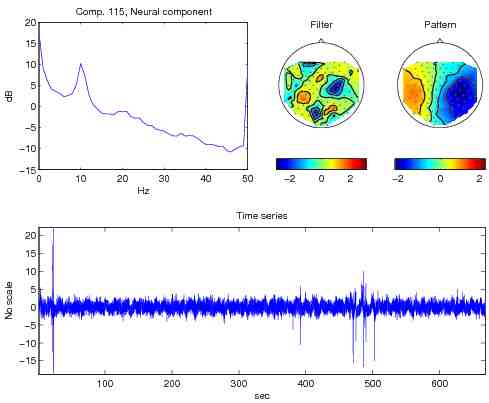

Supplement: Additional file 2 — TrainComponents. Visualization of the 690 independent components in the training RT data, together with the expert's labels. [file 1744-9081-7-30-S2.GZ › components_train/comp115.jpg]

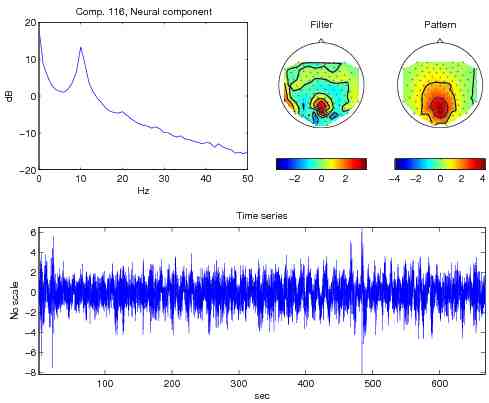

Supplement: Additional file 2 — TrainComponents. Visualization of the 690 independent components in the training RT data, together with the expert's labels. [file 1744-9081-7-30-S2.GZ › components_train/comp116.jpg]

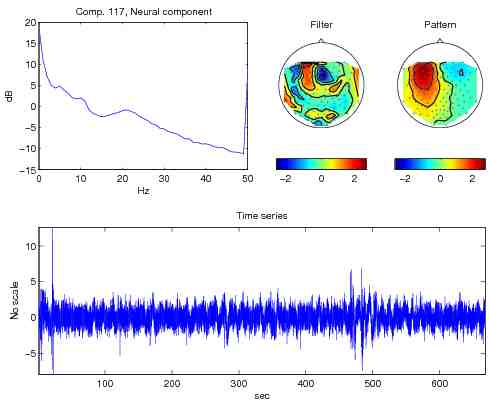

Supplement: Additional file 2 — TrainComponents. Visualization of the 690 independent components in the training RT data, together with the expert's labels. [file 1744-9081-7-30-S2.GZ › components_train/comp117.jpg]

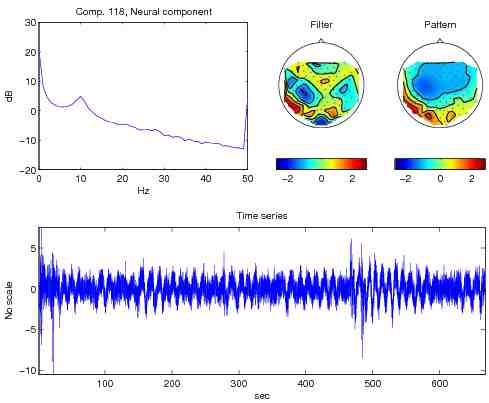

Supplement: Additional file 2 — TrainComponents. Visualization of the 690 independent components in the training RT data, together with the expert's labels. [file 1744-9081-7-30-S2.GZ › components_train/comp118.jpg]

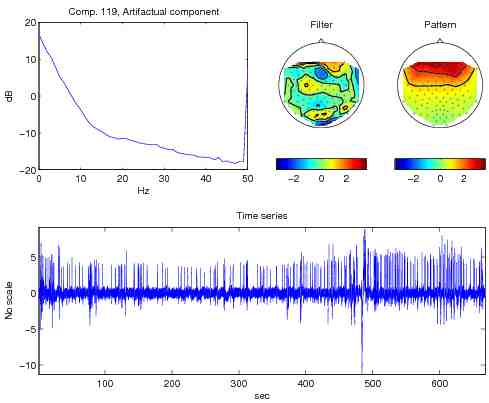

Supplement: Additional file 2 — TrainComponents. Visualization of the 690 independent components in the training RT data, together with the expert's labels. [file 1744-9081-7-30-S2.GZ › components_train/comp119.jpg]

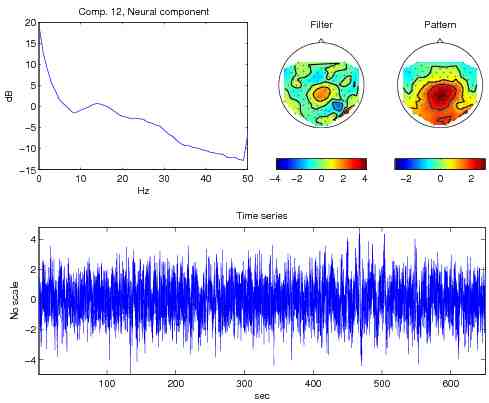

Supplement: Additional file 2 — TrainComponents. Visualization of the 690 independent components in the training RT data, together with the expert's labels. [file 1744-9081-7-30-S2.GZ › components_train/comp12.jpg]

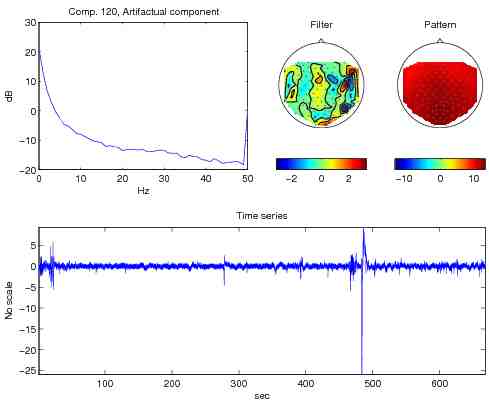

Supplement: Additional file 2 — TrainComponents. Visualization of the 690 independent components in the training RT data, together with the expert's labels. [file 1744-9081-7-30-S2.GZ › components_train/comp120.jpg]

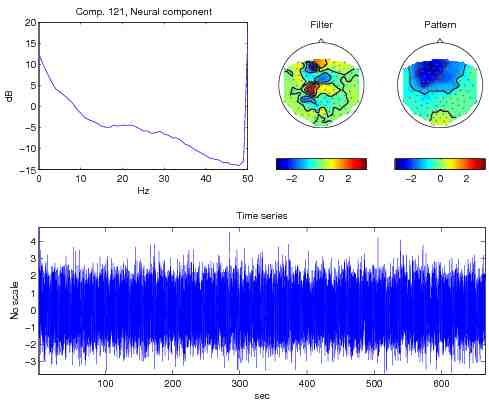

Supplement: Additional file 2 — TrainComponents. Visualization of the 690 independent components in the training RT data, together with the expert's labels. [file 1744-9081-7-30-S2.GZ › components_train/comp121.jpg]

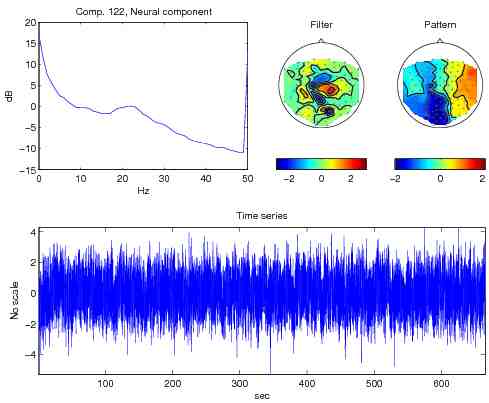

Supplement: Additional file 2 — TrainComponents. Visualization of the 690 independent components in the training RT data, together with the expert's labels. [file 1744-9081-7-30-S2.GZ › components_train/comp122.jpg]

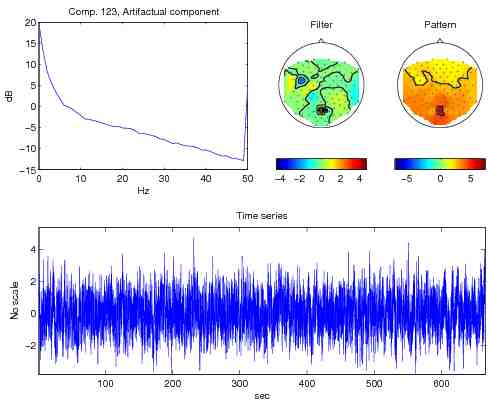

Supplement: Additional file 2 — TrainComponents. Visualization of the 690 independent components in the training RT data, together with the expert's labels. [file 1744-9081-7-30-S2.GZ › components_train/comp123.jpg]

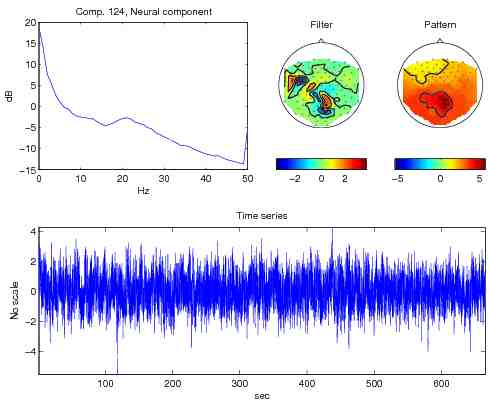

Supplement: Additional file 2 — TrainComponents. Visualization of the 690 independent components in the training RT data, together with the expert's labels. [file 1744-9081-7-30-S2.GZ › components_train/comp124.jpg]

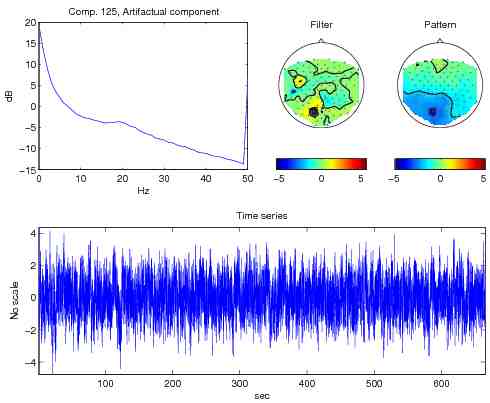

Supplement: Additional file 2 — TrainComponents. Visualization of the 690 independent components in the training RT data, together with the expert's labels. [file 1744-9081-7-30-S2.GZ › components_train/comp125.jpg]

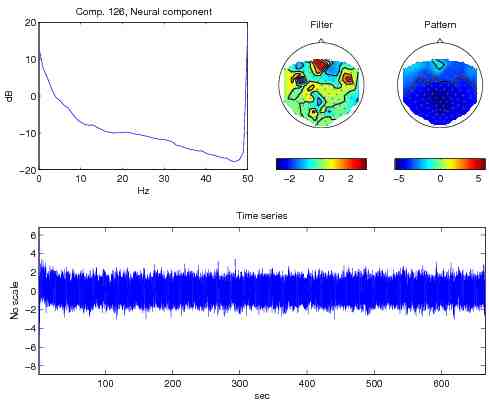

Supplement: Additional file 2 — TrainComponents. Visualization of the 690 independent components in the training RT data, together with the expert's labels. [file 1744-9081-7-30-S2.GZ › components_train/comp126.jpg]

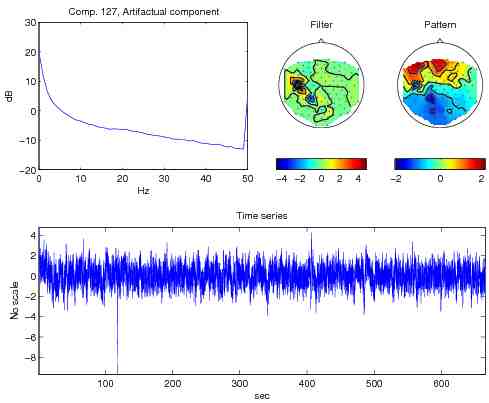

Supplement: Additional file 2 — TrainComponents. Visualization of the 690 independent components in the training RT data, together with the expert's labels. [file 1744-9081-7-30-S2.GZ › components_train/comp127.jpg]

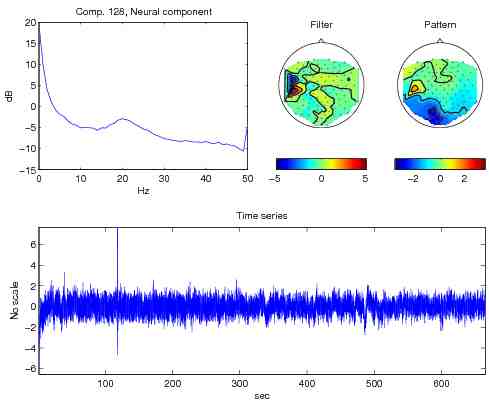

Supplement: Additional file 2 — TrainComponents. Visualization of the 690 independent components in the training RT data, together with the expert's labels. [file 1744-9081-7-30-S2.GZ › components_train/comp128.jpg]

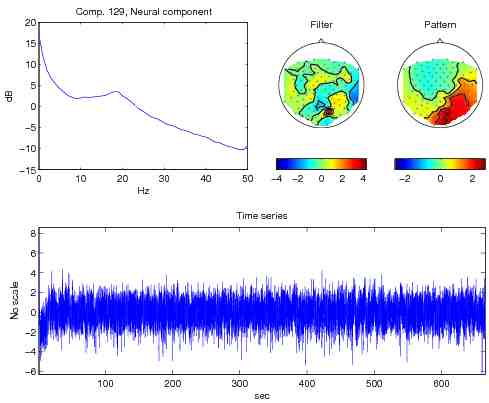

Supplement: Additional file 2 — TrainComponents. Visualization of the 690 independent components in the training RT data, together with the expert's labels. [file 1744-9081-7-30-S2.GZ › components_train/comp129.jpg]

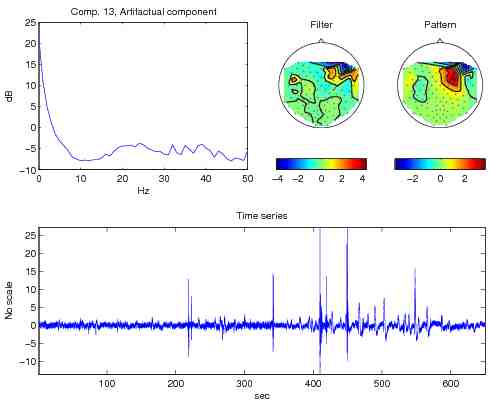

Supplement: Additional file 2 — TrainComponents. Visualization of the 690 independent components in the training RT data, together with the expert's labels. [file 1744-9081-7-30-S2.GZ › components_train/comp13.jpg]

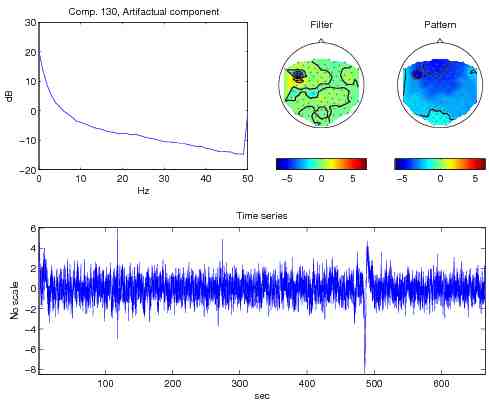

Supplement: Additional file 2 — TrainComponents. Visualization of the 690 independent components in the training RT data, together with the expert's labels. [file 1744-9081-7-30-S2.GZ › components_train/comp130.jpg]

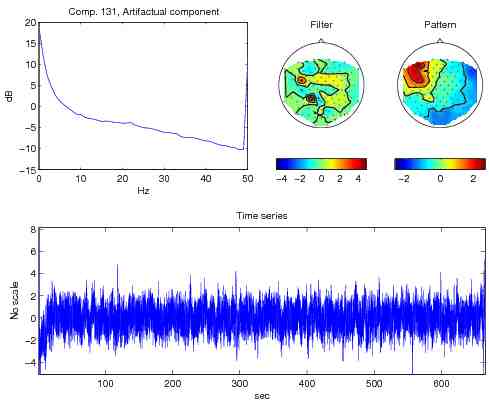

Supplement: Additional file 2 — TrainComponents. Visualization of the 690 independent components in the training RT data, together with the expert's labels. [file 1744-9081-7-30-S2.GZ › components_train/comp131.jpg]

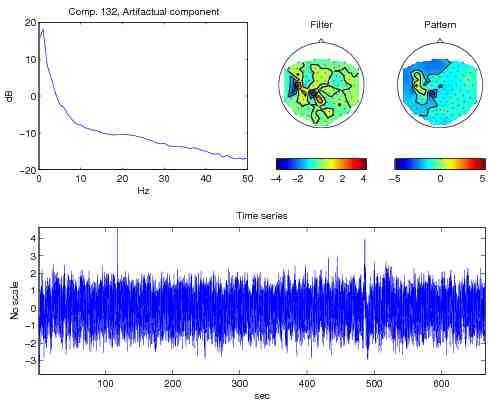

Supplement: Additional file 2 — TrainComponents. Visualization of the 690 independent components in the training RT data, together with the expert's labels. [file 1744-9081-7-30-S2.GZ › components_train/comp132.jpg]

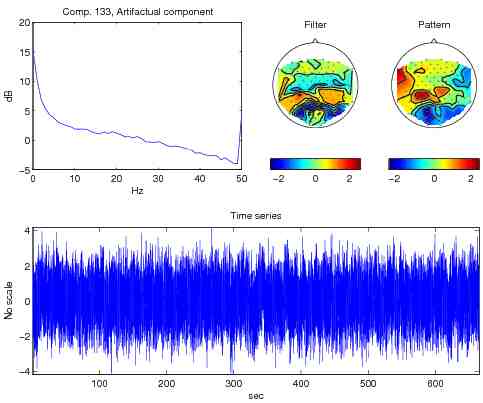

Supplement: Additional file 2 — TrainComponents. Visualization of the 690 independent components in the training RT data, together with the expert's labels. [file 1744-9081-7-30-S2.GZ › components_train/comp133.jpg]

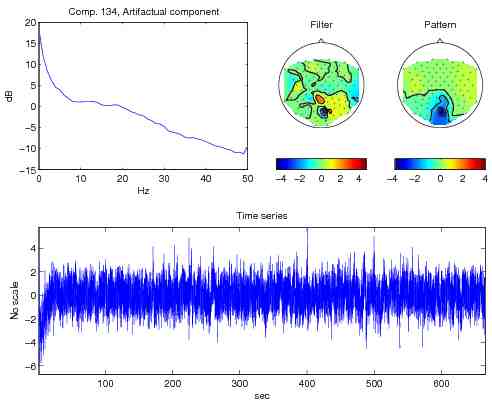

Supplement: Additional file 2 — TrainComponents. Visualization of the 690 independent components in the training RT data, together with the expert's labels. [file 1744-9081-7-30-S2.GZ › components_train/comp134.jpg]

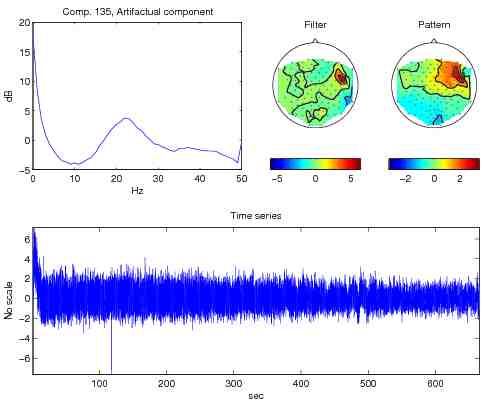

Supplement: Additional file 2 — TrainComponents. Visualization of the 690 independent components in the training RT data, together with the expert's labels. [file 1744-9081-7-30-S2.GZ › components_train/comp135.jpg]

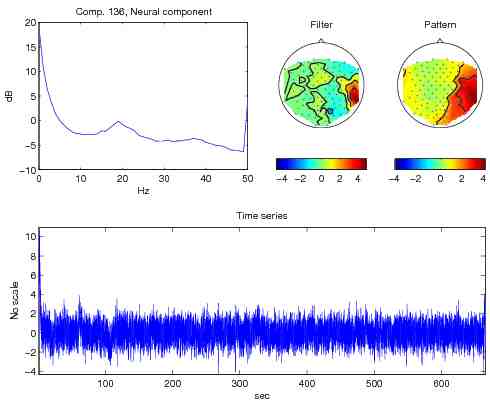

Supplement: Additional file 2 — TrainComponents. Visualization of the 690 independent components in the training RT data, together with the expert's labels. [file 1744-9081-7-30-S2.GZ › components_train/comp136.jpg]

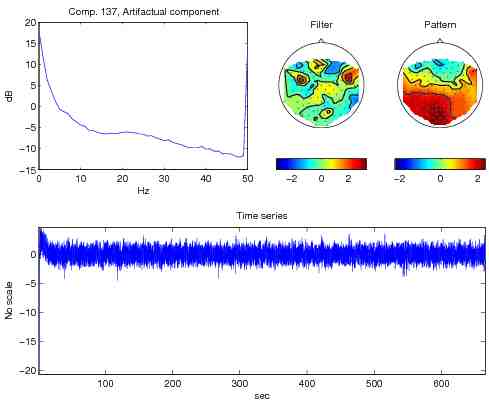

Supplement: Additional file 2 — TrainComponents. Visualization of the 690 independent components in the training RT data, together with the expert's labels. [file 1744-9081-7-30-S2.GZ › components_train/comp137.jpg]

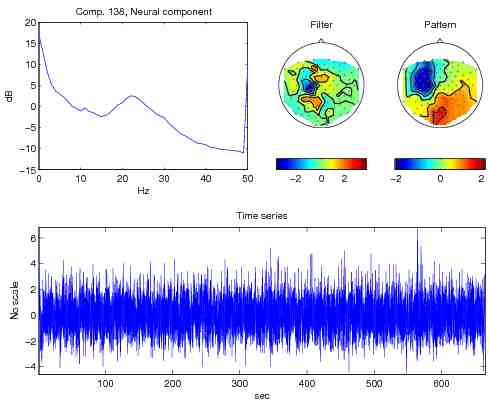

Supplement: Additional file 2 — TrainComponents. Visualization of the 690 independent components in the training RT data, together with the expert's labels. [file 1744-9081-7-30-S2.GZ › components_train/comp138.jpg]

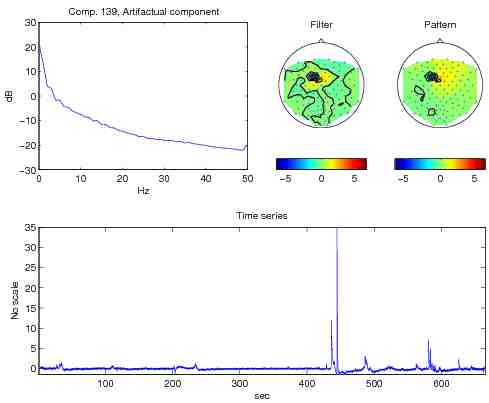

Supplement: Additional file 2 — TrainComponents. Visualization of the 690 independent components in the training RT data, together with the expert's labels. [file 1744-9081-7-30-S2.GZ › components_train/comp139.jpg]

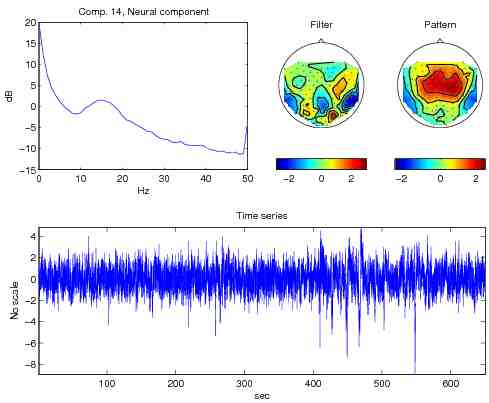

Supplement: Additional file 2 — TrainComponents. Visualization of the 690 independent components in the training RT data, together with the expert's labels. [file 1744-9081-7-30-S2.GZ › components_train/comp14.jpg]

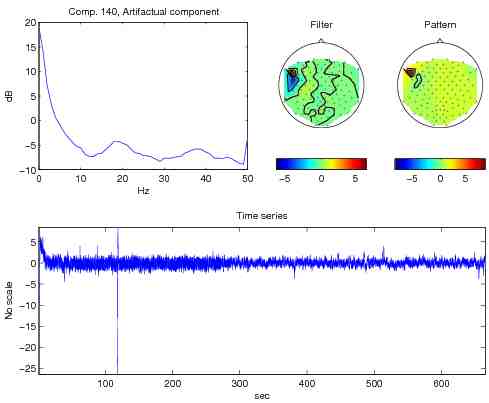

Supplement: Additional file 2 — TrainComponents. Visualization of the 690 independent components in the training RT data, together with the expert's labels. [file 1744-9081-7-30-S2.GZ › components_train/comp140.jpg]

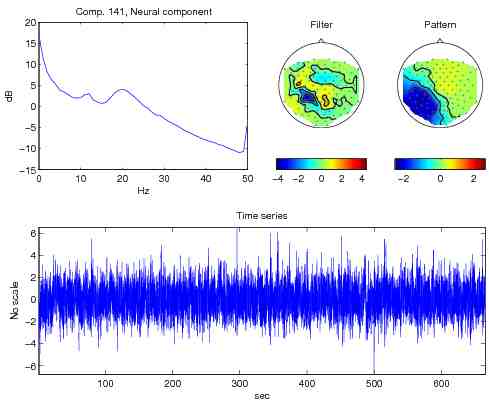

Supplement: Additional file 2 — TrainComponents. Visualization of the 690 independent components in the training RT data, together with the expert's labels. [file 1744-9081-7-30-S2.GZ › components_train/comp141.jpg]

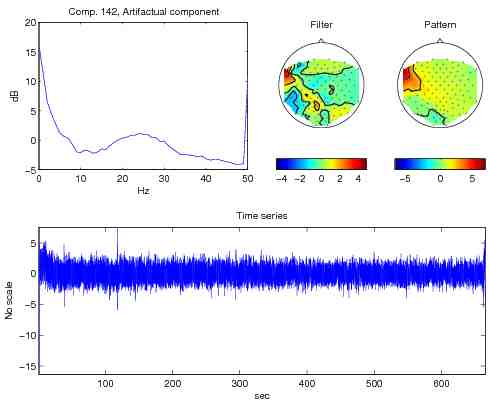

Supplement: Additional file 2 — TrainComponents. Visualization of the 690 independent components in the training RT data, together with the expert's labels. [file 1744-9081-7-30-S2.GZ › components_train/comp142.jpg]

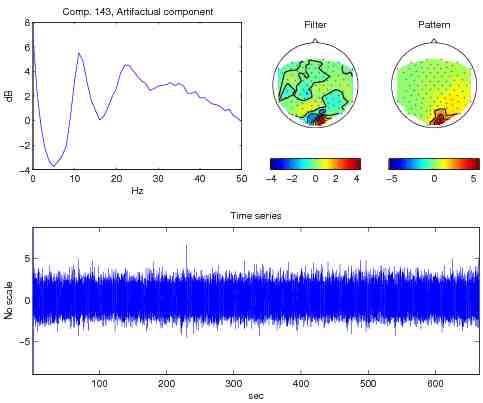

Supplement: Additional file 2 — TrainComponents. Visualization of the 690 independent components in the training RT data, together with the expert's labels. [file 1744-9081-7-30-S2.GZ › components_train/comp143.jpg]

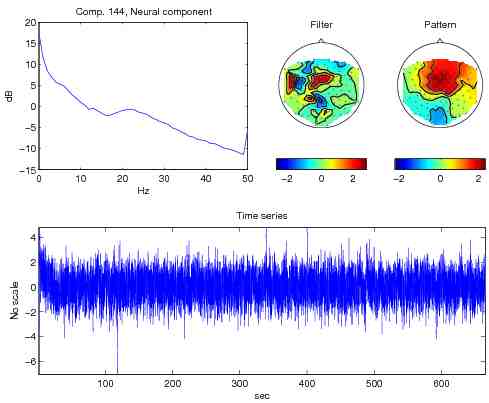

Supplement: Additional file 2 — TrainComponents. Visualization of the 690 independent components in the training RT data, together with the expert's labels. [file 1744-9081-7-30-S2.GZ › components_train/comp144.jpg]

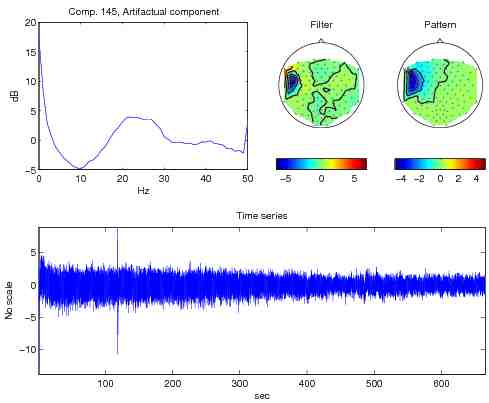

Supplement: Additional file 2 — TrainComponents. Visualization of the 690 independent components in the training RT data, together with the expert's labels. [file 1744-9081-7-30-S2.GZ › components_train/comp145.jpg]

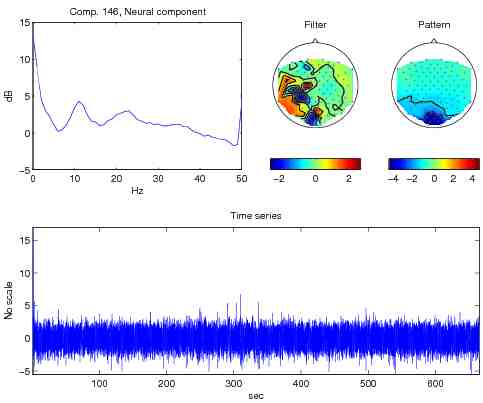

Supplement: Additional file 2 — TrainComponents. Visualization of the 690 independent components in the training RT data, together with the expert's labels. [file 1744-9081-7-30-S2.GZ › components_train/comp146.jpg]

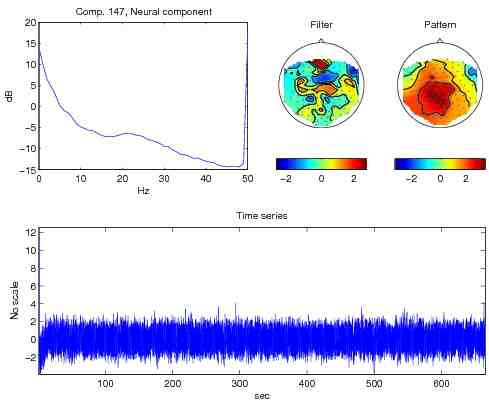

Supplement: Additional file 2 — TrainComponents. Visualization of the 690 independent components in the training RT data, together with the expert's labels. [file 1744-9081-7-30-S2.GZ › components_train/comp147.jpg]

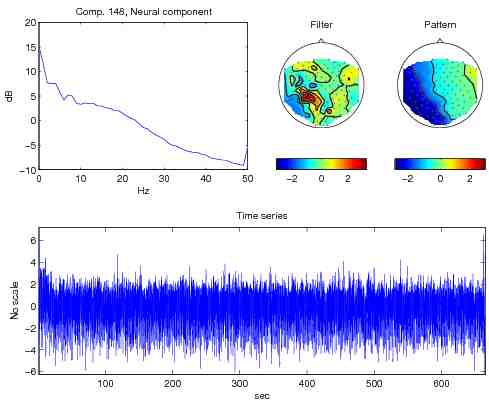

Supplement: Additional file 2 — TrainComponents. Visualization of the 690 independent components in the training RT data, together with the expert's labels. [file 1744-9081-7-30-S2.GZ › components_train/comp148.jpg]

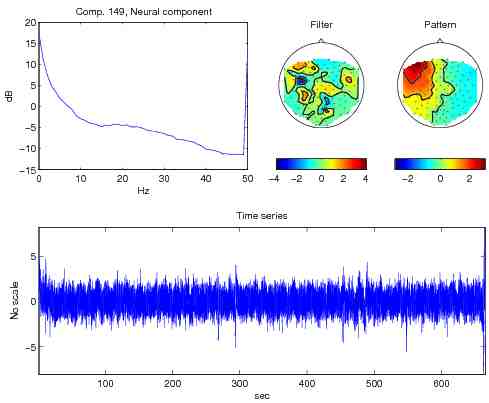

Supplement: Additional file 2 — TrainComponents. Visualization of the 690 independent components in the training RT data, together with the expert's labels. [file 1744-9081-7-30-S2.GZ › components_train/comp149.jpg]

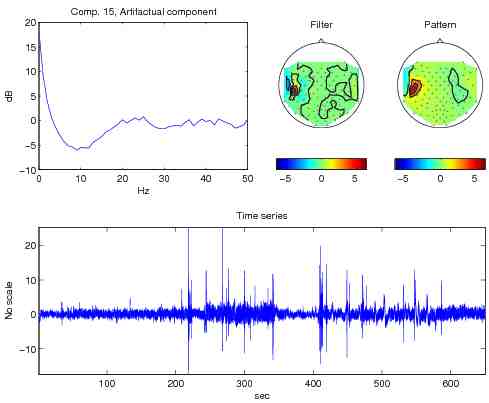

Supplement: Additional file 2 — TrainComponents. Visualization of the 690 independent components in the training RT data, together with the expert's labels. [file 1744-9081-7-30-S2.GZ › components_train/comp15.jpg]

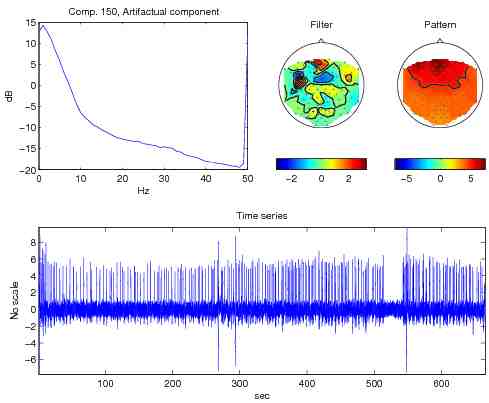

Supplement: Additional file 2 — TrainComponents. Visualization of the 690 independent components in the training RT data, together with the expert's labels. [file 1744-9081-7-30-S2.GZ › components_train/comp150.jpg]

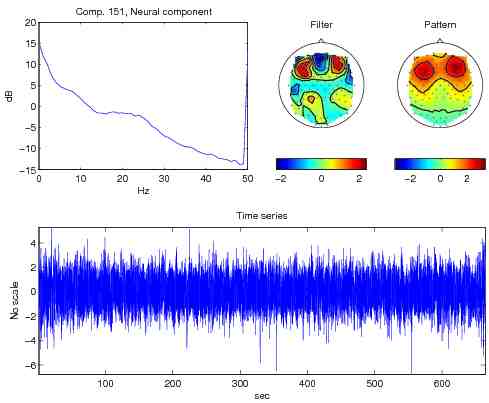

Supplement: Additional file 2 — TrainComponents. Visualization of the 690 independent components in the training RT data, together with the expert's labels. [file 1744-9081-7-30-S2.GZ › components_train/comp151.jpg]

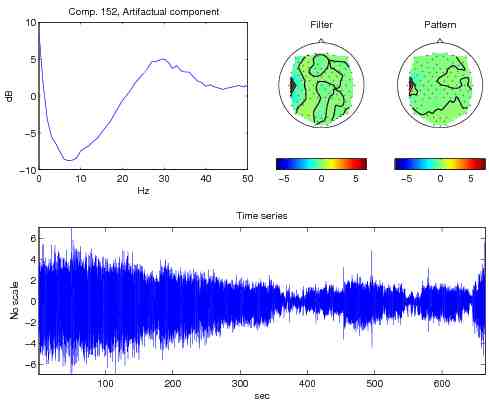

Supplement: Additional file 2 — TrainComponents. Visualization of the 690 independent components in the training RT data, together with the expert's labels. [file 1744-9081-7-30-S2.GZ › components_train/comp152.jpg]

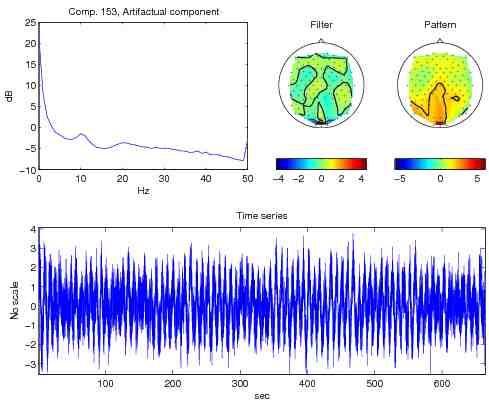

Supplement: Additional file 2 — TrainComponents. Visualization of the 690 independent components in the training RT data, together with the expert's labels. [file 1744-9081-7-30-S2.GZ › components_train/comp153.jpg]

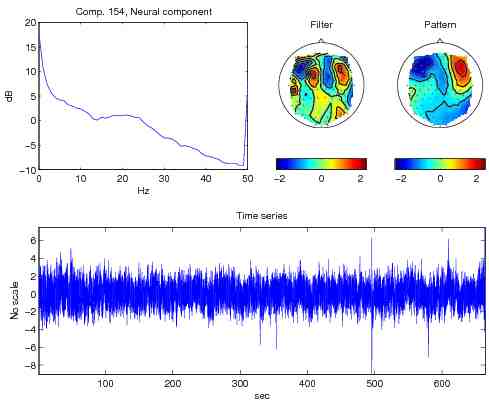

Supplement: Additional file 2 — TrainComponents. Visualization of the 690 independent components in the training RT data, together with the expert's labels. [file 1744-9081-7-30-S2.GZ › components_train/comp154.jpg]

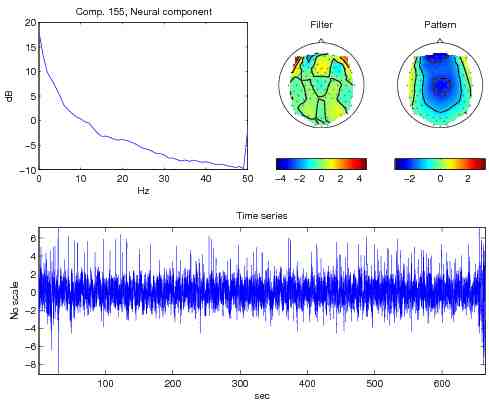

Supplement: Additional file 2 — TrainComponents. Visualization of the 690 independent components in the training RT data, together with the expert's labels. [file 1744-9081-7-30-S2.GZ › components_train/comp155.jpg]

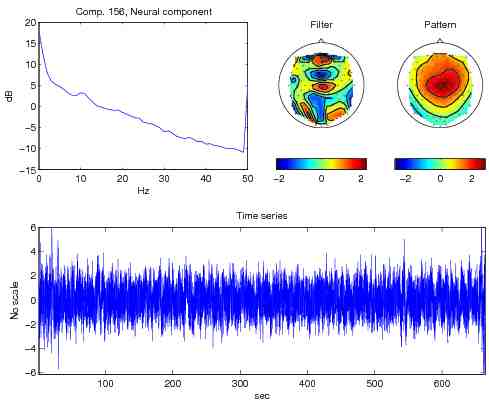

Supplement: Additional file 2 — TrainComponents. Visualization of the 690 independent components in the training RT data, together with the expert's labels. [file 1744-9081-7-30-S2.GZ › components_train/comp156.jpg]

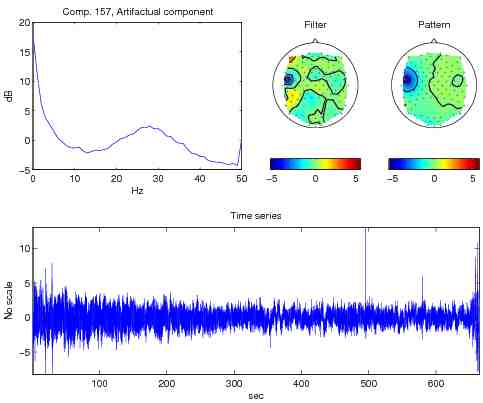

Supplement: Additional file 2 — TrainComponents. Visualization of the 690 independent components in the training RT data, together with the expert's labels. [file 1744-9081-7-30-S2.GZ › components_train/comp157.jpg]

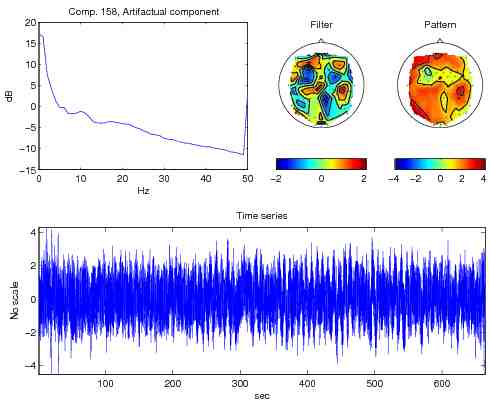

Supplement: Additional file 2 — TrainComponents. Visualization of the 690 independent components in the training RT data, together with the expert's labels. [file 1744-9081-7-30-S2.GZ › components_train/comp158.jpg]

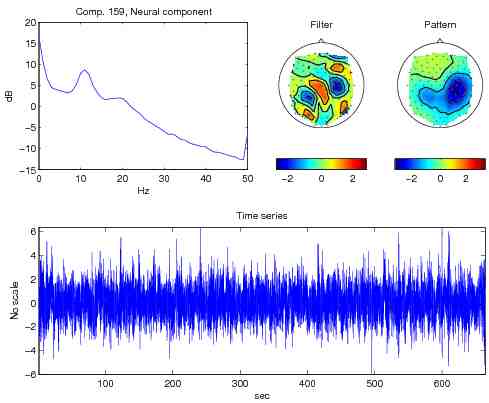

Supplement: Additional file 2 — TrainComponents. Visualization of the 690 independent components in the training RT data, together with the expert's labels. [file 1744-9081-7-30-S2.GZ › components_train/comp159.jpg]

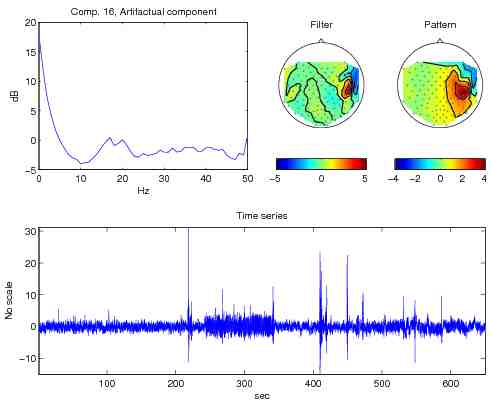

Supplement: Additional file 2 — TrainComponents. Visualization of the 690 independent components in the training RT data, together with the expert's labels. [file 1744-9081-7-30-S2.GZ › components_train/comp16.jpg]

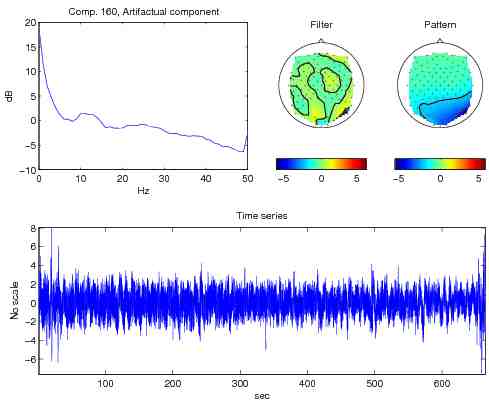

Supplement: Additional file 2 — TrainComponents. Visualization of the 690 independent components in the training RT data, together with the expert's labels. [file 1744-9081-7-30-S2.GZ › components_train/comp160.jpg]

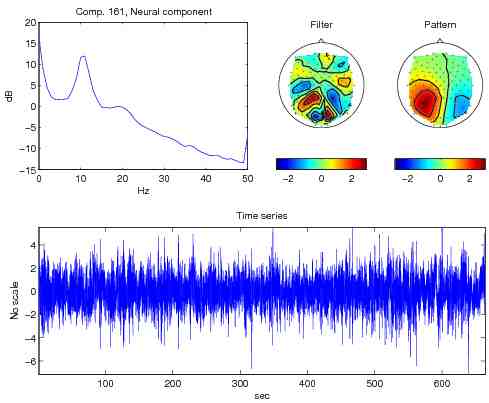

Supplement: Additional file 2 — TrainComponents. Visualization of the 690 independent components in the training RT data, together with the expert's labels. [file 1744-9081-7-30-S2.GZ › components_train/comp161.jpg]

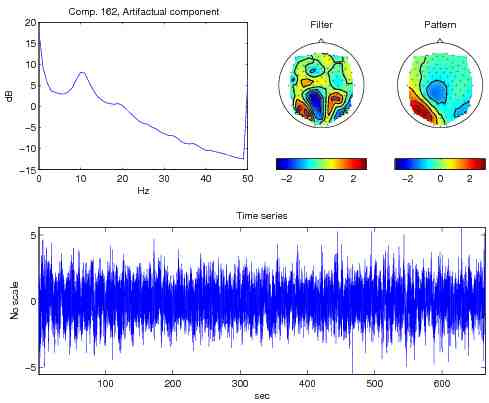

Supplement: Additional file 2 — TrainComponents. Visualization of the 690 independent components in the training RT data, together with the expert's labels. [file 1744-9081-7-30-S2.GZ › components_train/comp162.jpg]

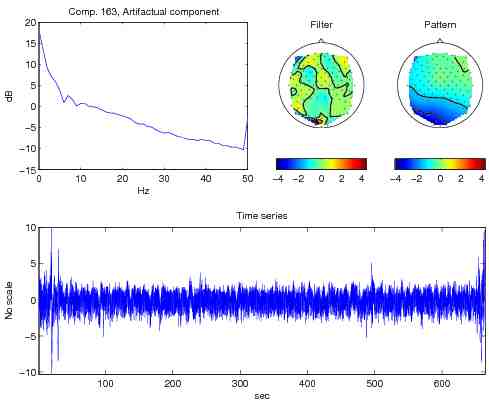

Supplement: Additional file 2 — TrainComponents. Visualization of the 690 independent components in the training RT data, together with the expert's labels. [file 1744-9081-7-30-S2.GZ › components_train/comp163.jpg]

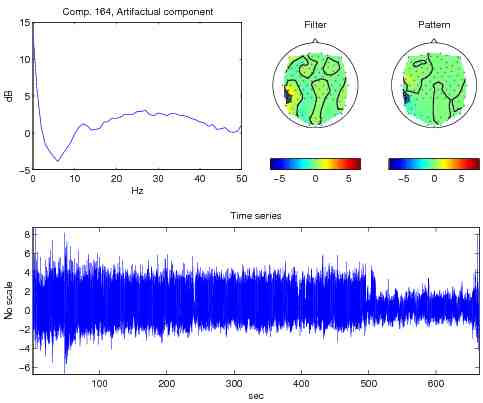

Supplement: Additional file 2 — TrainComponents. Visualization of the 690 independent components in the training RT data, together with the expert's labels. [file 1744-9081-7-30-S2.GZ › components_train/comp164.jpg]

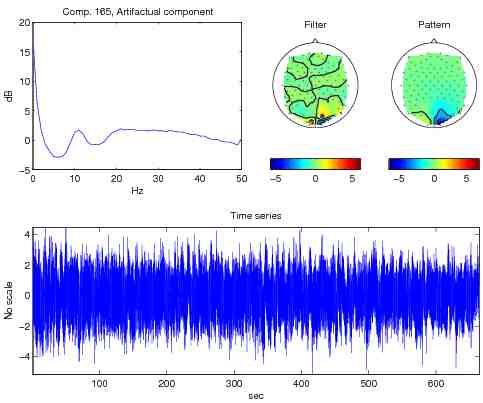

Supplement: Additional file 2 — TrainComponents. Visualization of the 690 independent components in the training RT data, together with the expert's labels. [file 1744-9081-7-30-S2.GZ › components_train/comp165.jpg]

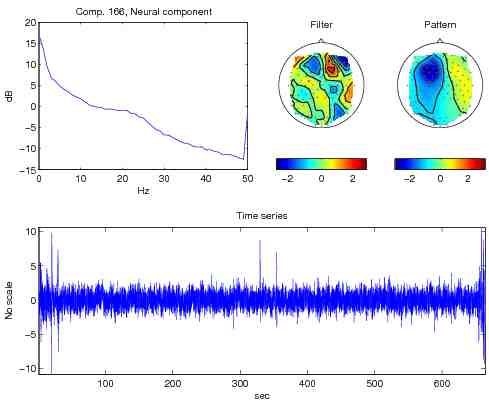

Supplement: Additional file 2 — TrainComponents. Visualization of the 690 independent components in the training RT data, together with the expert's labels. [file 1744-9081-7-30-S2.GZ › components_train/comp166.jpg]

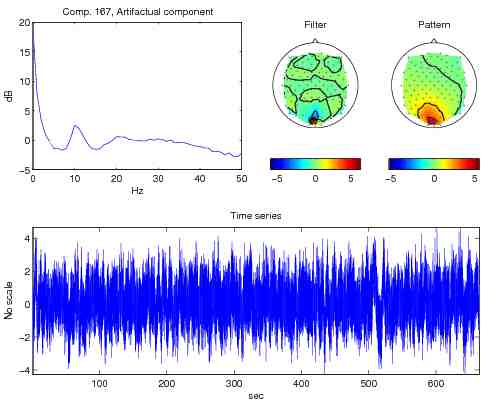

Supplement: Additional file 2 — TrainComponents. Visualization of the 690 independent components in the training RT data, together with the expert's labels. [file 1744-9081-7-30-S2.GZ › components_train/comp167.jpg]

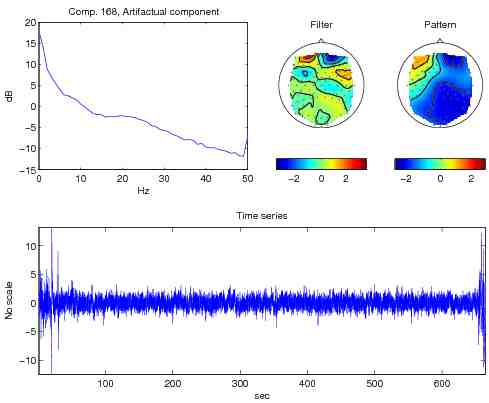

Supplement: Additional file 2 — TrainComponents. Visualization of the 690 independent components in the training RT data, together with the expert's labels. [file 1744-9081-7-30-S2.GZ › components_train/comp168.jpg]

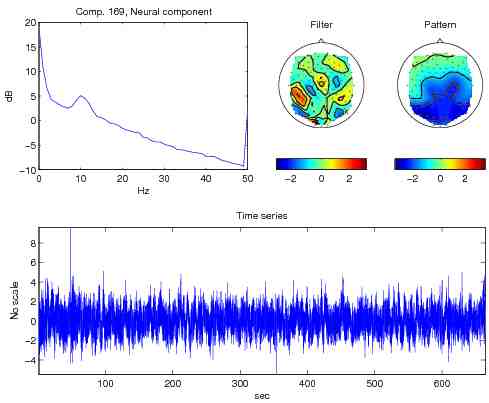

Supplement: Additional file 2 — TrainComponents. Visualization of the 690 independent components in the training RT data, together with the expert's labels. [file 1744-9081-7-30-S2.GZ › components_train/comp169.jpg]

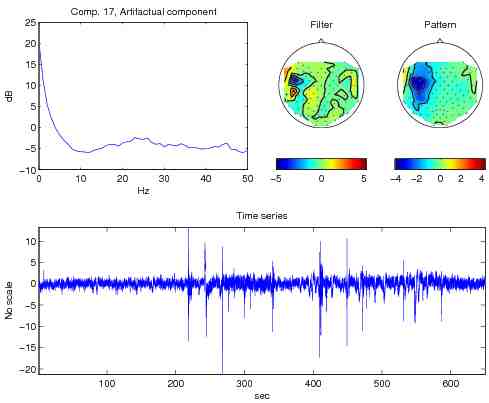

Supplement: Additional file 2 — TrainComponents. Visualization of the 690 independent components in the training RT data, together with the expert's labels. [file 1744-9081-7-30-S2.GZ › components_train/comp17.jpg]

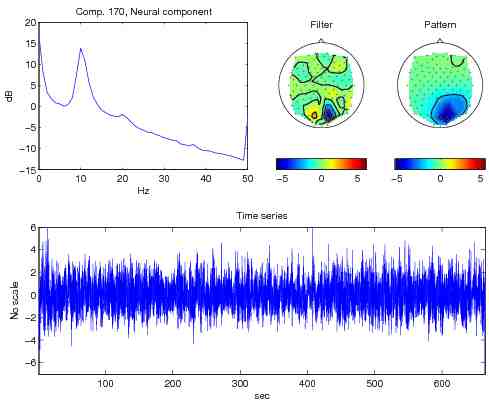

Supplement: Additional file 2 — TrainComponents. Visualization of the 690 independent components in the training RT data, together with the expert's labels. [file 1744-9081-7-30-S2.GZ › components_train/comp170.jpg]

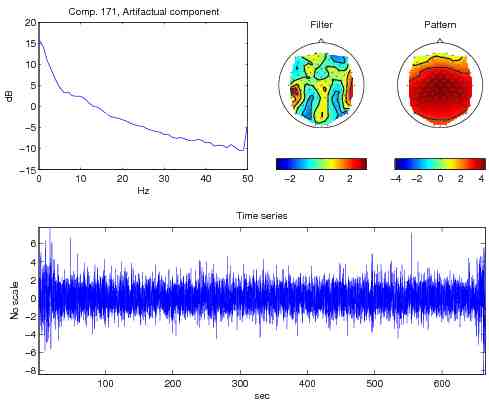

Supplement: Additional file 2 — TrainComponents. Visualization of the 690 independent components in the training RT data, together with the expert's labels. [file 1744-9081-7-30-S2.GZ › components_train/comp171.jpg]

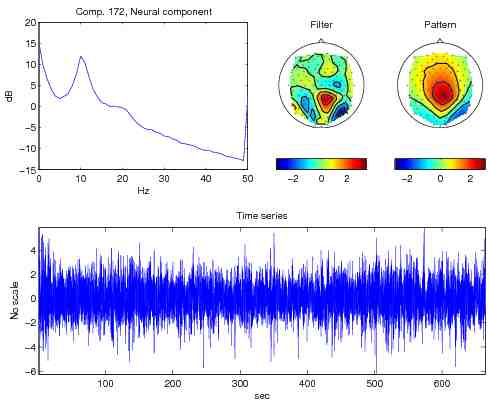

Supplement: Additional file 2 — TrainComponents. Visualization of the 690 independent components in the training RT data, together with the expert's labels. [file 1744-9081-7-30-S2.GZ › components_train/comp172.jpg]

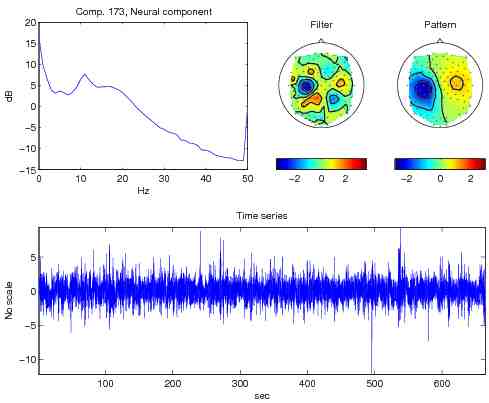

Supplement: Additional file 2 — TrainComponents. Visualization of the 690 independent components in the training RT data, together with the expert's labels. [file 1744-9081-7-30-S2.GZ › components_train/comp173.jpg]

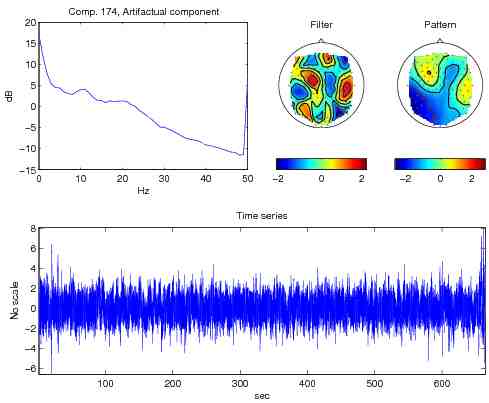

Supplement: Additional file 2 — TrainComponents. Visualization of the 690 independent components in the training RT data, together with the expert's labels. [file 1744-9081-7-30-S2.GZ › components_train/comp174.jpg]

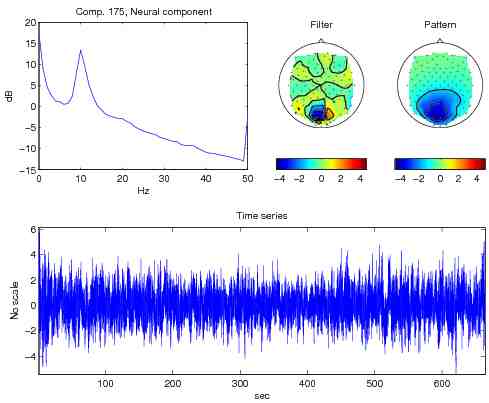

Supplement: Additional file 2 — TrainComponents. Visualization of the 690 independent components in the training RT data, together with the expert's labels. [file 1744-9081-7-30-S2.GZ › components_train/comp175.jpg]

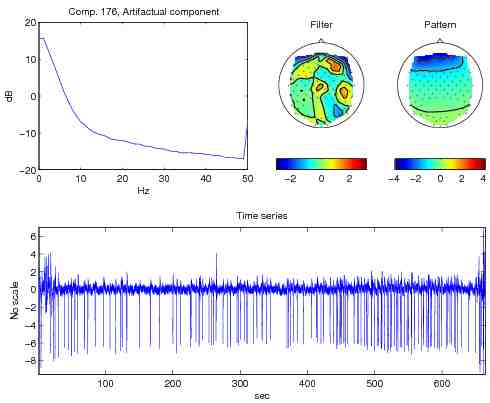

Supplement: Additional file 2 — TrainComponents. Visualization of the 690 independent components in the training RT data, together with the expert's labels. [file 1744-9081-7-30-S2.GZ › components_train/comp176.jpg]

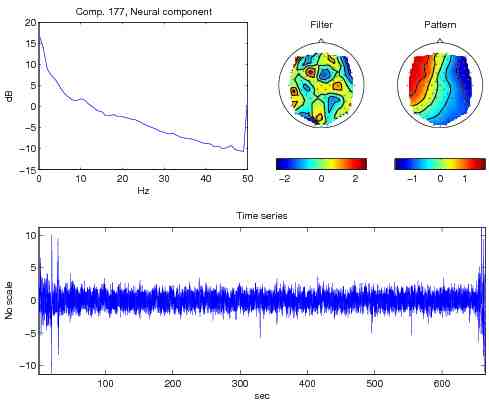

Supplement: Additional file 2 — TrainComponents. Visualization of the 690 independent components in the training RT data, together with the expert's labels. [file 1744-9081-7-30-S2.GZ › components_train/comp177.jpg]

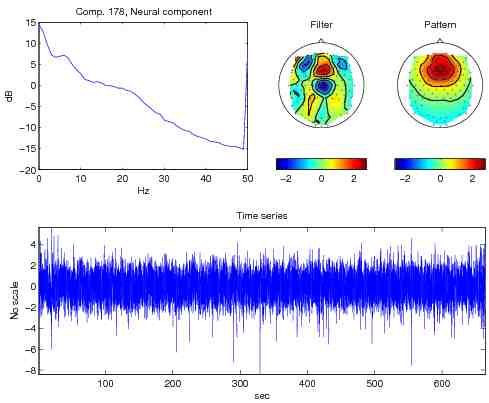

Supplement: Additional file 2 — TrainComponents. Visualization of the 690 independent components in the training RT data, together with the expert's labels. [file 1744-9081-7-30-S2.GZ › components_train/comp178.jpg]

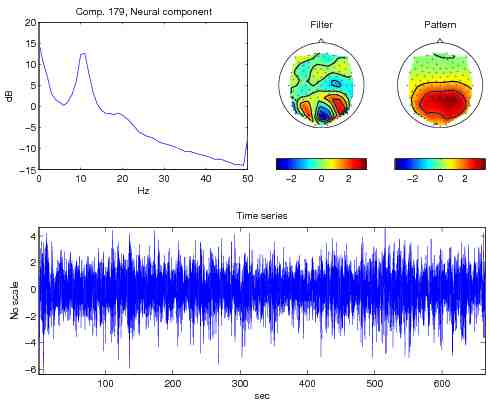

Supplement: Additional file 2 — TrainComponents. Visualization of the 690 independent components in the training RT data, together with the expert's labels. [file 1744-9081-7-30-S2.GZ › components_train/comp179.jpg]

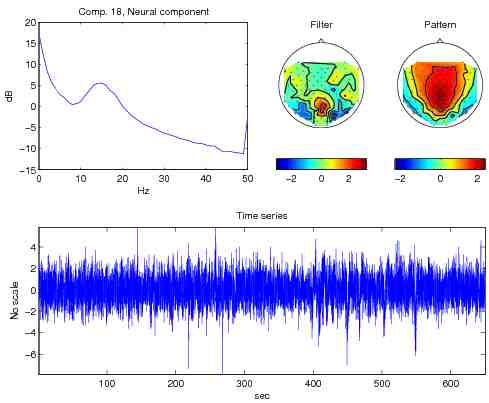

Supplement: Additional file 2 — TrainComponents. Visualization of the 690 independent components in the training RT data, together with the expert's labels. [file 1744-9081-7-30-S2.GZ › components_train/comp18.jpg]

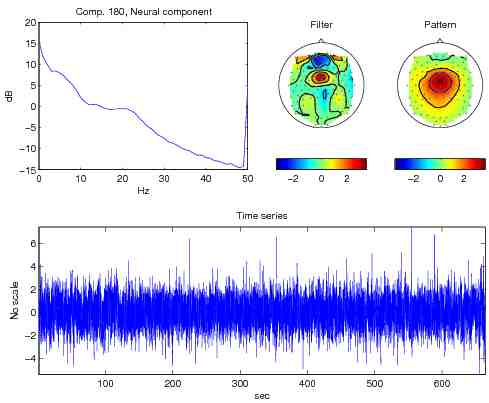

Supplement: Additional file 2 — TrainComponents. Visualization of the 690 independent components in the training RT data, together with the expert's labels. [file 1744-9081-7-30-S2.GZ › components_train/comp180.jpg]

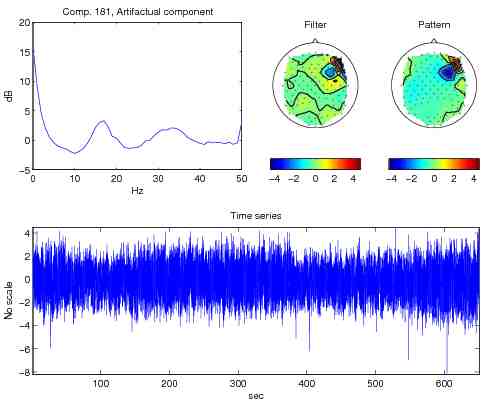

Supplement: Additional file 2 — TrainComponents. Visualization of the 690 independent components in the training RT data, together with the expert's labels. [file 1744-9081-7-30-S2.GZ › components_train/comp181.jpg]

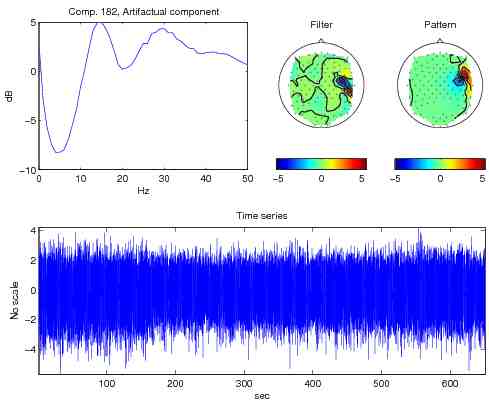

Supplement: Additional file 2 — TrainComponents. Visualization of the 690 independent components in the training RT data, together with the expert's labels. [file 1744-9081-7-30-S2.GZ › components_train/comp182.jpg]

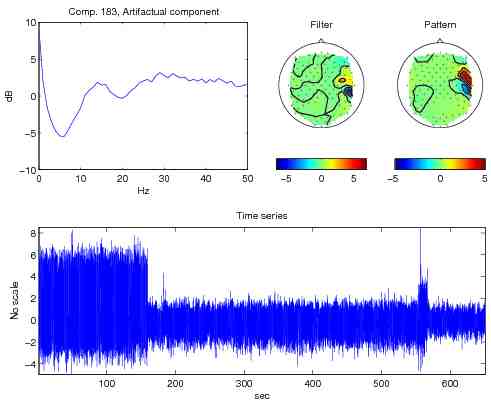

Supplement: Additional file 2 — TrainComponents. Visualization of the 690 independent components in the training RT data, together with the expert's labels. [file 1744-9081-7-30-S2.GZ › components_train/comp183.jpg]

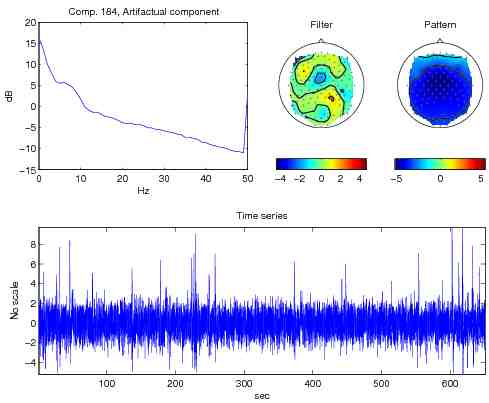

Supplement: Additional file 2 — TrainComponents. Visualization of the 690 independent components in the training RT data, together with the expert's labels. [file 1744-9081-7-30-S2.GZ › components_train/comp184.jpg]

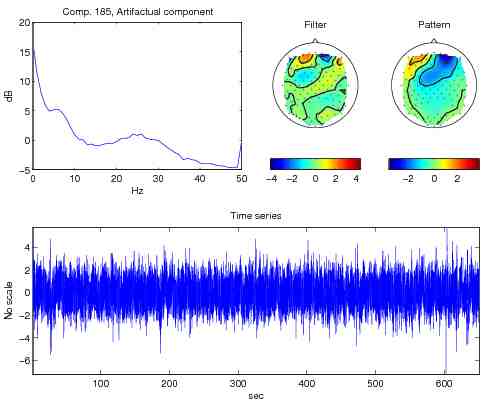

Supplement: Additional file 2 — TrainComponents. Visualization of the 690 independent components in the training RT data, together with the expert's labels. [file 1744-9081-7-30-S2.GZ › components_train/comp185.jpg]

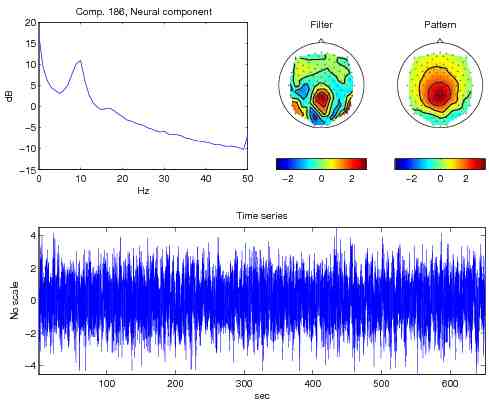

Supplement: Additional file 2 — TrainComponents. Visualization of the 690 independent components in the training RT data, together with the expert's labels. [file 1744-9081-7-30-S2.GZ › components_train/comp186.jpg]

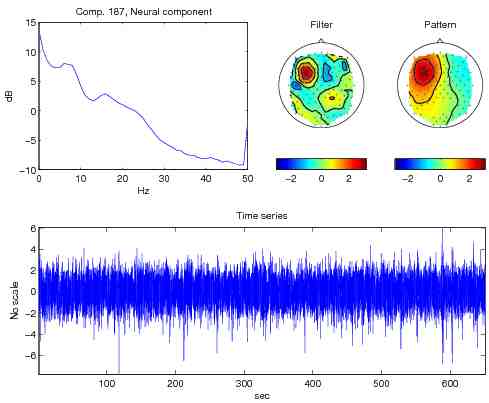

Supplement: Additional file 2 — TrainComponents. Visualization of the 690 independent components in the training RT data, together with the expert's labels. [file 1744-9081-7-30-S2.GZ › components_train/comp187.jpg]

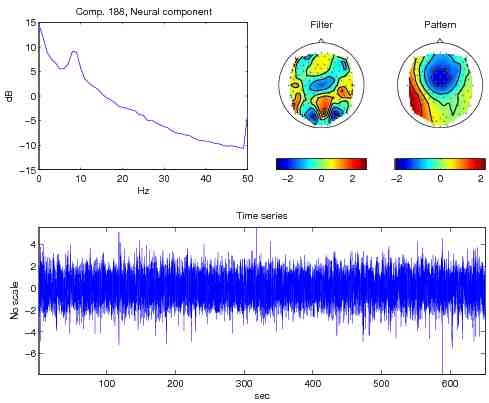

Supplement: Additional file 2 — TrainComponents. Visualization of the 690 independent components in the training RT data, together with the expert's labels. [file 1744-9081-7-30-S2.GZ › components_train/comp188.jpg]

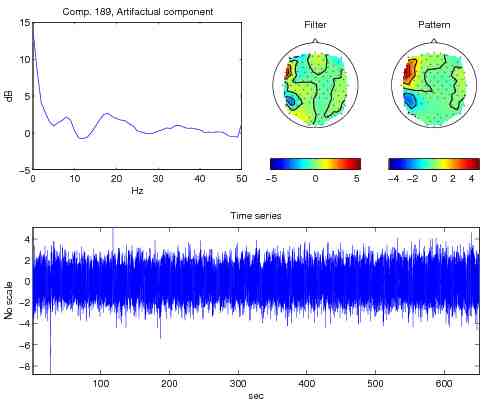

Supplement: Additional file 2 — TrainComponents. Visualization of the 690 independent components in the training RT data, together with the expert's labels. [file 1744-9081-7-30-S2.GZ › components_train/comp189.jpg]
