# Supplementary figures and images for: Automatic Classification of Artifactual ICA-Components for Artifact Removal in EEG Signals (part 4 of 5)
Source: Behav Brain Funct. 2011 Aug 2;7:30. doi: 10.1186/1744-9081-7-30 (PMC3175453; doi:10.1186/1744-9081-7-30)

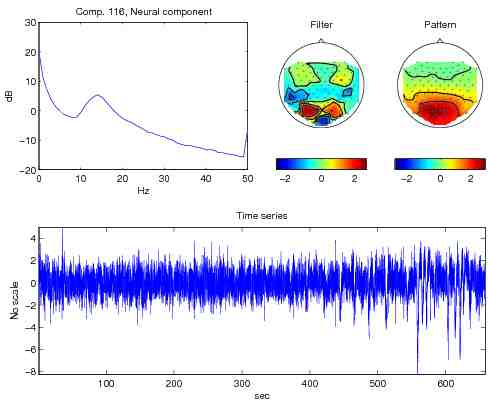

Supplement: Additional file 3 — TestComponents. Visualization of the 1080 independent components in the RT test data, together with the expert's labels. [file 1744-9081-7-30-S3.GZ › components_test/comp116.jpg]

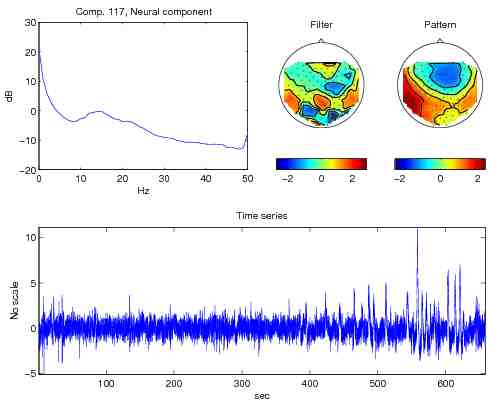

Supplement: Additional file 3 — TestComponents. Visualization of the 1080 independent components in the RT test data, together with the expert's labels. [file 1744-9081-7-30-S3.GZ › components_test/comp117.jpg]

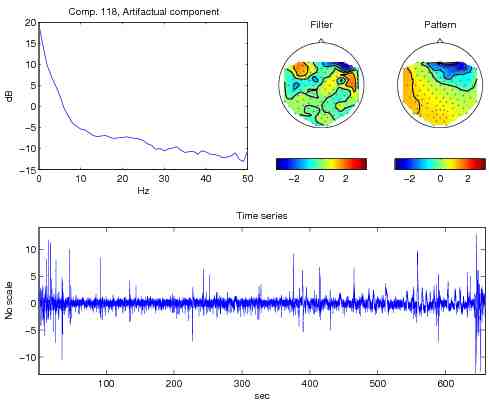

Supplement: Additional file 3 — TestComponents. Visualization of the 1080 independent components in the RT test data, together with the expert's labels. [file 1744-9081-7-30-S3.GZ › components_test/comp118.jpg]

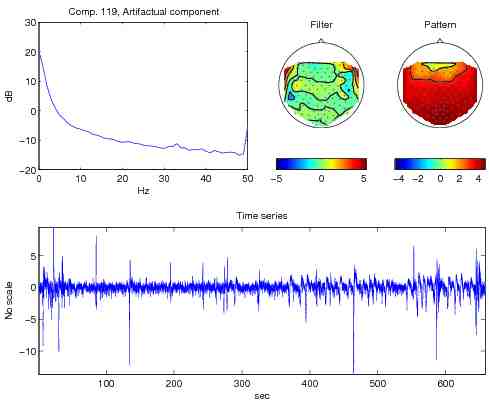

Supplement: Additional file 3 — TestComponents. Visualization of the 1080 independent components in the RT test data, together with the expert's labels. [file 1744-9081-7-30-S3.GZ › components_test/comp119.jpg]

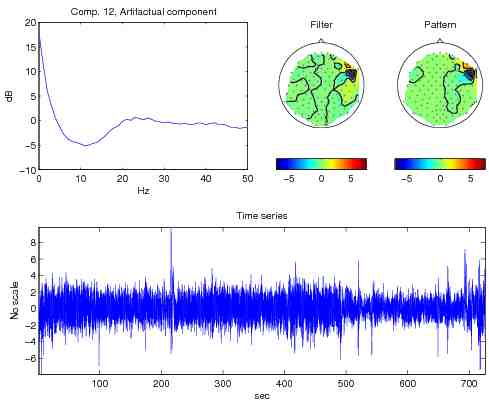

Supplement: Additional file 3 — TestComponents. Visualization of the 1080 independent components in the RT test data, together with the expert's labels. [file 1744-9081-7-30-S3.GZ › components_test/comp12.jpg]

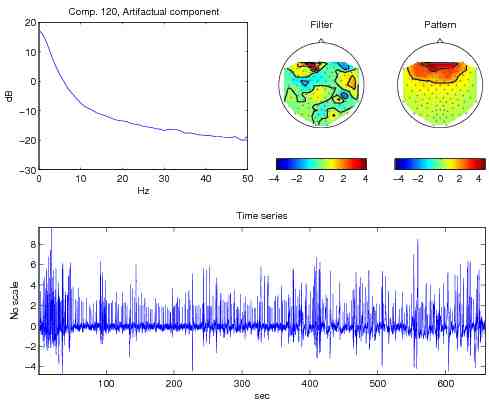

Supplement: Additional file 3 — TestComponents. Visualization of the 1080 independent components in the RT test data, together with the expert's labels. [file 1744-9081-7-30-S3.GZ › components_test/comp120.jpg]

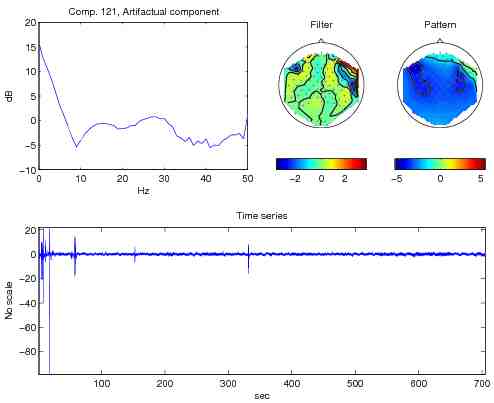

Supplement: Additional file 3 — TestComponents. Visualization of the 1080 independent components in the RT test data, together with the expert's labels. [file 1744-9081-7-30-S3.GZ › components_test/comp121.jpg]

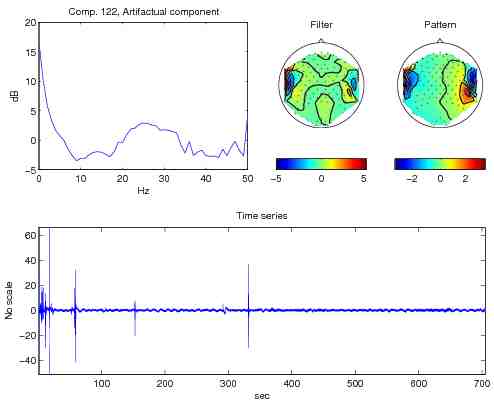

Supplement: Additional file 3 — TestComponents. Visualization of the 1080 independent components in the RT test data, together with the expert's labels. [file 1744-9081-7-30-S3.GZ › components_test/comp122.jpg]

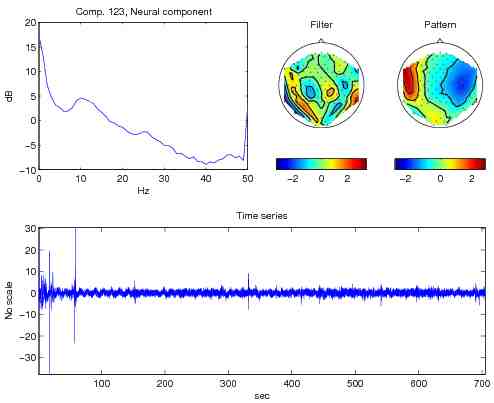

Supplement: Additional file 3 — TestComponents. Visualization of the 1080 independent components in the RT test data, together with the expert's labels. [file 1744-9081-7-30-S3.GZ › components_test/comp123.jpg]

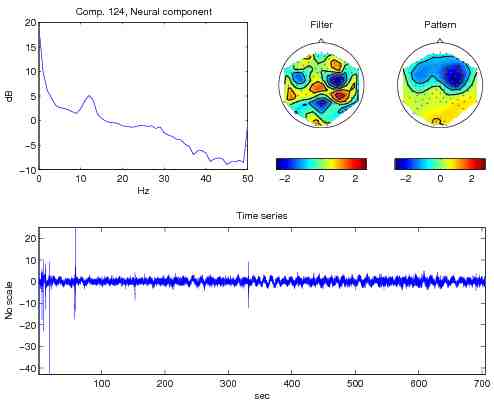

Supplement: Additional file 3 — TestComponents. Visualization of the 1080 independent components in the RT test data, together with the expert's labels. [file 1744-9081-7-30-S3.GZ › components_test/comp124.jpg]

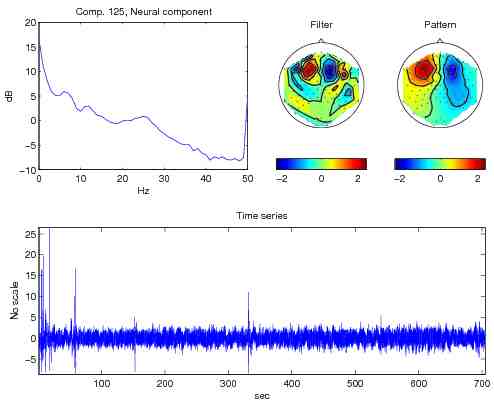

Supplement: Additional file 3 — TestComponents. Visualization of the 1080 independent components in the RT test data, together with the expert's labels. [file 1744-9081-7-30-S3.GZ › components_test/comp125.jpg]

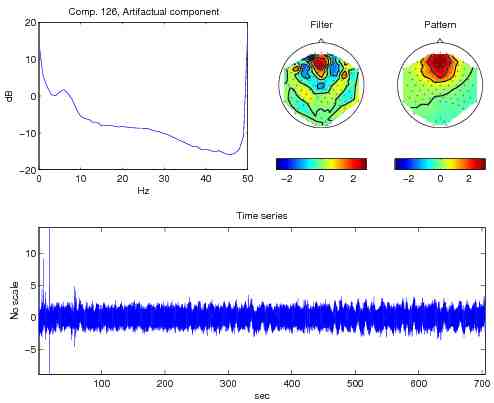

Supplement: Additional file 3 — TestComponents. Visualization of the 1080 independent components in the RT test data, together with the expert's labels. [file 1744-9081-7-30-S3.GZ › components_test/comp126.jpg]

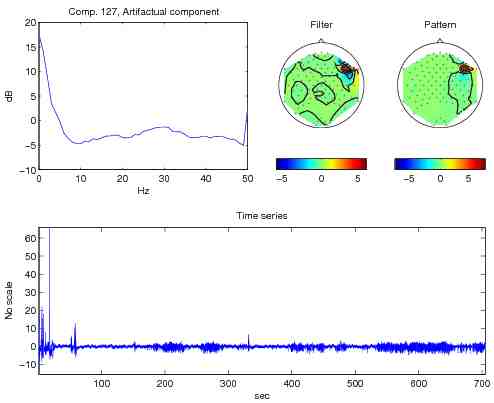

Supplement: Additional file 3 — TestComponents. Visualization of the 1080 independent components in the RT test data, together with the expert's labels. [file 1744-9081-7-30-S3.GZ › components_test/comp127.jpg]

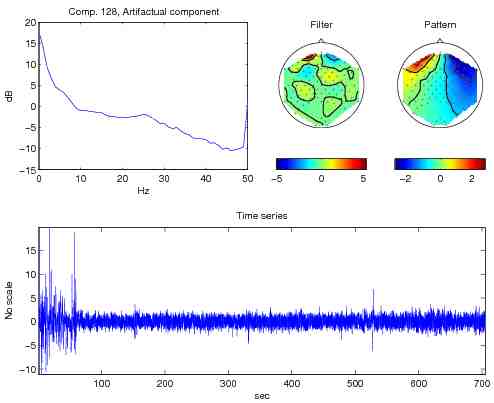

Supplement: Additional file 3 — TestComponents. Visualization of the 1080 independent components in the RT test data, together with the expert's labels. [file 1744-9081-7-30-S3.GZ › components_test/comp128.jpg]

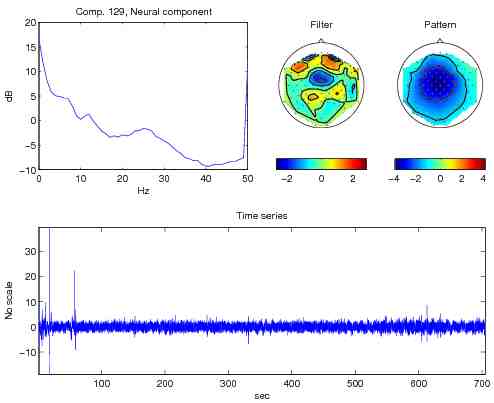

Supplement: Additional file 3 — TestComponents. Visualization of the 1080 independent components in the RT test data, together with the expert's labels. [file 1744-9081-7-30-S3.GZ › components_test/comp129.jpg]

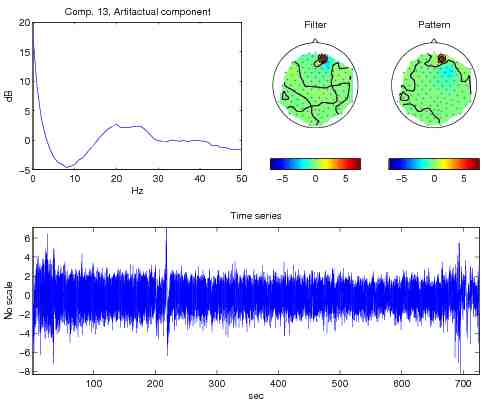

Supplement: Additional file 3 — TestComponents. Visualization of the 1080 independent components in the RT test data, together with the expert's labels. [file 1744-9081-7-30-S3.GZ › components_test/comp13.jpg]

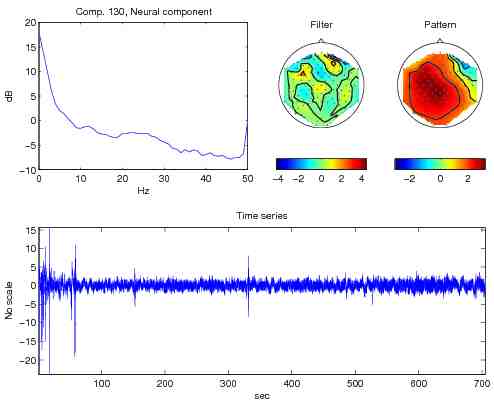

Supplement: Additional file 3 — TestComponents. Visualization of the 1080 independent components in the RT test data, together with the expert's labels. [file 1744-9081-7-30-S3.GZ › components_test/comp130.jpg]

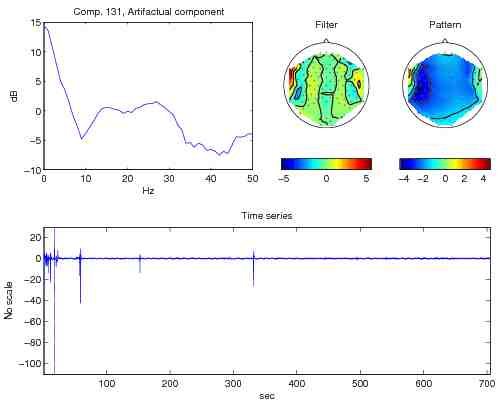

Supplement: Additional file 3 — TestComponents. Visualization of the 1080 independent components in the RT test data, together with the expert's labels. [file 1744-9081-7-30-S3.GZ › components_test/comp131.jpg]

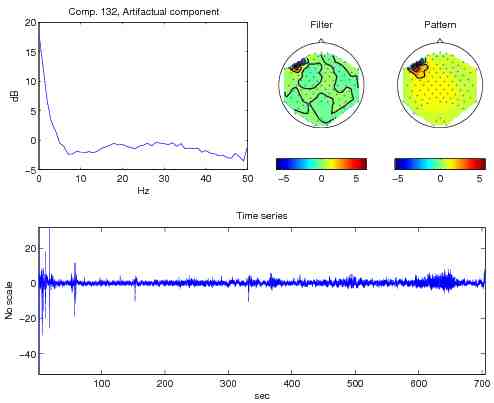

Supplement: Additional file 3 — TestComponents. Visualization of the 1080 independent components in the RT test data, together with the expert's labels. [file 1744-9081-7-30-S3.GZ › components_test/comp132.jpg]

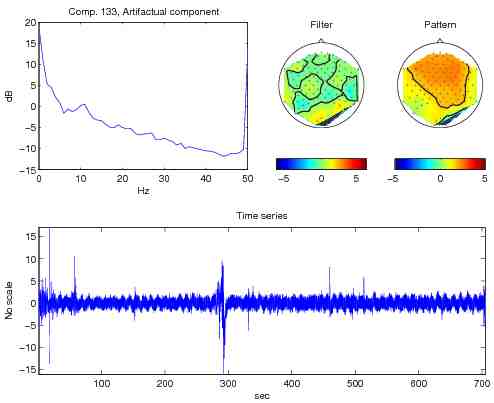

Supplement: Additional file 3 — TestComponents. Visualization of the 1080 independent components in the RT test data, together with the expert's labels. [file 1744-9081-7-30-S3.GZ › components_test/comp133.jpg]

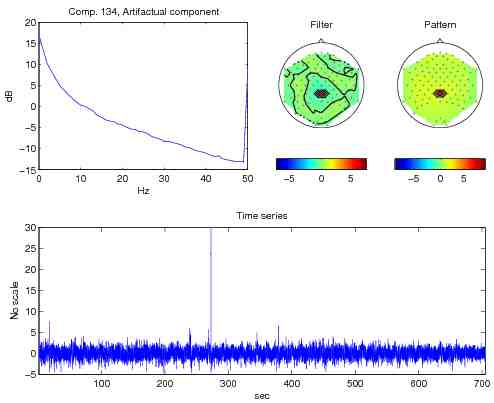

Supplement: Additional file 3 — TestComponents. Visualization of the 1080 independent components in the RT test data, together with the expert's labels. [file 1744-9081-7-30-S3.GZ › components_test/comp134.jpg]

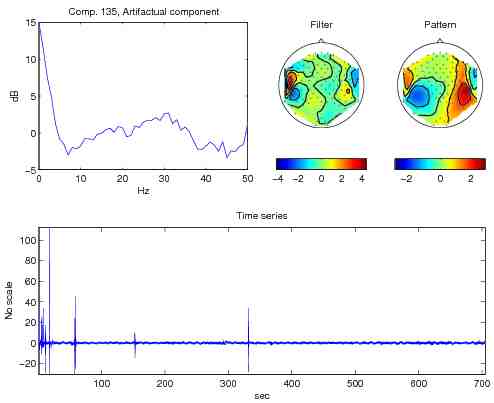

Supplement: Additional file 3 — TestComponents. Visualization of the 1080 independent components in the RT test data, together with the expert's labels. [file 1744-9081-7-30-S3.GZ › components_test/comp135.jpg]

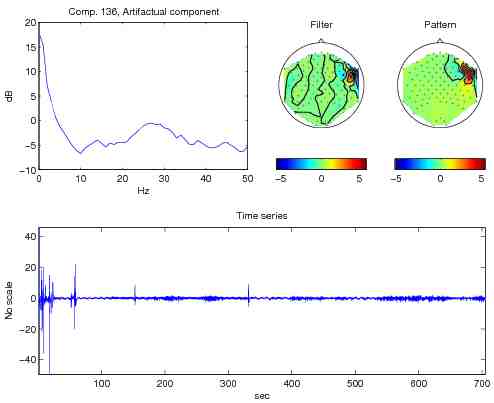

Supplement: Additional file 3 — TestComponents. Visualization of the 1080 independent components in the RT test data, together with the expert's labels. [file 1744-9081-7-30-S3.GZ › components_test/comp136.jpg]

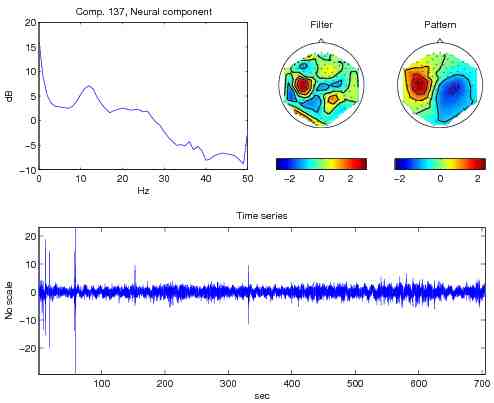

Supplement: Additional file 3 — TestComponents. Visualization of the 1080 independent components in the RT test data, together with the expert's labels. [file 1744-9081-7-30-S3.GZ › components_test/comp137.jpg]

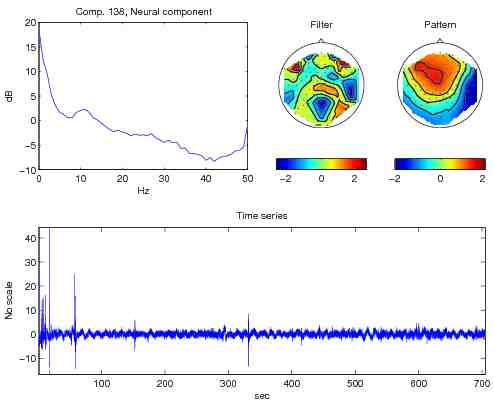

Supplement: Additional file 3 — TestComponents. Visualization of the 1080 independent components in the RT test data, together with the expert's labels. [file 1744-9081-7-30-S3.GZ › components_test/comp138.jpg]

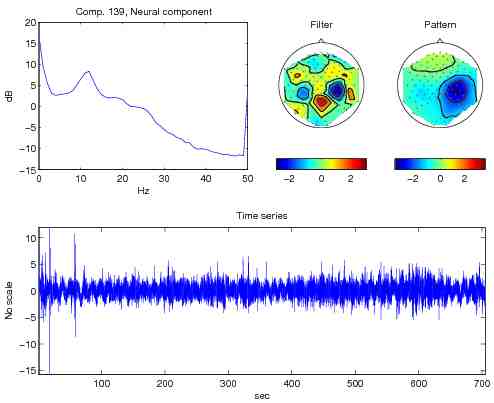

Supplement: Additional file 3 — TestComponents. Visualization of the 1080 independent components in the RT test data, together with the expert's labels. [file 1744-9081-7-30-S3.GZ › components_test/comp139.jpg]

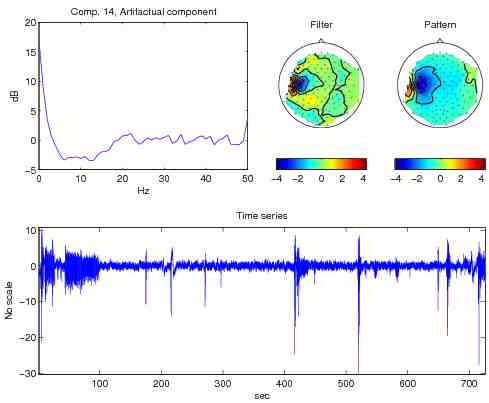

Supplement: Additional file 3 — TestComponents. Visualization of the 1080 independent components in the RT test data, together with the expert's labels. [file 1744-9081-7-30-S3.GZ › components_test/comp14.jpg]

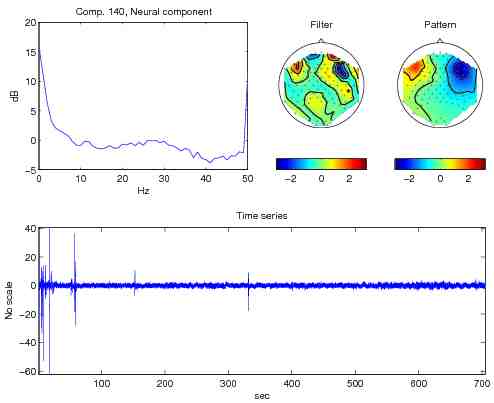

Supplement: Additional file 3 — TestComponents. Visualization of the 1080 independent components in the RT test data, together with the expert's labels. [file 1744-9081-7-30-S3.GZ › components_test/comp140.jpg]

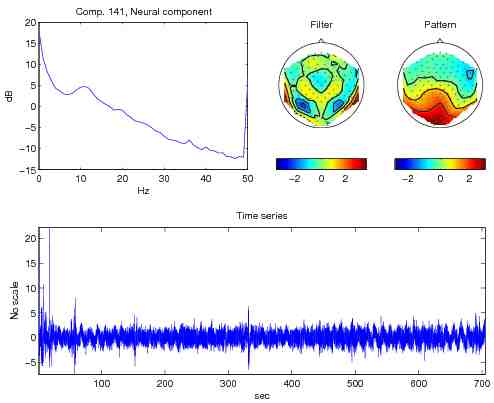

Supplement: Additional file 3 — TestComponents. Visualization of the 1080 independent components in the RT test data, together with the expert's labels. [file 1744-9081-7-30-S3.GZ › components_test/comp141.jpg]

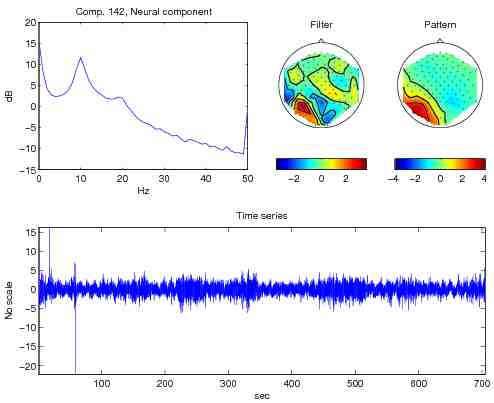

Supplement: Additional file 3 — TestComponents. Visualization of the 1080 independent components in the RT test data, together with the expert's labels. [file 1744-9081-7-30-S3.GZ › components_test/comp142.jpg]

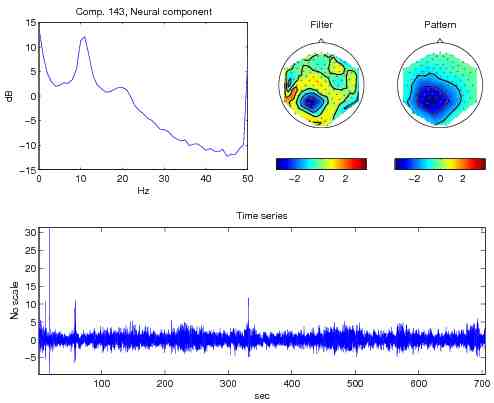

Supplement: Additional file 3 — TestComponents. Visualization of the 1080 independent components in the RT test data, together with the expert's labels. [file 1744-9081-7-30-S3.GZ › components_test/comp143.jpg]

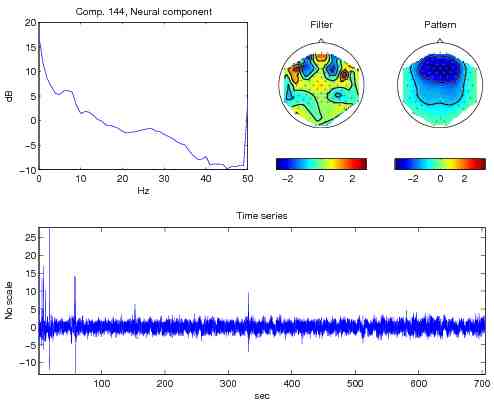

Supplement: Additional file 3 — TestComponents. Visualization of the 1080 independent components in the RT test data, together with the expert's labels. [file 1744-9081-7-30-S3.GZ › components_test/comp144.jpg]

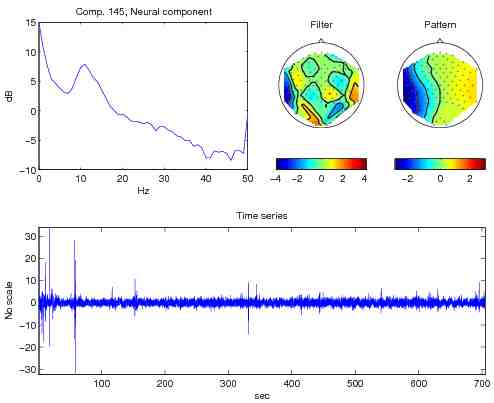

Supplement: Additional file 3 — TestComponents. Visualization of the 1080 independent components in the RT test data, together with the expert's labels. [file 1744-9081-7-30-S3.GZ › components_test/comp145.jpg]

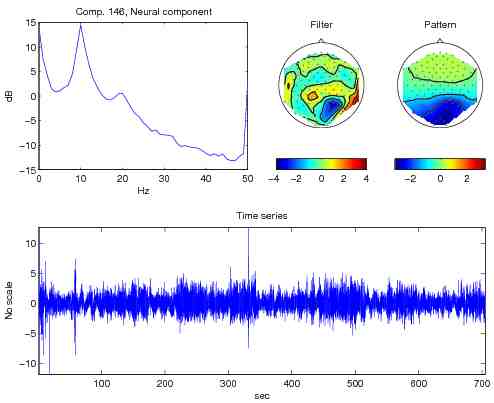

Supplement: Additional file 3 — TestComponents. Visualization of the 1080 independent components in the RT test data, together with the expert's labels. [file 1744-9081-7-30-S3.GZ › components_test/comp146.jpg]

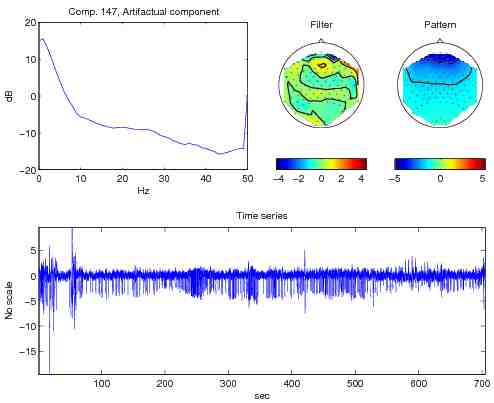

Supplement: Additional file 3 — TestComponents. Visualization of the 1080 independent components in the RT test data, together with the expert's labels. [file 1744-9081-7-30-S3.GZ › components_test/comp147.jpg]

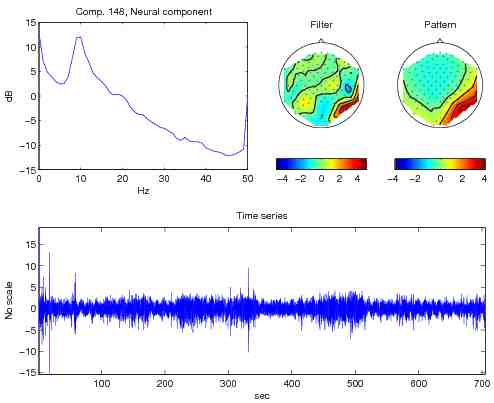

Supplement: Additional file 3 — TestComponents. Visualization of the 1080 independent components in the RT test data, together with the expert's labels. [file 1744-9081-7-30-S3.GZ › components_test/comp148.jpg]

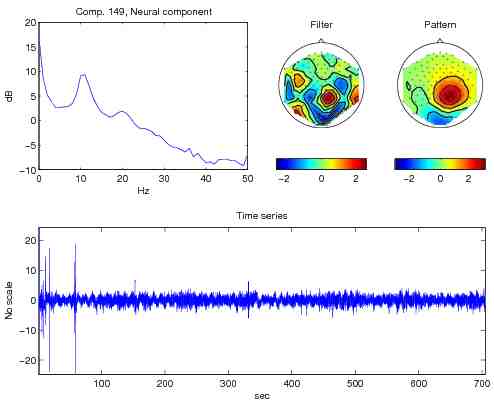

Supplement: Additional file 3 — TestComponents. Visualization of the 1080 independent components in the RT test data, together with the expert's labels. [file 1744-9081-7-30-S3.GZ › components_test/comp149.jpg]

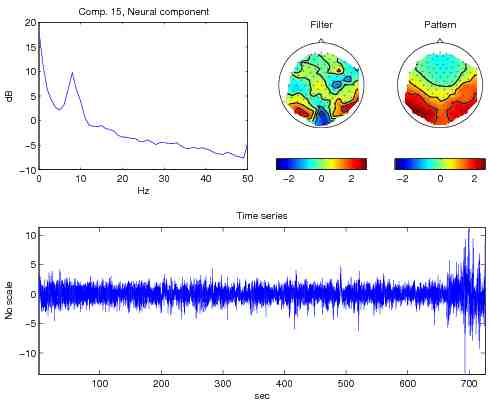

Supplement: Additional file 3 — TestComponents. Visualization of the 1080 independent components in the RT test data, together with the expert's labels. [file 1744-9081-7-30-S3.GZ › components_test/comp15.jpg]

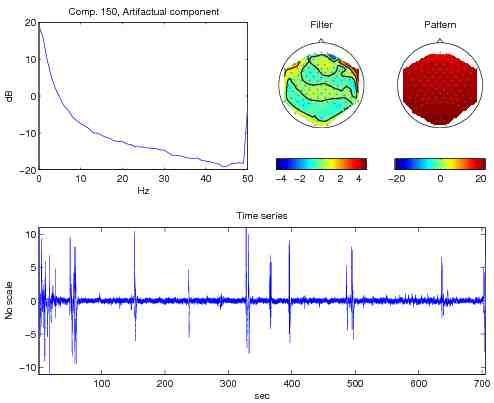

Supplement: Additional file 3 — TestComponents. Visualization of the 1080 independent components in the RT test data, together with the expert's labels. [file 1744-9081-7-30-S3.GZ › components_test/comp150.jpg]

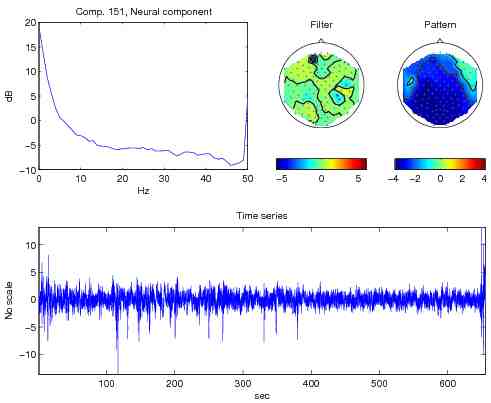

Supplement: Additional file 3 — TestComponents. Visualization of the 1080 independent components in the RT test data, together with the expert's labels. [file 1744-9081-7-30-S3.GZ › components_test/comp151.jpg]

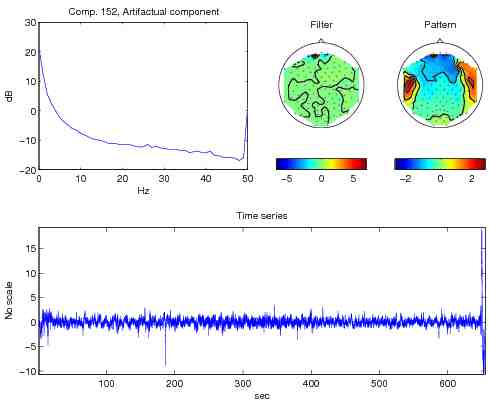

Supplement: Additional file 3 — TestComponents. Visualization of the 1080 independent components in the RT test data, together with the expert's labels. [file 1744-9081-7-30-S3.GZ › components_test/comp152.jpg]

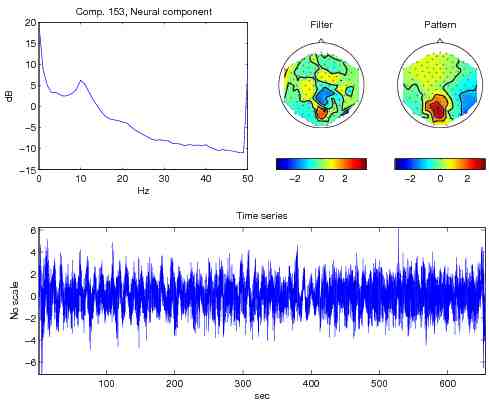

Supplement: Additional file 3 — TestComponents. Visualization of the 1080 independent components in the RT test data, together with the expert's labels. [file 1744-9081-7-30-S3.GZ › components_test/comp153.jpg]

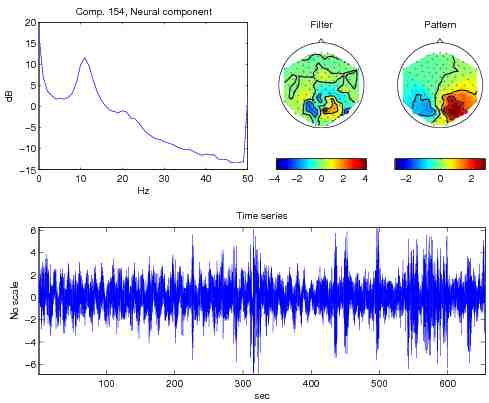

Supplement: Additional file 3 — TestComponents. Visualization of the 1080 independent components in the RT test data, together with the expert's labels. [file 1744-9081-7-30-S3.GZ › components_test/comp154.jpg]

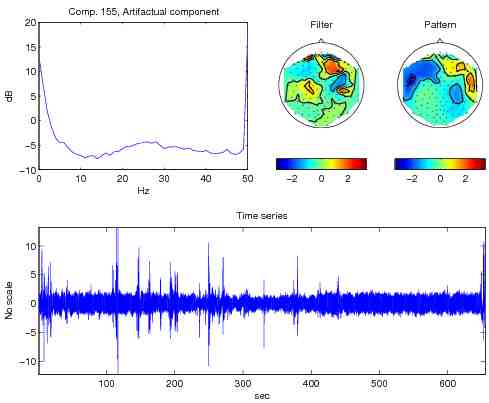

Supplement: Additional file 3 — TestComponents. Visualization of the 1080 independent components in the RT test data, together with the expert's labels. [file 1744-9081-7-30-S3.GZ › components_test/comp155.jpg]

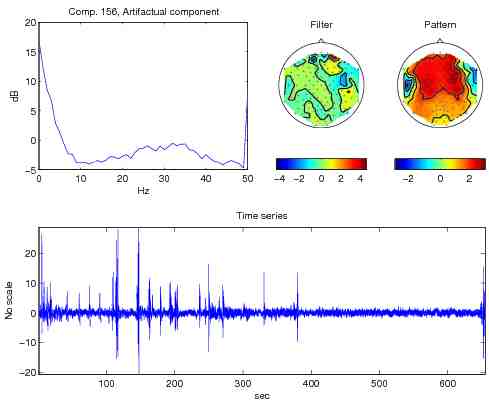

Supplement: Additional file 3 — TestComponents. Visualization of the 1080 independent components in the RT test data, together with the expert's labels. [file 1744-9081-7-30-S3.GZ › components_test/comp156.jpg]

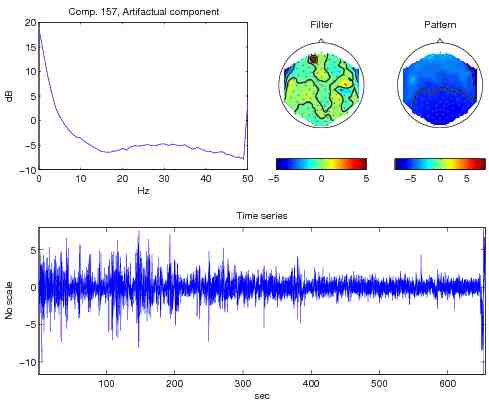

Supplement: Additional file 3 — TestComponents. Visualization of the 1080 independent components in the RT test data, together with the expert's labels. [file 1744-9081-7-30-S3.GZ › components_test/comp157.jpg]

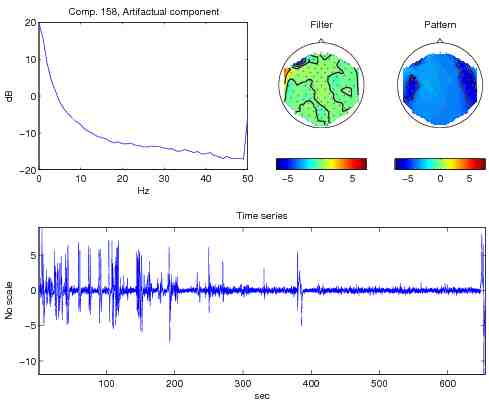

Supplement: Additional file 3 — TestComponents. Visualization of the 1080 independent components in the RT test data, together with the expert's labels. [file 1744-9081-7-30-S3.GZ › components_test/comp158.jpg]

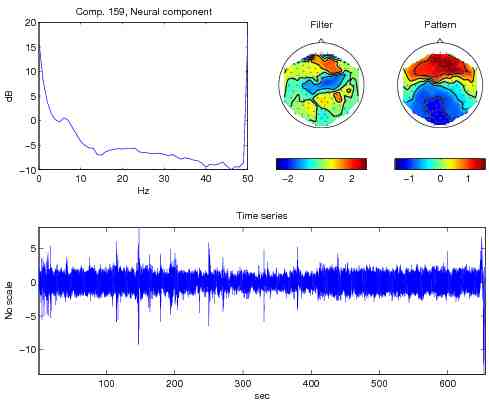

Supplement: Additional file 3 — TestComponents. Visualization of the 1080 independent components in the RT test data, together with the expert's labels. [file 1744-9081-7-30-S3.GZ › components_test/comp159.jpg]

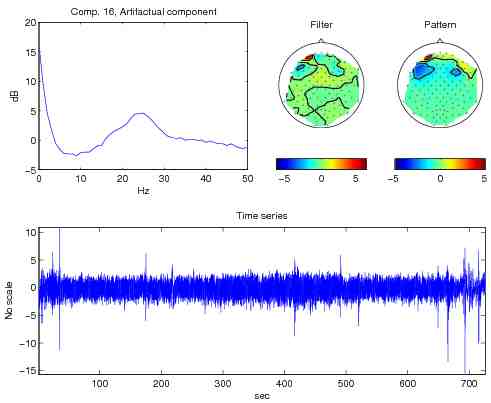

Supplement: Additional file 3 — TestComponents. Visualization of the 1080 independent components in the RT test data, together with the expert's labels. [file 1744-9081-7-30-S3.GZ › components_test/comp16.jpg]

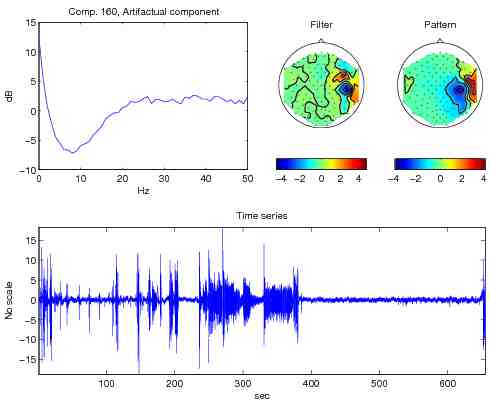

Supplement: Additional file 3 — TestComponents. Visualization of the 1080 independent components in the RT test data, together with the expert's labels. [file 1744-9081-7-30-S3.GZ › components_test/comp160.jpg]

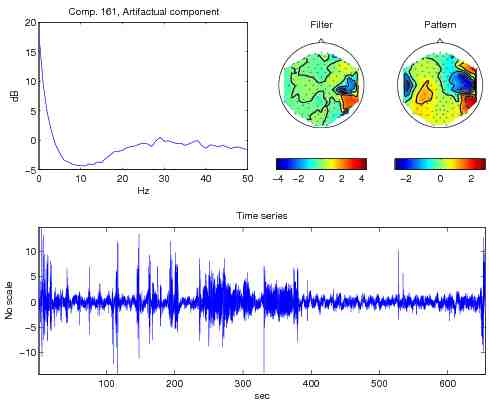

Supplement: Additional file 3 — TestComponents. Visualization of the 1080 independent components in the RT test data, together with the expert's labels. [file 1744-9081-7-30-S3.GZ › components_test/comp161.jpg]

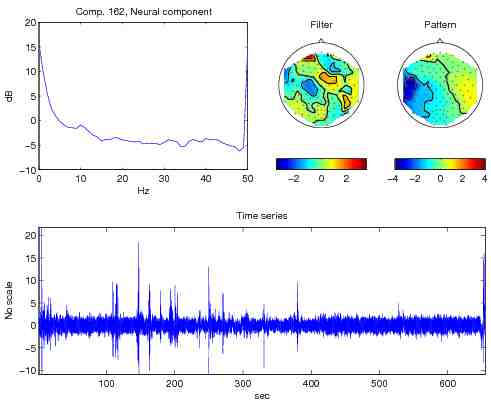

Supplement: Additional file 3 — TestComponents. Visualization of the 1080 independent components in the RT test data, together with the expert's labels. [file 1744-9081-7-30-S3.GZ › components_test/comp162.jpg]

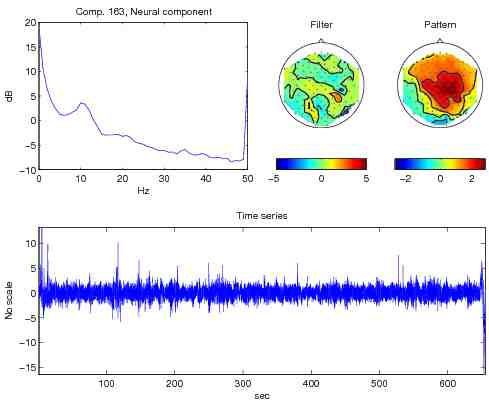

Supplement: Additional file 3 — TestComponents. Visualization of the 1080 independent components in the RT test data, together with the expert's labels. [file 1744-9081-7-30-S3.GZ › components_test/comp163.jpg]

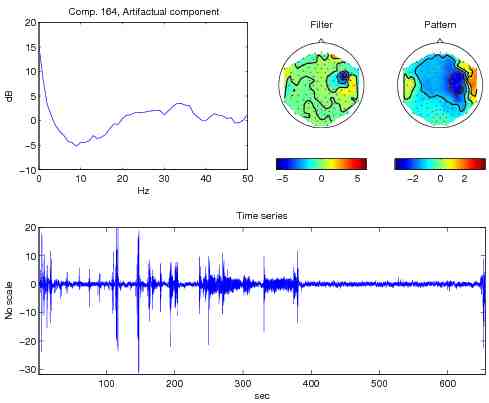

Supplement: Additional file 3 — TestComponents. Visualization of the 1080 independent components in the RT test data, together with the expert's labels. [file 1744-9081-7-30-S3.GZ › components_test/comp164.jpg]

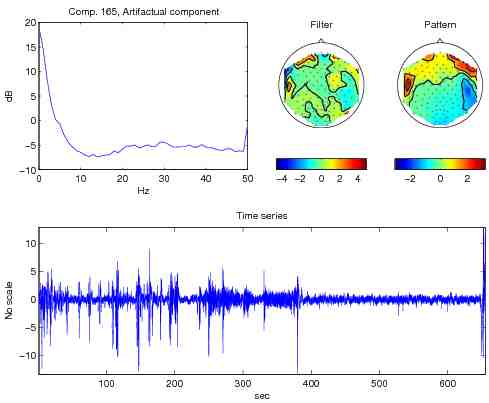

Supplement: Additional file 3 — TestComponents. Visualization of the 1080 independent components in the RT test data, together with the expert's labels. [file 1744-9081-7-30-S3.GZ › components_test/comp165.jpg]

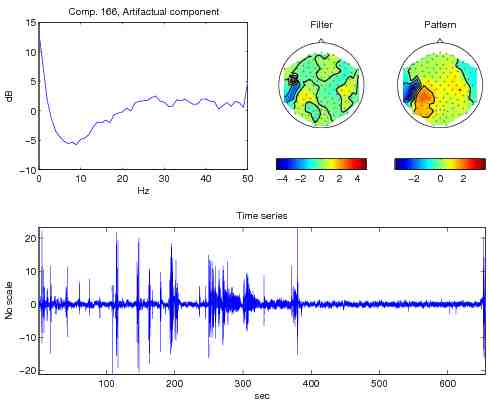

Supplement: Additional file 3 — TestComponents. Visualization of the 1080 independent components in the RT test data, together with the expert's labels. [file 1744-9081-7-30-S3.GZ › components_test/comp166.jpg]

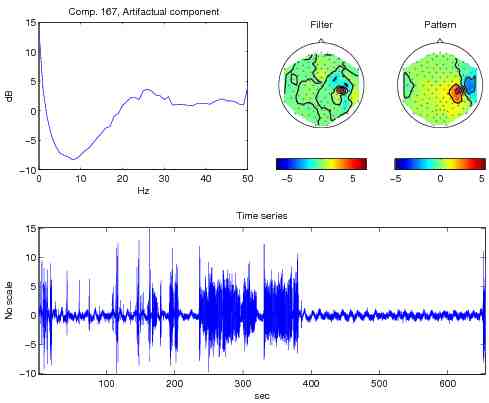

Supplement: Additional file 3 — TestComponents. Visualization of the 1080 independent components in the RT test data, together with the expert's labels. [file 1744-9081-7-30-S3.GZ › components_test/comp167.jpg]

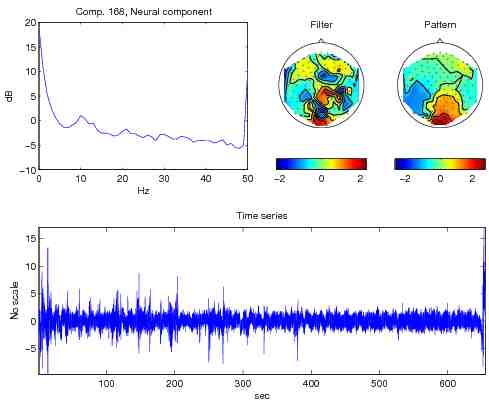

Supplement: Additional file 3 — TestComponents. Visualization of the 1080 independent components in the RT test data, together with the expert's labels. [file 1744-9081-7-30-S3.GZ › components_test/comp168.jpg]

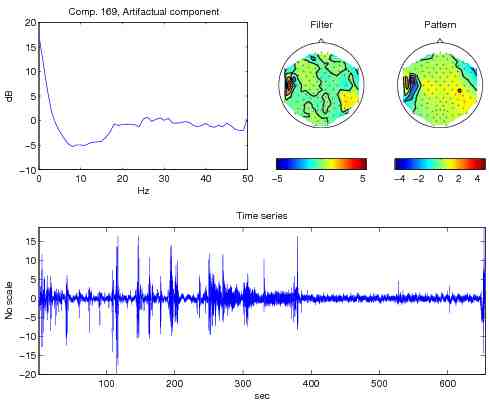

Supplement: Additional file 3 — TestComponents. Visualization of the 1080 independent components in the RT test data, together with the expert's labels. [file 1744-9081-7-30-S3.GZ › components_test/comp169.jpg]

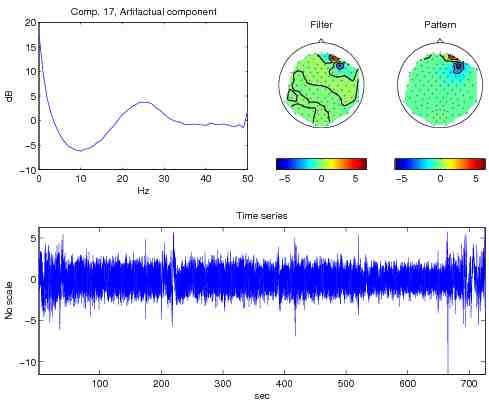

Supplement: Additional file 3 — TestComponents. Visualization of the 1080 independent components in the RT test data, together with the expert's labels. [file 1744-9081-7-30-S3.GZ › components_test/comp17.jpg]

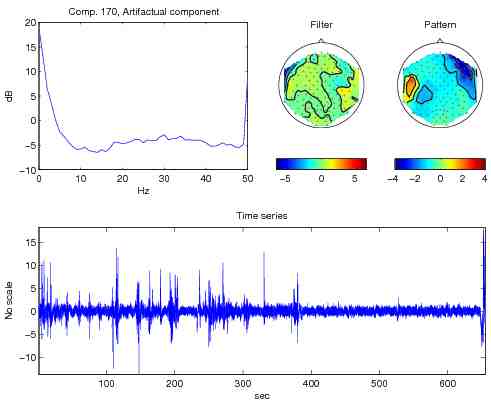

Supplement: Additional file 3 — TestComponents. Visualization of the 1080 independent components in the RT test data, together with the expert's labels. [file 1744-9081-7-30-S3.GZ › components_test/comp170.jpg]

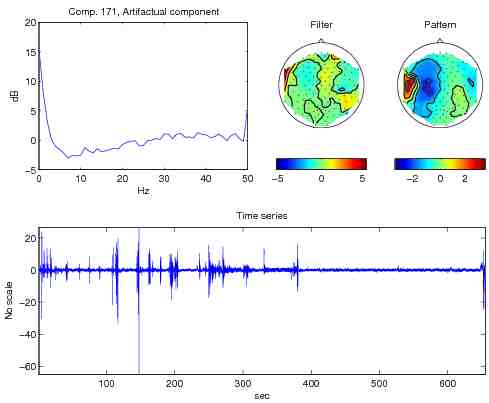

Supplement: Additional file 3 — TestComponents. Visualization of the 1080 independent components in the RT test data, together with the expert's labels. [file 1744-9081-7-30-S3.GZ › components_test/comp171.jpg]

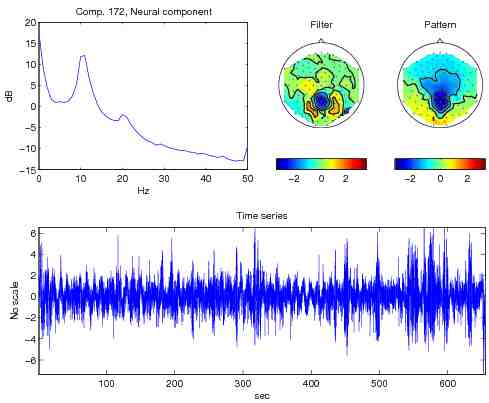

Supplement: Additional file 3 — TestComponents. Visualization of the 1080 independent components in the RT test data, together with the expert's labels. [file 1744-9081-7-30-S3.GZ › components_test/comp172.jpg]

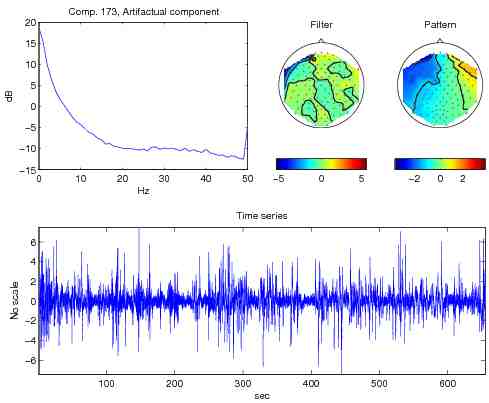

Supplement: Additional file 3 — TestComponents. Visualization of the 1080 independent components in the RT test data, together with the expert's labels. [file 1744-9081-7-30-S3.GZ › components_test/comp173.jpg]

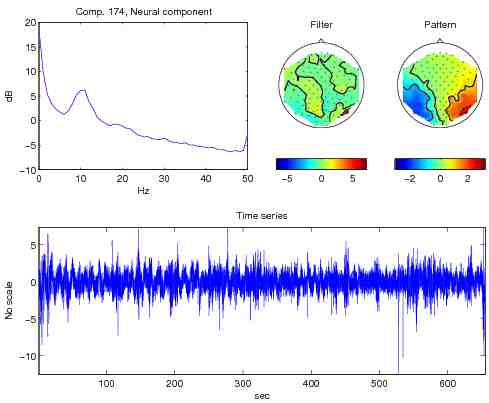

Supplement: Additional file 3 — TestComponents. Visualization of the 1080 independent components in the RT test data, together with the expert's labels. [file 1744-9081-7-30-S3.GZ › components_test/comp174.jpg]

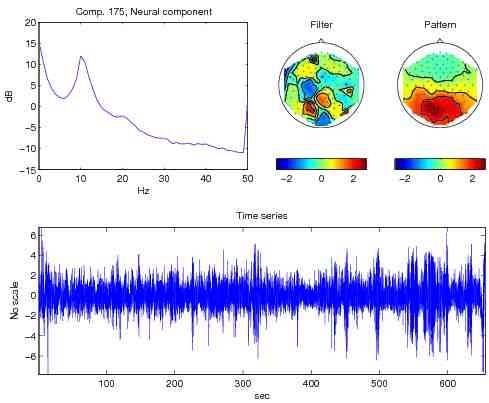

Supplement: Additional file 3 — TestComponents. Visualization of the 1080 independent components in the RT test data, together with the expert's labels. [file 1744-9081-7-30-S3.GZ › components_test/comp175.jpg]

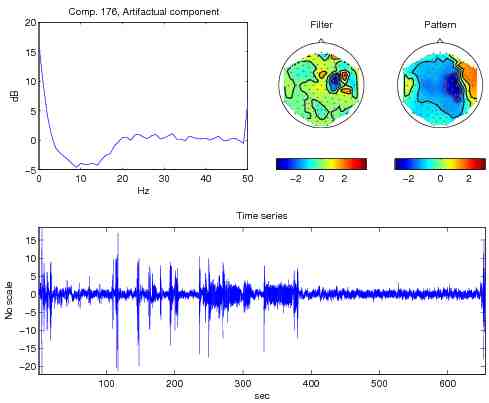

Supplement: Additional file 3 — TestComponents. Visualization of the 1080 independent components in the RT test data, together with the expert's labels. [file 1744-9081-7-30-S3.GZ › components_test/comp176.jpg]

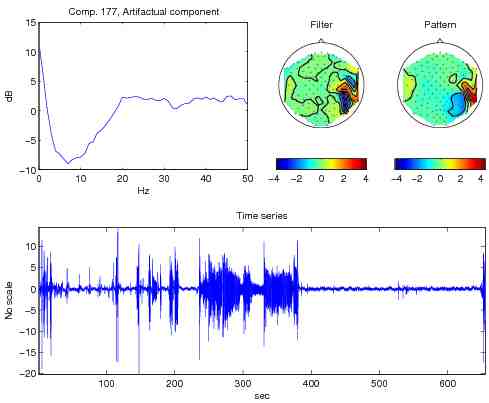

Supplement: Additional file 3 — TestComponents. Visualization of the 1080 independent components in the RT test data, together with the expert's labels. [file 1744-9081-7-30-S3.GZ › components_test/comp177.jpg]

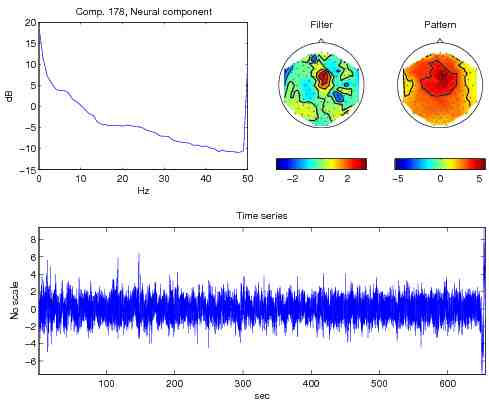

Supplement: Additional file 3 — TestComponents. Visualization of the 1080 independent components in the RT test data, together with the expert's labels. [file 1744-9081-7-30-S3.GZ › components_test/comp178.jpg]

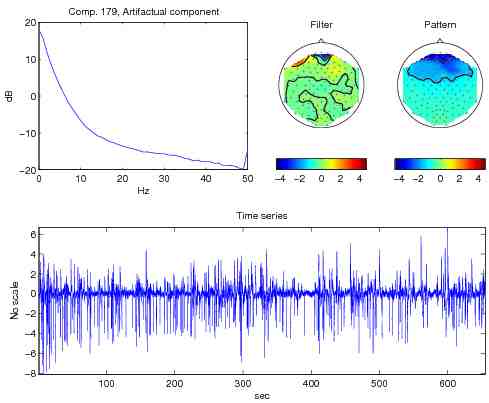

Supplement: Additional file 3 — TestComponents. Visualization of the 1080 independent components in the RT test data, together with the expert's labels. [file 1744-9081-7-30-S3.GZ › components_test/comp179.jpg]

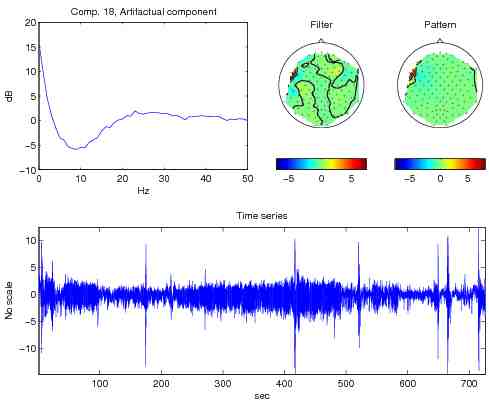

Supplement: Additional file 3 — TestComponents. Visualization of the 1080 independent components in the RT test data, together with the expert's labels. [file 1744-9081-7-30-S3.GZ › components_test/comp18.jpg]

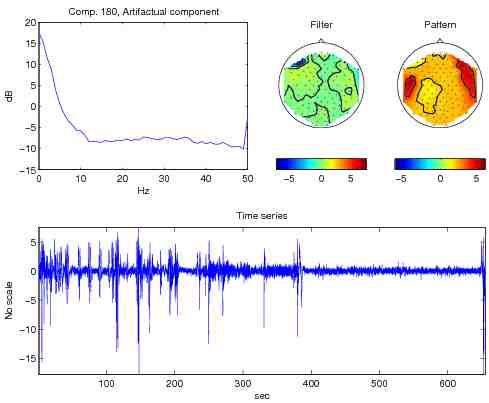

Supplement: Additional file 3 — TestComponents. Visualization of the 1080 independent components in the RT test data, together with the expert's labels. [file 1744-9081-7-30-S3.GZ › components_test/comp180.jpg]

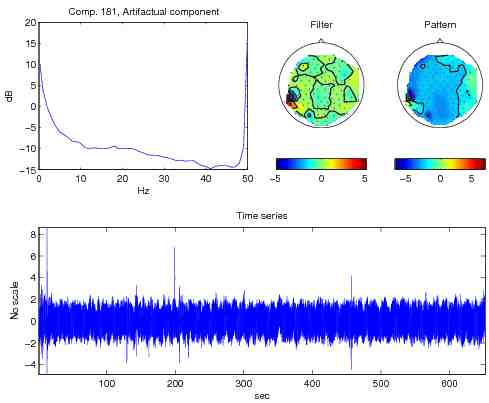

Supplement: Additional file 3 — TestComponents. Visualization of the 1080 independent components in the RT test data, together with the expert's labels. [file 1744-9081-7-30-S3.GZ › components_test/comp181.jpg]

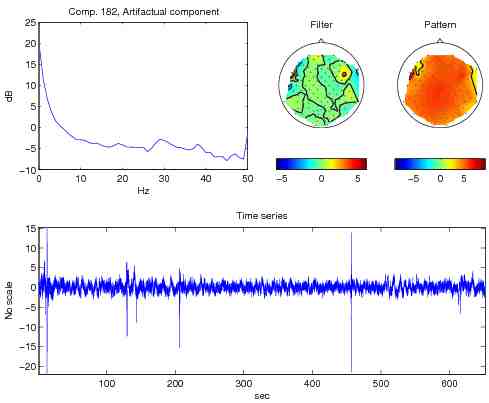

Supplement: Additional file 3 — TestComponents. Visualization of the 1080 independent components in the RT test data, together with the expert's labels. [file 1744-9081-7-30-S3.GZ › components_test/comp182.jpg]

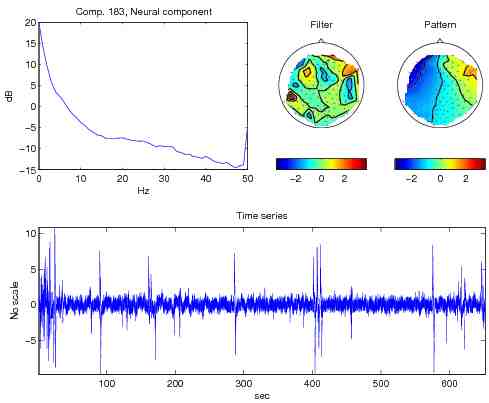

Supplement: Additional file 3 — TestComponents. Visualization of the 1080 independent components in the RT test data, together with the expert's labels. [file 1744-9081-7-30-S3.GZ › components_test/comp183.jpg]

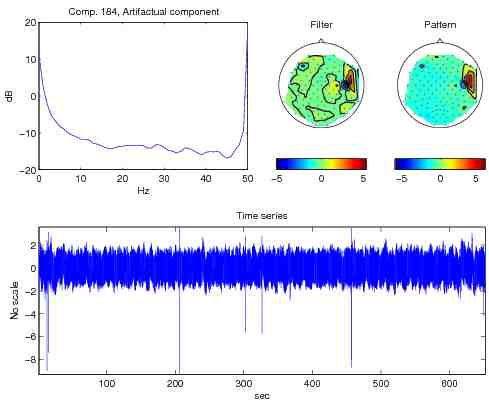

Supplement: Additional file 3 — TestComponents. Visualization of the 1080 independent components in the RT test data, together with the expert's labels. [file 1744-9081-7-30-S3.GZ › components_test/comp184.jpg]

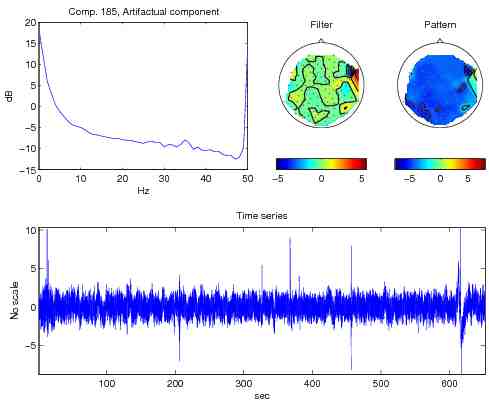

Supplement: Additional file 3 — TestComponents. Visualization of the 1080 independent components in the RT test data, together with the expert's labels. [file 1744-9081-7-30-S3.GZ › components_test/comp185.jpg]

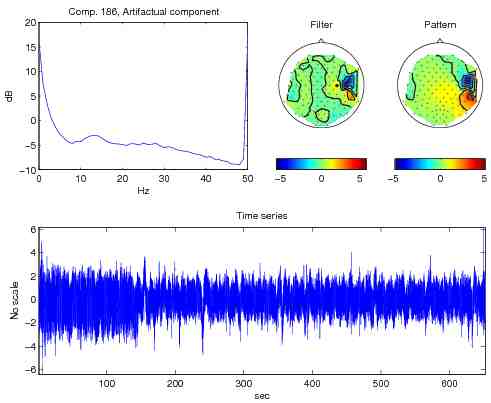

Supplement: Additional file 3 — TestComponents. Visualization of the 1080 independent components in the RT test data, together with the expert's labels. [file 1744-9081-7-30-S3.GZ › components_test/comp186.jpg]

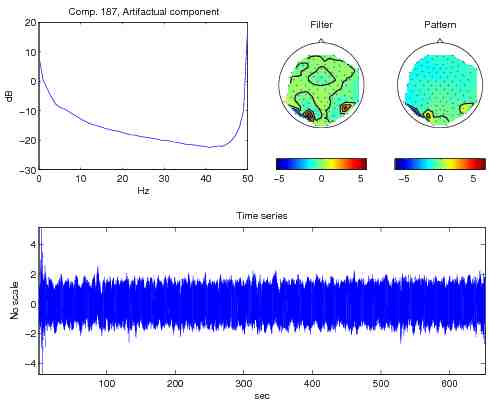

Supplement: Additional file 3 — TestComponents. Visualization of the 1080 independent components in the RT test data, together with the expert's labels. [file 1744-9081-7-30-S3.GZ › components_test/comp187.jpg]

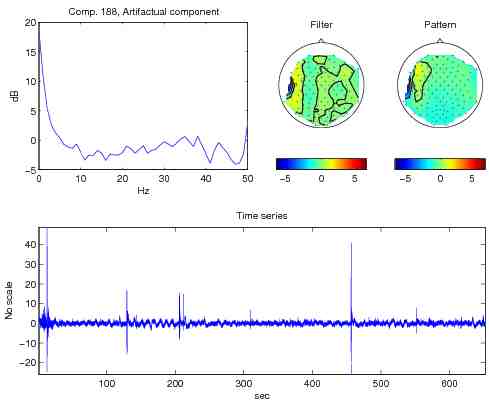

Supplement: Additional file 3 — TestComponents. Visualization of the 1080 independent components in the RT test data, together with the expert's labels. [file 1744-9081-7-30-S3.GZ › components_test/comp188.jpg]

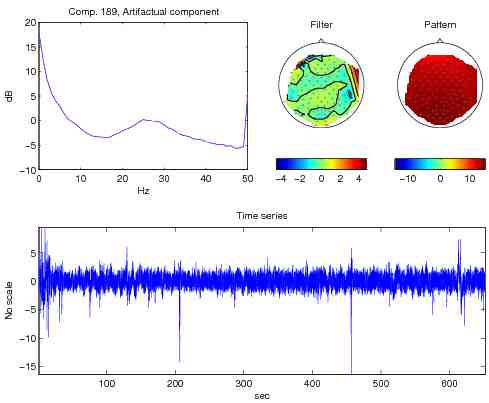

Supplement: Additional file 3 — TestComponents. Visualization of the 1080 independent components in the RT test data, together with the expert's labels. [file 1744-9081-7-30-S3.GZ › components_test/comp189.jpg]

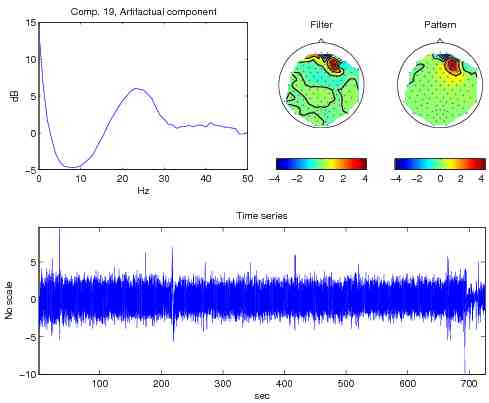

Supplement: Additional file 3 — TestComponents. Visualization of the 1080 independent components in the RT test data, together with the expert's labels. [file 1744-9081-7-30-S3.GZ › components_test/comp19.jpg]

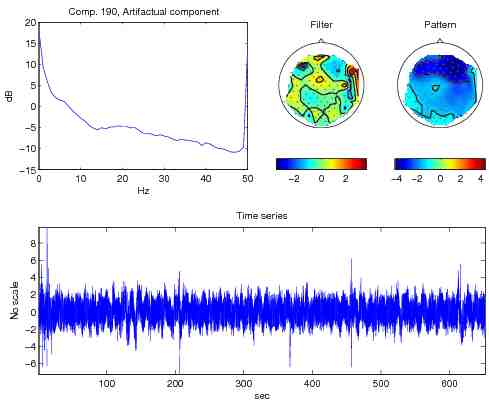

Supplement: Additional file 3 — TestComponents. Visualization of the 1080 independent components in the RT test data, together with the expert's labels. [file 1744-9081-7-30-S3.GZ › components_test/comp190.jpg]

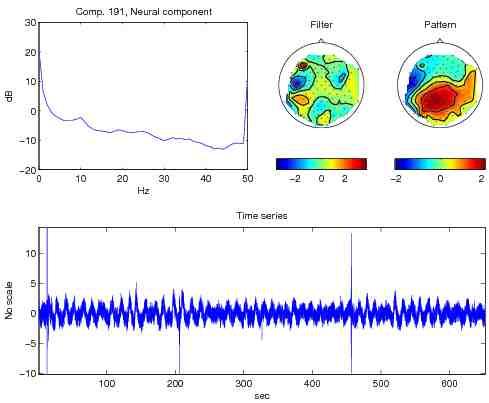

Supplement: Additional file 3 — TestComponents. Visualization of the 1080 independent components in the RT test data, together with the expert's labels. [file 1744-9081-7-30-S3.GZ › components_test/comp191.jpg]

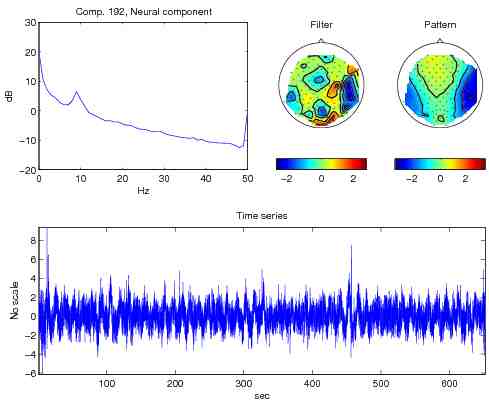

Supplement: Additional file 3 — TestComponents. Visualization of the 1080 independent components in the RT test data, together with the expert's labels. [file 1744-9081-7-30-S3.GZ › components_test/comp192.jpg]

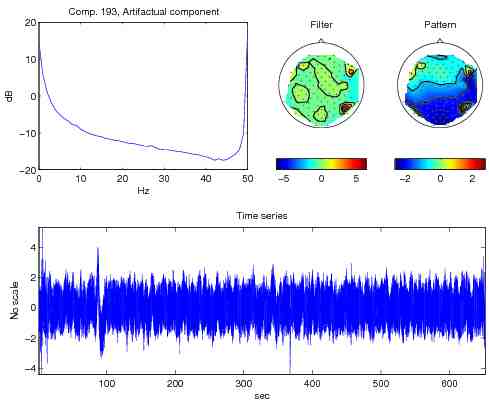

Supplement: Additional file 3 — TestComponents. Visualization of the 1080 independent components in the RT test data, together with the expert's labels. [file 1744-9081-7-30-S3.GZ › components_test/comp193.jpg]

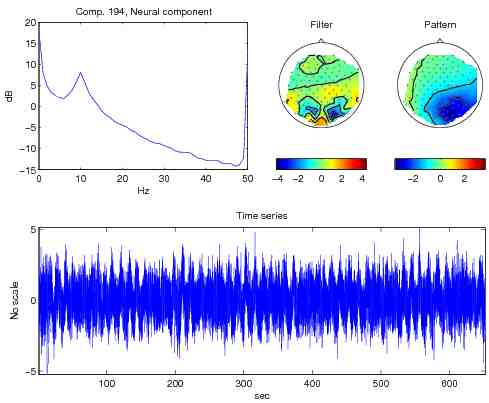

Supplement: Additional file 3 — TestComponents. Visualization of the 1080 independent components in the RT test data, together with the expert's labels. [file 1744-9081-7-30-S3.GZ › components_test/comp194.jpg]

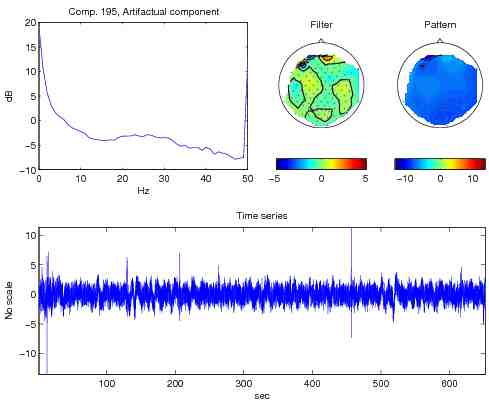

Supplement: Additional file 3 — TestComponents. Visualization of the 1080 independent components in the RT test data, together with the expert's labels. [file 1744-9081-7-30-S3.GZ › components_test/comp195.jpg]

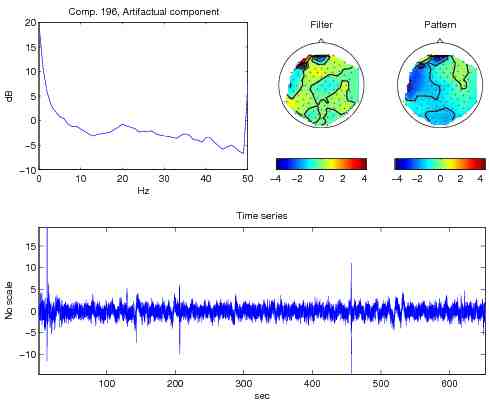

Supplement: Additional file 3 — TestComponents. Visualization of the 1080 independent components in the RT test data, together with the expert's labels. [file 1744-9081-7-30-S3.GZ › components_test/comp196.jpg]

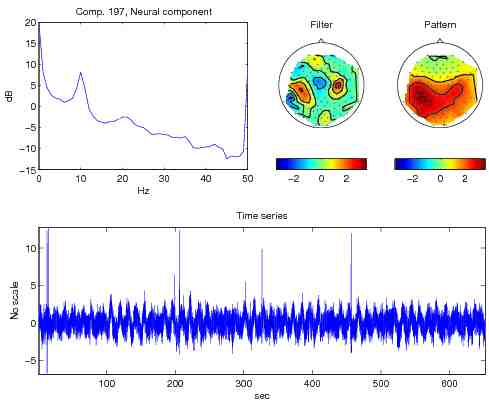

Supplement: Additional file 3 — TestComponents. Visualization of the 1080 independent components in the RT test data, together with the expert's labels. [file 1744-9081-7-30-S3.GZ › components_test/comp197.jpg]

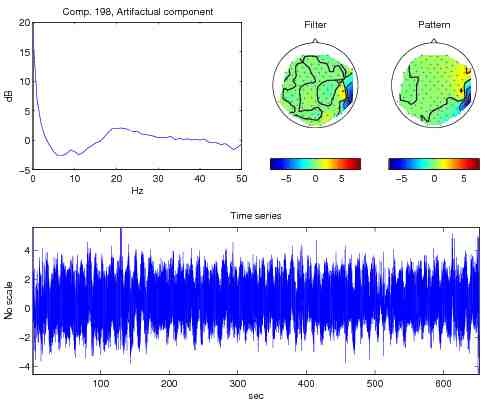

Supplement: Additional file 3 — TestComponents. Visualization of the 1080 independent components in the RT test data, together with the expert's labels. [file 1744-9081-7-30-S3.GZ › components_test/comp198.jpg]

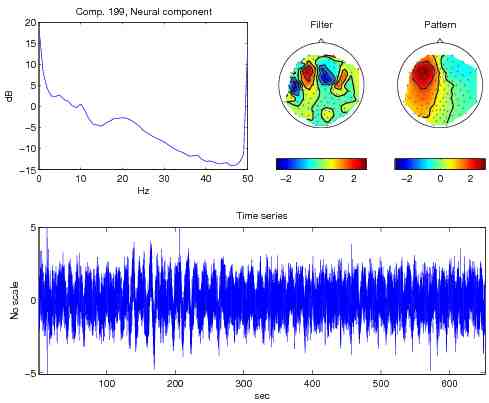

Supplement: Additional file 3 — TestComponents. Visualization of the 1080 independent components in the RT test data, together with the expert's labels. [file 1744-9081-7-30-S3.GZ › components_test/comp199.jpg]

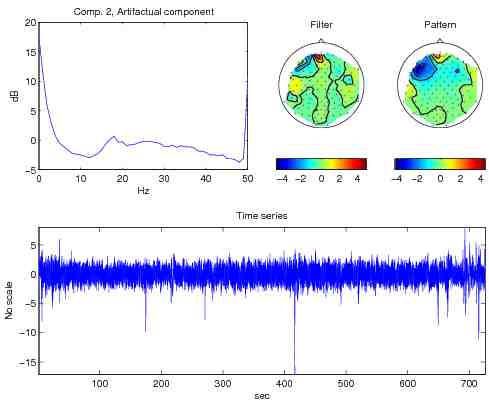

Supplement: Additional file 3 — TestComponents. Visualization of the 1080 independent components in the RT test data, together with the expert's labels. [file 1744-9081-7-30-S3.GZ › components_test/comp2.jpg]

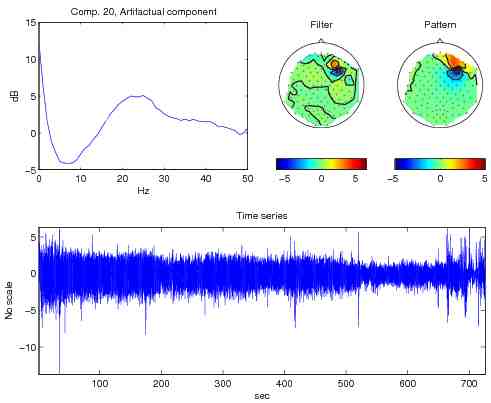

Supplement: Additional file 3 — TestComponents. Visualization of the 1080 independent components in the RT test data, together with the expert's labels. [file 1744-9081-7-30-S3.GZ › components_test/comp20.jpg]

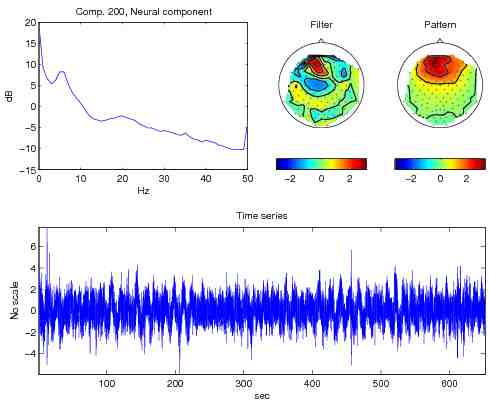

Supplement: Additional file 3 — TestComponents. Visualization of the 1080 independent components in the RT test data, together with the expert's labels. [file 1744-9081-7-30-S3.GZ › components_test/comp200.jpg]

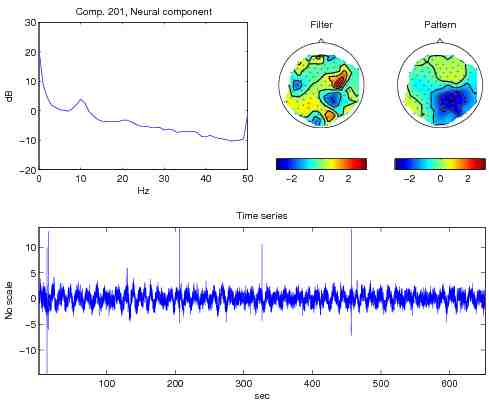

Supplement: Additional file 3 — TestComponents. Visualization of the 1080 independent components in the RT test data, together with the expert's labels. [file 1744-9081-7-30-S3.GZ › components_test/comp201.jpg]

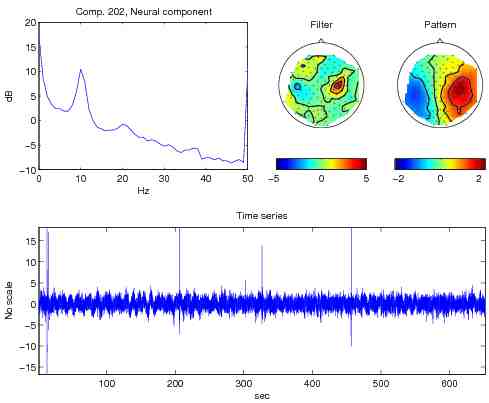

Supplement: Additional file 3 — TestComponents. Visualization of the 1080 independent components in the RT test data, together with the expert's labels. [file 1744-9081-7-30-S3.GZ › components_test/comp202.jpg]

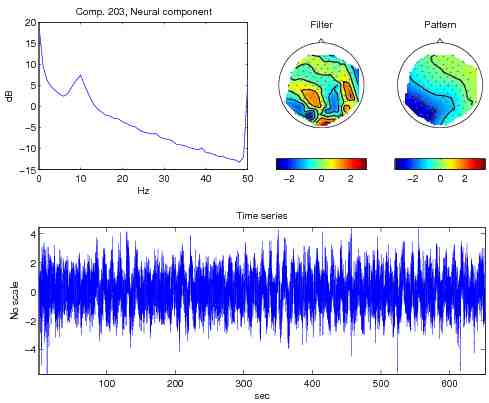

Supplement: Additional file 3 — TestComponents. Visualization of the 1080 independent components in the RT test data, together with the expert's labels. [file 1744-9081-7-30-S3.GZ › components_test/comp203.jpg]

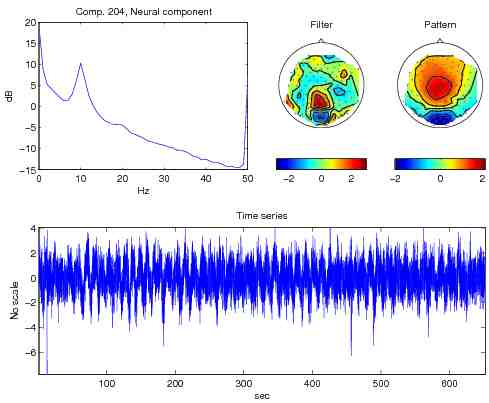

Supplement: Additional file 3 — TestComponents. Visualization of the 1080 independent components in the RT test data, together with the expert's labels. [file 1744-9081-7-30-S3.GZ › components_test/comp204.jpg]

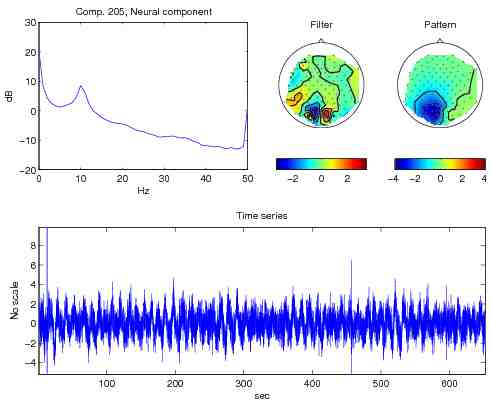

Supplement: Additional file 3 — TestComponents. Visualization of the 1080 independent components in the RT test data, together with the expert's labels. [file 1744-9081-7-30-S3.GZ › components_test/comp205.jpg]
