# Supplementary figures and images for: Automatic Classification of Artifactual ICA-Components for Artifact Removal in EEG Signals (part 3 of 5)
Source: Behav Brain Funct. 2011 Aug 2;7:30. doi: 10.1186/1744-9081-7-30 (PMC3175453; doi:10.1186/1744-9081-7-30)

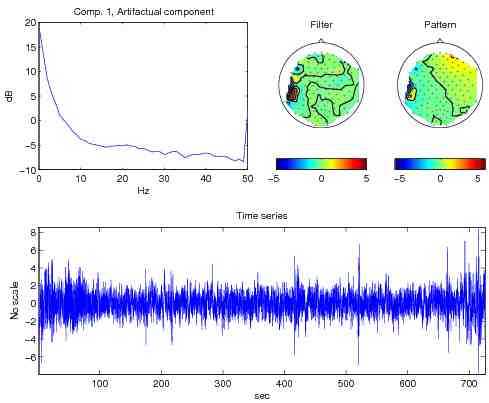

Supplement: Additional file 3 — TestComponents. Visualization of the 1080 independent components in the RT test data, together with the expert's labels. [file 1744-9081-7-30-S3.GZ › components_test/comp1.jpg]

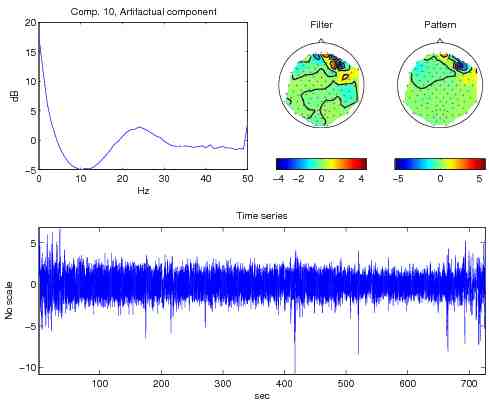

Supplement: Additional file 3 — TestComponents. Visualization of the 1080 independent components in the RT test data, together with the expert's labels. [file 1744-9081-7-30-S3.GZ › components_test/comp10.jpg]

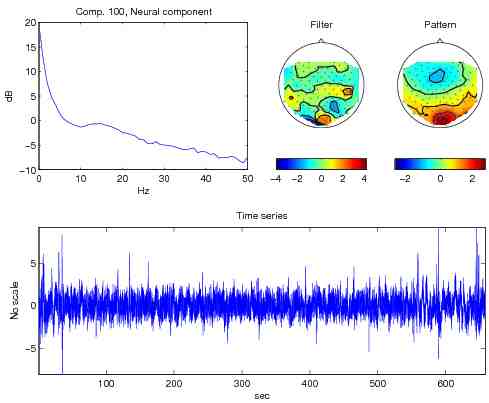

Supplement: Additional file 3 — TestComponents. Visualization of the 1080 independent components in the RT test data, together with the expert's labels. [file 1744-9081-7-30-S3.GZ › components_test/comp100.jpg]

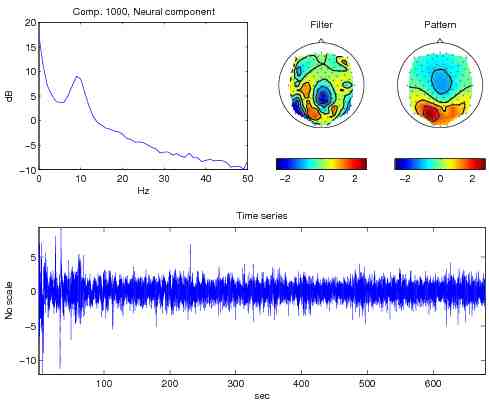

Supplement: Additional file 3 — TestComponents. Visualization of the 1080 independent components in the RT test data, together with the expert's labels. [file 1744-9081-7-30-S3.GZ › components_test/comp1000.jpg]

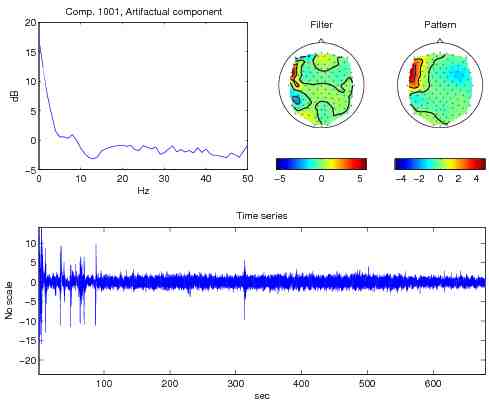

Supplement: Additional file 3 — TestComponents. Visualization of the 1080 independent components in the RT test data, together with the expert's labels. [file 1744-9081-7-30-S3.GZ › components_test/comp1001.jpg]

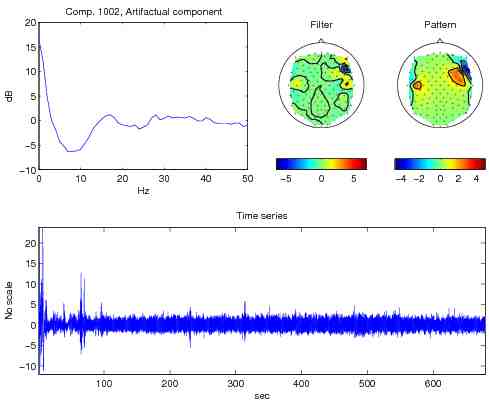

Supplement: Additional file 3 — TestComponents. Visualization of the 1080 independent components in the RT test data, together with the expert's labels. [file 1744-9081-7-30-S3.GZ › components_test/comp1002.jpg]

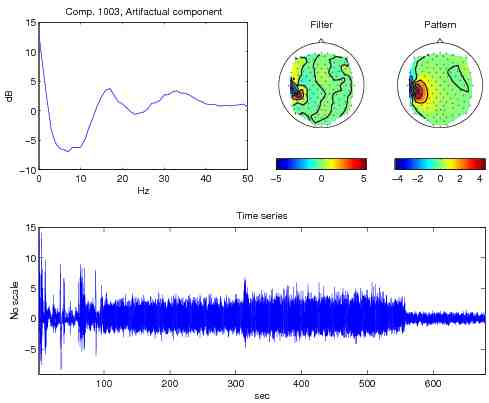

Supplement: Additional file 3 — TestComponents. Visualization of the 1080 independent components in the RT test data, together with the expert's labels. [file 1744-9081-7-30-S3.GZ › components_test/comp1003.jpg]

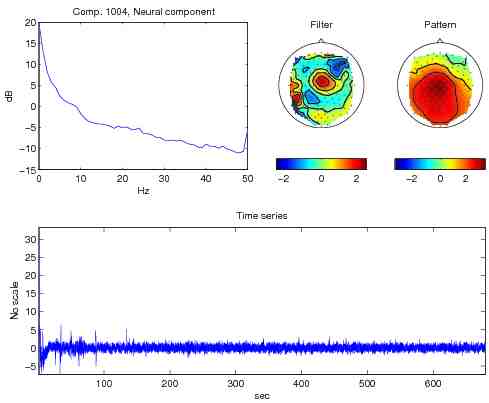

Supplement: Additional file 3 — TestComponents. Visualization of the 1080 independent components in the RT test data, together with the expert's labels. [file 1744-9081-7-30-S3.GZ › components_test/comp1004.jpg]

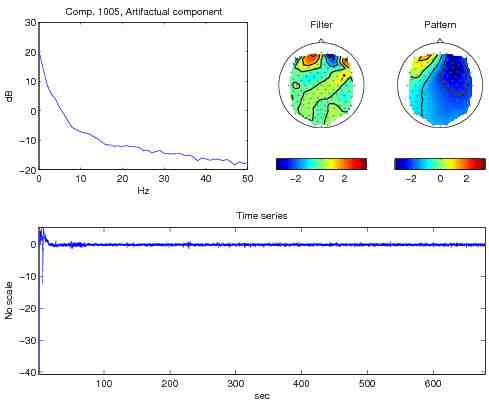

Supplement: Additional file 3 — TestComponents. Visualization of the 1080 independent components in the RT test data, together with the expert's labels. [file 1744-9081-7-30-S3.GZ › components_test/comp1005.jpg]

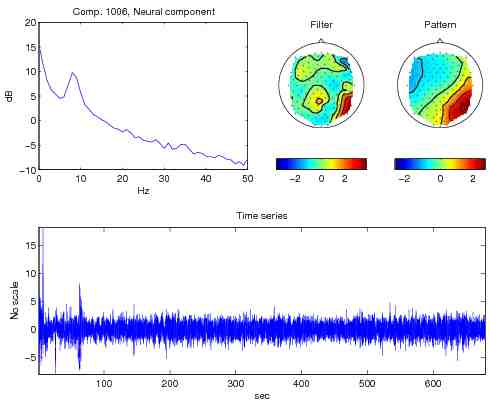

Supplement: Additional file 3 — TestComponents. Visualization of the 1080 independent components in the RT test data, together with the expert's labels. [file 1744-9081-7-30-S3.GZ › components_test/comp1006.jpg]

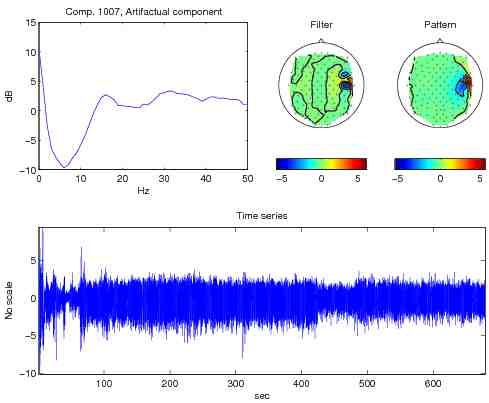

Supplement: Additional file 3 — TestComponents. Visualization of the 1080 independent components in the RT test data, together with the expert's labels. [file 1744-9081-7-30-S3.GZ › components_test/comp1007.jpg]

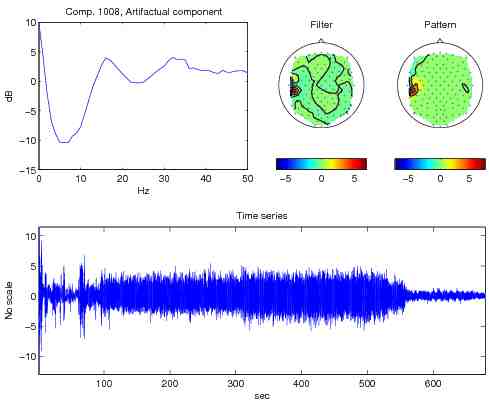

Supplement: Additional file 3 — TestComponents. Visualization of the 1080 independent components in the RT test data, together with the expert's labels. [file 1744-9081-7-30-S3.GZ › components_test/comp1008.jpg]

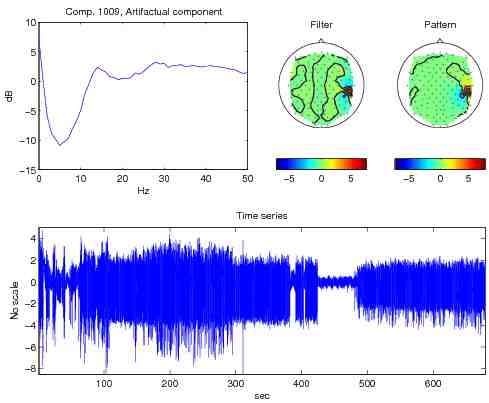

Supplement: Additional file 3 — TestComponents. Visualization of the 1080 independent components in the RT test data, together with the expert's labels. [file 1744-9081-7-30-S3.GZ › components_test/comp1009.jpg]

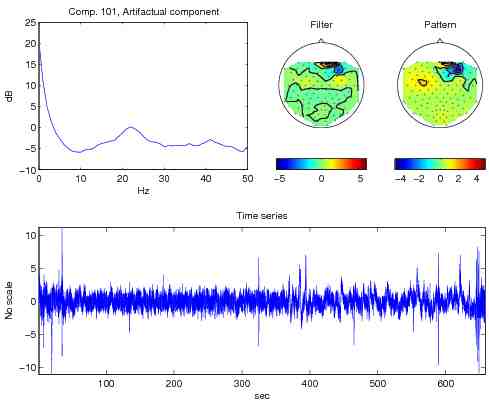

Supplement: Additional file 3 — TestComponents. Visualization of the 1080 independent components in the RT test data, together with the expert's labels. [file 1744-9081-7-30-S3.GZ › components_test/comp101.jpg]

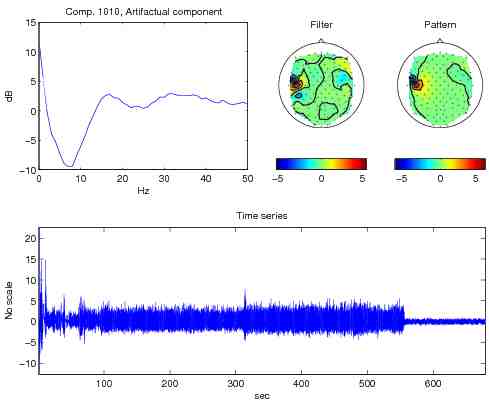

Supplement: Additional file 3 — TestComponents. Visualization of the 1080 independent components in the RT test data, together with the expert's labels. [file 1744-9081-7-30-S3.GZ › components_test/comp1010.jpg]

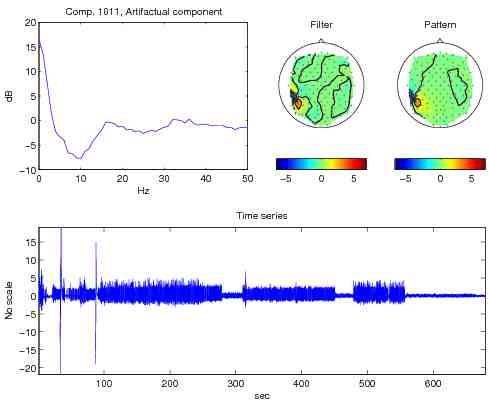

Supplement: Additional file 3 — TestComponents. Visualization of the 1080 independent components in the RT test data, together with the expert's labels. [file 1744-9081-7-30-S3.GZ › components_test/comp1011.jpg]

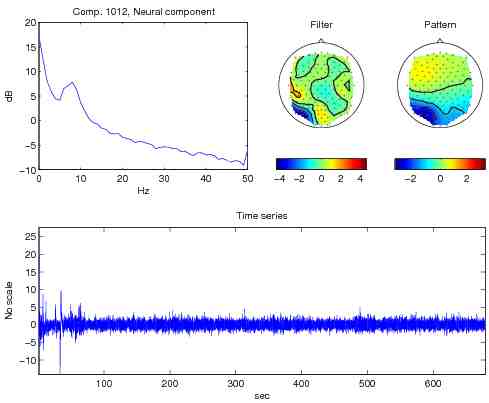

Supplement: Additional file 3 — TestComponents. Visualization of the 1080 independent components in the RT test data, together with the expert's labels. [file 1744-9081-7-30-S3.GZ › components_test/comp1012.jpg]

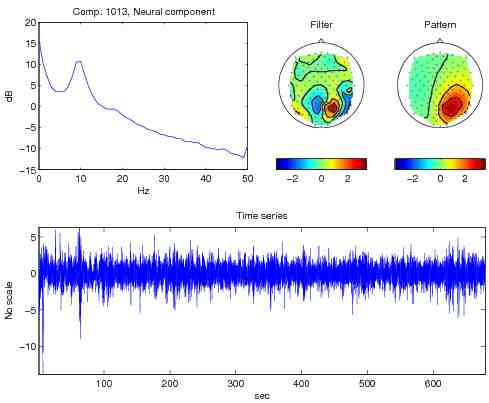

Supplement: Additional file 3 — TestComponents. Visualization of the 1080 independent components in the RT test data, together with the expert's labels. [file 1744-9081-7-30-S3.GZ › components_test/comp1013.jpg]

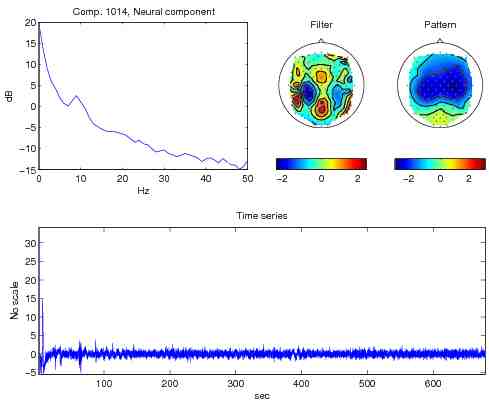

Supplement: Additional file 3 — TestComponents. Visualization of the 1080 independent components in the RT test data, together with the expert's labels. [file 1744-9081-7-30-S3.GZ › components_test/comp1014.jpg]

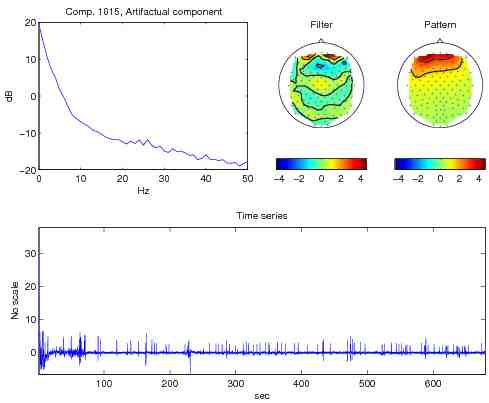

Supplement: Additional file 3 — TestComponents. Visualization of the 1080 independent components in the RT test data, together with the expert's labels. [file 1744-9081-7-30-S3.GZ › components_test/comp1015.jpg]

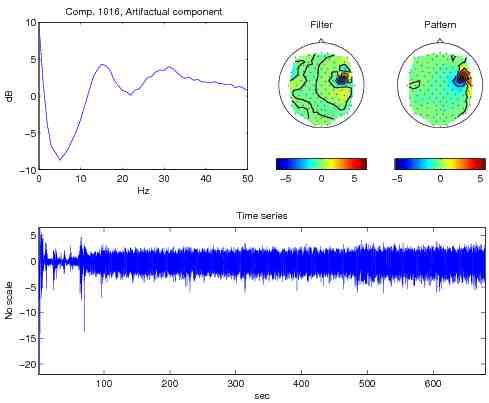

Supplement: Additional file 3 — TestComponents. Visualization of the 1080 independent components in the RT test data, together with the expert's labels. [file 1744-9081-7-30-S3.GZ › components_test/comp1016.jpg]

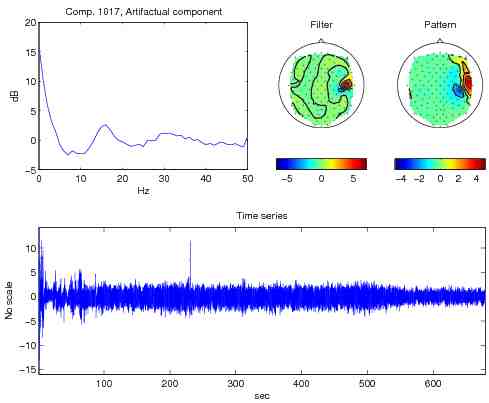

Supplement: Additional file 3 — TestComponents. Visualization of the 1080 independent components in the RT test data, together with the expert's labels. [file 1744-9081-7-30-S3.GZ › components_test/comp1017.jpg]

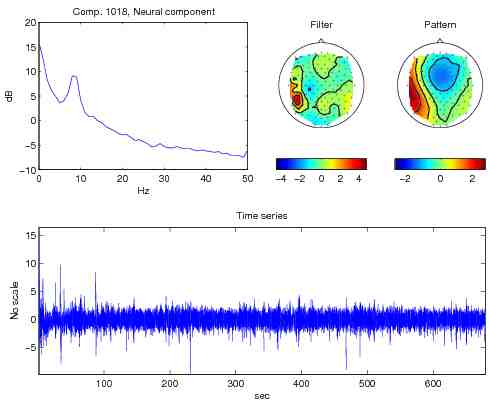

Supplement: Additional file 3 — TestComponents. Visualization of the 1080 independent components in the RT test data, together with the expert's labels. [file 1744-9081-7-30-S3.GZ › components_test/comp1018.jpg]

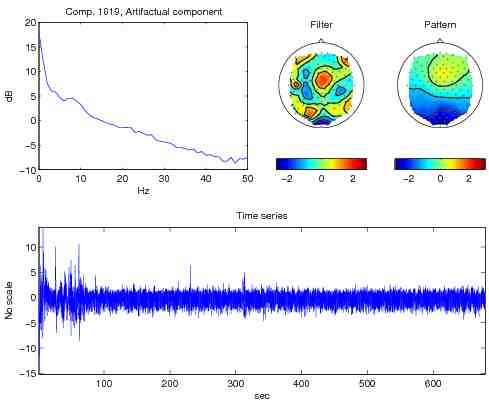

Supplement: Additional file 3 — TestComponents. Visualization of the 1080 independent components in the RT test data, together with the expert's labels. [file 1744-9081-7-30-S3.GZ › components_test/comp1019.jpg]

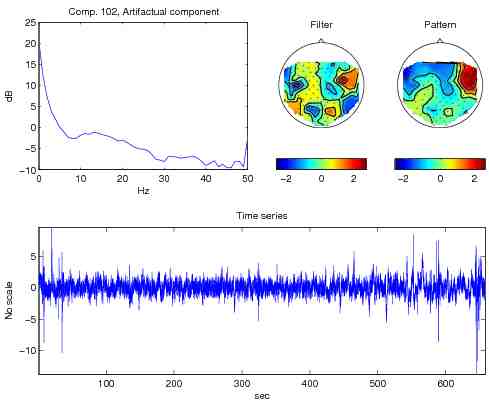

Supplement: Additional file 3 — TestComponents. Visualization of the 1080 independent components in the RT test data, together with the expert's labels. [file 1744-9081-7-30-S3.GZ › components_test/comp102.jpg]

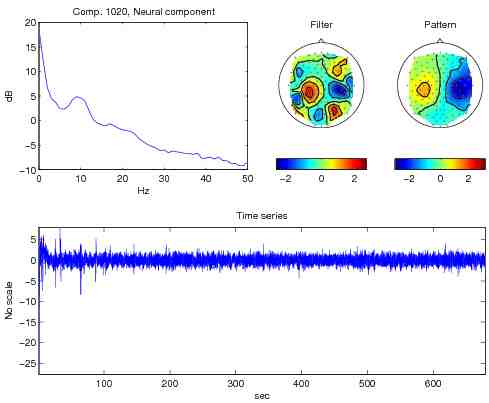

Supplement: Additional file 3 — TestComponents. Visualization of the 1080 independent components in the RT test data, together with the expert's labels. [file 1744-9081-7-30-S3.GZ › components_test/comp1020.jpg]

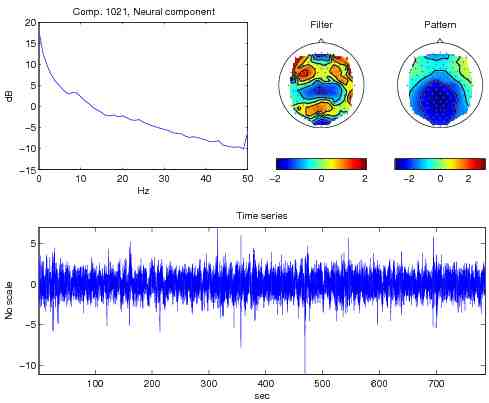

Supplement: Additional file 3 — TestComponents. Visualization of the 1080 independent components in the RT test data, together with the expert's labels. [file 1744-9081-7-30-S3.GZ › components_test/comp1021.jpg]

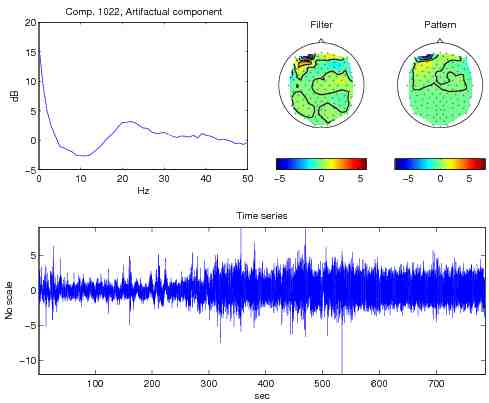

Supplement: Additional file 3 — TestComponents. Visualization of the 1080 independent components in the RT test data, together with the expert's labels. [file 1744-9081-7-30-S3.GZ › components_test/comp1022.jpg]

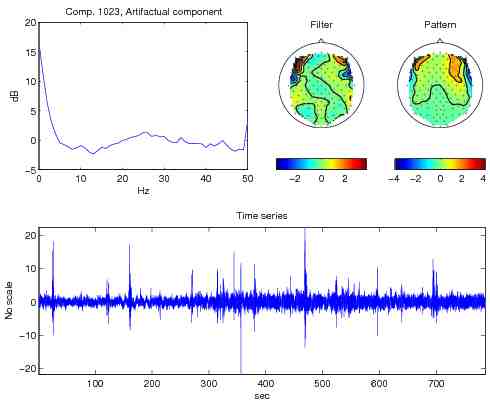

Supplement: Additional file 3 — TestComponents. Visualization of the 1080 independent components in the RT test data, together with the expert's labels. [file 1744-9081-7-30-S3.GZ › components_test/comp1023.jpg]

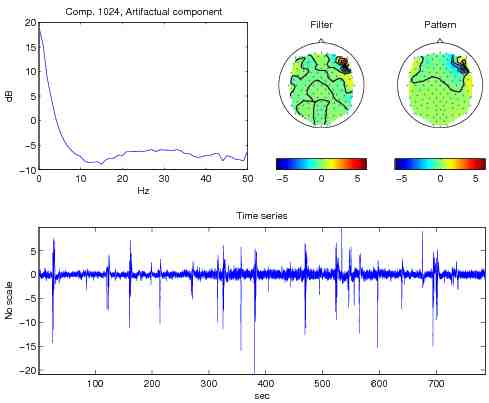

Supplement: Additional file 3 — TestComponents. Visualization of the 1080 independent components in the RT test data, together with the expert's labels. [file 1744-9081-7-30-S3.GZ › components_test/comp1024.jpg]

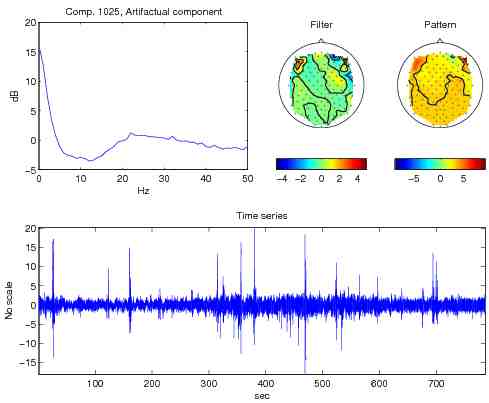

Supplement: Additional file 3 — TestComponents. Visualization of the 1080 independent components in the RT test data, together with the expert's labels. [file 1744-9081-7-30-S3.GZ › components_test/comp1025.jpg]

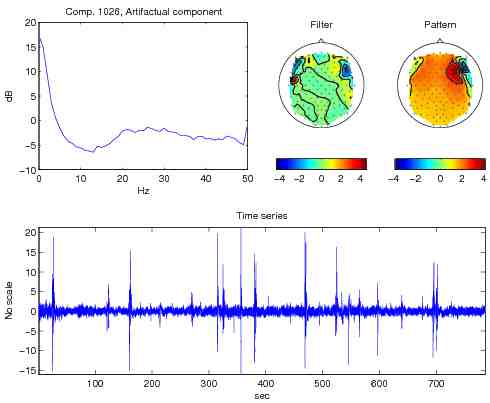

Supplement: Additional file 3 — TestComponents. Visualization of the 1080 independent components in the RT test data, together with the expert's labels. [file 1744-9081-7-30-S3.GZ › components_test/comp1026.jpg]

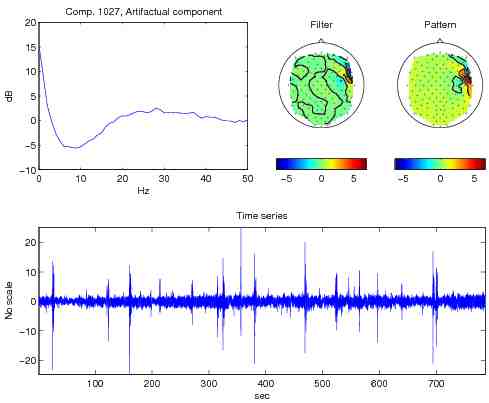

Supplement: Additional file 3 — TestComponents. Visualization of the 1080 independent components in the RT test data, together with the expert's labels. [file 1744-9081-7-30-S3.GZ › components_test/comp1027.jpg]

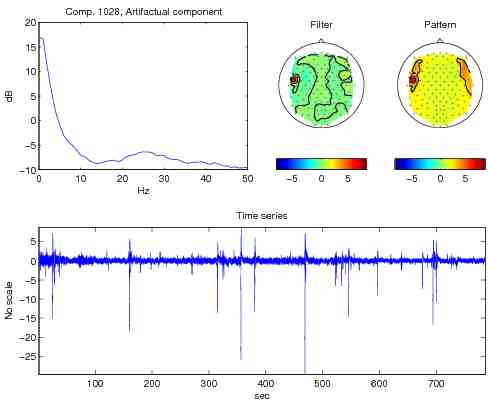

Supplement: Additional file 3 — TestComponents. Visualization of the 1080 independent components in the RT test data, together with the expert's labels. [file 1744-9081-7-30-S3.GZ › components_test/comp1028.jpg]

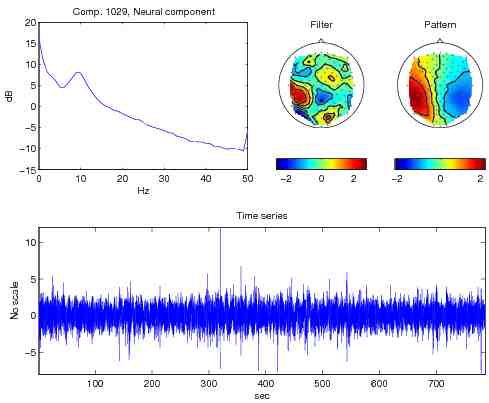

Supplement: Additional file 3 — TestComponents. Visualization of the 1080 independent components in the RT test data, together with the expert's labels. [file 1744-9081-7-30-S3.GZ › components_test/comp1029.jpg]

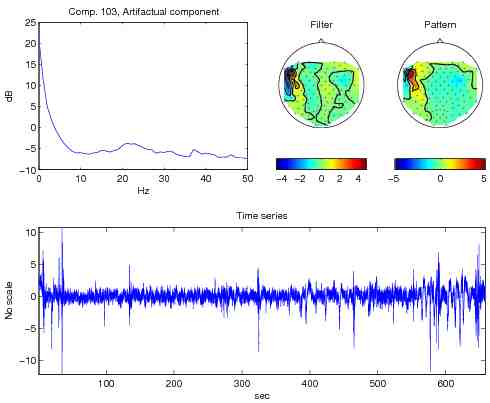

Supplement: Additional file 3 — TestComponents. Visualization of the 1080 independent components in the RT test data, together with the expert's labels. [file 1744-9081-7-30-S3.GZ › components_test/comp103.jpg]

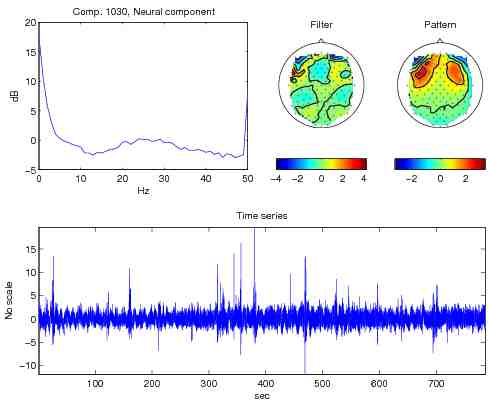

Supplement: Additional file 3 — TestComponents. Visualization of the 1080 independent components in the RT test data, together with the expert's labels. [file 1744-9081-7-30-S3.GZ › components_test/comp1030.jpg]

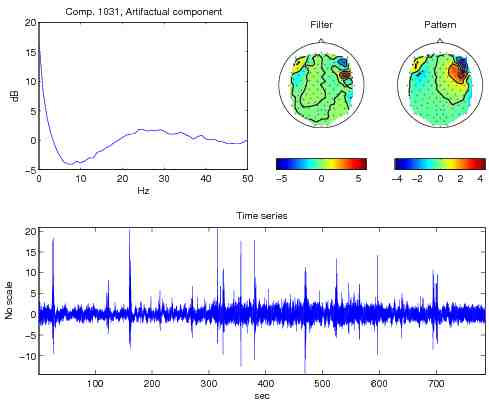

Supplement: Additional file 3 — TestComponents. Visualization of the 1080 independent components in the RT test data, together with the expert's labels. [file 1744-9081-7-30-S3.GZ › components_test/comp1031.jpg]

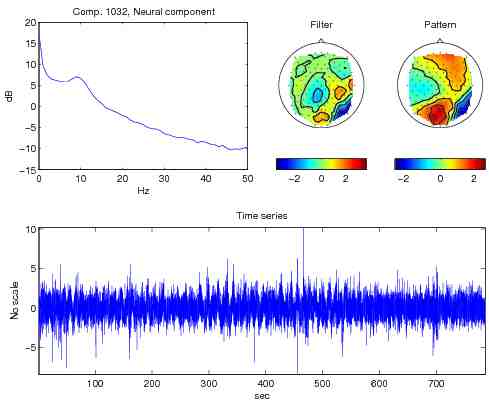

Supplement: Additional file 3 — TestComponents. Visualization of the 1080 independent components in the RT test data, together with the expert's labels. [file 1744-9081-7-30-S3.GZ › components_test/comp1032.jpg]

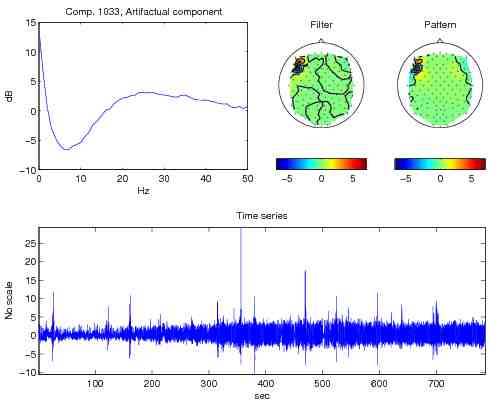

Supplement: Additional file 3 — TestComponents. Visualization of the 1080 independent components in the RT test data, together with the expert's labels. [file 1744-9081-7-30-S3.GZ › components_test/comp1033.jpg]

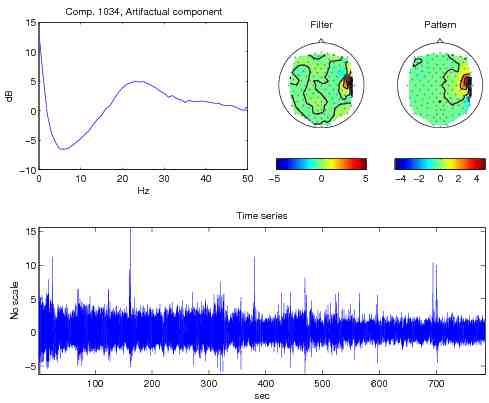

Supplement: Additional file 3 — TestComponents. Visualization of the 1080 independent components in the RT test data, together with the expert's labels. [file 1744-9081-7-30-S3.GZ › components_test/comp1034.jpg]

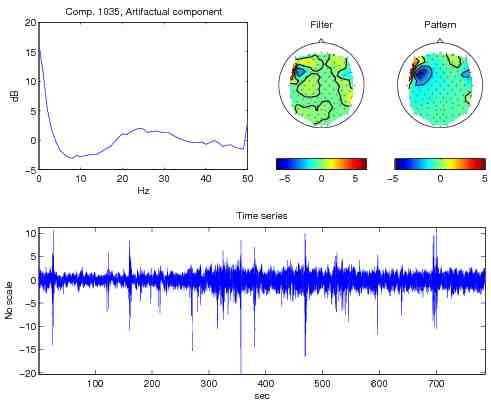

Supplement: Additional file 3 — TestComponents. Visualization of the 1080 independent components in the RT test data, together with the expert's labels. [file 1744-9081-7-30-S3.GZ › components_test/comp1035.jpg]

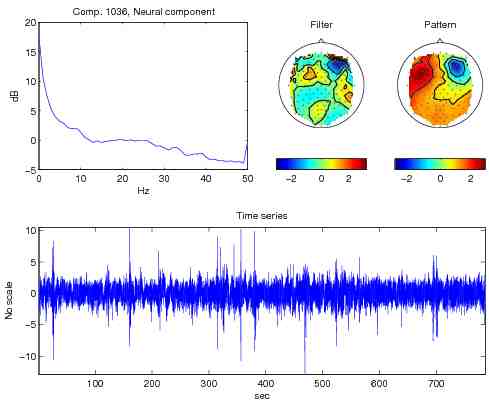

Supplement: Additional file 3 — TestComponents. Visualization of the 1080 independent components in the RT test data, together with the expert's labels. [file 1744-9081-7-30-S3.GZ › components_test/comp1036.jpg]

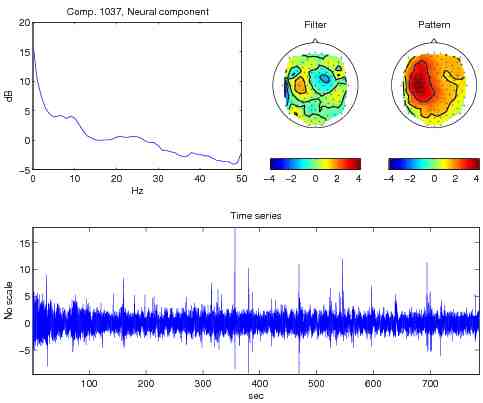

Supplement: Additional file 3 — TestComponents. Visualization of the 1080 independent components in the RT test data, together with the expert's labels. [file 1744-9081-7-30-S3.GZ › components_test/comp1037.jpg]

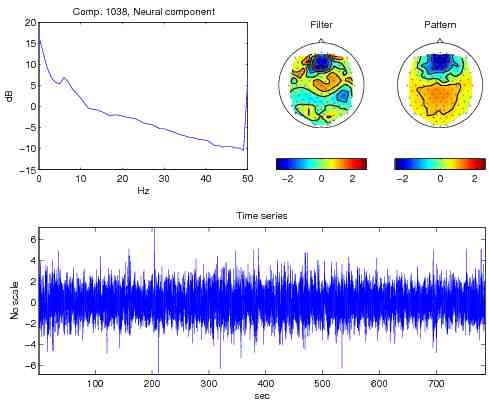

Supplement: Additional file 3 — TestComponents. Visualization of the 1080 independent components in the RT test data, together with the expert's labels. [file 1744-9081-7-30-S3.GZ › components_test/comp1038.jpg]

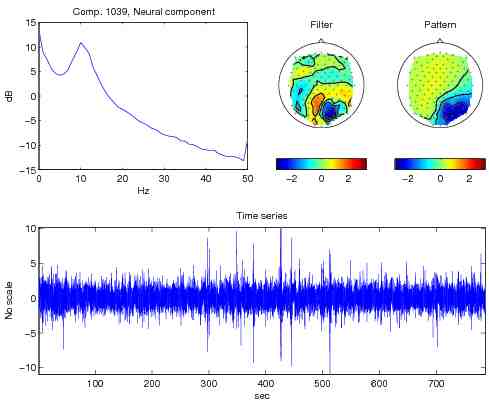

Supplement: Additional file 3 — TestComponents. Visualization of the 1080 independent components in the RT test data, together with the expert's labels. [file 1744-9081-7-30-S3.GZ › components_test/comp1039.jpg]

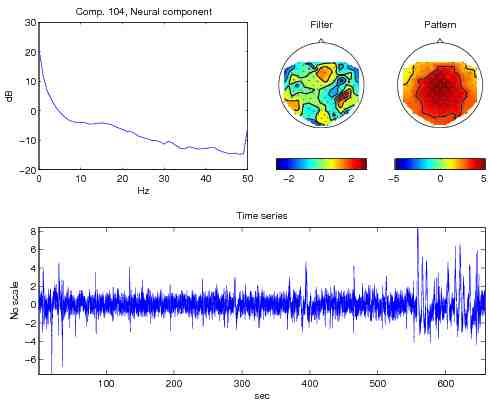

Supplement: Additional file 3 — TestComponents. Visualization of the 1080 independent components in the RT test data, together with the expert's labels. [file 1744-9081-7-30-S3.GZ › components_test/comp104.jpg]

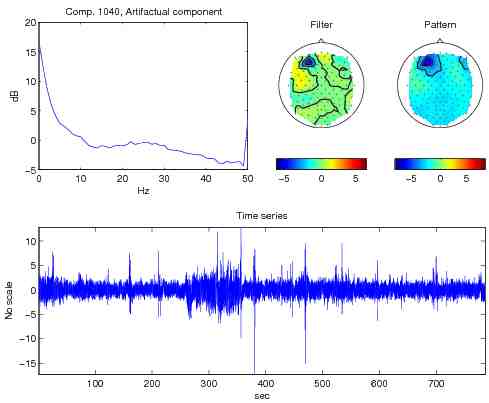

Supplement: Additional file 3 — TestComponents. Visualization of the 1080 independent components in the RT test data, together with the expert's labels. [file 1744-9081-7-30-S3.GZ › components_test/comp1040.jpg]

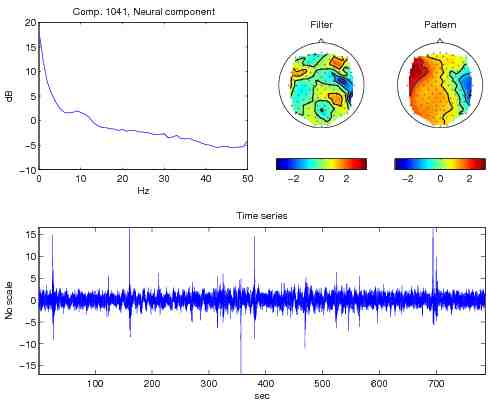

Supplement: Additional file 3 — TestComponents. Visualization of the 1080 independent components in the RT test data, together with the expert's labels. [file 1744-9081-7-30-S3.GZ › components_test/comp1041.jpg]

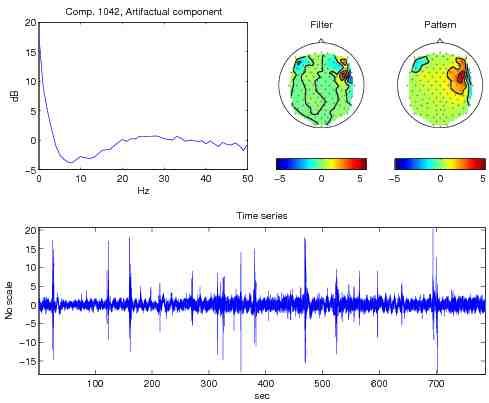

Supplement: Additional file 3 — TestComponents. Visualization of the 1080 independent components in the RT test data, together with the expert's labels. [file 1744-9081-7-30-S3.GZ › components_test/comp1042.jpg]

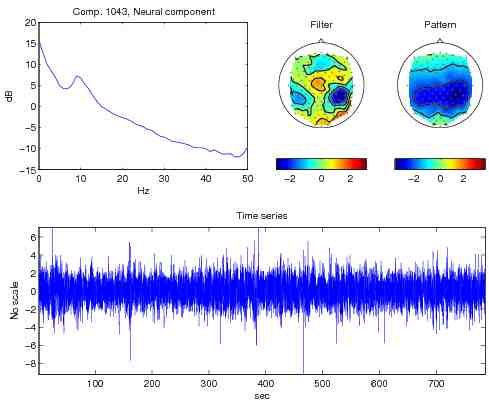

Supplement: Additional file 3 — TestComponents. Visualization of the 1080 independent components in the RT test data, together with the expert's labels. [file 1744-9081-7-30-S3.GZ › components_test/comp1043.jpg]

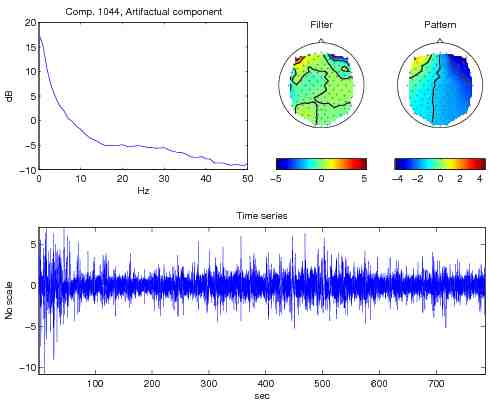

Supplement: Additional file 3 — TestComponents. Visualization of the 1080 independent components in the RT test data, together with the expert's labels. [file 1744-9081-7-30-S3.GZ › components_test/comp1044.jpg]

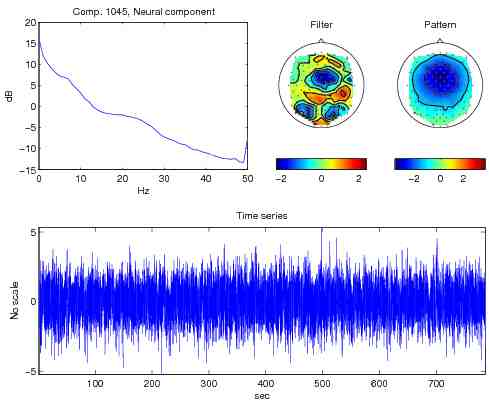

Supplement: Additional file 3 — TestComponents. Visualization of the 1080 independent components in the RT test data, together with the expert's labels. [file 1744-9081-7-30-S3.GZ › components_test/comp1045.jpg]

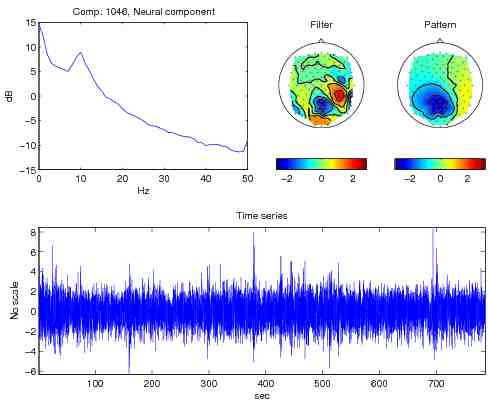

Supplement: Additional file 3 — TestComponents. Visualization of the 1080 independent components in the RT test data, together with the expert's labels. [file 1744-9081-7-30-S3.GZ › components_test/comp1046.jpg]

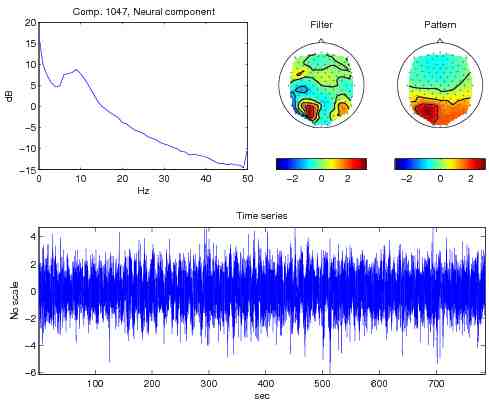

Supplement: Additional file 3 — TestComponents. Visualization of the 1080 independent components in the RT test data, together with the expert's labels. [file 1744-9081-7-30-S3.GZ › components_test/comp1047.jpg]

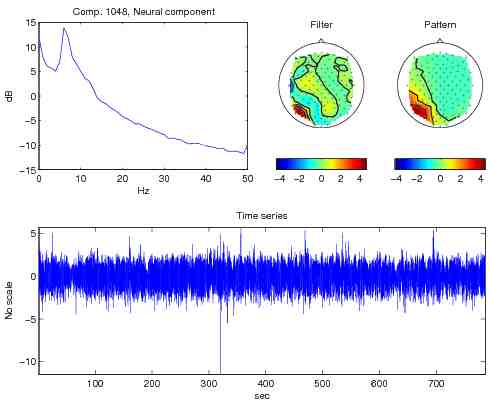

Supplement: Additional file 3 — TestComponents. Visualization of the 1080 independent components in the RT test data, together with the expert's labels. [file 1744-9081-7-30-S3.GZ › components_test/comp1048.jpg]

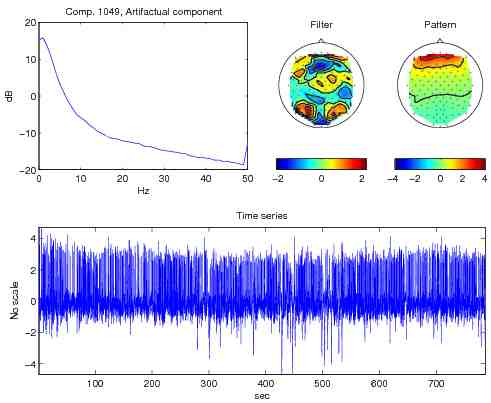

Supplement: Additional file 3 — TestComponents. Visualization of the 1080 independent components in the RT test data, together with the expert's labels. [file 1744-9081-7-30-S3.GZ › components_test/comp1049.jpg]

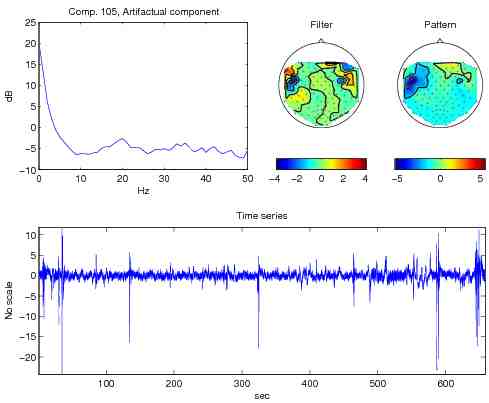

Supplement: Additional file 3 — TestComponents. Visualization of the 1080 independent components in the RT test data, together with the expert's labels. [file 1744-9081-7-30-S3.GZ › components_test/comp105.jpg]

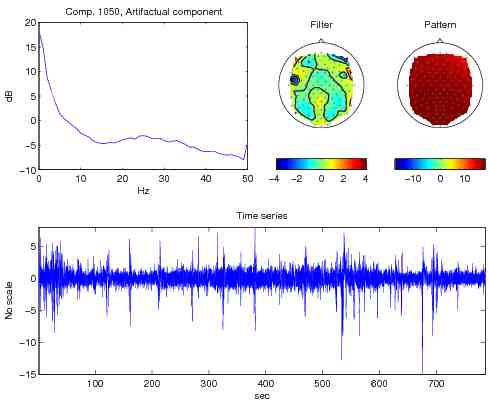

Supplement: Additional file 3 — TestComponents. Visualization of the 1080 independent components in the RT test data, together with the expert's labels. [file 1744-9081-7-30-S3.GZ › components_test/comp1050.jpg]

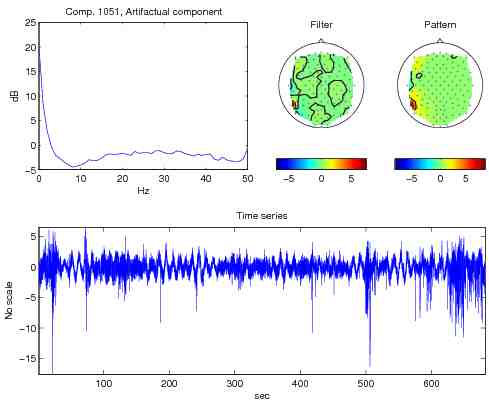

Supplement: Additional file 3 — TestComponents. Visualization of the 1080 independent components in the RT test data, together with the expert's labels. [file 1744-9081-7-30-S3.GZ › components_test/comp1051.jpg]

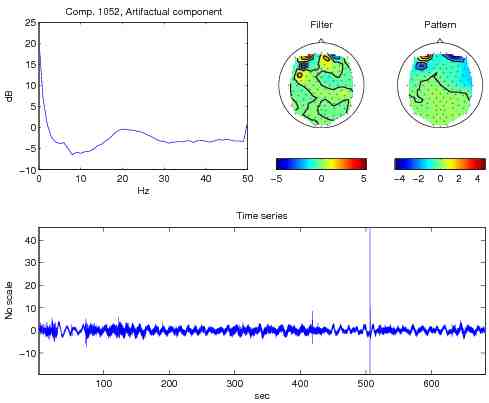

Supplement: Additional file 3 — TestComponents. Visualization of the 1080 independent components in the RT test data, together with the expert's labels. [file 1744-9081-7-30-S3.GZ › components_test/comp1052.jpg]

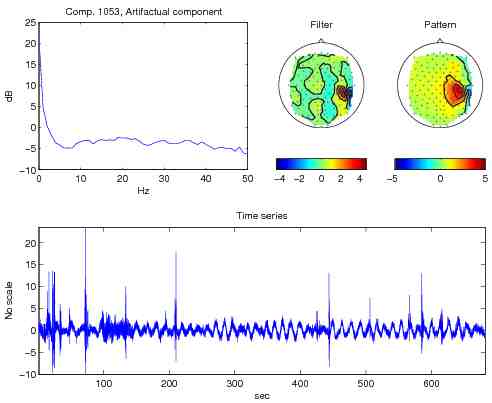

Supplement: Additional file 3 — TestComponents. Visualization of the 1080 independent components in the RT test data, together with the expert's labels. [file 1744-9081-7-30-S3.GZ › components_test/comp1053.jpg]

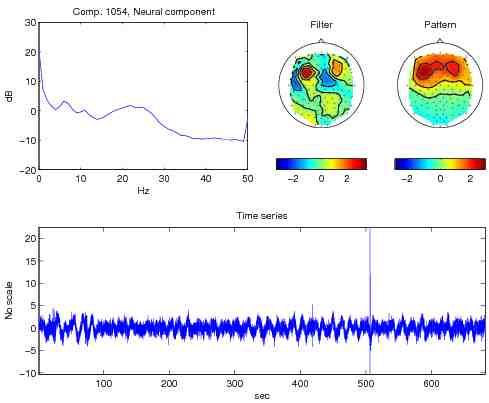

Supplement: Additional file 3 — TestComponents. Visualization of the 1080 independent components in the RT test data, together with the expert's labels. [file 1744-9081-7-30-S3.GZ › components_test/comp1054.jpg]

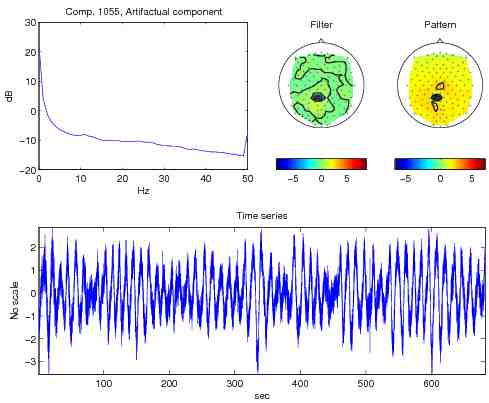

Supplement: Additional file 3 — TestComponents. Visualization of the 1080 independent components in the RT test data, together with the expert's labels. [file 1744-9081-7-30-S3.GZ › components_test/comp1055.jpg]

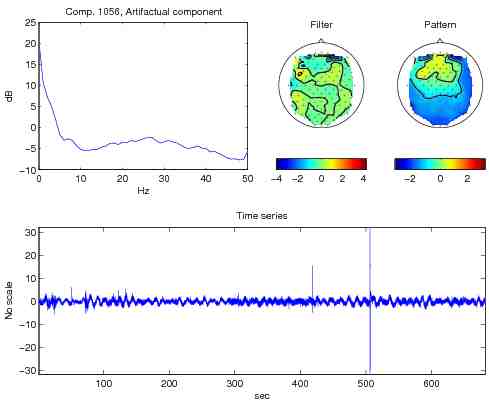

Supplement: Additional file 3 — TestComponents. Visualization of the 1080 independent components in the RT test data, together with the expert's labels. [file 1744-9081-7-30-S3.GZ › components_test/comp1056.jpg]

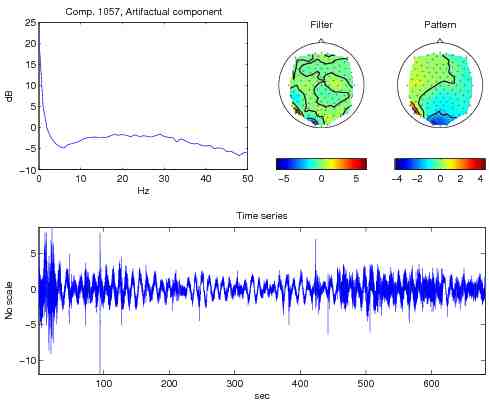

Supplement: Additional file 3 — TestComponents. Visualization of the 1080 independent components in the RT test data, together with the expert's labels. [file 1744-9081-7-30-S3.GZ › components_test/comp1057.jpg]

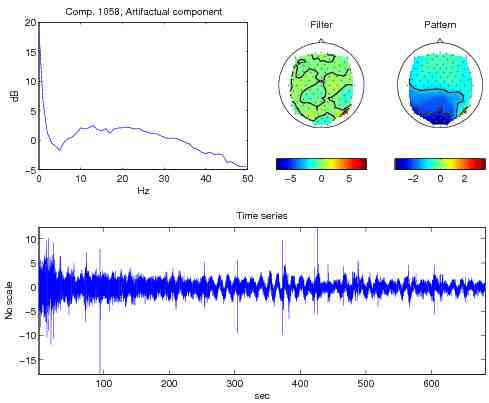

Supplement: Additional file 3 — TestComponents. Visualization of the 1080 independent components in the RT test data, together with the expert's labels. [file 1744-9081-7-30-S3.GZ › components_test/comp1058.jpg]

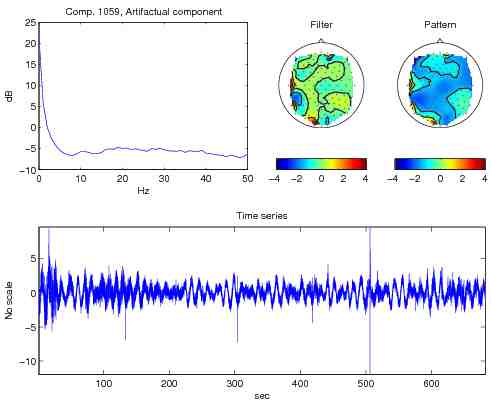

Supplement: Additional file 3 — TestComponents. Visualization of the 1080 independent components in the RT test data, together with the expert's labels. [file 1744-9081-7-30-S3.GZ › components_test/comp1059.jpg]

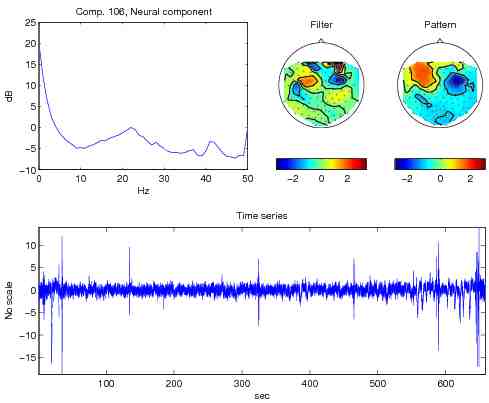

Supplement: Additional file 3 — TestComponents. Visualization of the 1080 independent components in the RT test data, together with the expert's labels. [file 1744-9081-7-30-S3.GZ › components_test/comp106.jpg]

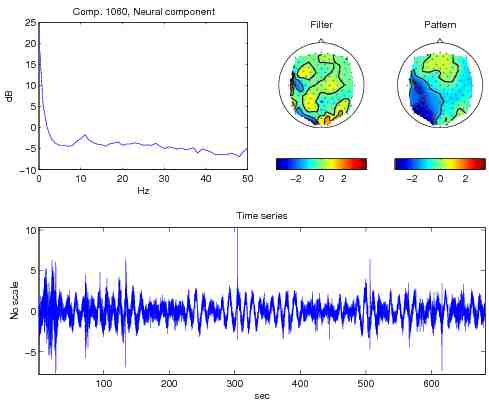

Supplement: Additional file 3 — TestComponents. Visualization of the 1080 independent components in the RT test data, together with the expert's labels. [file 1744-9081-7-30-S3.GZ › components_test/comp1060.jpg]

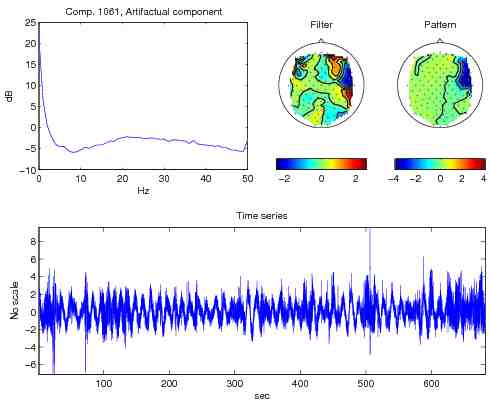

Supplement: Additional file 3 — TestComponents. Visualization of the 1080 independent components in the RT test data, together with the expert's labels. [file 1744-9081-7-30-S3.GZ › components_test/comp1061.jpg]

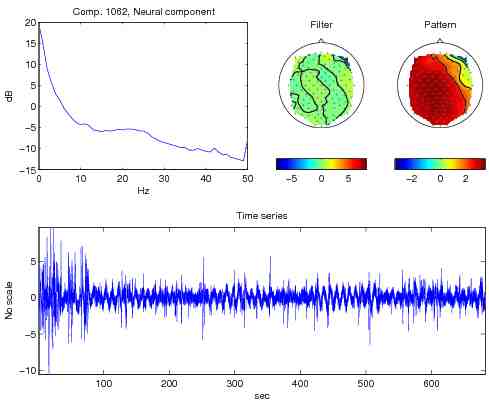

Supplement: Additional file 3 — TestComponents. Visualization of the 1080 independent components in the RT test data, together with the expert's labels. [file 1744-9081-7-30-S3.GZ › components_test/comp1062.jpg]

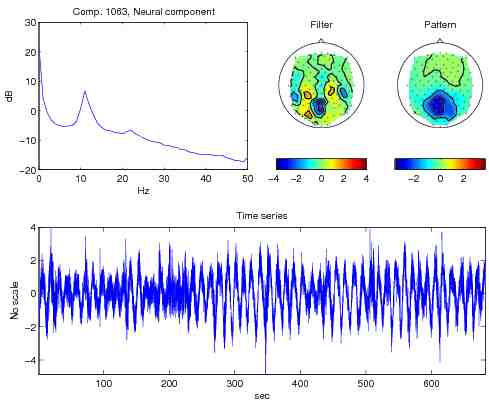

Supplement: Additional file 3 — TestComponents. Visualization of the 1080 independent components in the RT test data, together with the expert's labels. [file 1744-9081-7-30-S3.GZ › components_test/comp1063.jpg]

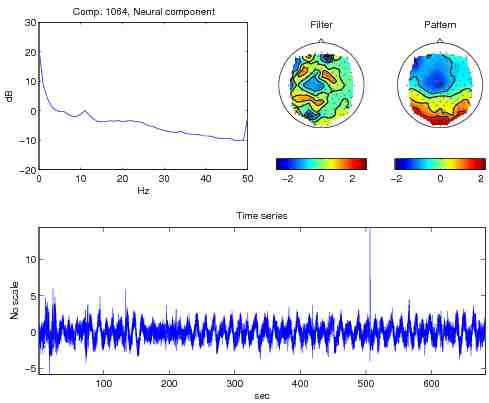

Supplement: Additional file 3 — TestComponents. Visualization of the 1080 independent components in the RT test data, together with the expert's labels. [file 1744-9081-7-30-S3.GZ › components_test/comp1064.jpg]

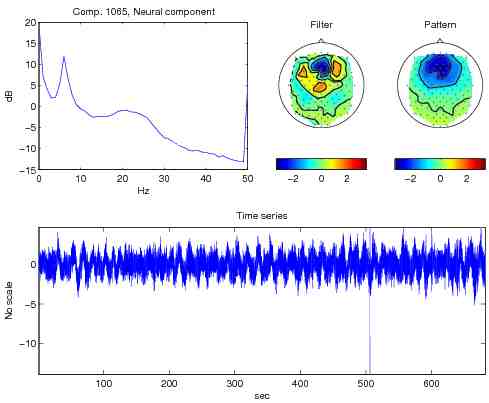

Supplement: Additional file 3 — TestComponents. Visualization of the 1080 independent components in the RT test data, together with the expert's labels. [file 1744-9081-7-30-S3.GZ › components_test/comp1065.jpg]

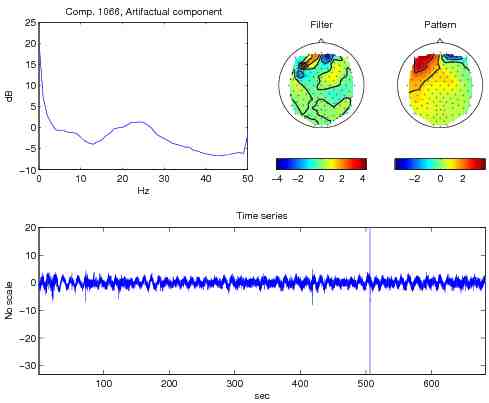

Supplement: Additional file 3 — TestComponents. Visualization of the 1080 independent components in the RT test data, together with the expert's labels. [file 1744-9081-7-30-S3.GZ › components_test/comp1066.jpg]

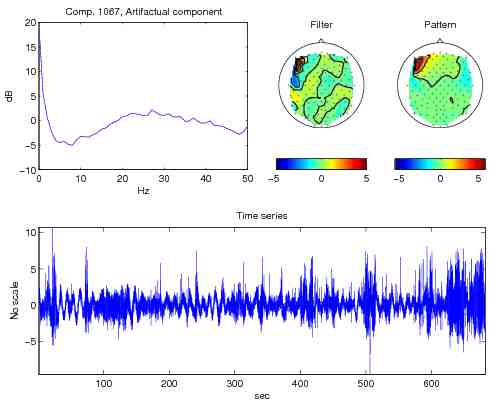

Supplement: Additional file 3 — TestComponents. Visualization of the 1080 independent components in the RT test data, together with the expert's labels. [file 1744-9081-7-30-S3.GZ › components_test/comp1067.jpg]

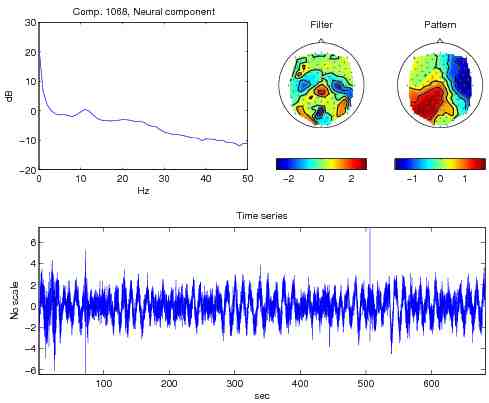

Supplement: Additional file 3 — TestComponents. Visualization of the 1080 independent components in the RT test data, together with the expert's labels. [file 1744-9081-7-30-S3.GZ › components_test/comp1068.jpg]

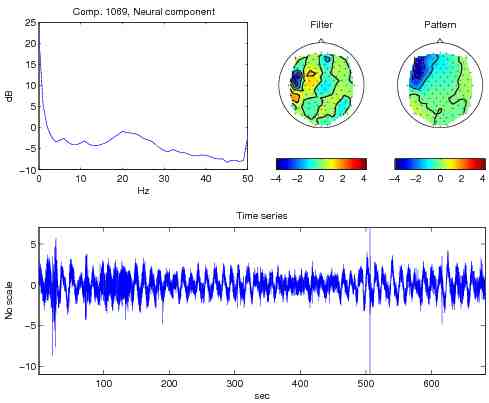

Supplement: Additional file 3 — TestComponents. Visualization of the 1080 independent components in the RT test data, together with the expert's labels. [file 1744-9081-7-30-S3.GZ › components_test/comp1069.jpg]

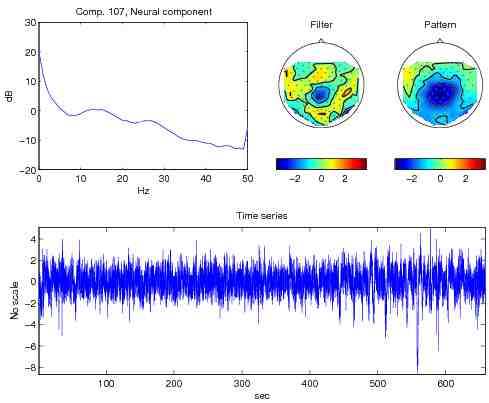

Supplement: Additional file 3 — TestComponents. Visualization of the 1080 independent components in the RT test data, together with the expert's labels. [file 1744-9081-7-30-S3.GZ › components_test/comp107.jpg]

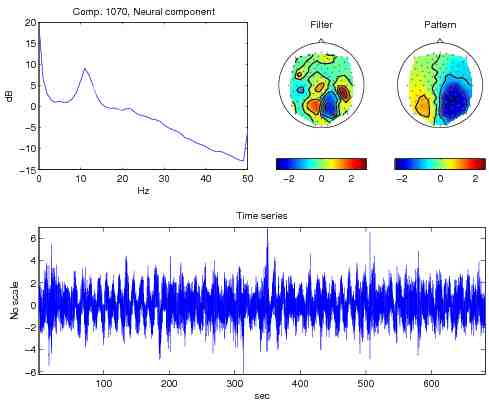

Supplement: Additional file 3 — TestComponents. Visualization of the 1080 independent components in the RT test data, together with the expert's labels. [file 1744-9081-7-30-S3.GZ › components_test/comp1070.jpg]

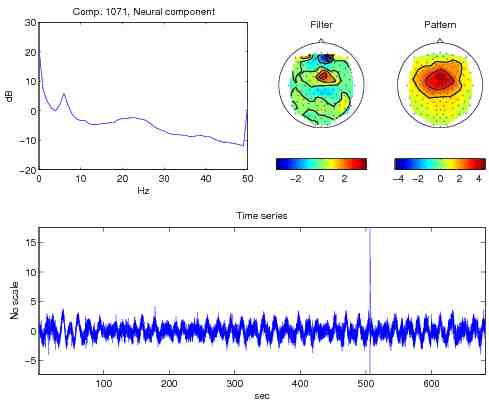

Supplement: Additional file 3 — TestComponents. Visualization of the 1080 independent components in the RT test data, together with the expert's labels. [file 1744-9081-7-30-S3.GZ › components_test/comp1071.jpg]

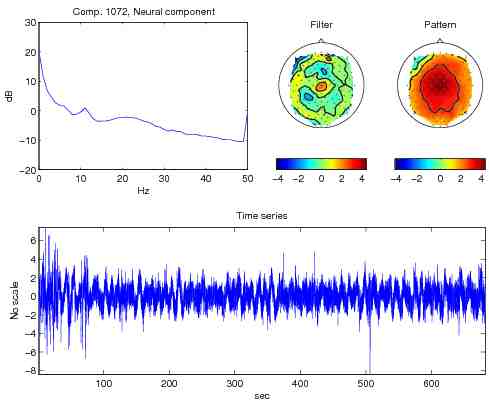

Supplement: Additional file 3 — TestComponents. Visualization of the 1080 independent components in the RT test data, together with the expert's labels. [file 1744-9081-7-30-S3.GZ › components_test/comp1072.jpg]

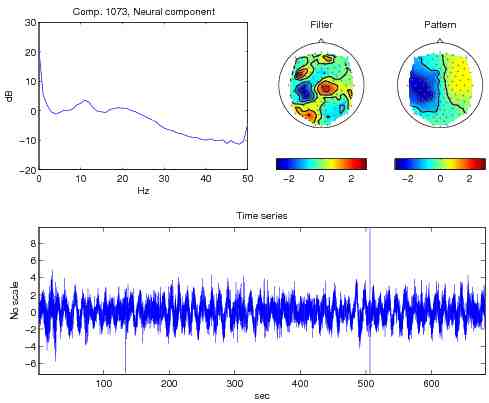

Supplement: Additional file 3 — TestComponents. Visualization of the 1080 independent components in the RT test data, together with the expert's labels. [file 1744-9081-7-30-S3.GZ › components_test/comp1073.jpg]

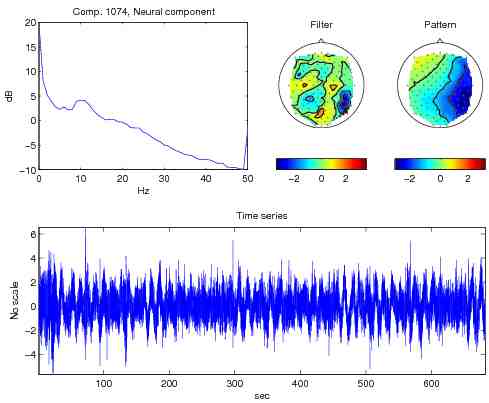

Supplement: Additional file 3 — TestComponents. Visualization of the 1080 independent components in the RT test data, together with the expert's labels. [file 1744-9081-7-30-S3.GZ › components_test/comp1074.jpg]

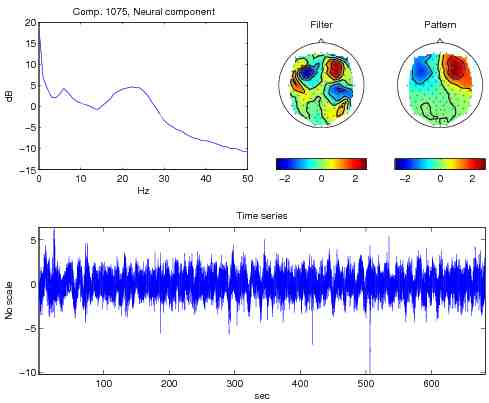

Supplement: Additional file 3 — TestComponents. Visualization of the 1080 independent components in the RT test data, together with the expert's labels. [file 1744-9081-7-30-S3.GZ › components_test/comp1075.jpg]

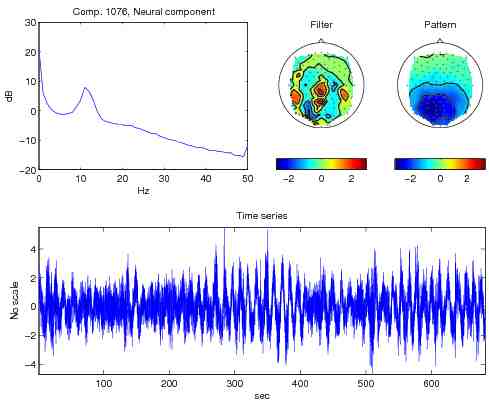

Supplement: Additional file 3 — TestComponents. Visualization of the 1080 independent components in the RT test data, together with the expert's labels. [file 1744-9081-7-30-S3.GZ › components_test/comp1076.jpg]

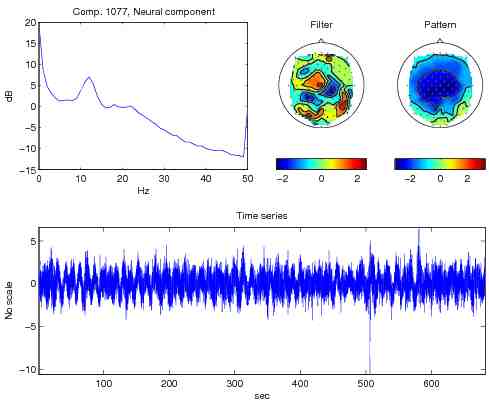

Supplement: Additional file 3 — TestComponents. Visualization of the 1080 independent components in the RT test data, together with the expert's labels. [file 1744-9081-7-30-S3.GZ › components_test/comp1077.jpg]

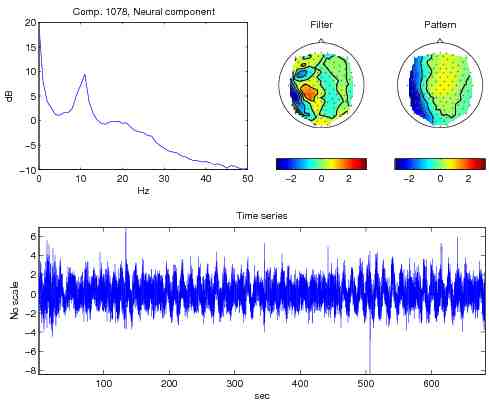

Supplement: Additional file 3 — TestComponents. Visualization of the 1080 independent components in the RT test data, together with the expert's labels. [file 1744-9081-7-30-S3.GZ › components_test/comp1078.jpg]

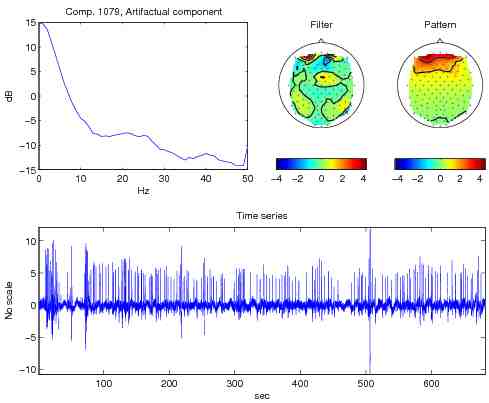

Supplement: Additional file 3 — TestComponents. Visualization of the 1080 independent components in the RT test data, together with the expert's labels. [file 1744-9081-7-30-S3.GZ › components_test/comp1079.jpg]

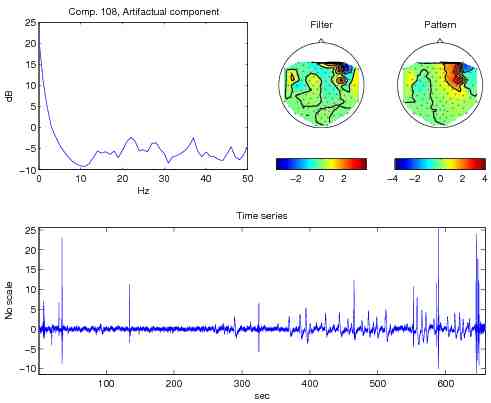

Supplement: Additional file 3 — TestComponents. Visualization of the 1080 independent components in the RT test data, together with the expert's labels. [file 1744-9081-7-30-S3.GZ › components_test/comp108.jpg]

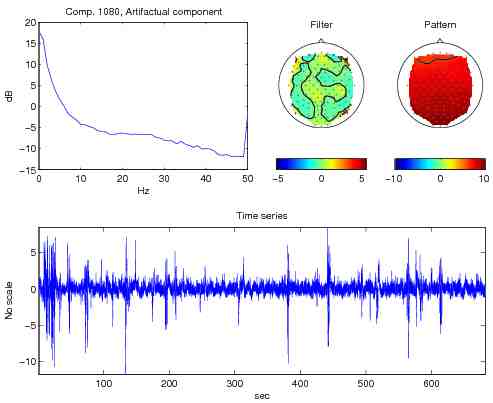

Supplement: Additional file 3 — TestComponents. Visualization of the 1080 independent components in the RT test data, together with the expert's labels. [file 1744-9081-7-30-S3.GZ › components_test/comp1080.jpg]

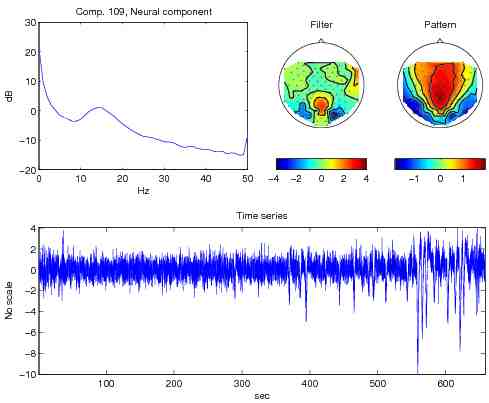

Supplement: Additional file 3 — TestComponents. Visualization of the 1080 independent components in the RT test data, together with the expert's labels. [file 1744-9081-7-30-S3.GZ › components_test/comp109.jpg]

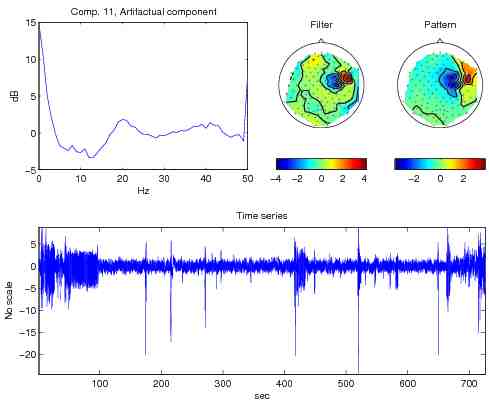

Supplement: Additional file 3 — TestComponents. Visualization of the 1080 independent components in the RT test data, together with the expert's labels. [file 1744-9081-7-30-S3.GZ › components_test/comp11.jpg]

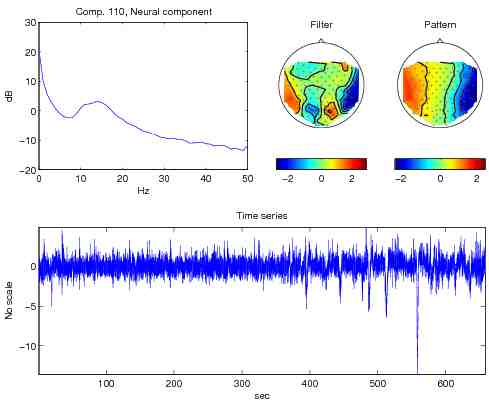

Supplement: Additional file 3 — TestComponents. Visualization of the 1080 independent components in the RT test data, together with the expert's labels. [file 1744-9081-7-30-S3.GZ › components_test/comp110.jpg]

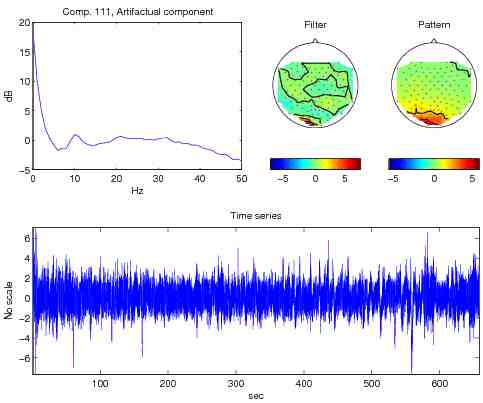

Supplement: Additional file 3 — TestComponents. Visualization of the 1080 independent components in the RT test data, together with the expert's labels. [file 1744-9081-7-30-S3.GZ › components_test/comp111.jpg]

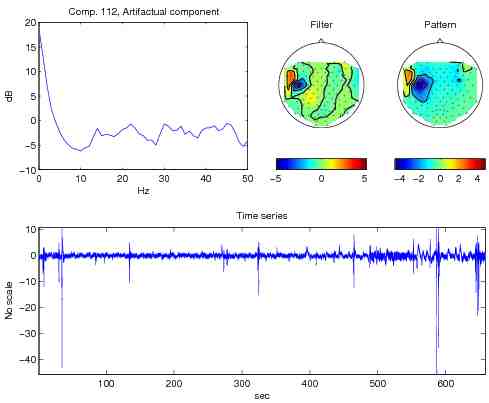

Supplement: Additional file 3 — TestComponents. Visualization of the 1080 independent components in the RT test data, together with the expert's labels. [file 1744-9081-7-30-S3.GZ › components_test/comp112.jpg]

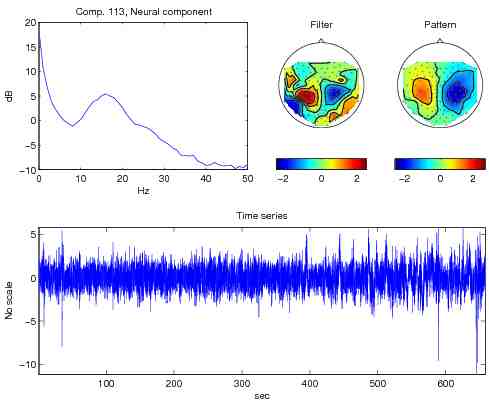

Supplement: Additional file 3 — TestComponents. Visualization of the 1080 independent components in the RT test data, together with the expert's labels. [file 1744-9081-7-30-S3.GZ › components_test/comp113.jpg]

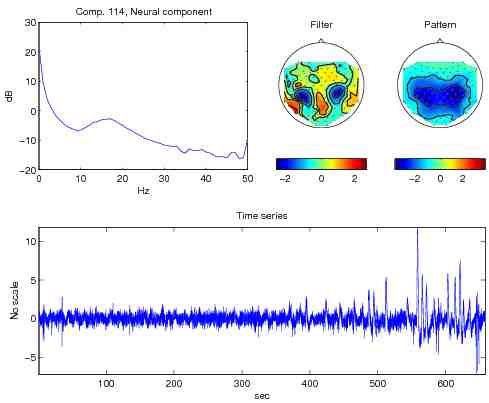

Supplement: Additional file 3 — TestComponents. Visualization of the 1080 independent components in the RT test data, together with the expert's labels. [file 1744-9081-7-30-S3.GZ › components_test/comp114.jpg]

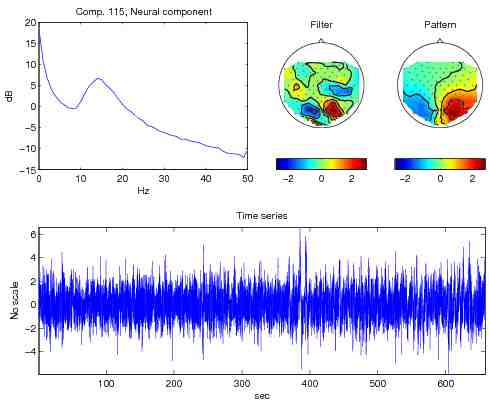

Supplement: Additional file 3 — TestComponents. Visualization of the 1080 independent components in the RT test data, together with the expert's labels. [file 1744-9081-7-30-S3.GZ › components_test/comp115.jpg]
