# Supplementary material for: Automatic Classification of Artifactual ICA-Components for Artifact Removal in EEG Signals
Source: Behav Brain Funct. 2011 Aug 2;7:30. doi: 10.1186/1744-9081-7-30 (PMC3175453; doi:10.1186/1744-9081-7-30)
Supplement: Additional file 4 — Misclassifications. Visualization of the 75 + 21 misclassified components of the RT test data [file 1744-9081-7-30-S4.GZ › misclass/RLDA_says_artifact.pdf]

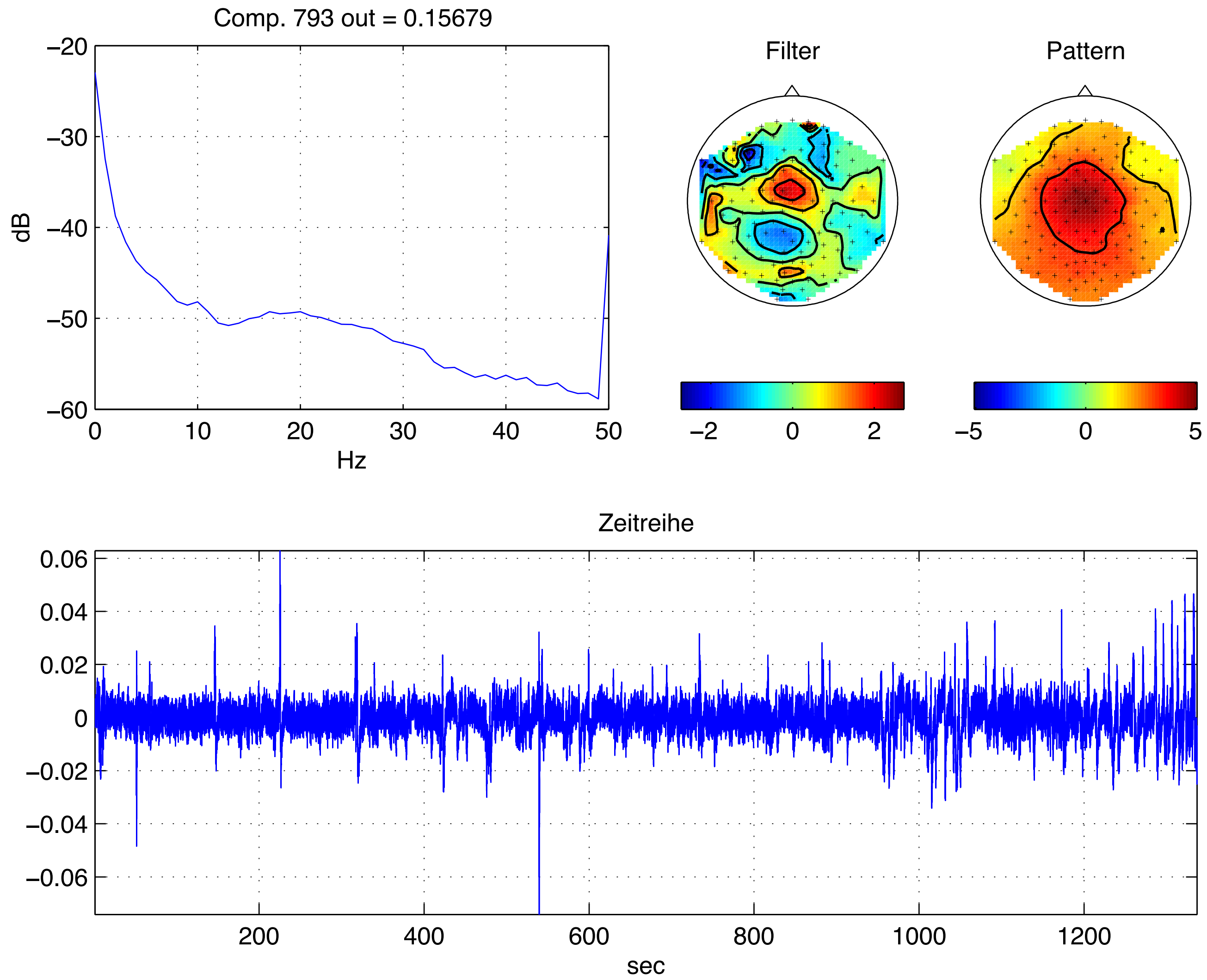
Comp. 793 out = 0.15679
dB
Hz
Filter
Pattern
Zeitreihe
sec

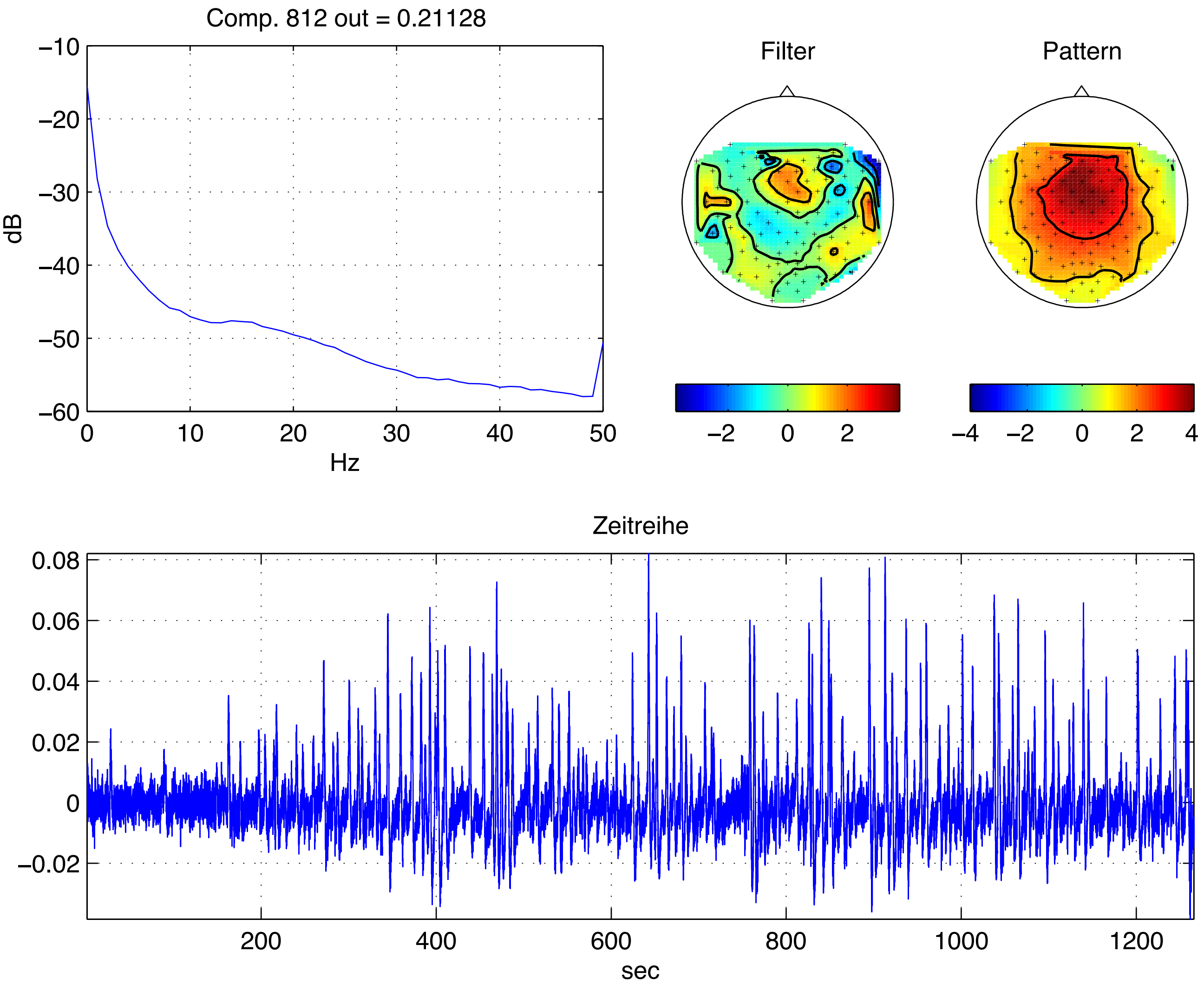

Comp. 812 out = 0.21128
dB
Hz
Filter
Pattern
Zeitreihe
sec

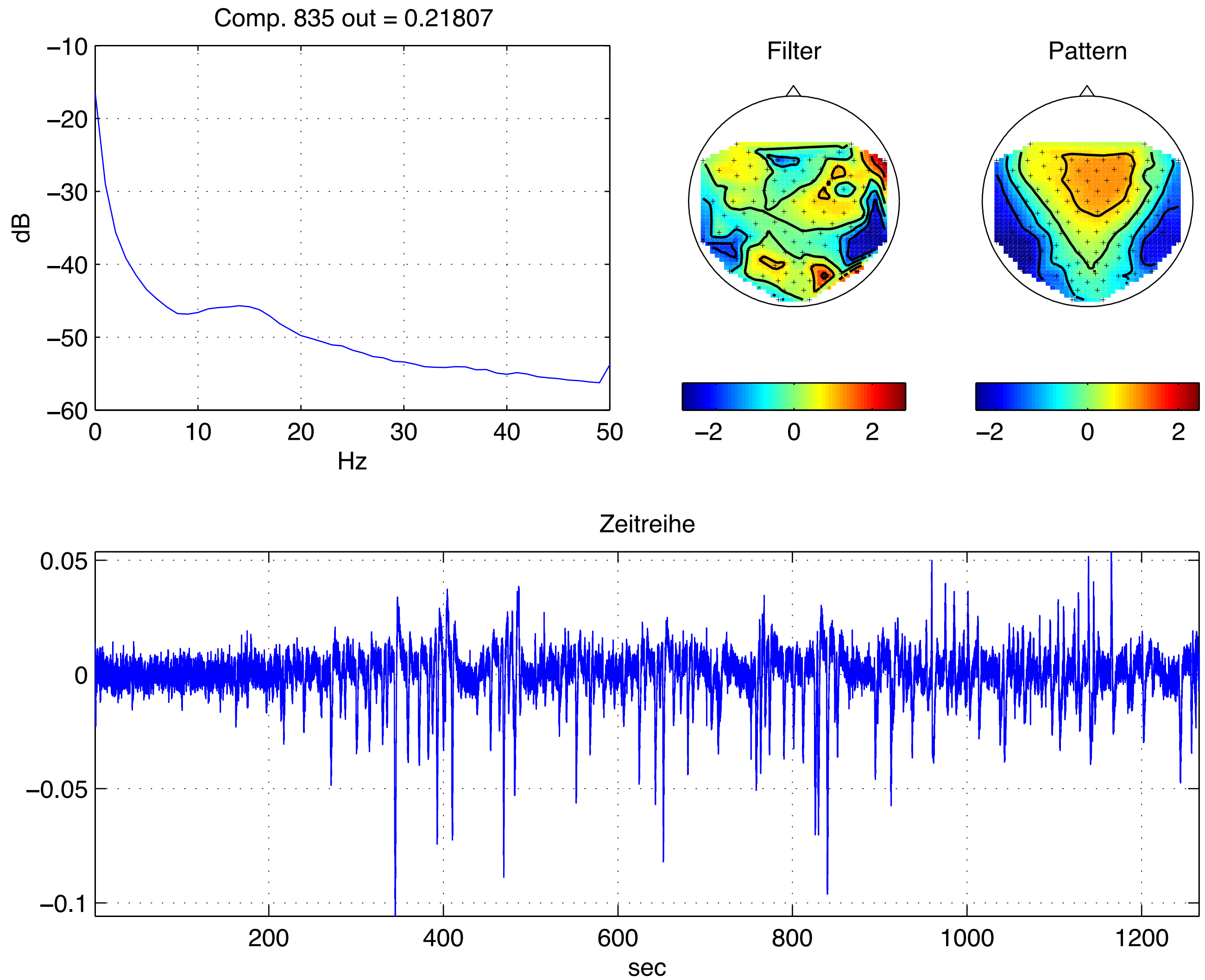

Comp. 835 out = 0.21807
dB
Hz
Filter
Pattern
Zeitreihe
sec

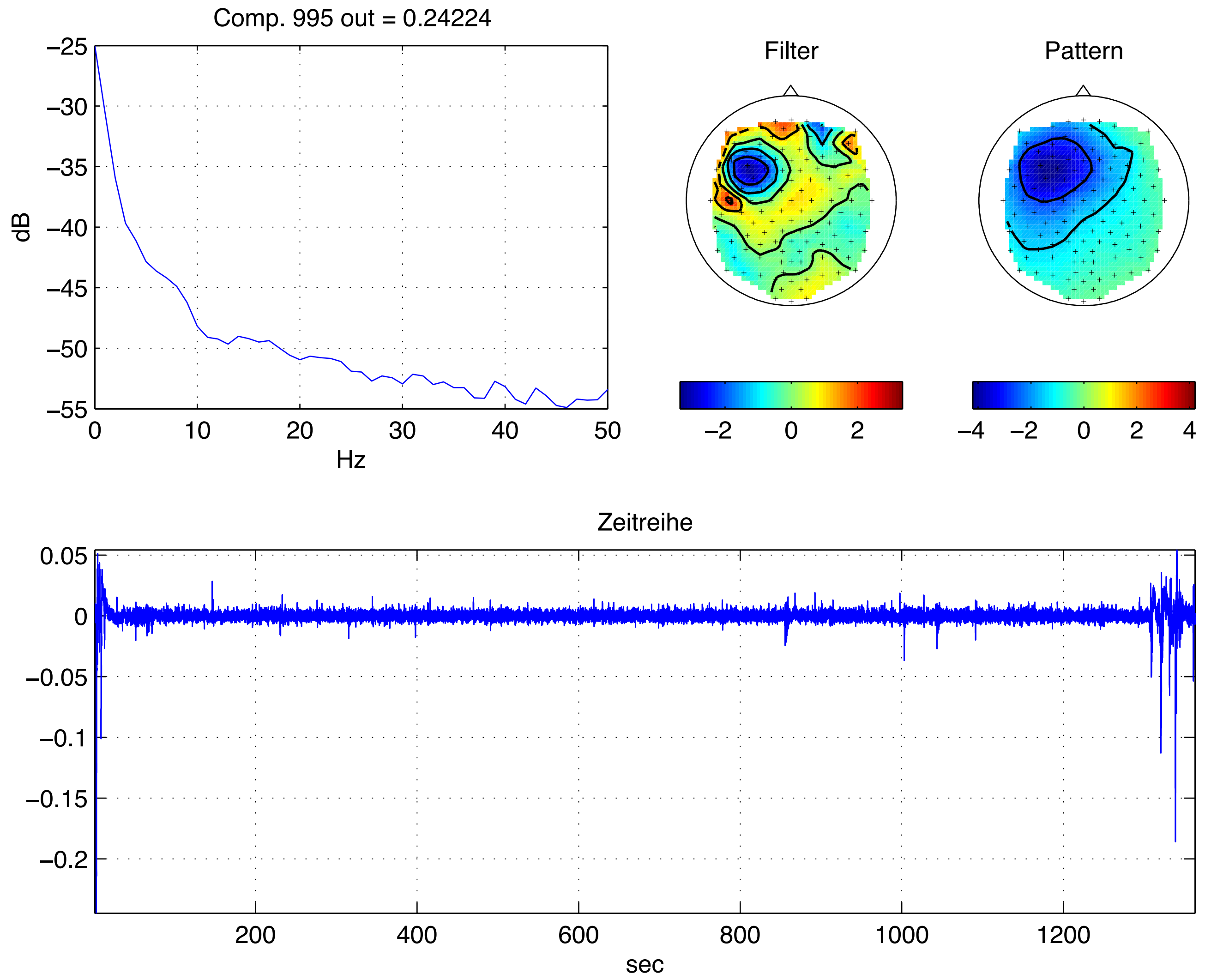
Comp. 995 out = 0.24224
dB
Hz
Filter
Pattern
Zeitreihe
sec

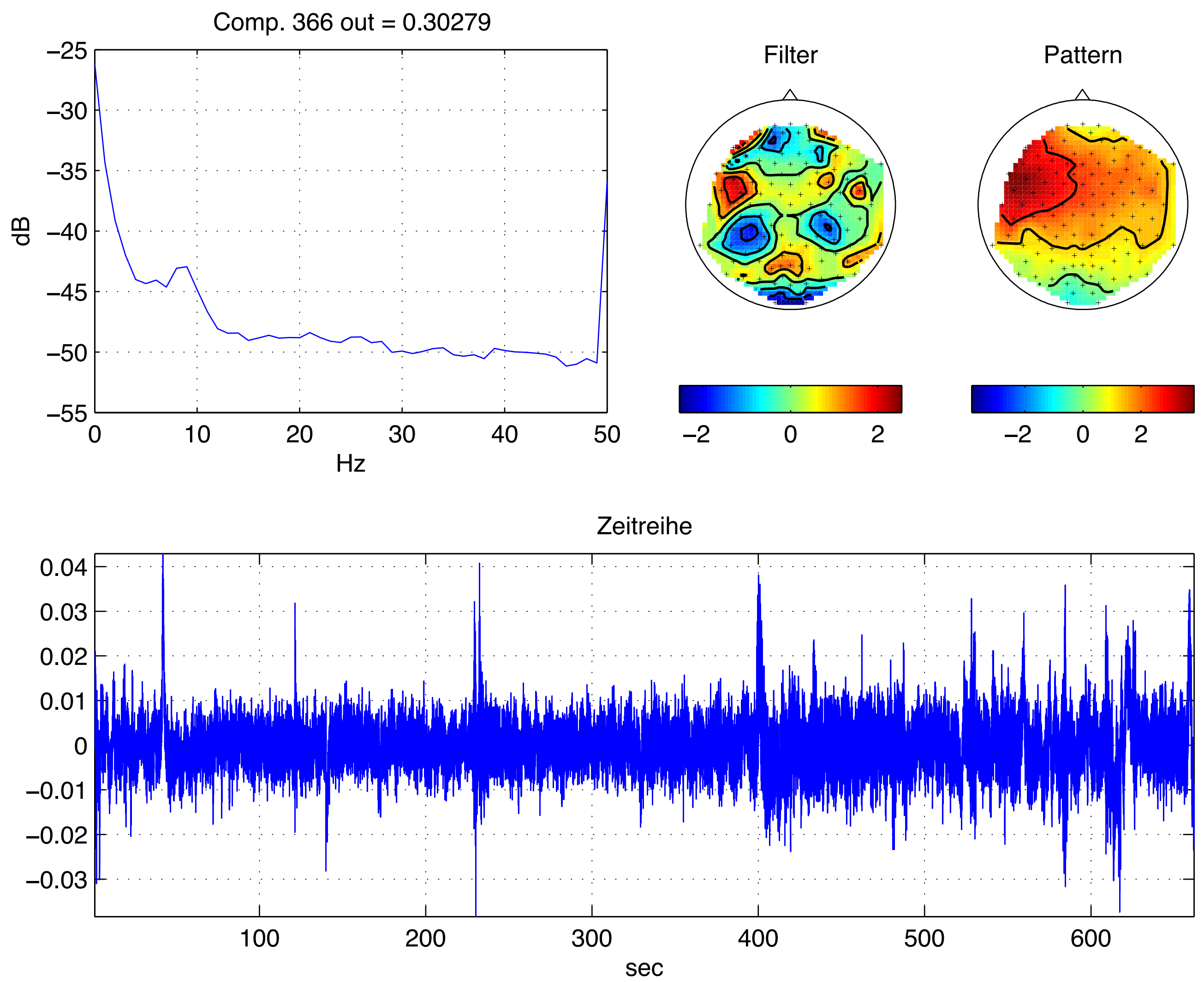
Comp. 366 out = 0.30279
dB
Hz
-25
-30
-35
-40
-45
-50
-55
0
10
20
30
40
50
Filter
Pattern
-2
0
2
Zeitreihe
0.04
0.03
0.02
0.01
-0.01
-0.02
-0.03
100
200
300
400
500
600
sec

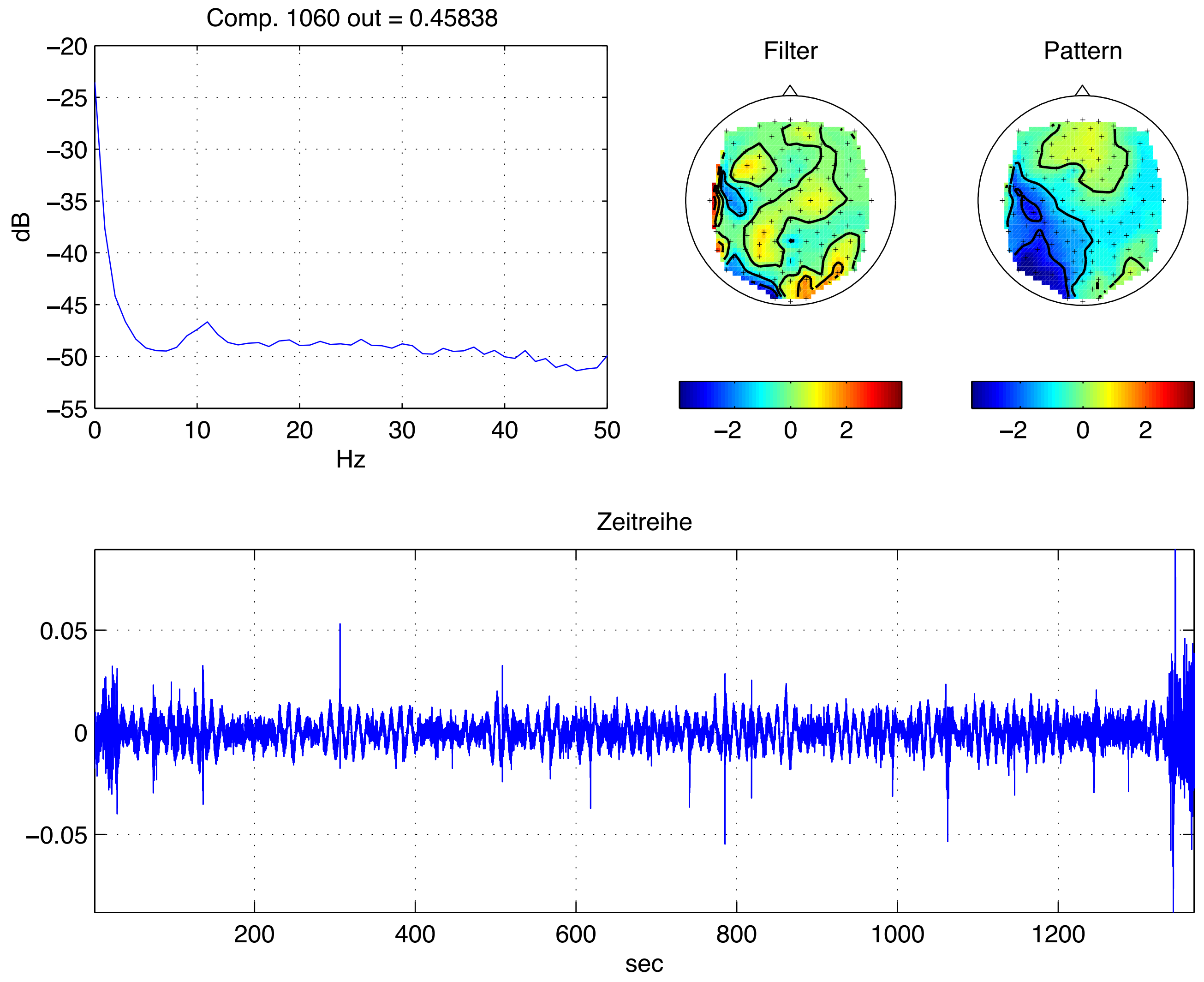

Comp. 1060 out = 0.45838
dB
Hz
−20
−25
−30
−35
−40
−45
−50
−55
0
10
20
30
40
50
Filter
Pattern
−2
0
2
Zeitreihe
0.05
0
−0.05
200
400
600
800
1000
1200
sec

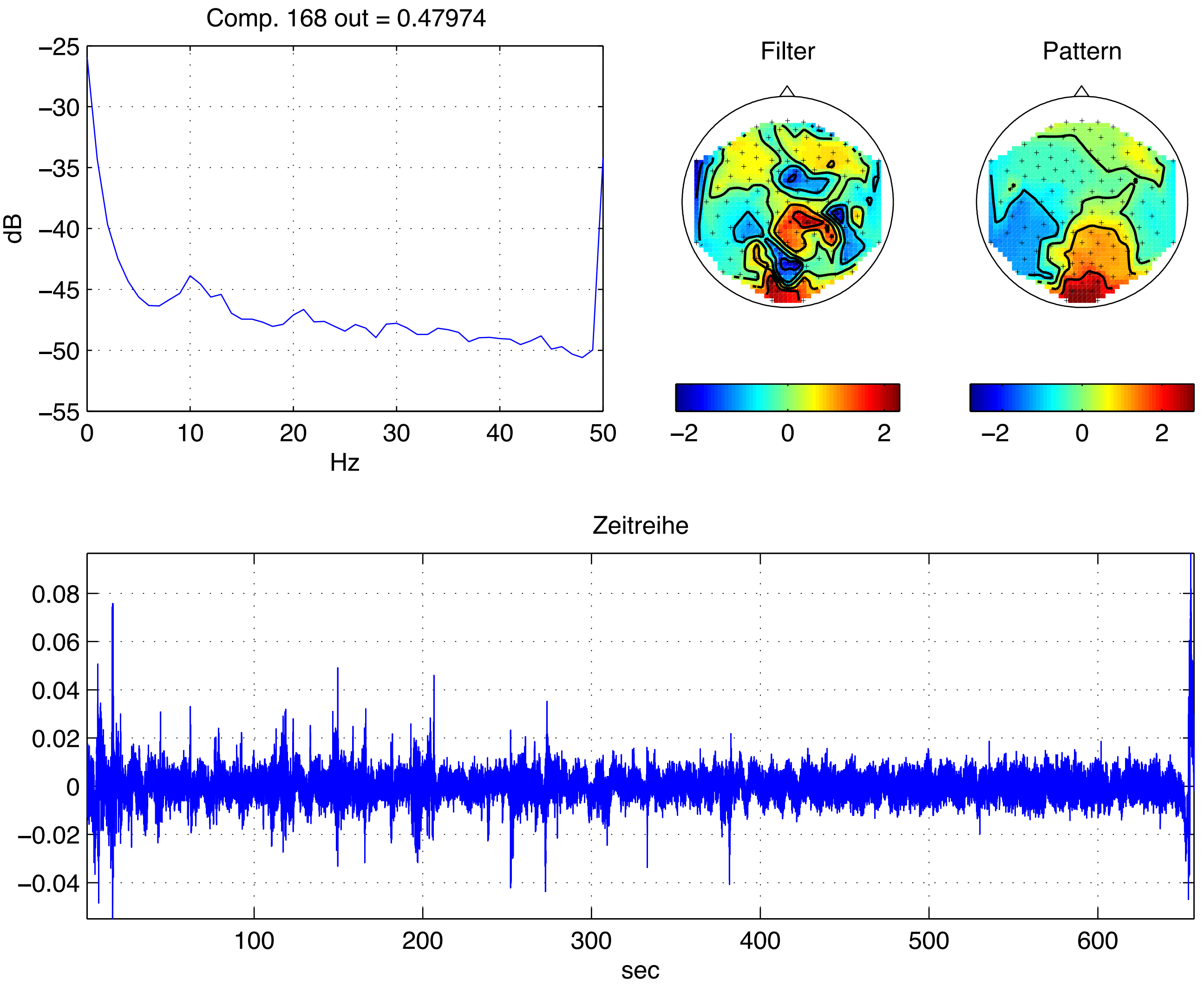

Comp. 168 out = 0.47974
dB
Hz
Filter
Pattern
Zeitreihe
sec

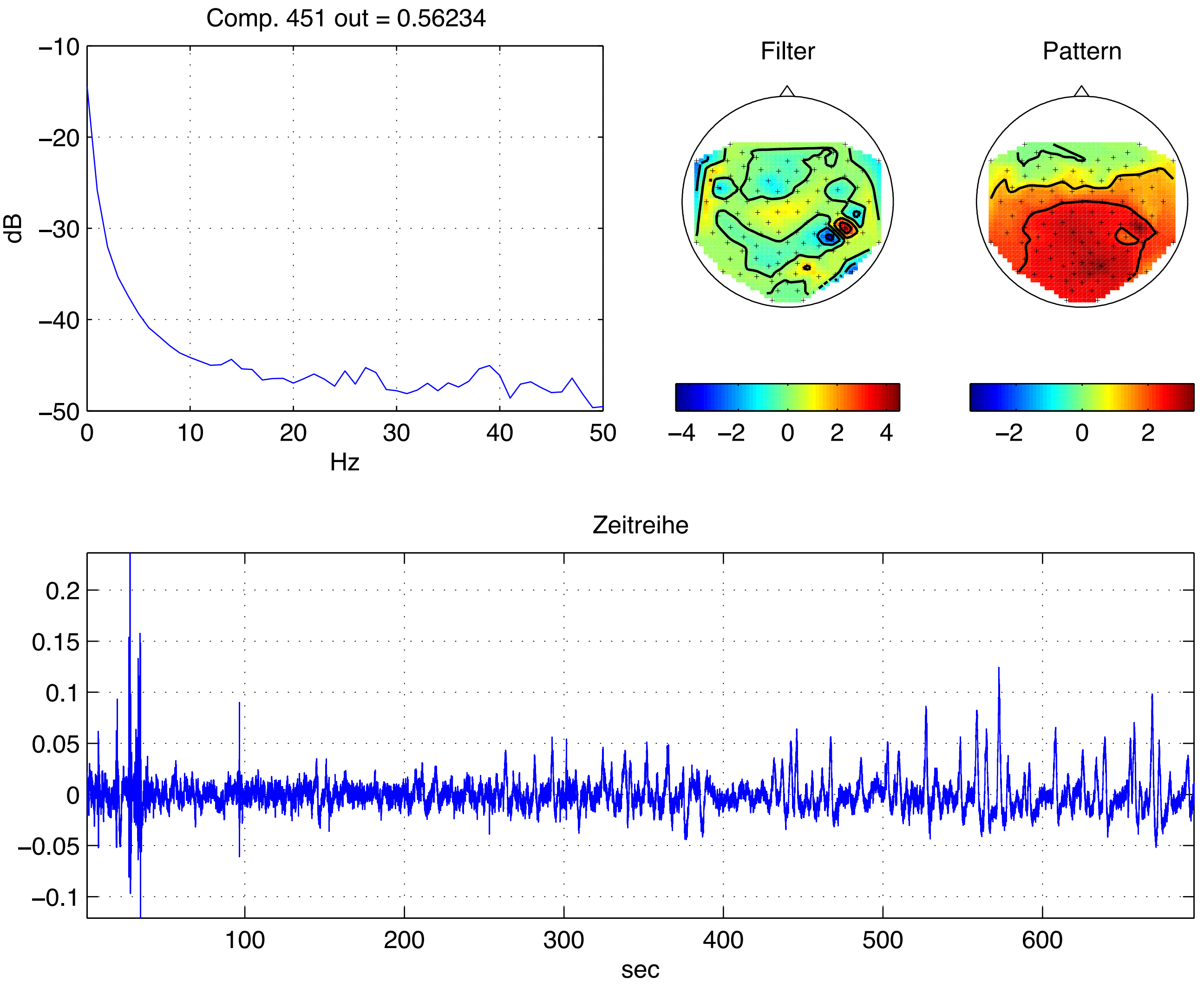
Comp. 451 out = 0.56234
dB
Hz
Filter
Pattern
Zeitreihe
sec
−10
−20
−30
−40
−50
0
10
20
30
40
50
−4
−2
0
2
4
−2
0
2
0.2
0.15
0.1
0.05
0
−0.05
−0.1
100
200
300
400
500
600

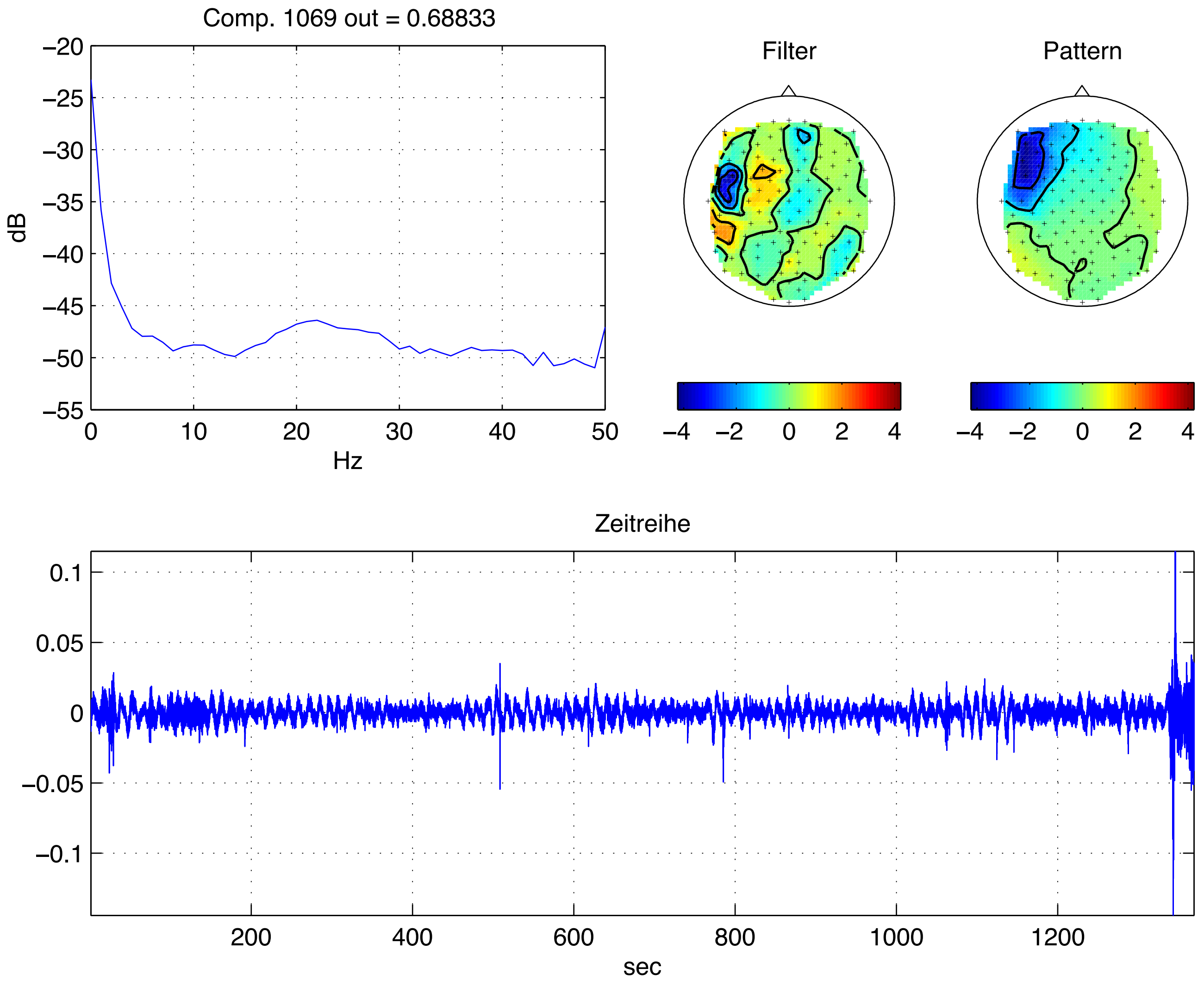
Comp. 1069 out = 0.68833
dB
Hz
Filter
Pattern
Zeitreihe
sec

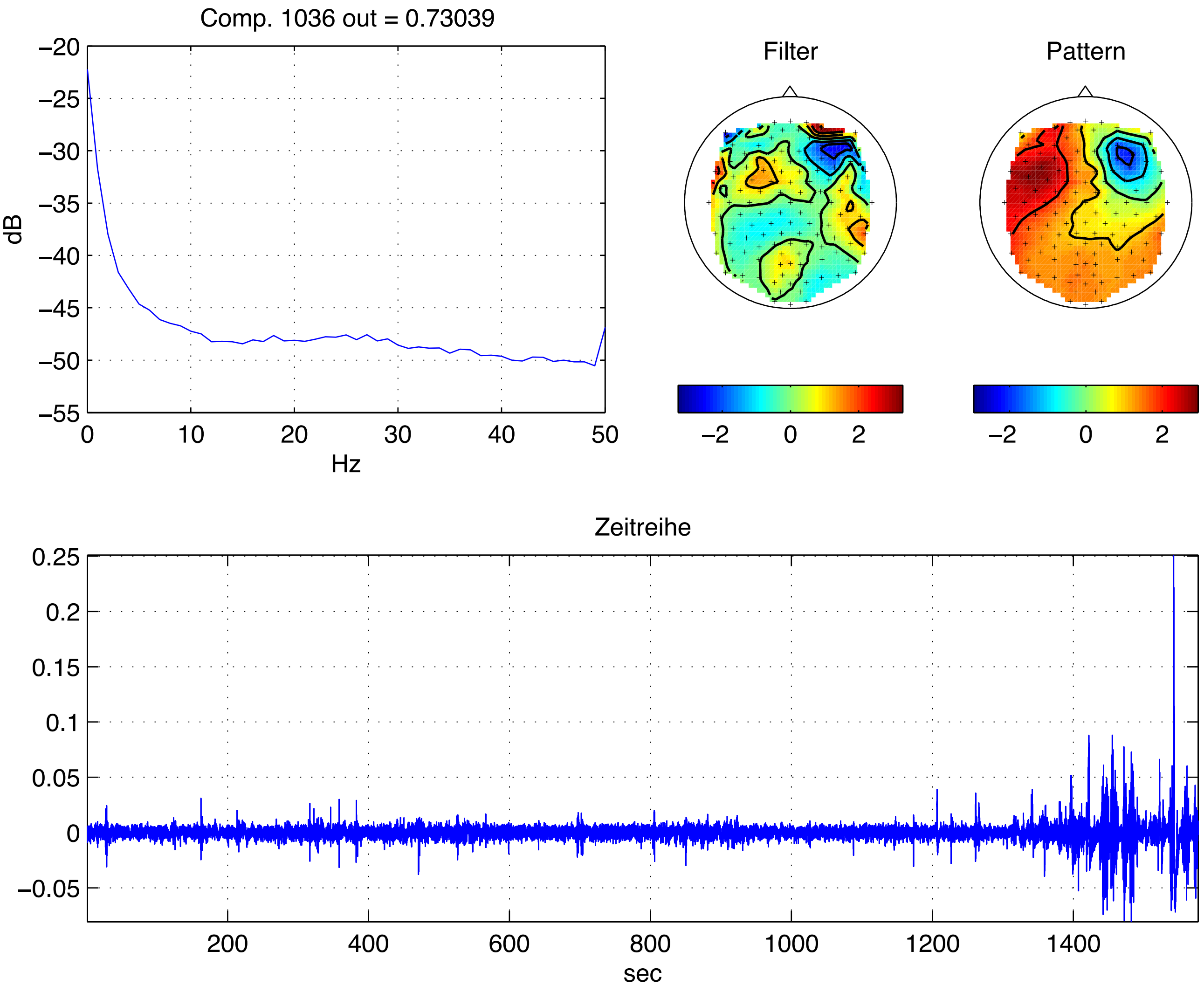

Comp. 1036 out = 0.73039
dB
Hz
Filter
Pattern
Zeitreihe
sec

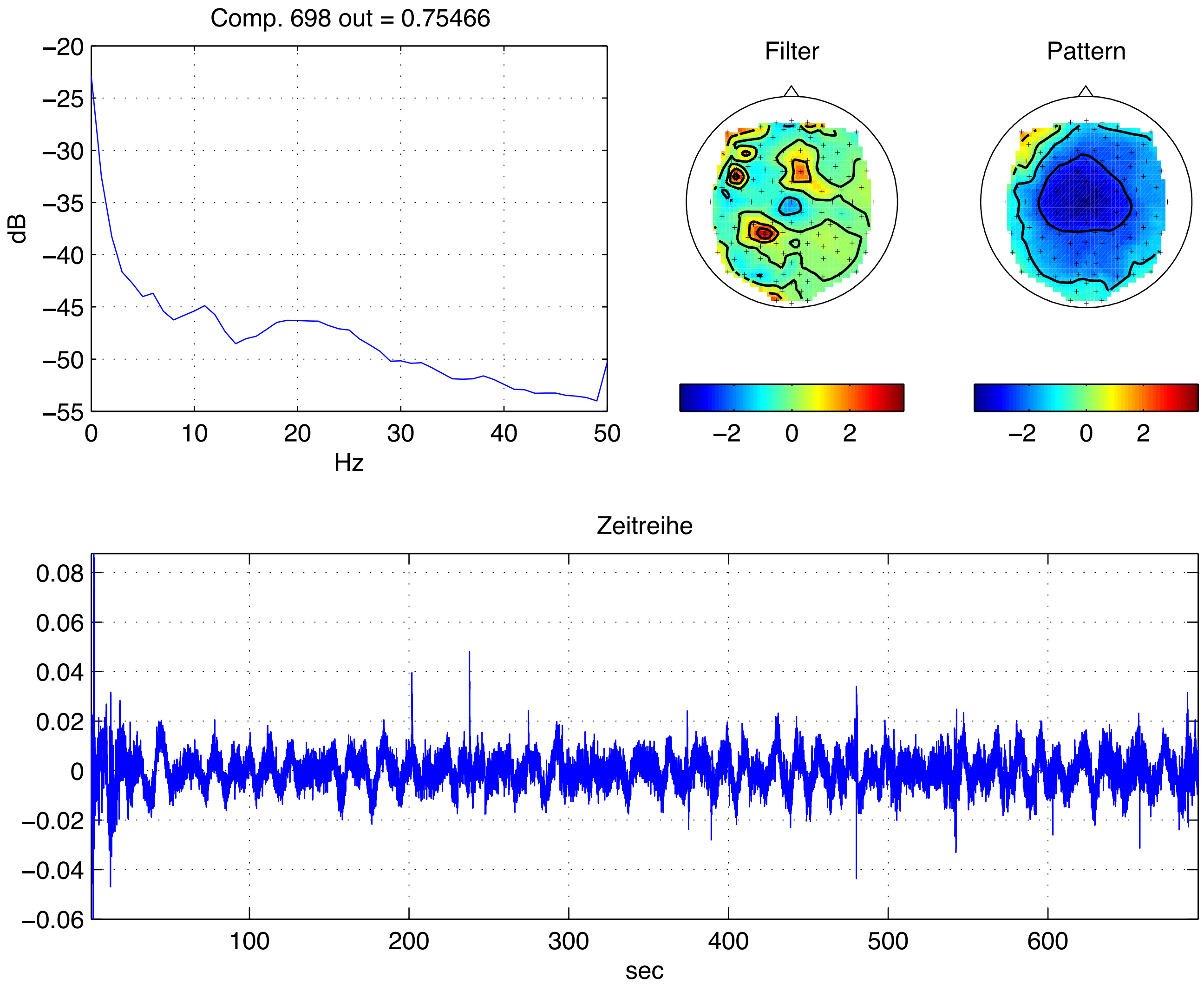
Comp. 698 out = 0.75466
dB
Hz
Filter
Pattern
Zeitreihe
sec

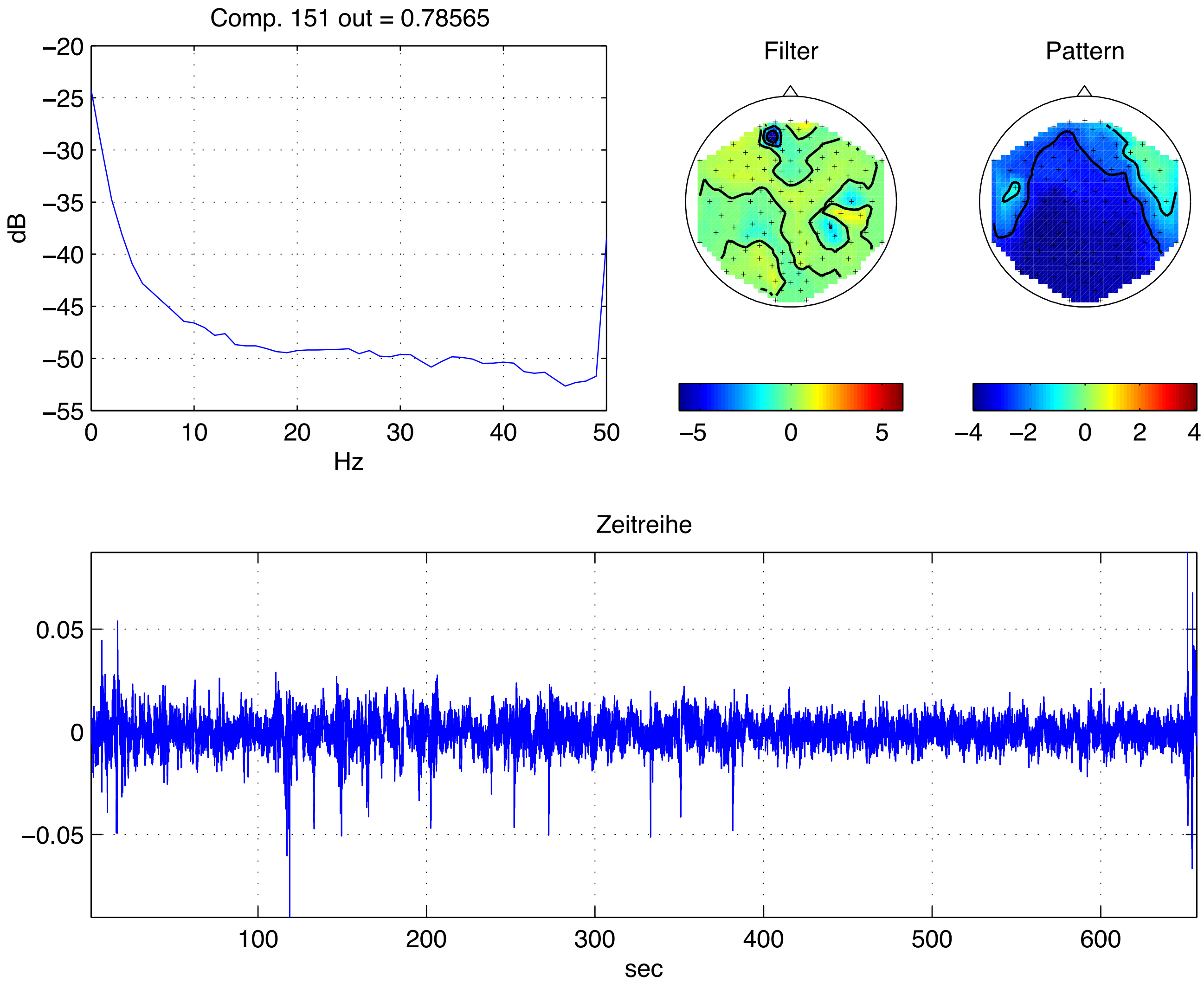
Comp. 151 out = 0.78565
dB
Hz
Filter
Pattern
Zeitreihe
sec

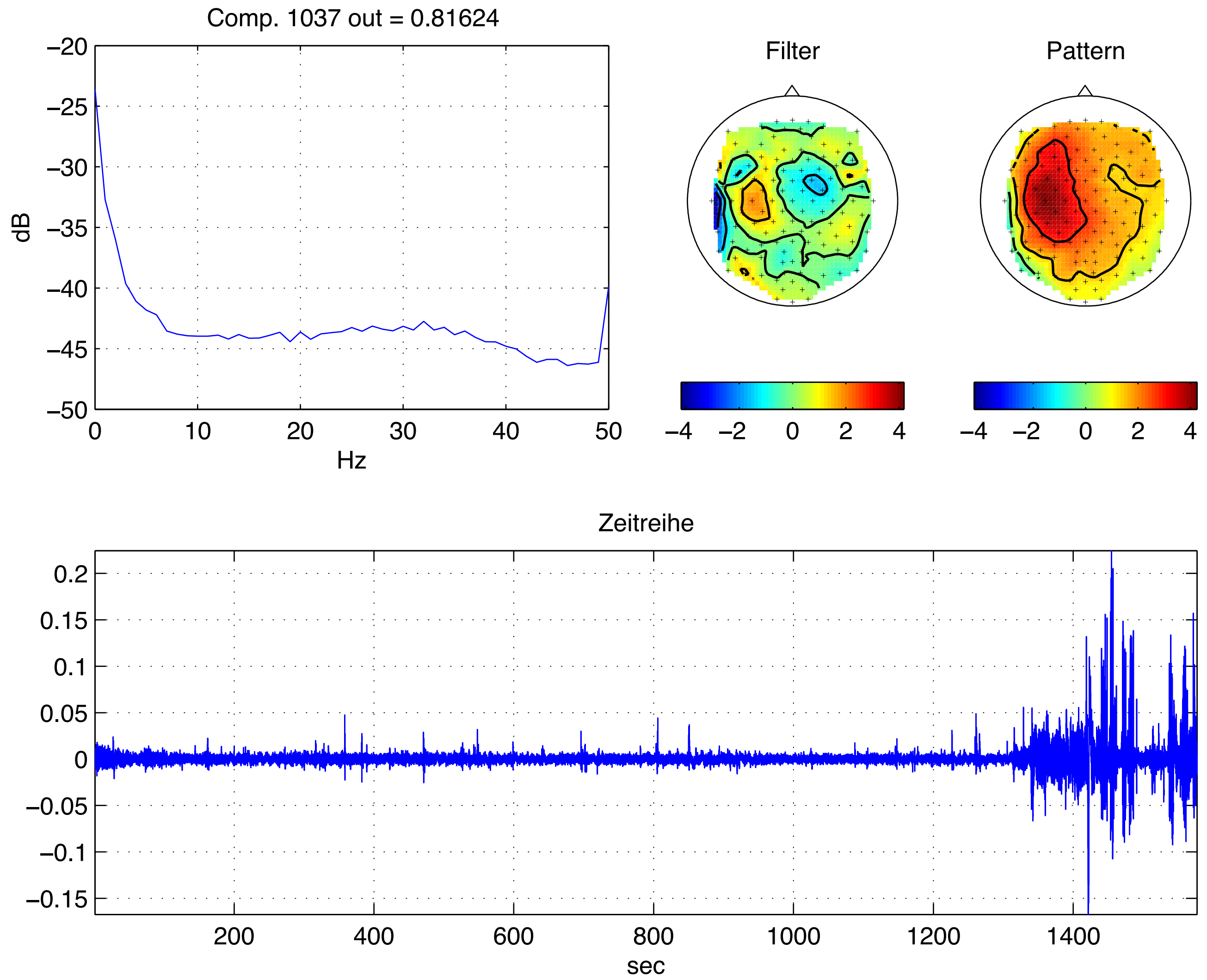
Comp. 1037 out = 0.81624
dB
Hz
Filter
Pattern
Zeitreihe
sec

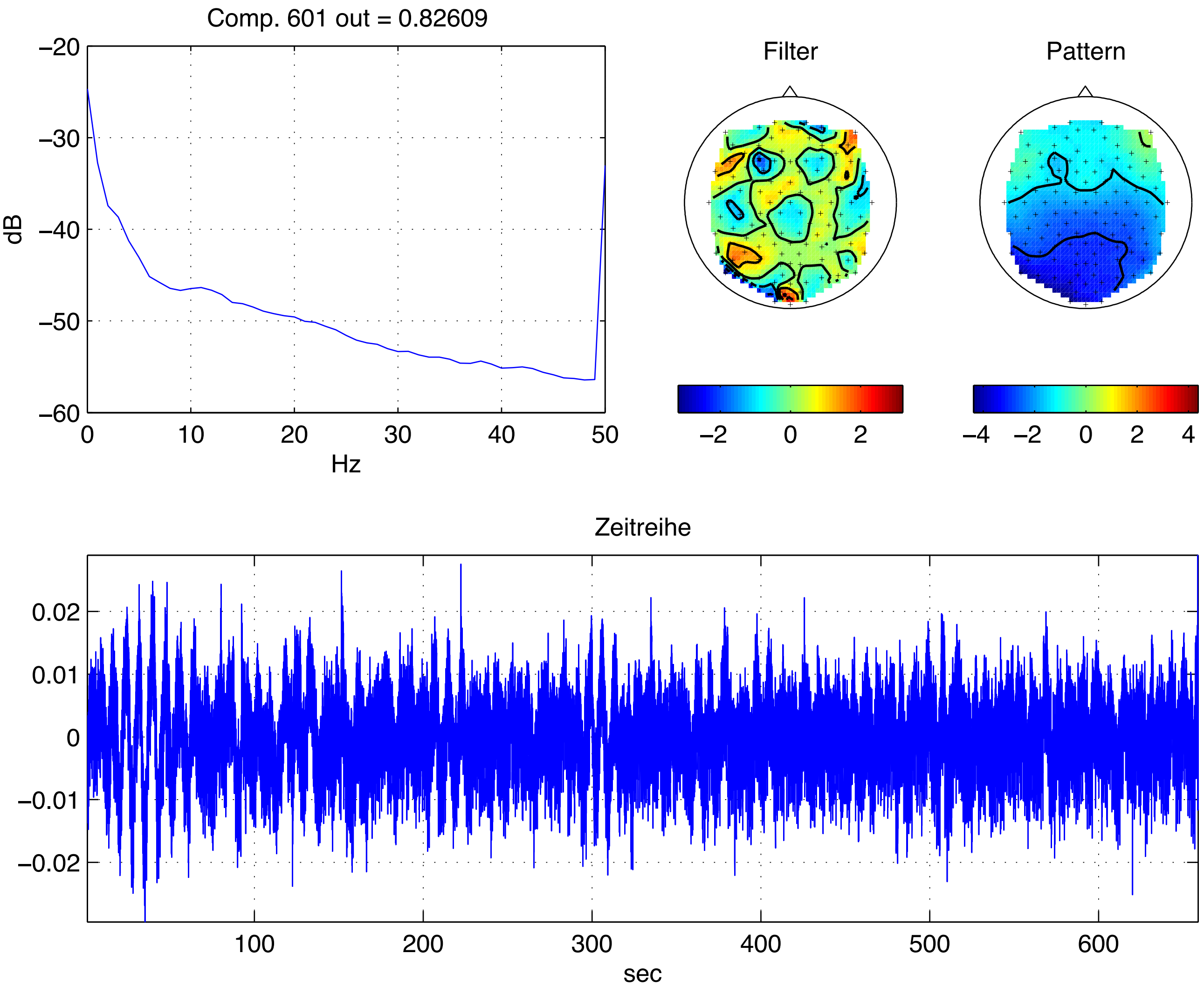

Comp. 601 out = 0.82609
dB
Hz
−20
−30
−40
−50
−60
0
10
20
30
40
50
Filter
Pattern
−2
0
2
−4
−2
0
2
4
Zeitreihe
0.02
0.01
0
−0.01
−0.02
100
200
300
400
500
600
sec

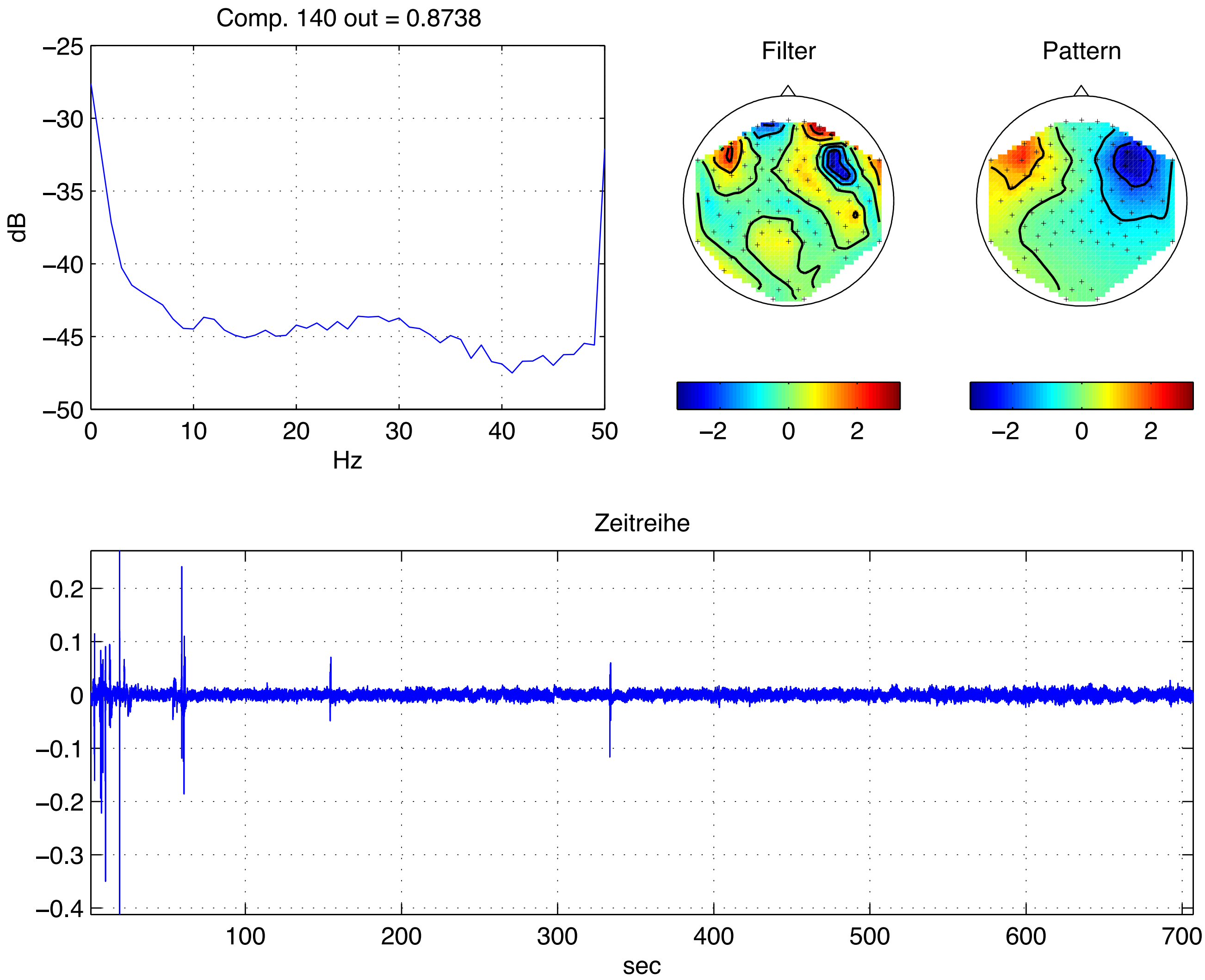
Comp. 140 out = 0.8738
dB
Hz
Filter
Pattern
Zeitreihe
sec

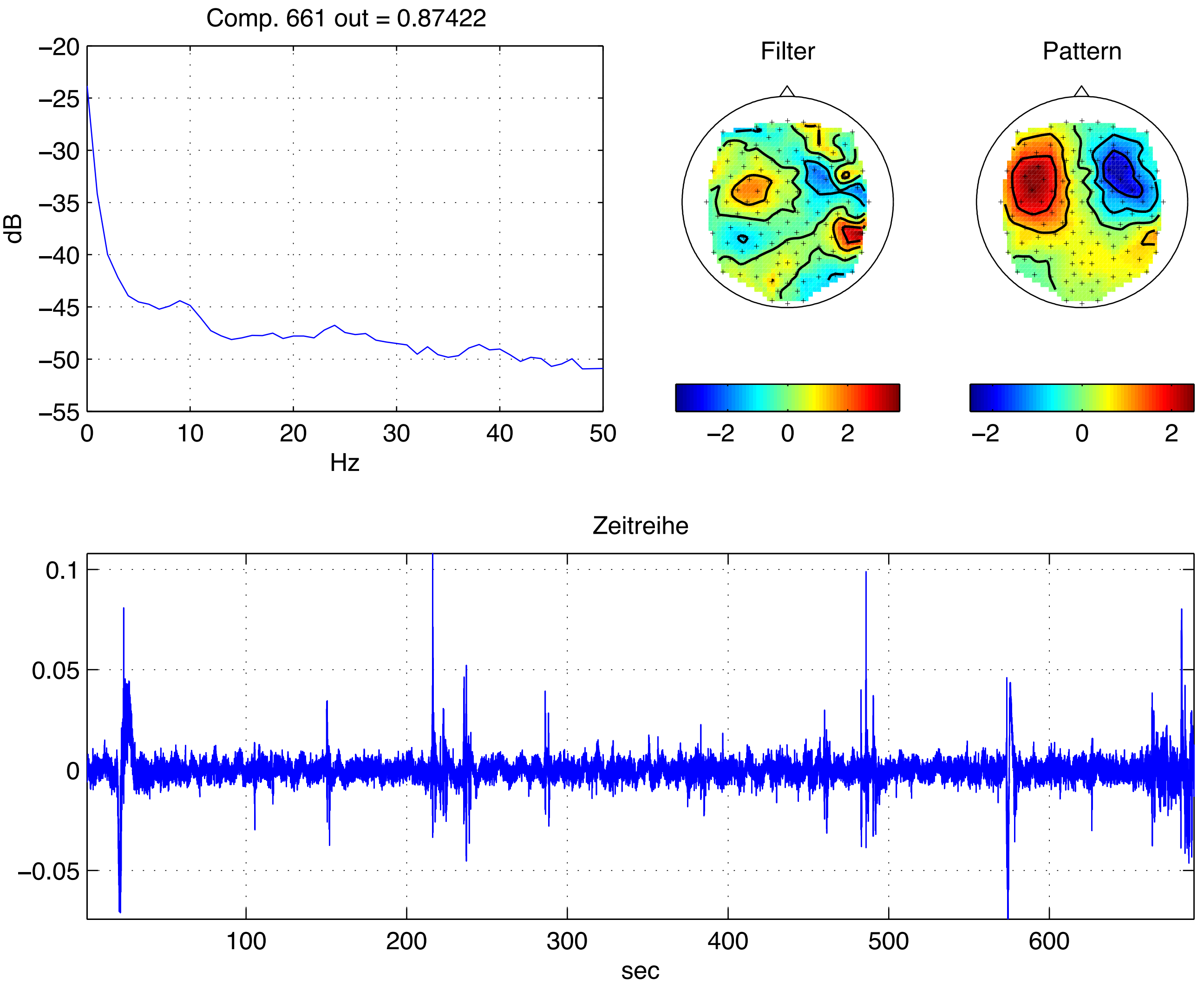

Comp. 661 out = 0.87422
dB
Hz
Filter
Pattern
Zeitreihe
sec

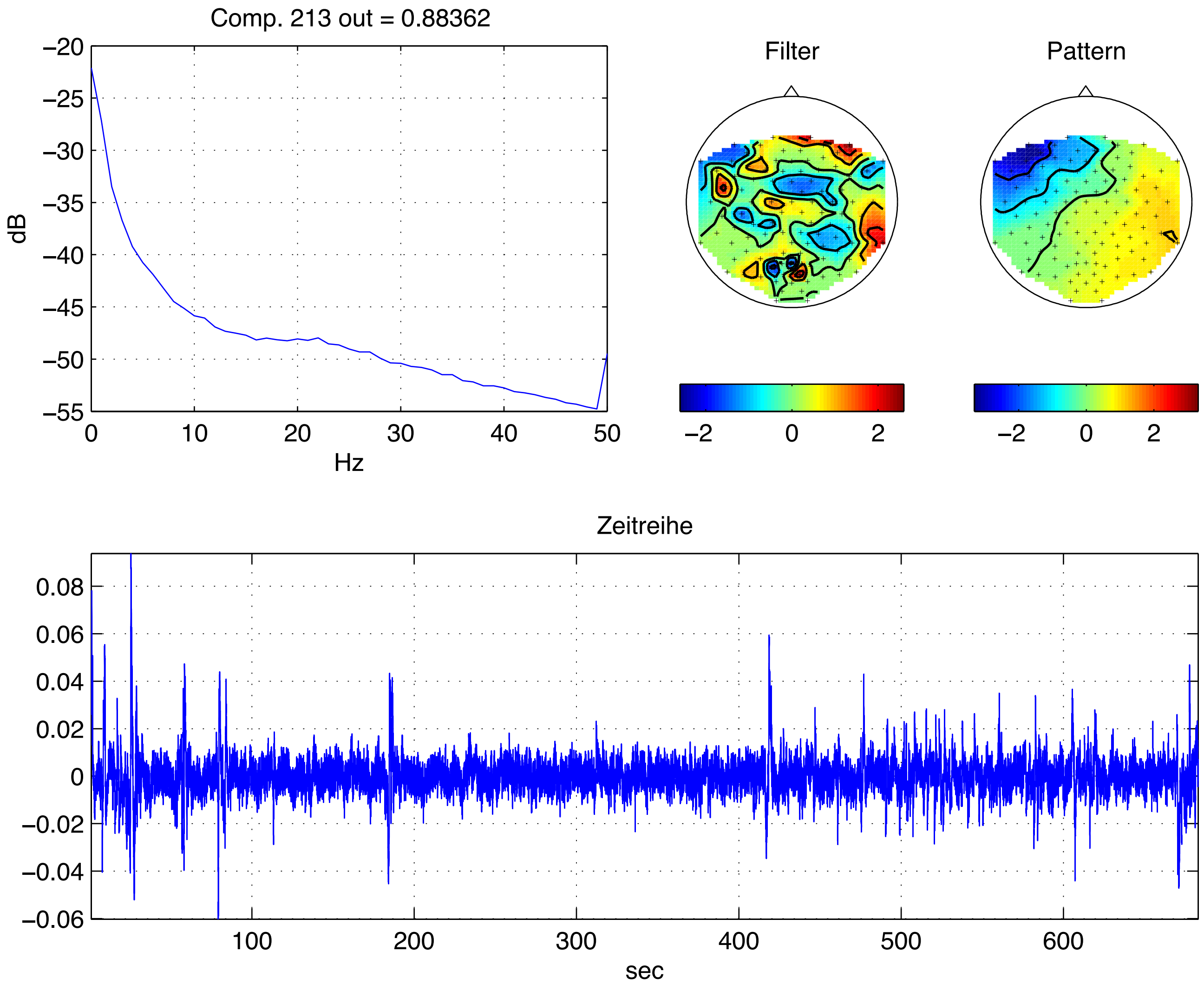
Comp. 213 out = 0.88362
dB
Hz
Filter
Pattern
Zeitreihe
sec

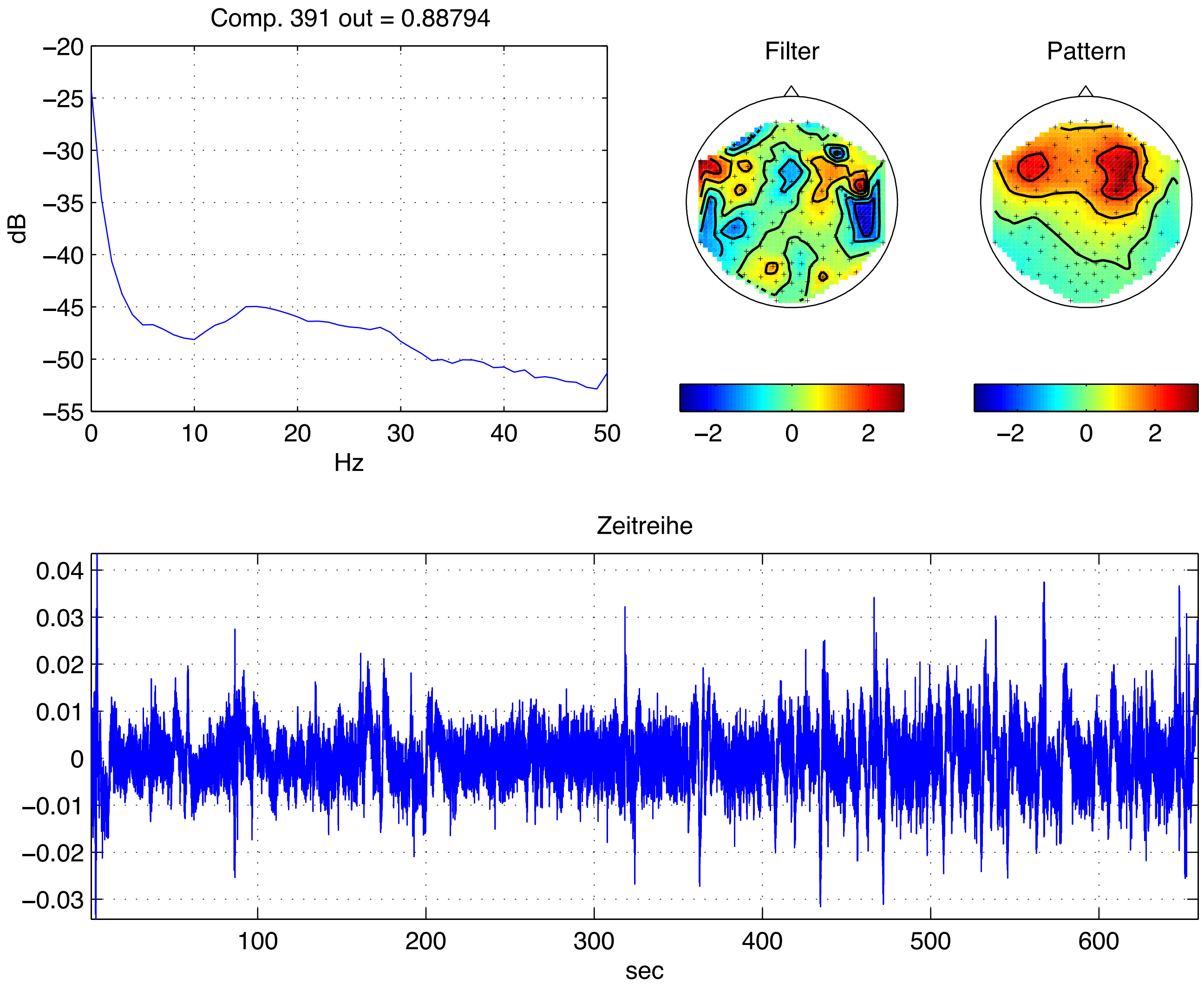

Comp. 391 out = 0.88794
dB
Hz
Filter
Pattern
Zeitreihe
sec

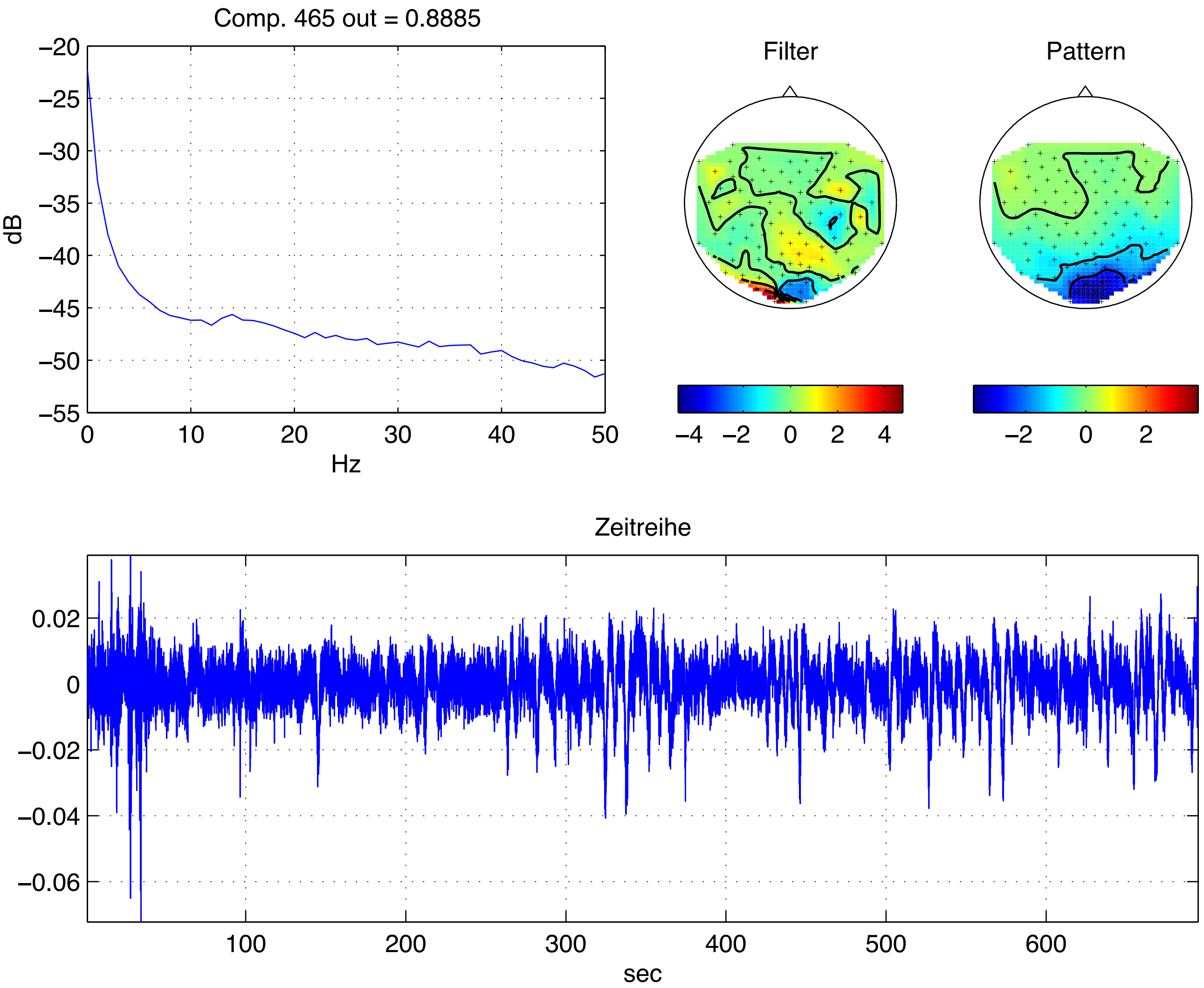

Comp. 465 out = 0.8885
dB
Hz
Filter
Pattern
Zeitreihe
sec

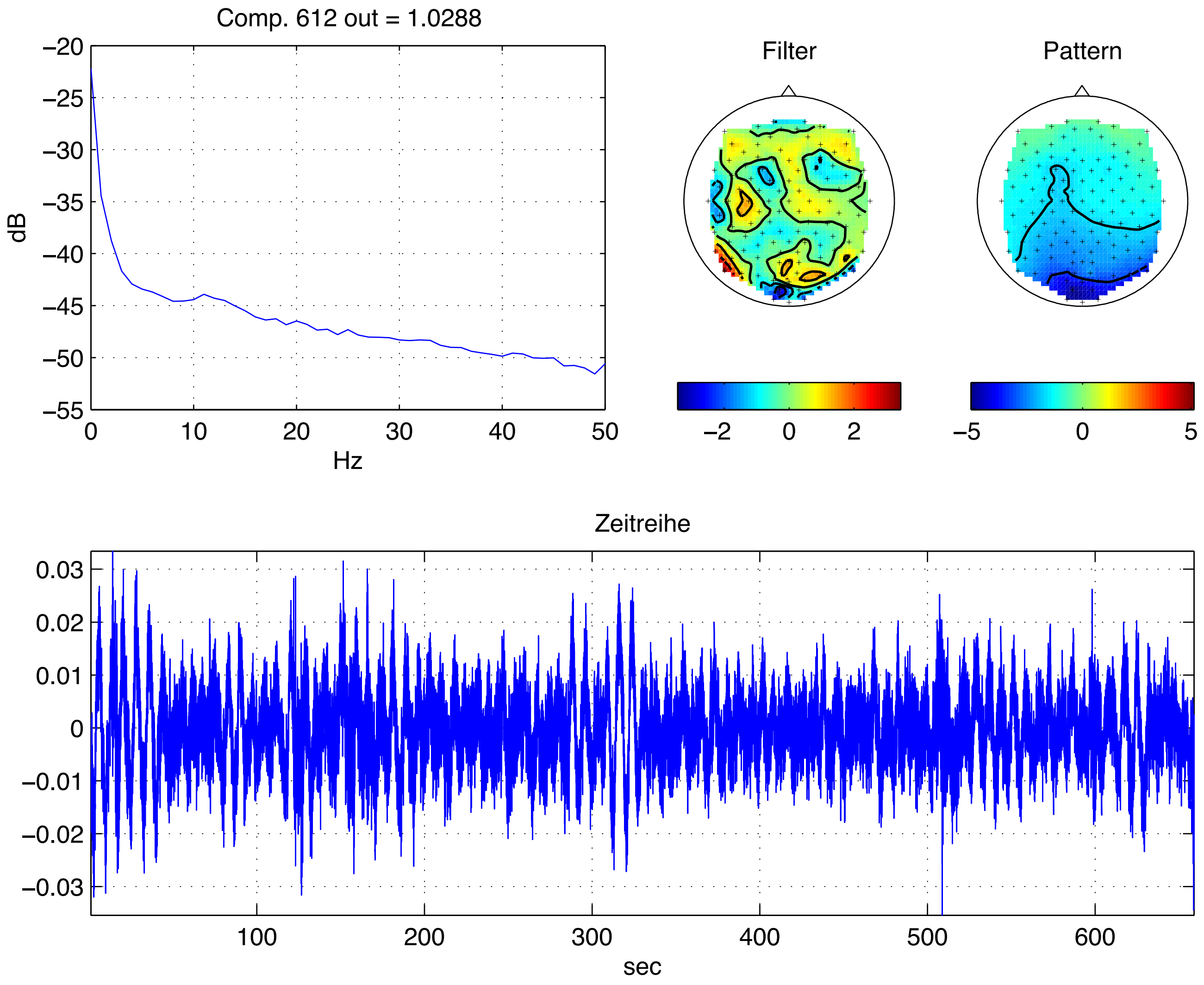

Comp. 612 out = 1.0288
dB
Hz
Filter
Pattern
Zeitreihe
sec

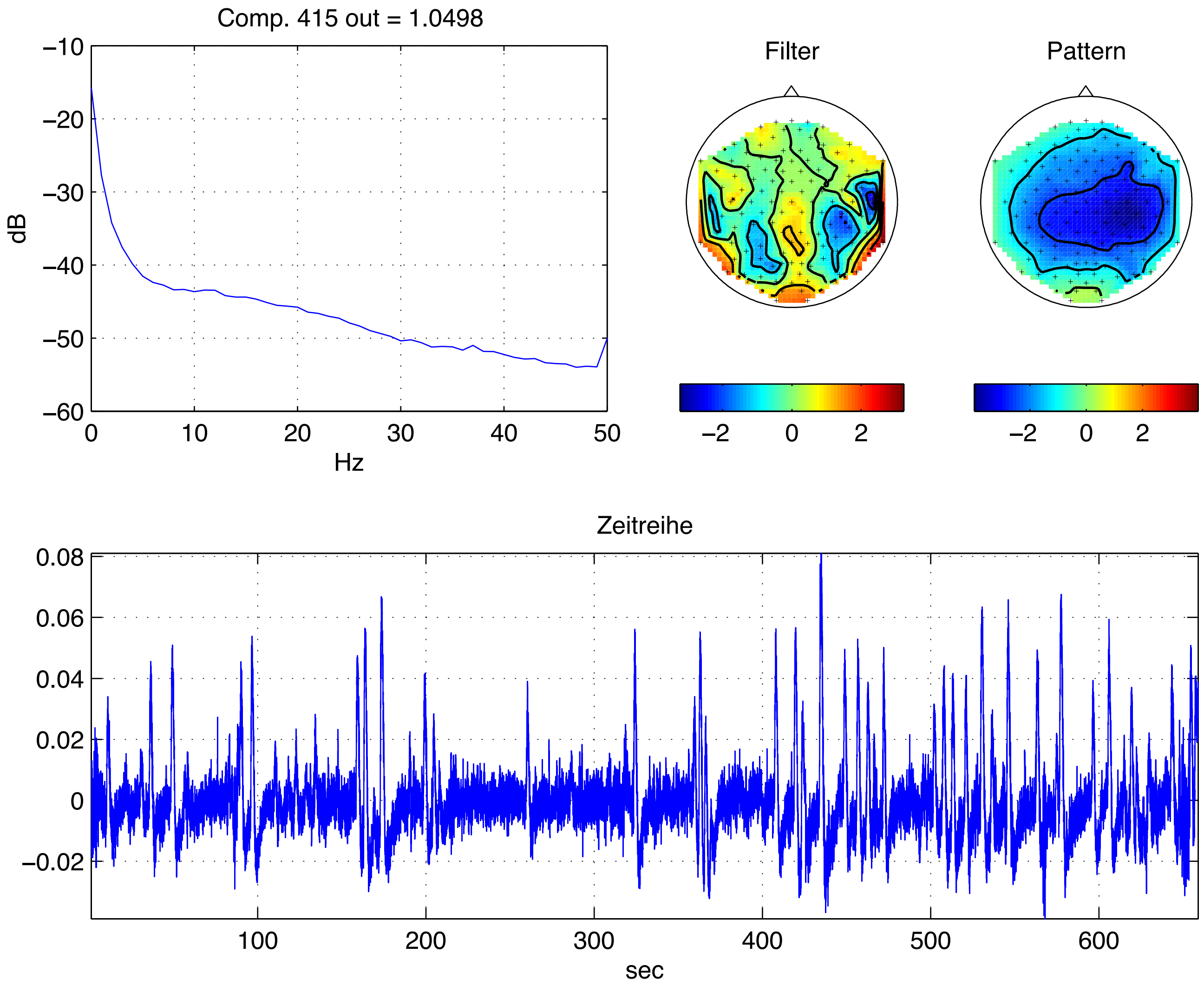
Comp. 415 out = 1.0498
dB
Hz
Filter
Pattern
Zeitreihe
sec

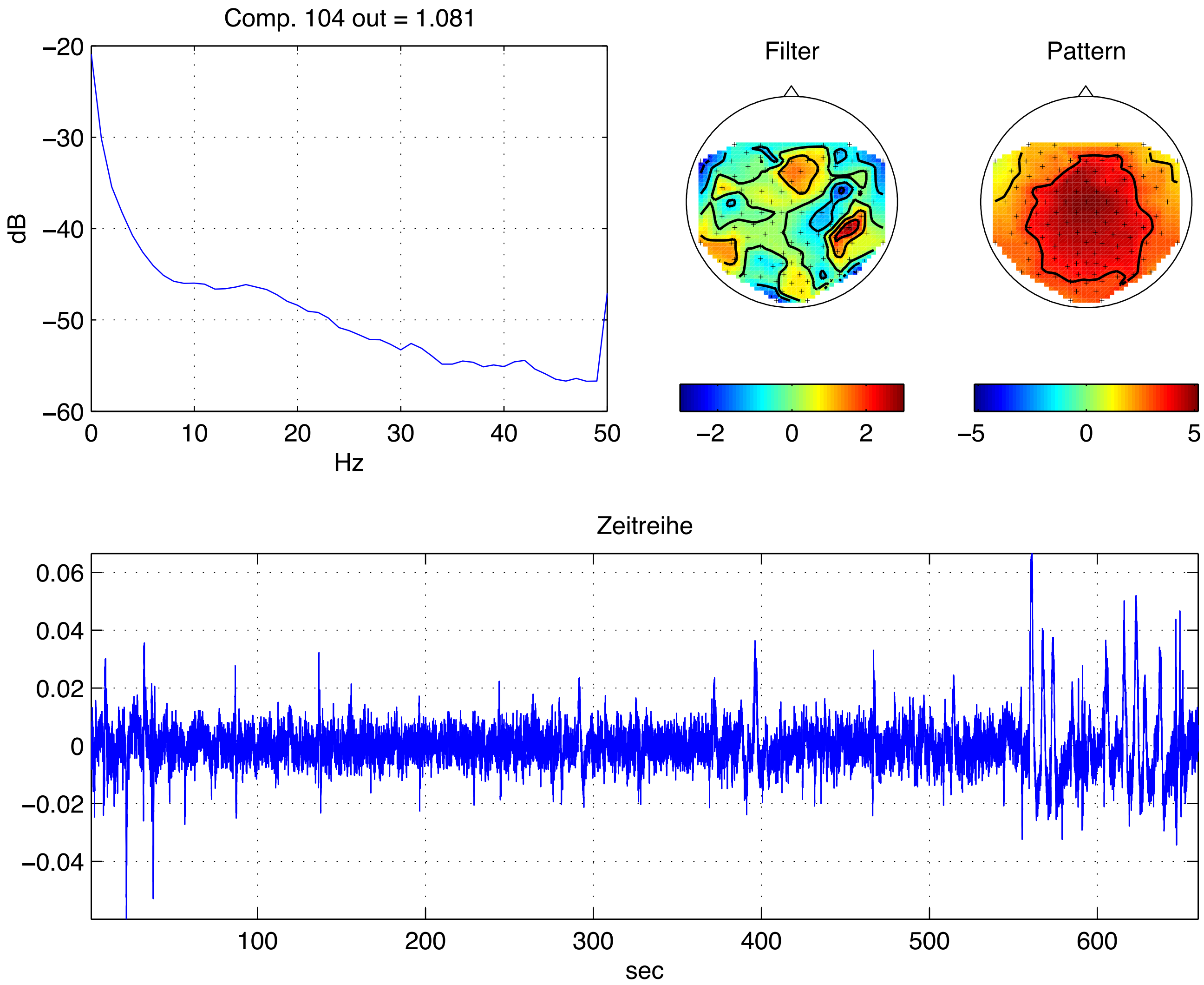

Comp. 104 out = 1.081
dB
Hz
Filter
Pattern
Zeitreihe
sec

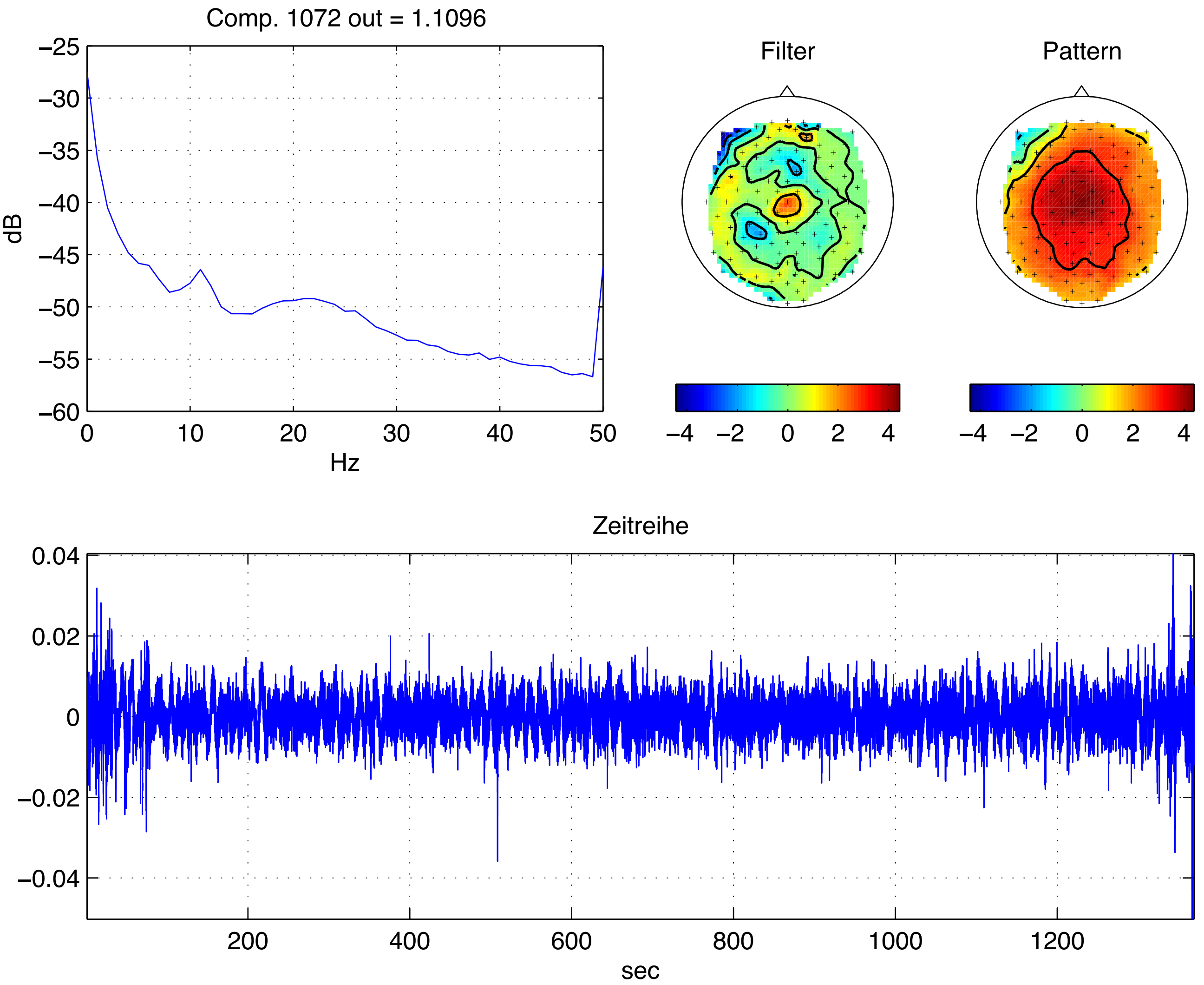

Comp. 1072 out = 1.1096
dB
Hz
Filter
Pattern
Zeitreihe
sec

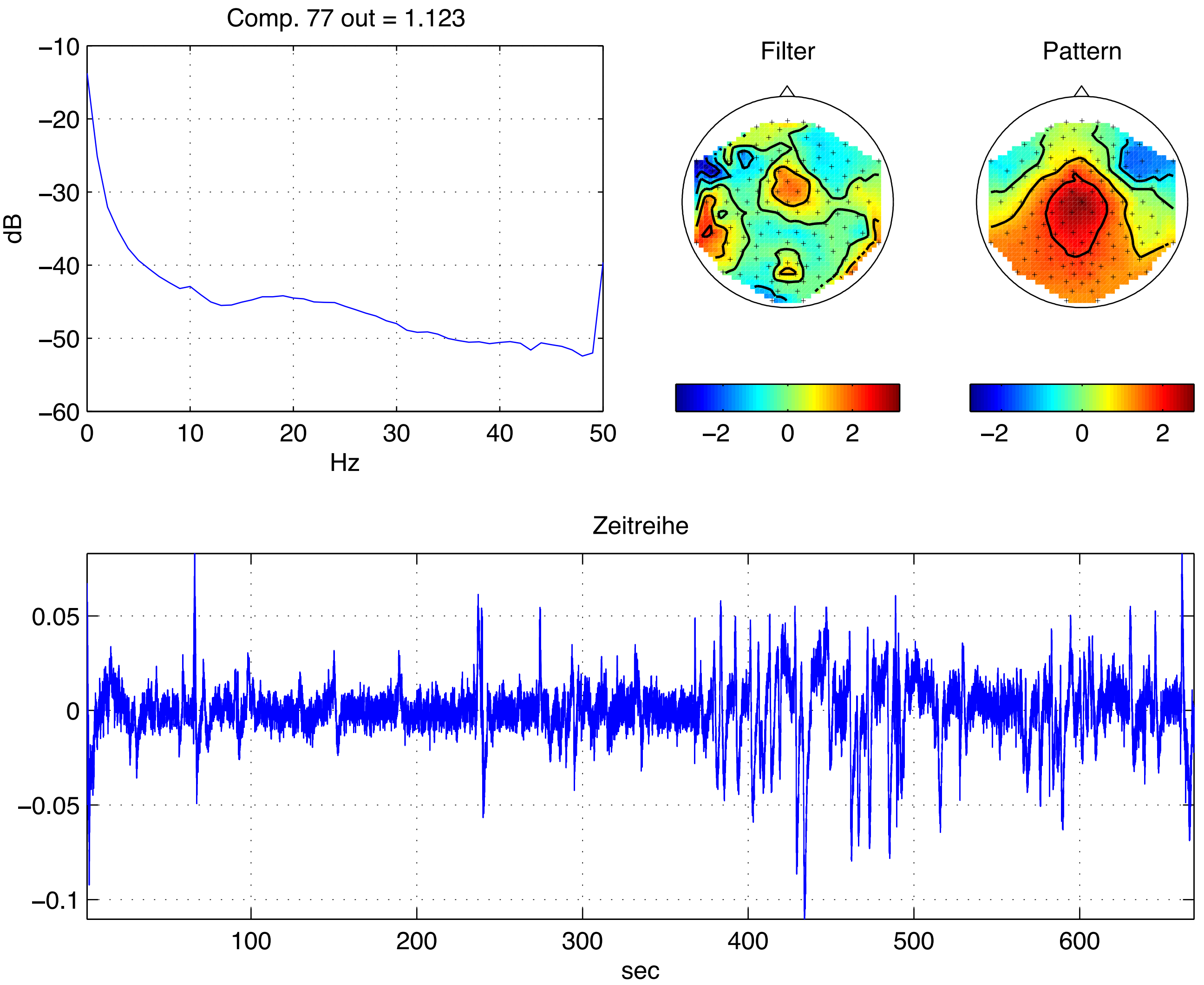
Comp. 77 out = 1.123
dB
Hz
Filter
Pattern
Zeitreihe
sec

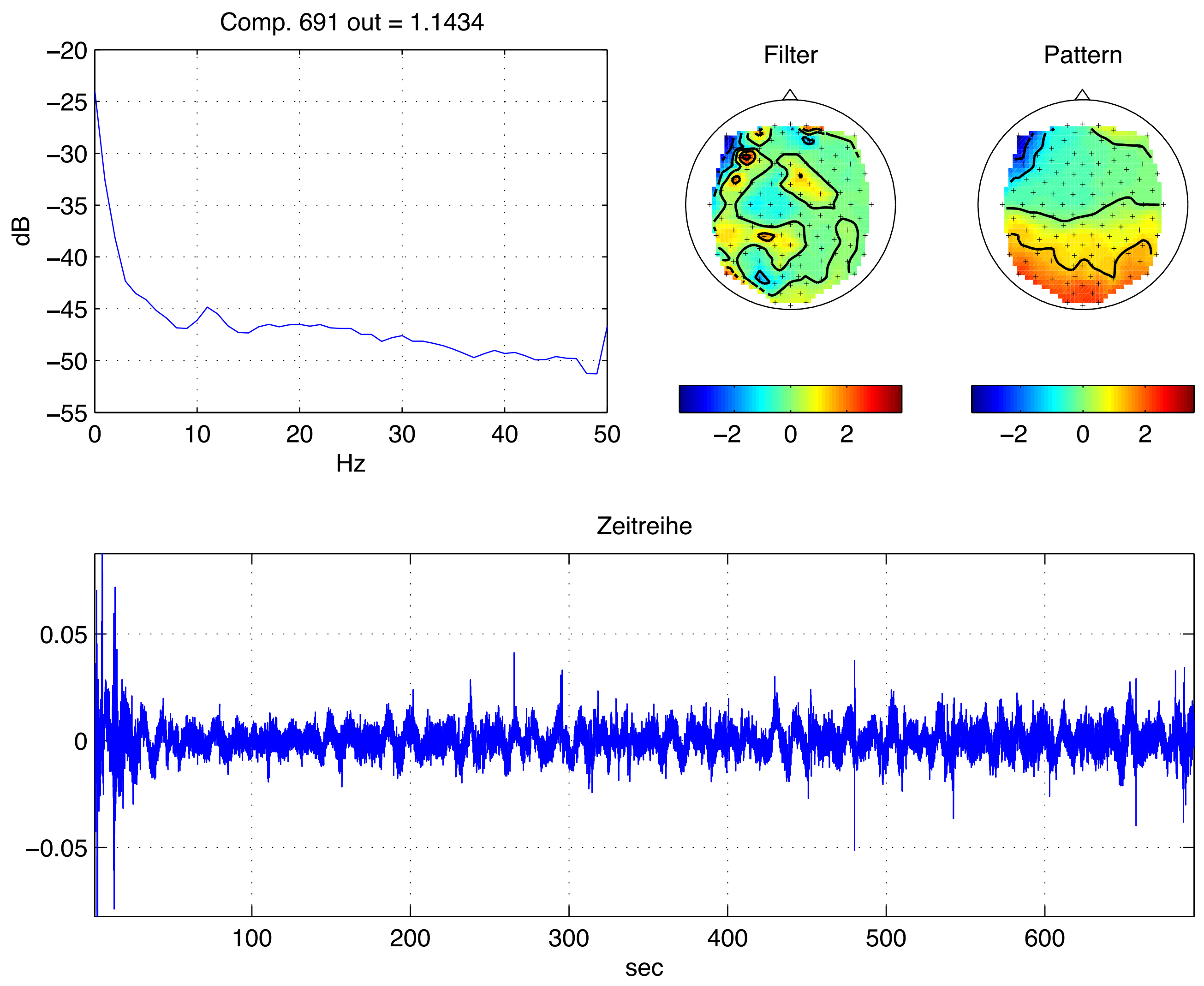

Comp. 691 out = 1.1434
dB
Hz
−20
−25
−30
−35
−40
−45
−50
−55
0
10
20
30
40
50
Filter
Pattern
−2
0
2
−2
0
2
Zeitreihe
0.05
0
−0.05
100
200
300
400
500
600
sec

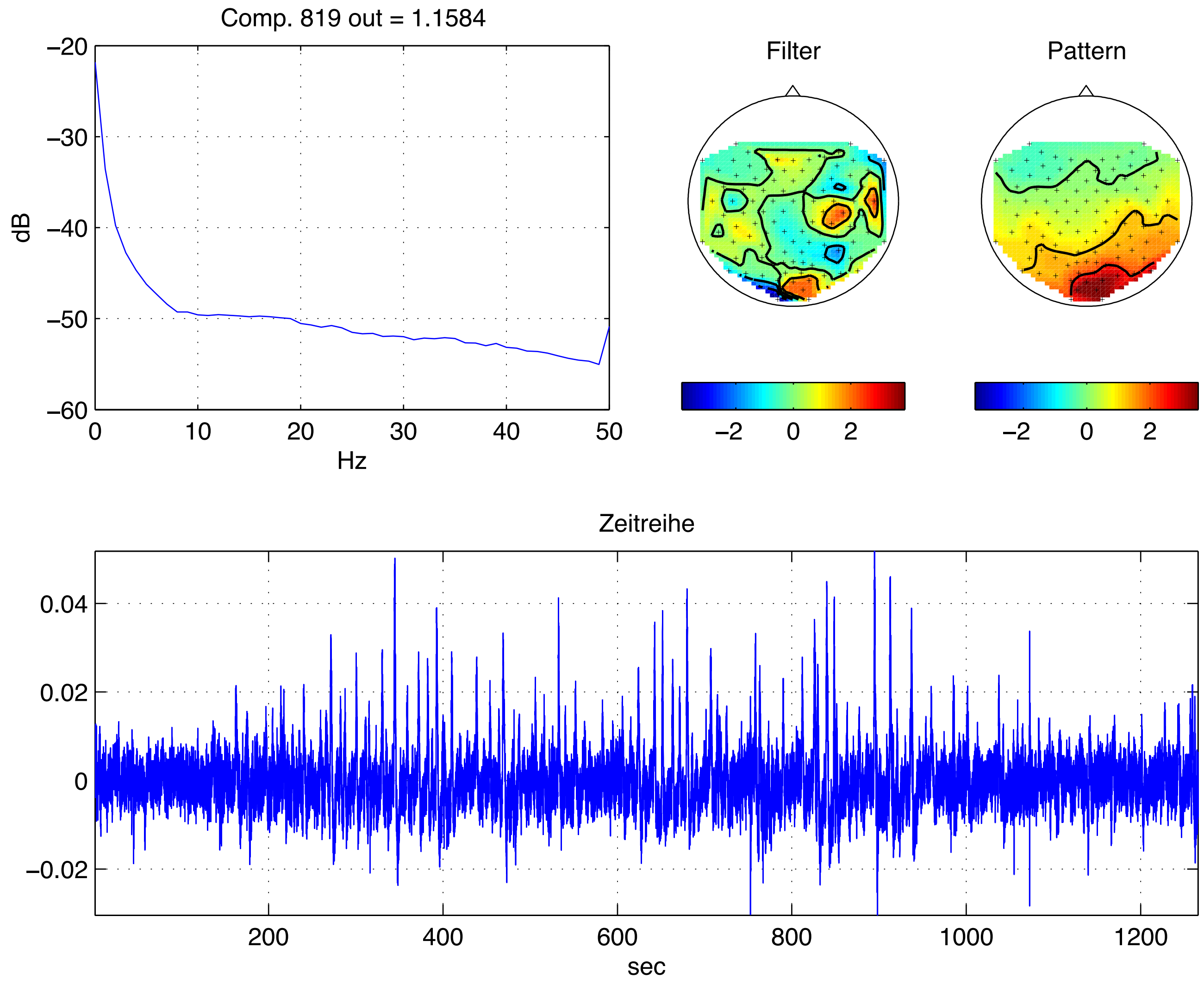
Comp. 819 out = 1.1584
dB
Hz
Filter
Pattern
Zeitreihe
sec

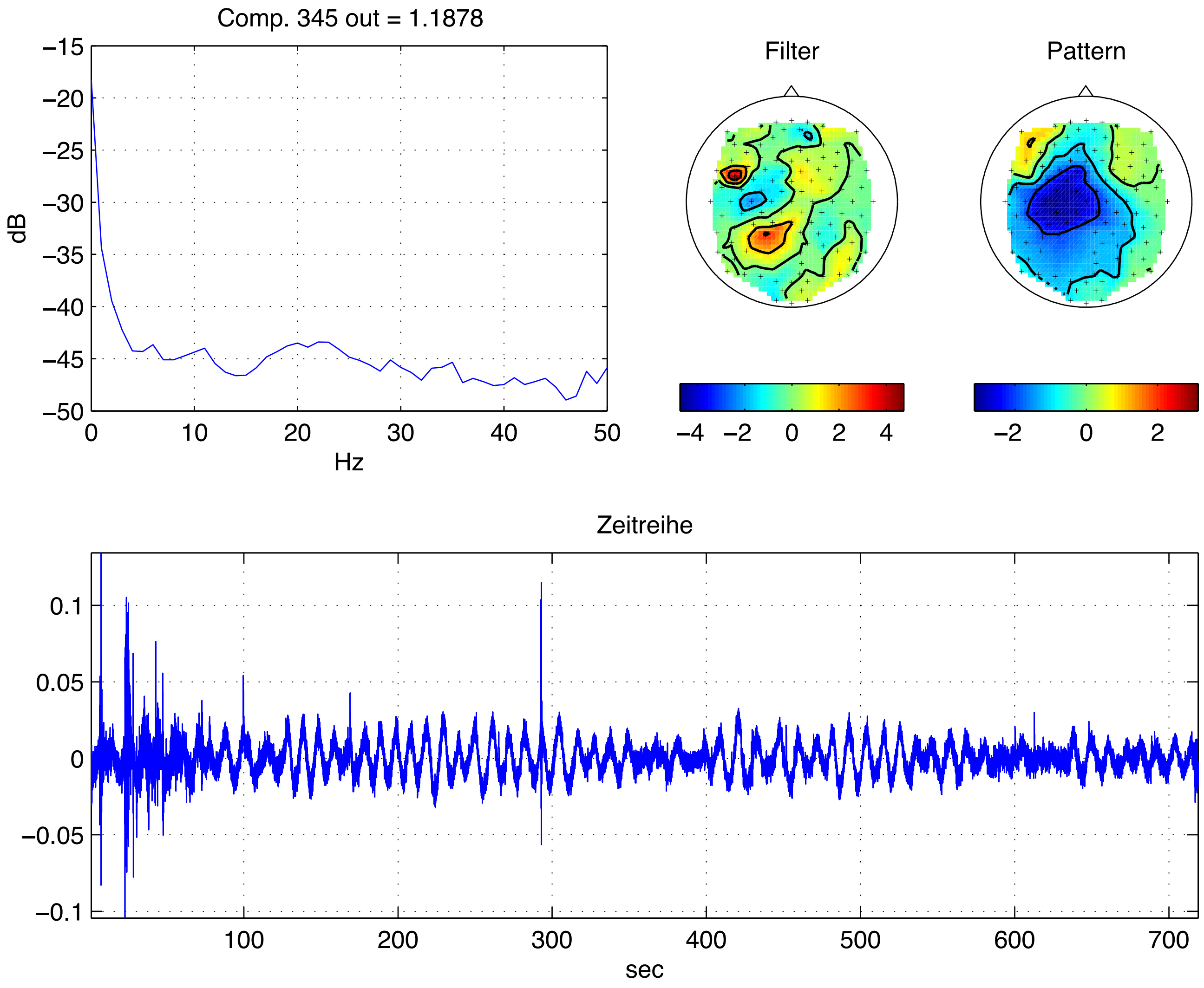
Comp. 345 out = 1.1878
dB
Hz
Filter
Pattern
Zeitreihe
sec

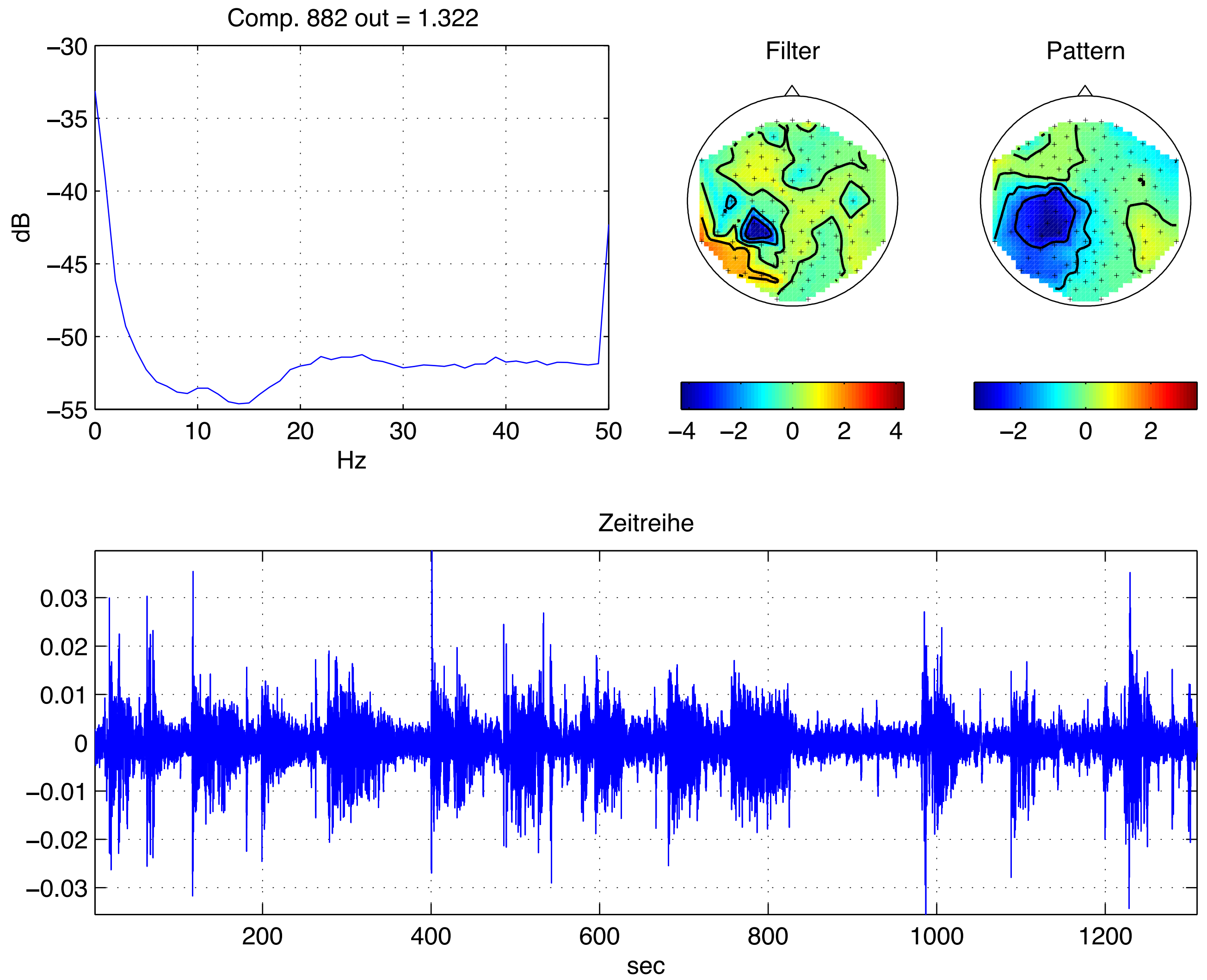
Comp. 882 out = 1.322
dB
Hz
Filter
Pattern
Zeitreihe
sec

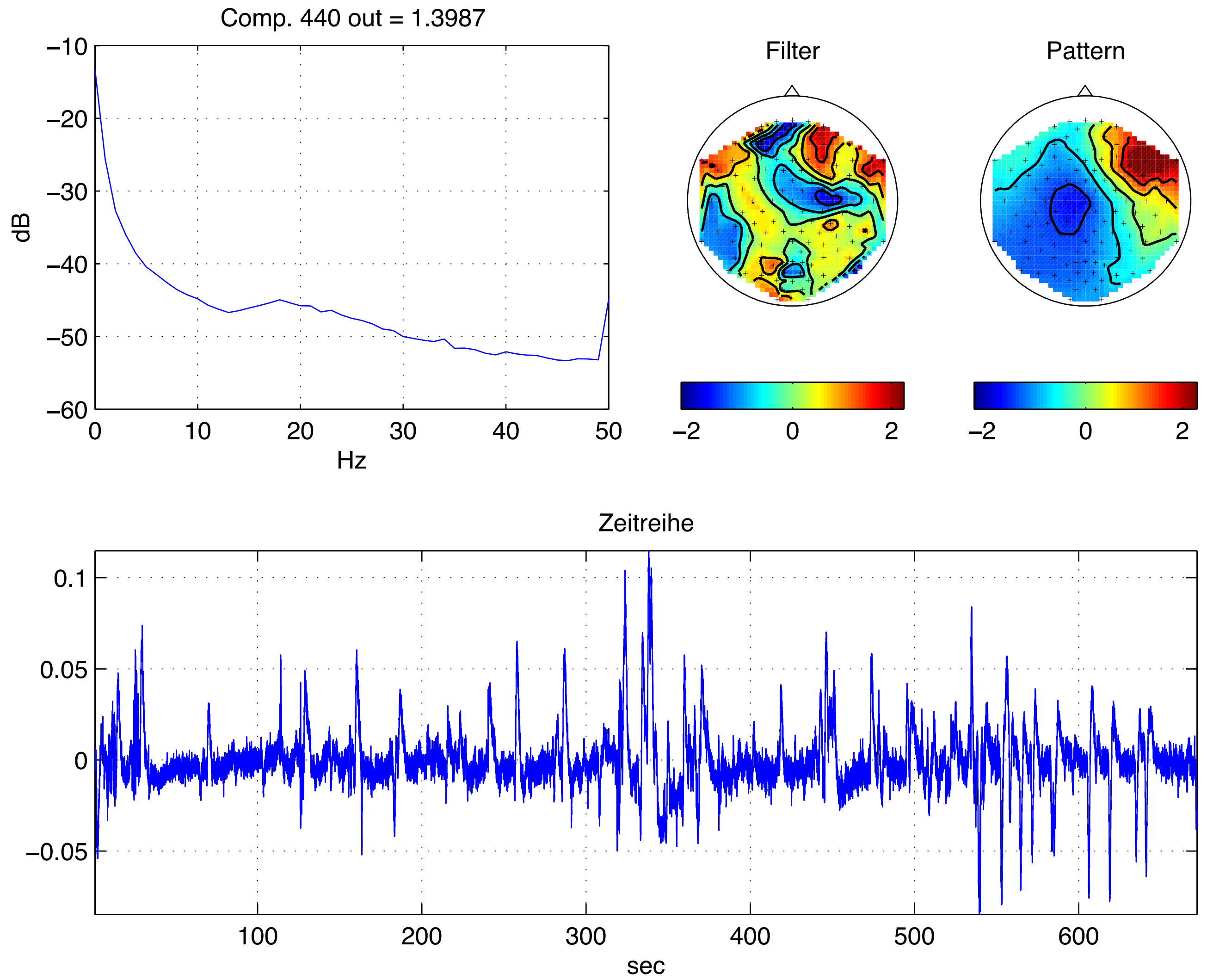

Comp. 440 out = 1.3987
dB
Hz
Filter
Pattern
Zeitreihe
sec

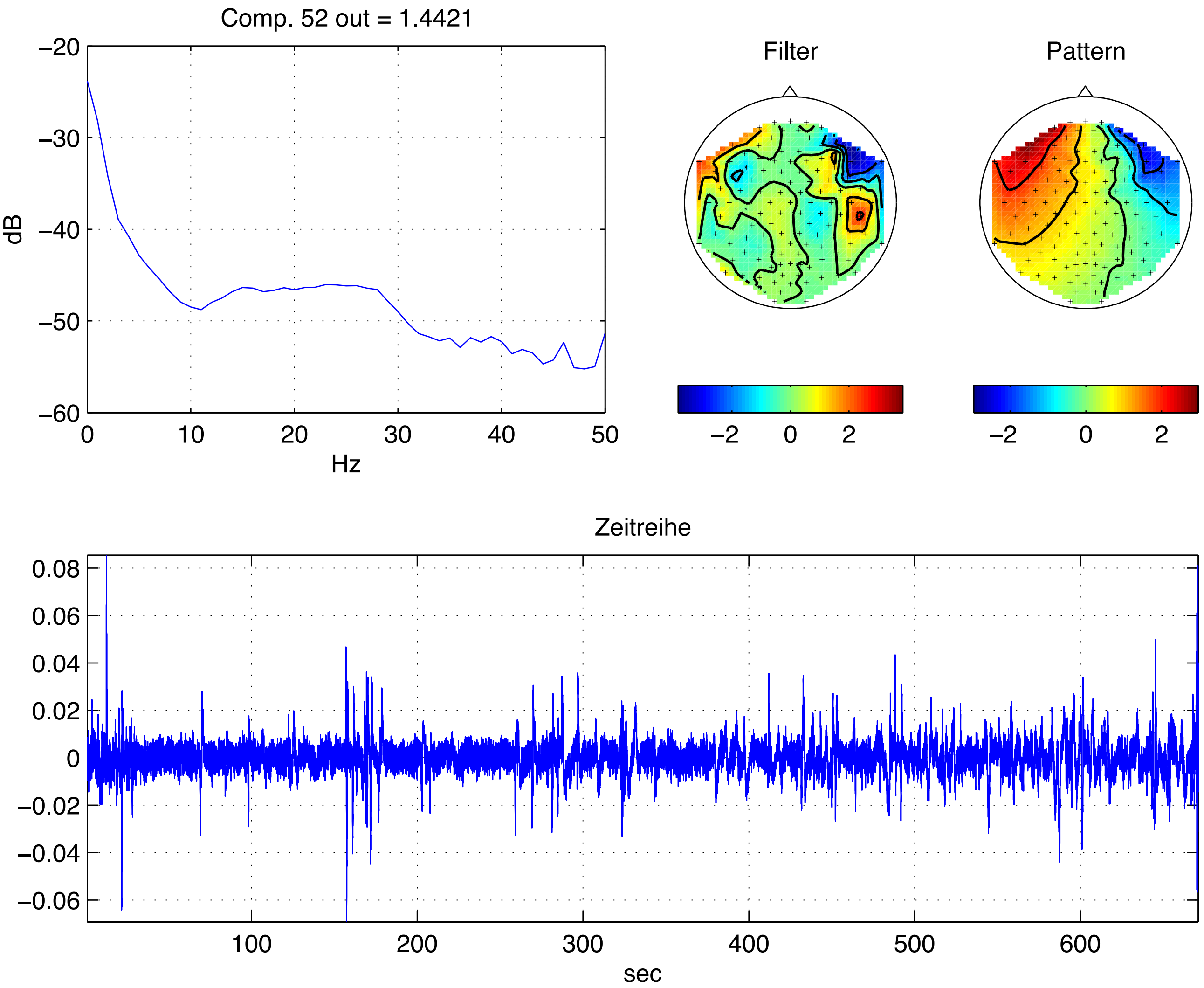

Comp. 52 out = 1.4421
dB
Hz
Filter
Pattern
Zeitreihe
sec

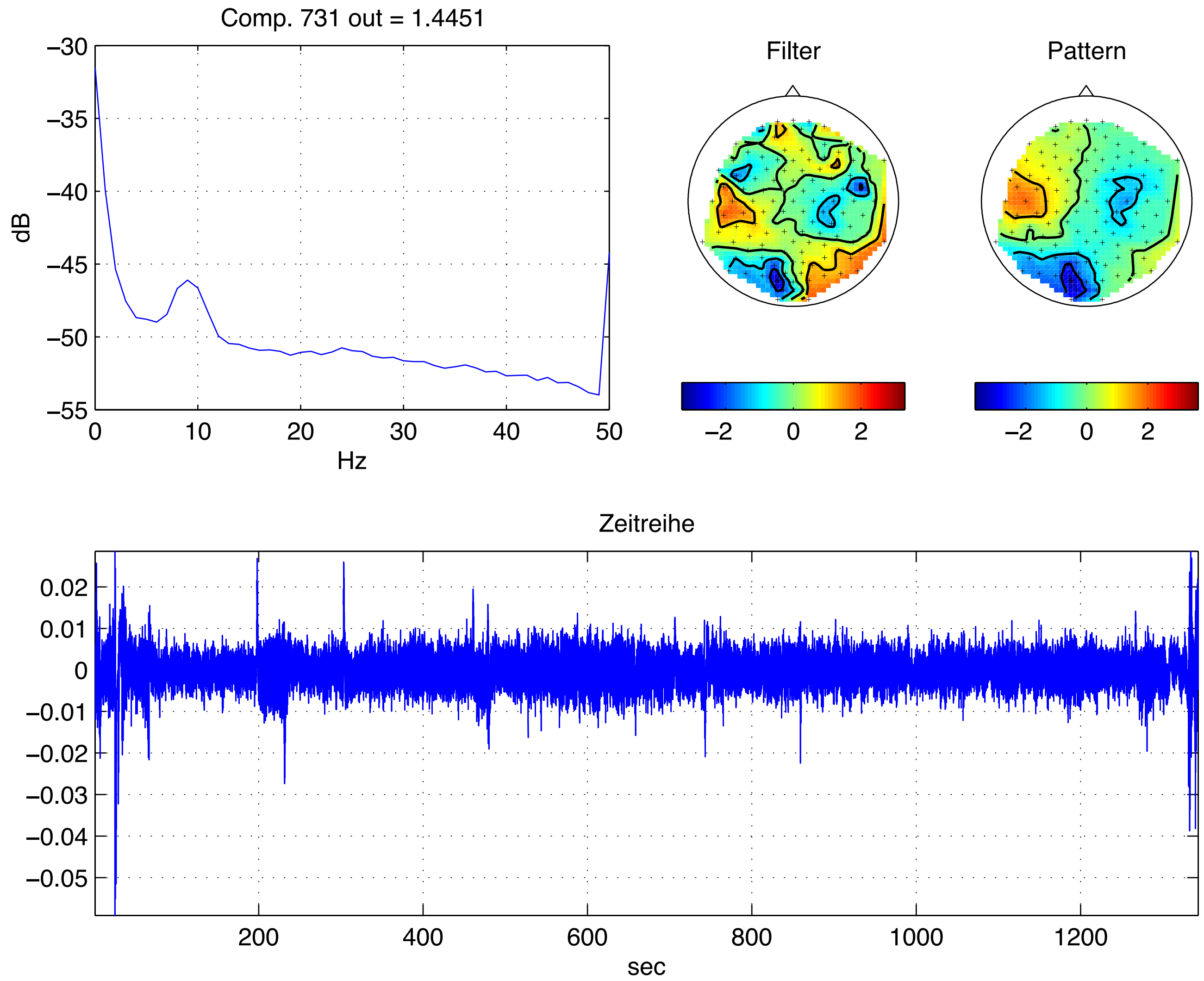

Comp. 731 out = 1.4451
dB
Hz
−30
−35
−40
−45
−50
−55
0
10
20
30
40
50
Filter
Pattern
−2
0
2
Zeitreihe
0.02
0.01
0
-0.01
-0.02
-0.03
-0.04
-0.05
200
400
600
800
1000
1200
sec

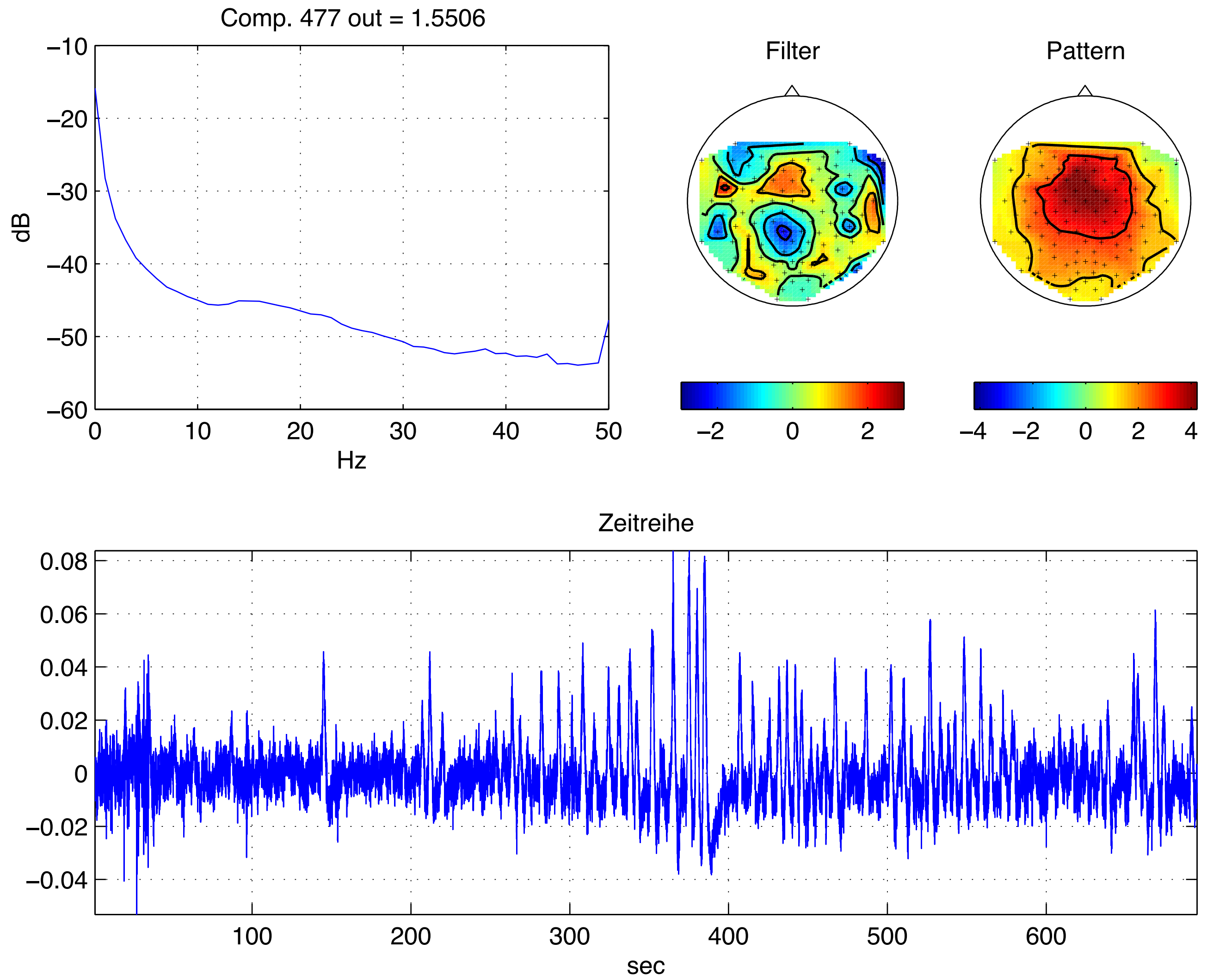

Comp. 477 out = 1.5506
dB
Hz
Filter
Pattern
Zeitreihe
sec

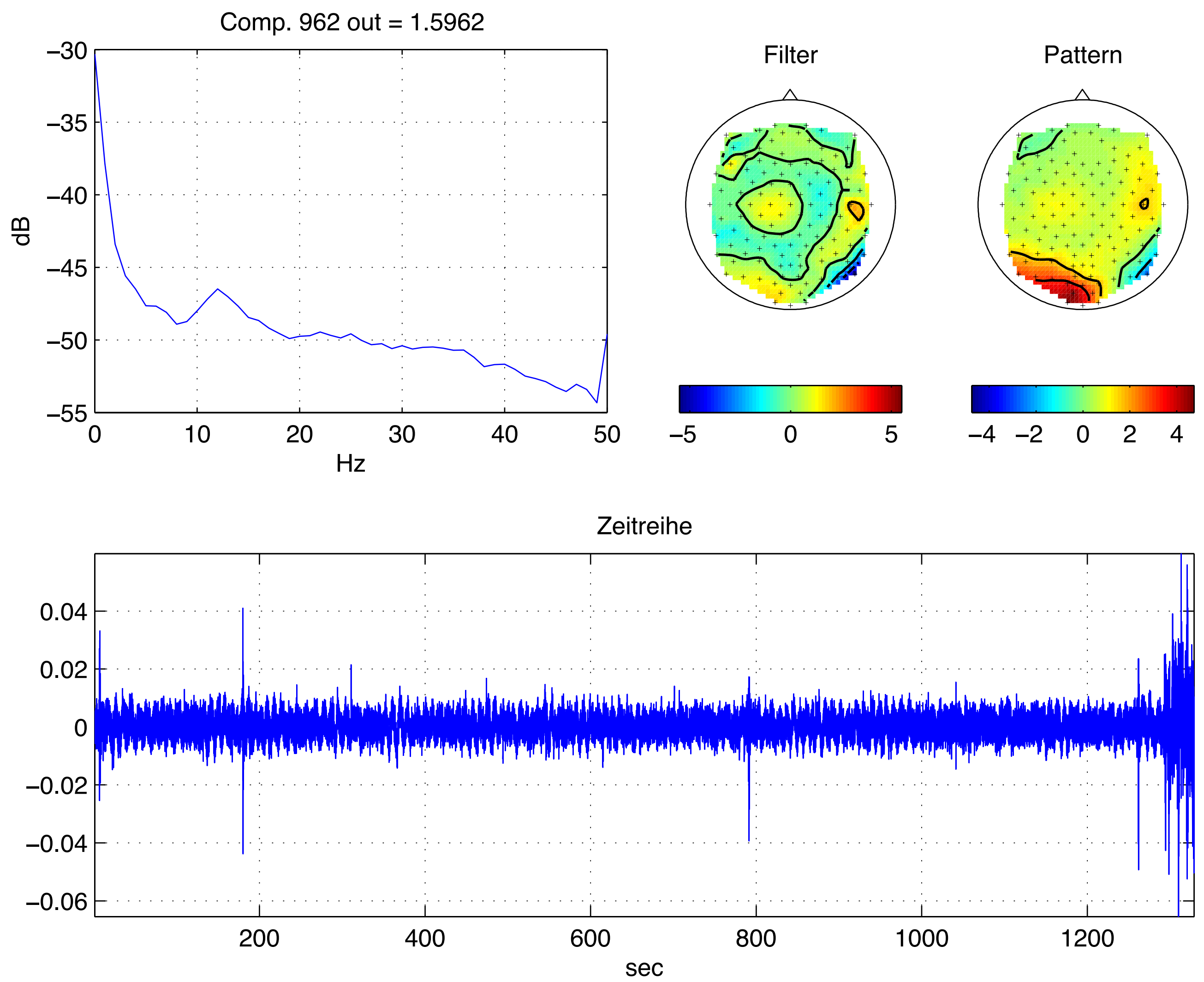
Comp. 962 out = 1.5962
dB
Hz
−30
−35
−40
−45
−50
−55
0
10
20
30
40
50
Filter
Pattern
−5
0
5
−4
−2
0
2
4
Zeitreihe
0.04
0.02
0
−0.02
−0.04
−0.06
200
400
600
800
1000
1200
sec

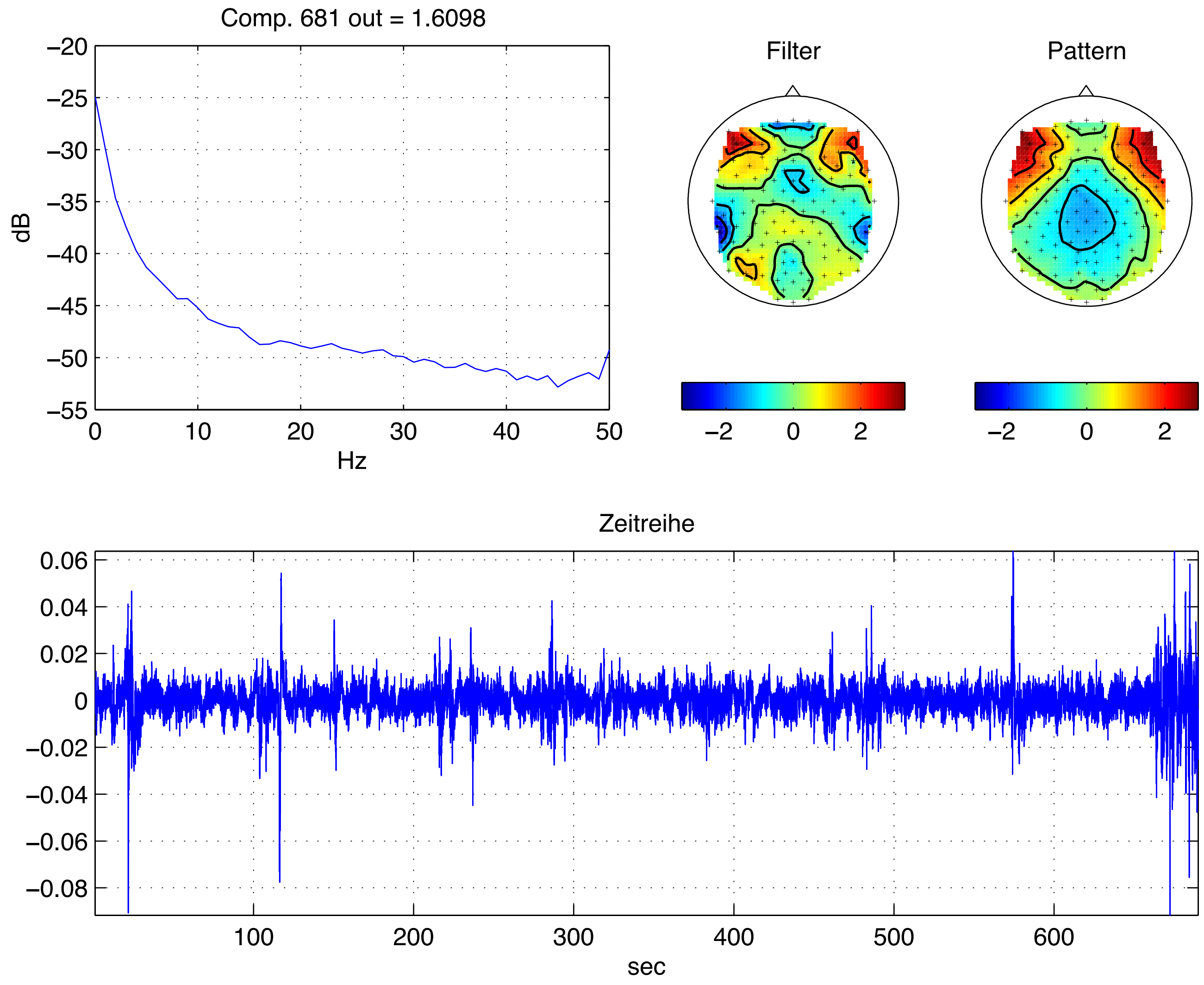

Comp. 681 out = 1.6098
dB
Hz
Filter
Pattern
Zeitreihe
sec

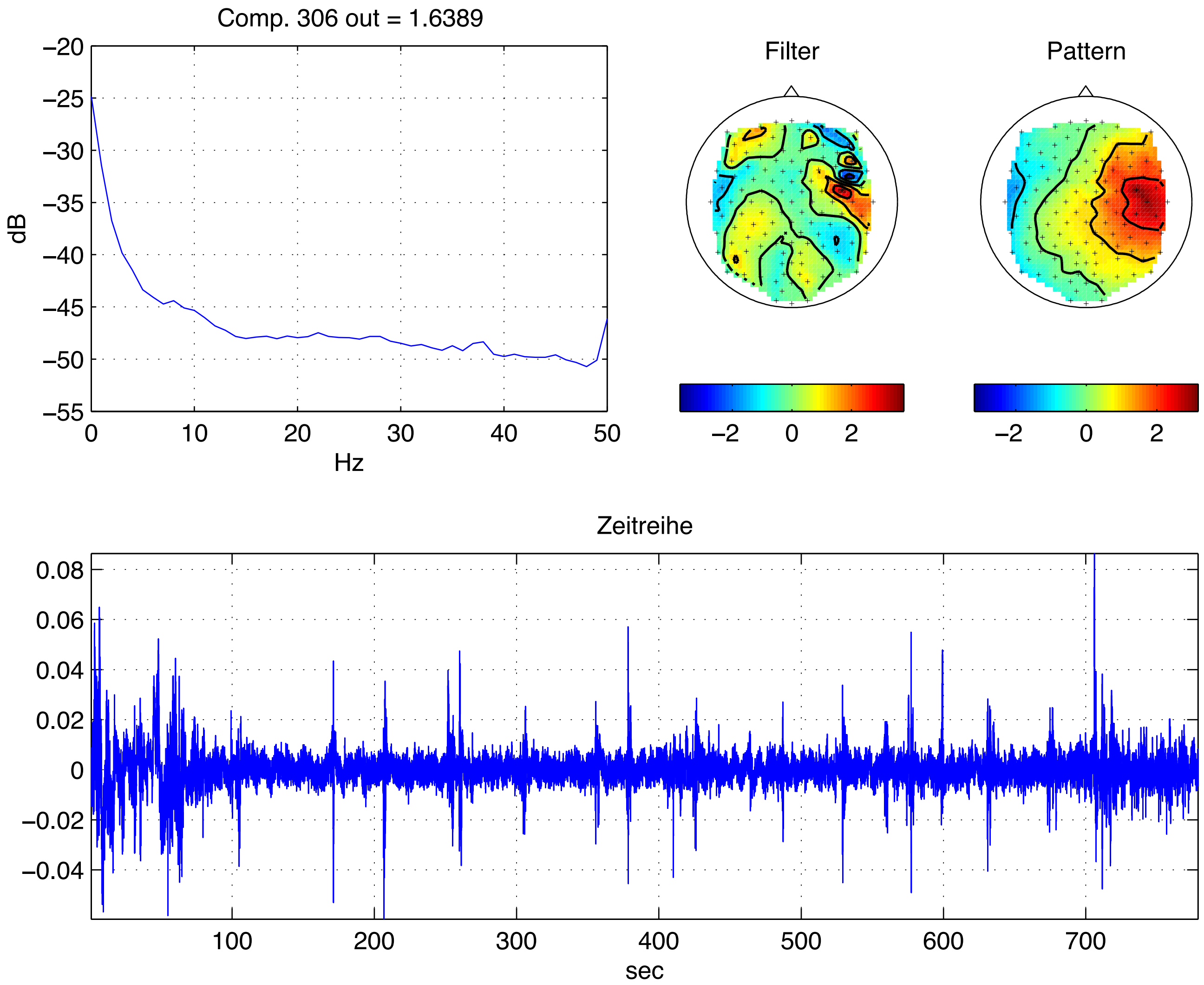

Comp. 306 out = 1.6389
dB
Hz
Filter
Pattern
Zeitreihe
sec

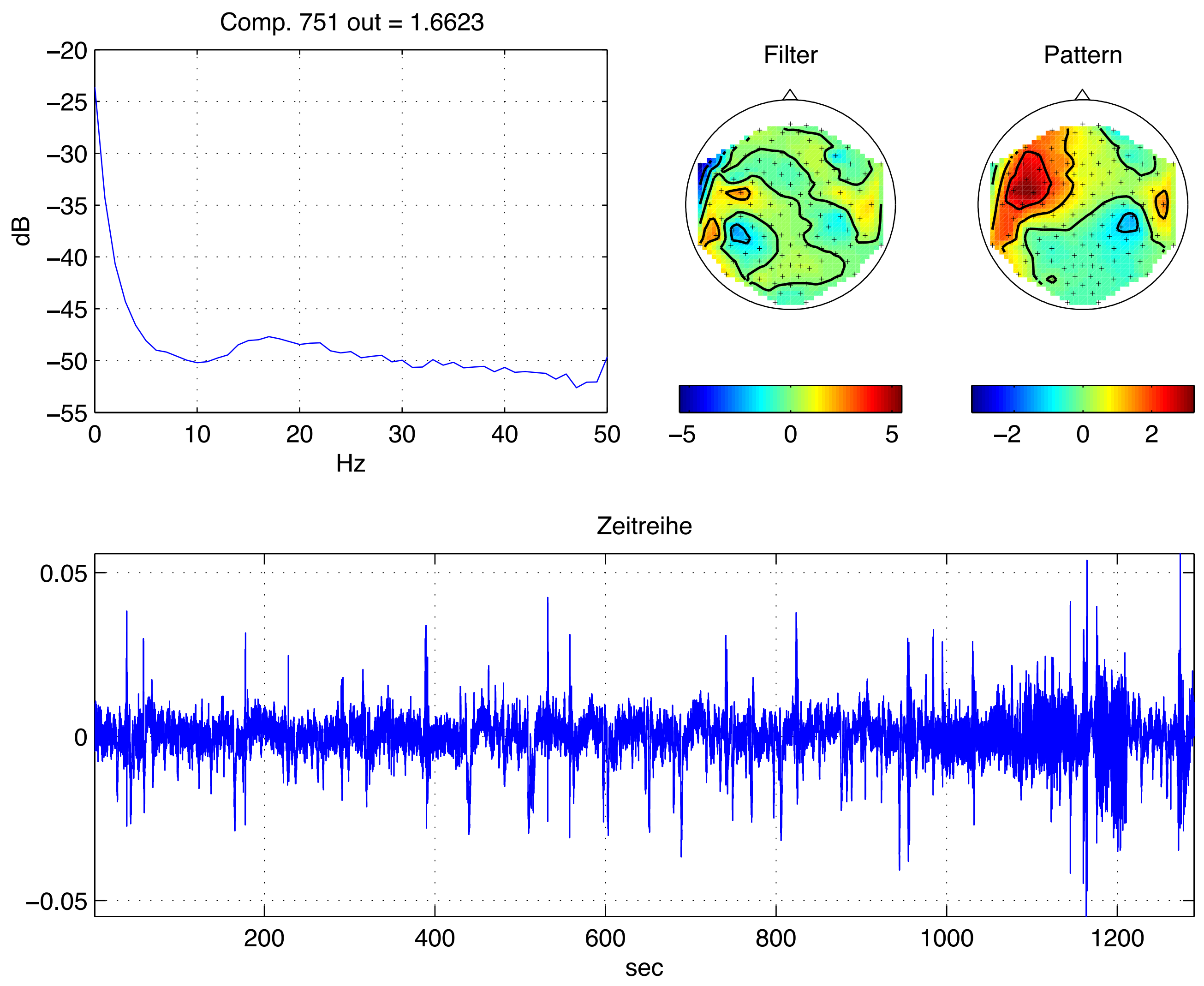

Comp. 751 out = 1.6623
dB
Hz
Filter
Pattern
Zeitreihe
sec

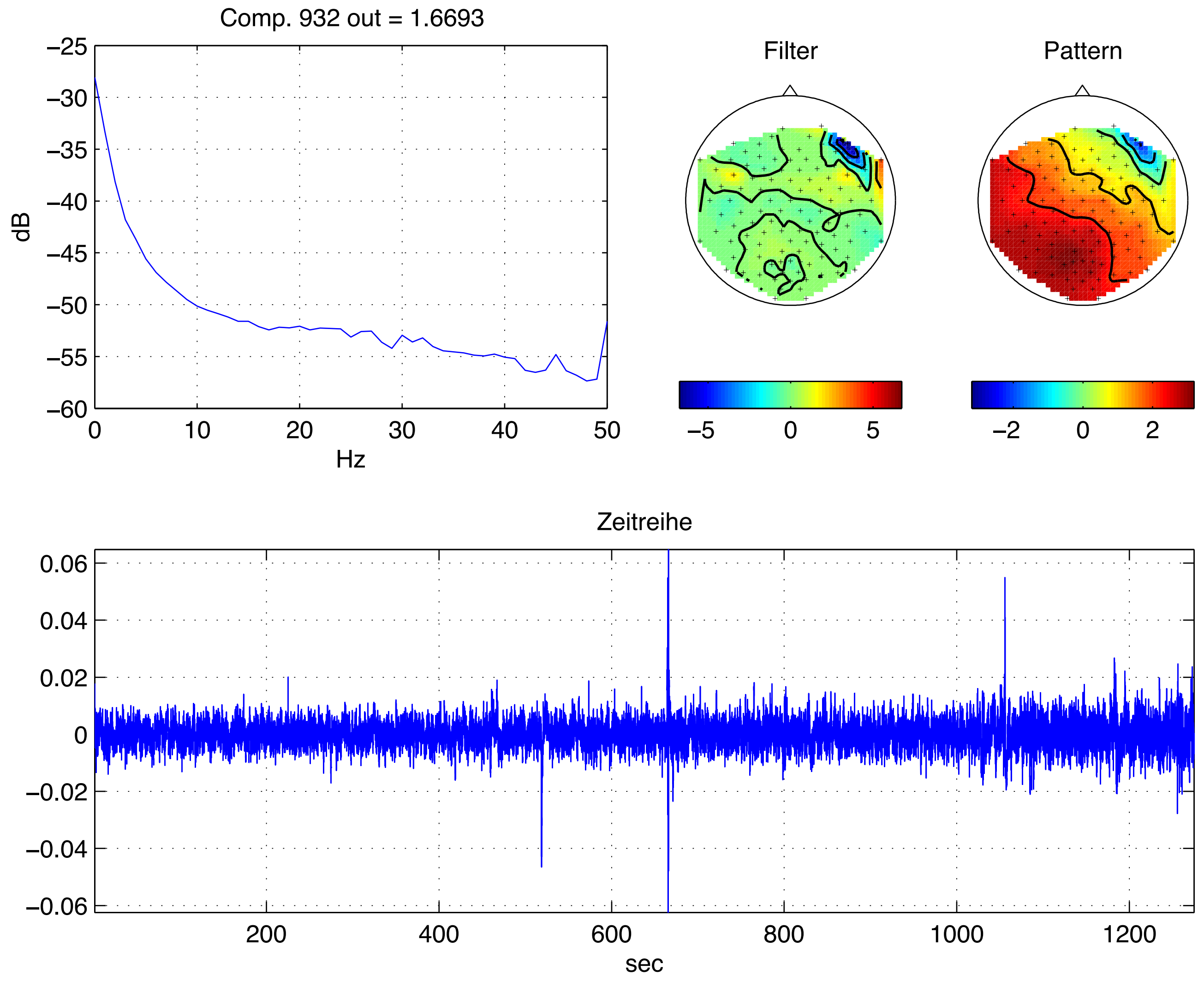
Comp. 932 out = 1.6693
dB
Hz
Filter
Pattern
Zeitreihe
sec

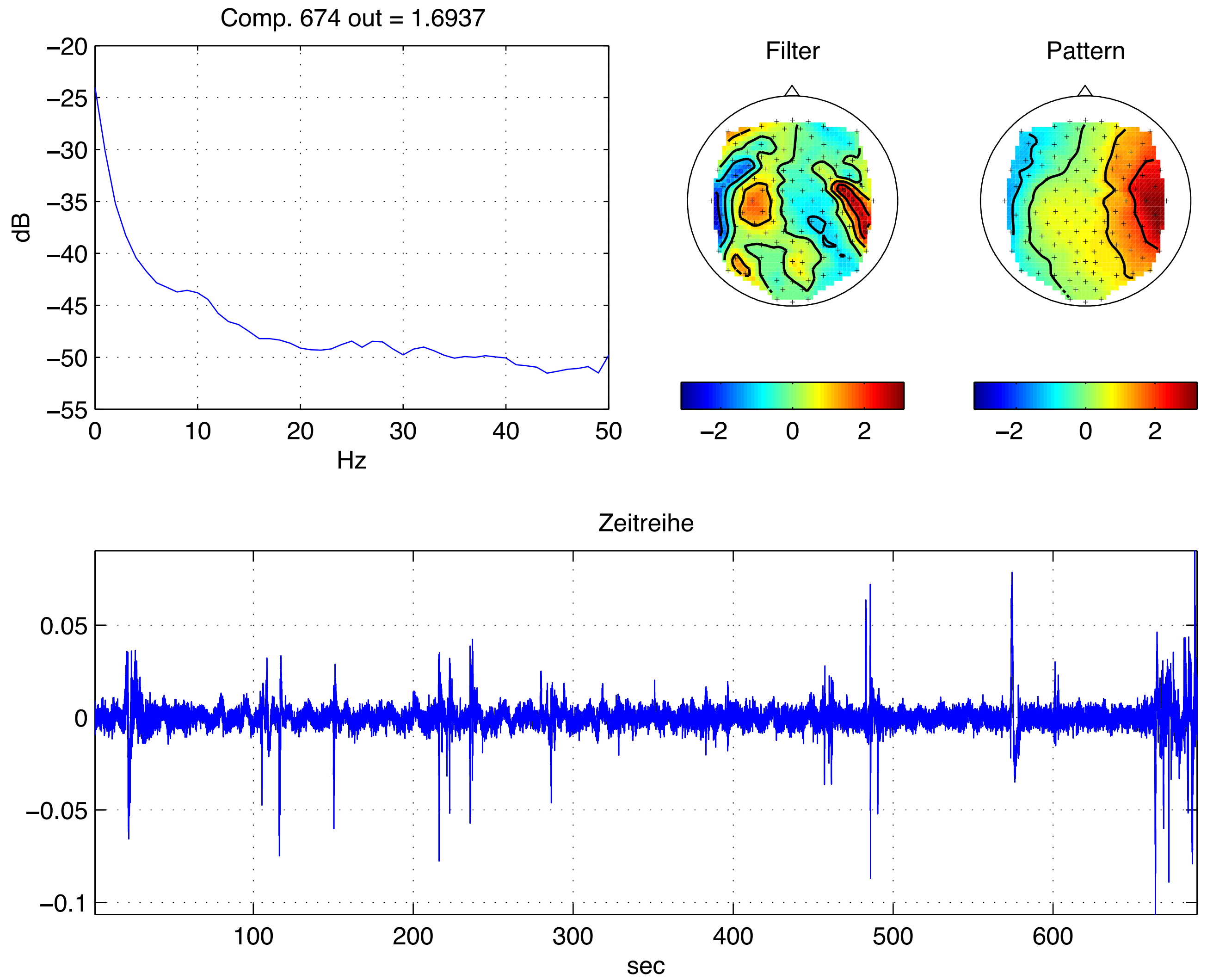
Comp. 674 out = 1.6937
dB
Hz
Filter
Pattern
Zeitreihe
sec

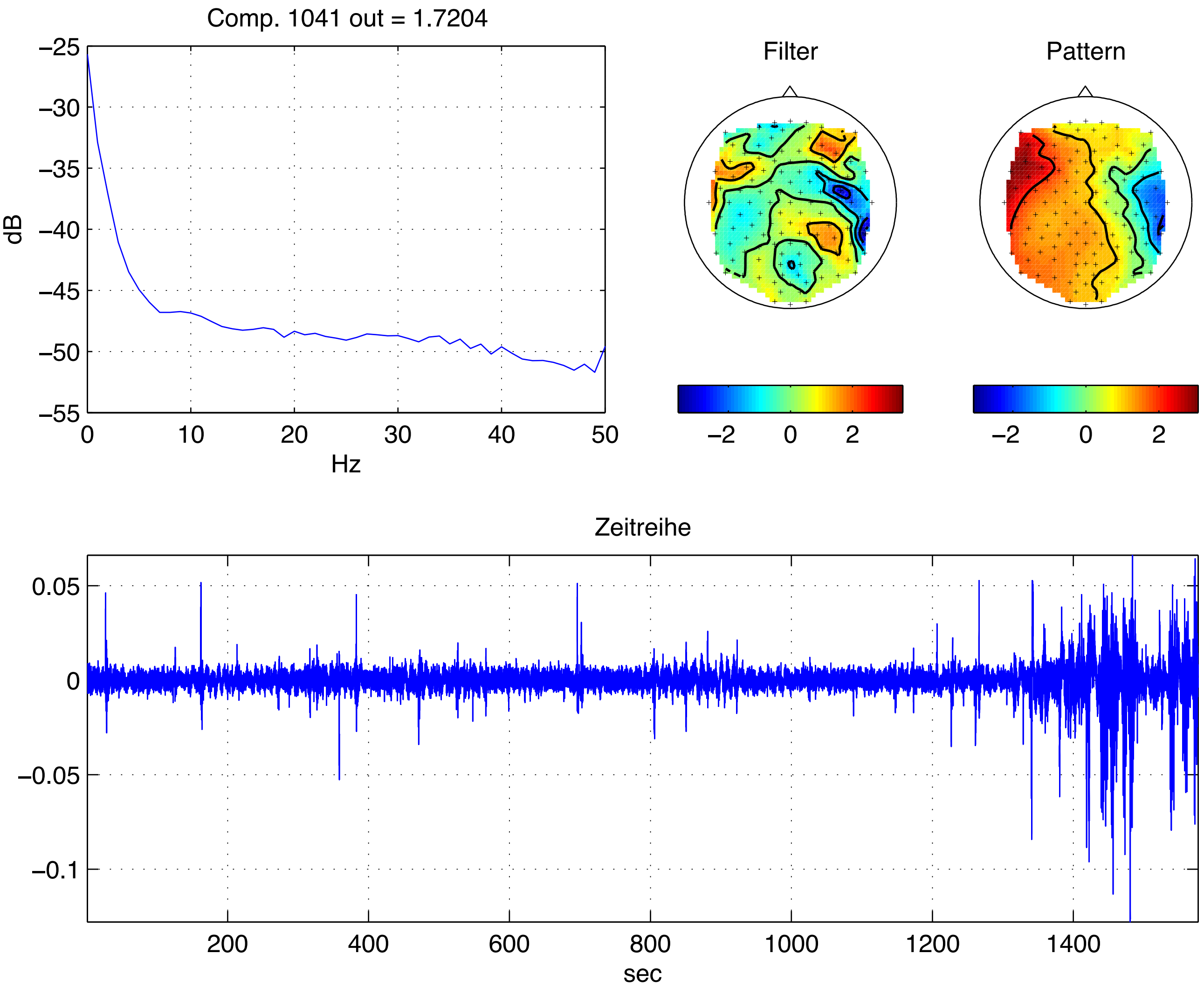

Comp. 1041 out = 1.7204
dB
Hz
Filter
Pattern
Zeitreihe
sec

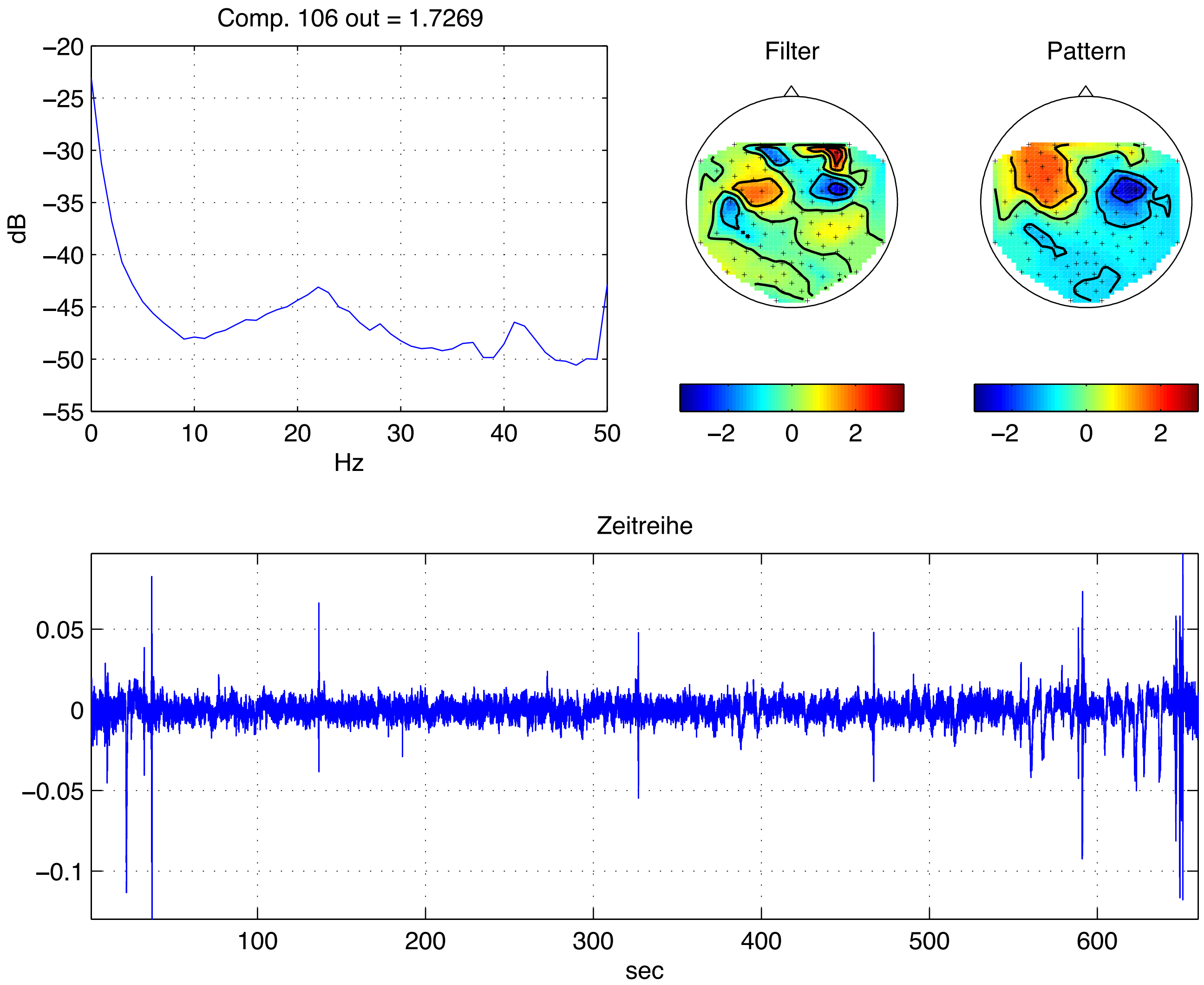

Comp. 106 out = 1.7269
dB
Hz
Filter
Pattern
Zeitreihe
sec

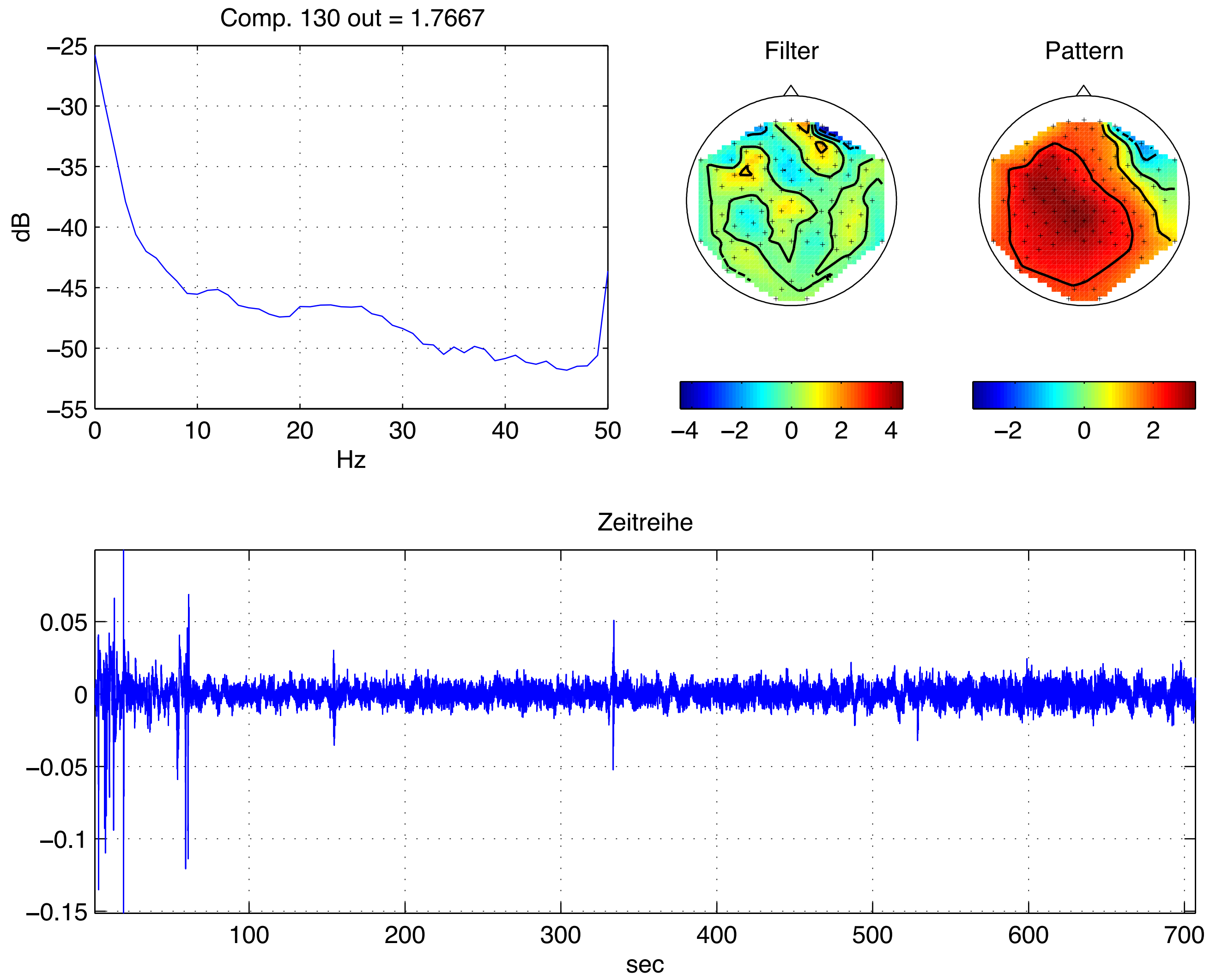

Comp. 130 out = 1.7667
dB
Hz
−25
−30
−35
−40
−45
−50
−55
0
10
20
30
40
50
Filter
Pattern
−4
−2
0
2
4
Zeitreihe
sec
0.05
0
−0.05
−0.1
−0.15
100
200
300
400
500
600
700

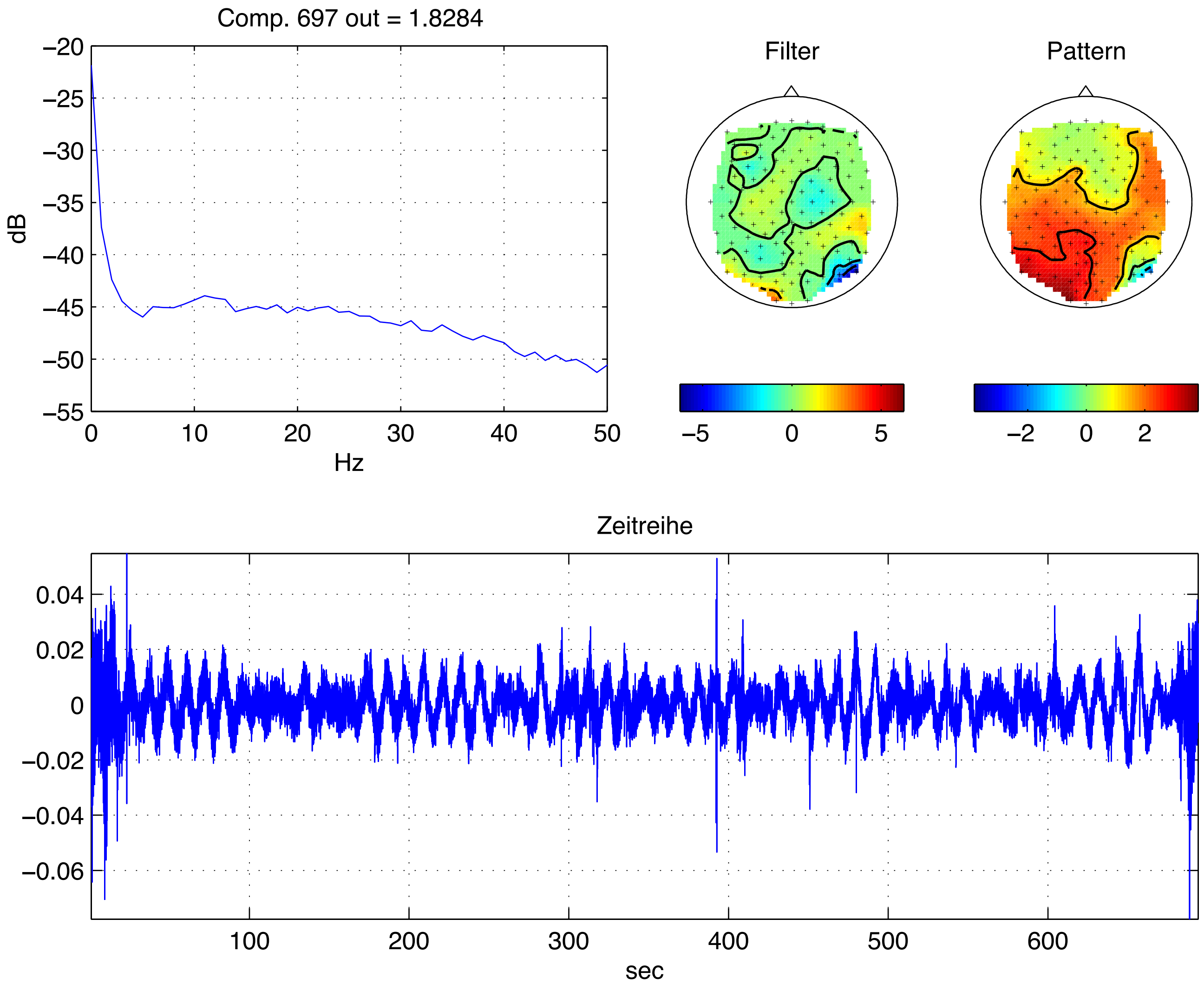

Comp. 697 out = 1.8284
dB
−20
−25
−30
−35
−40
−45
−50
−55
0
10
20
30
40
50
Hz
Filter
Pattern
−5
0
5
−2
0
2
Zeitreihe
0.04
0.02
0
−0.02
−0.04
−0.06
100
200
300
400
500
600
sec

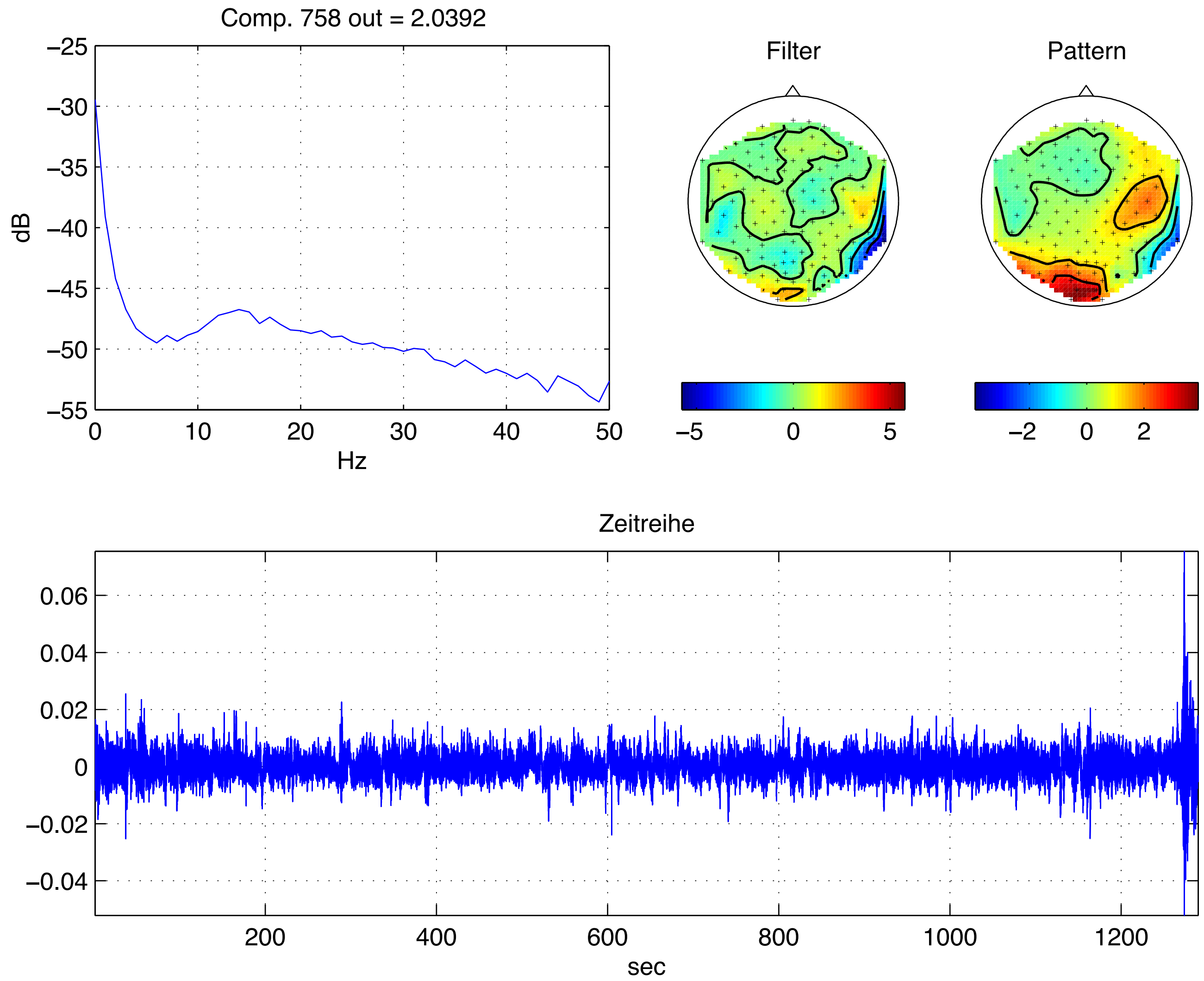

Comp. 758 out = 2.0392
dB
Hz
Filter
Pattern
Zeitreihe
sec

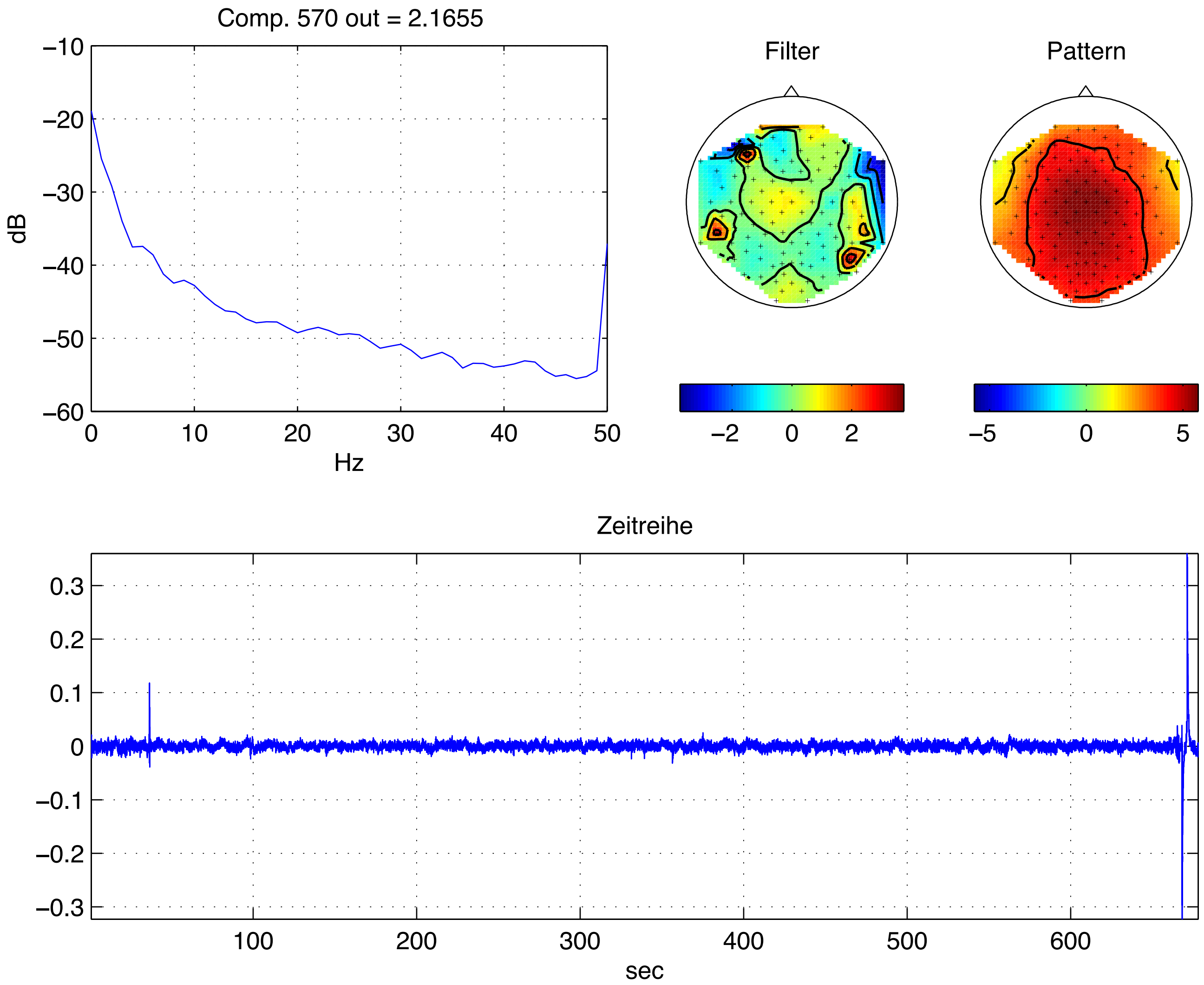

Comp. 570 out = 2.1655
dB
Hz
Filter
Pattern
Zeitreihe
sec

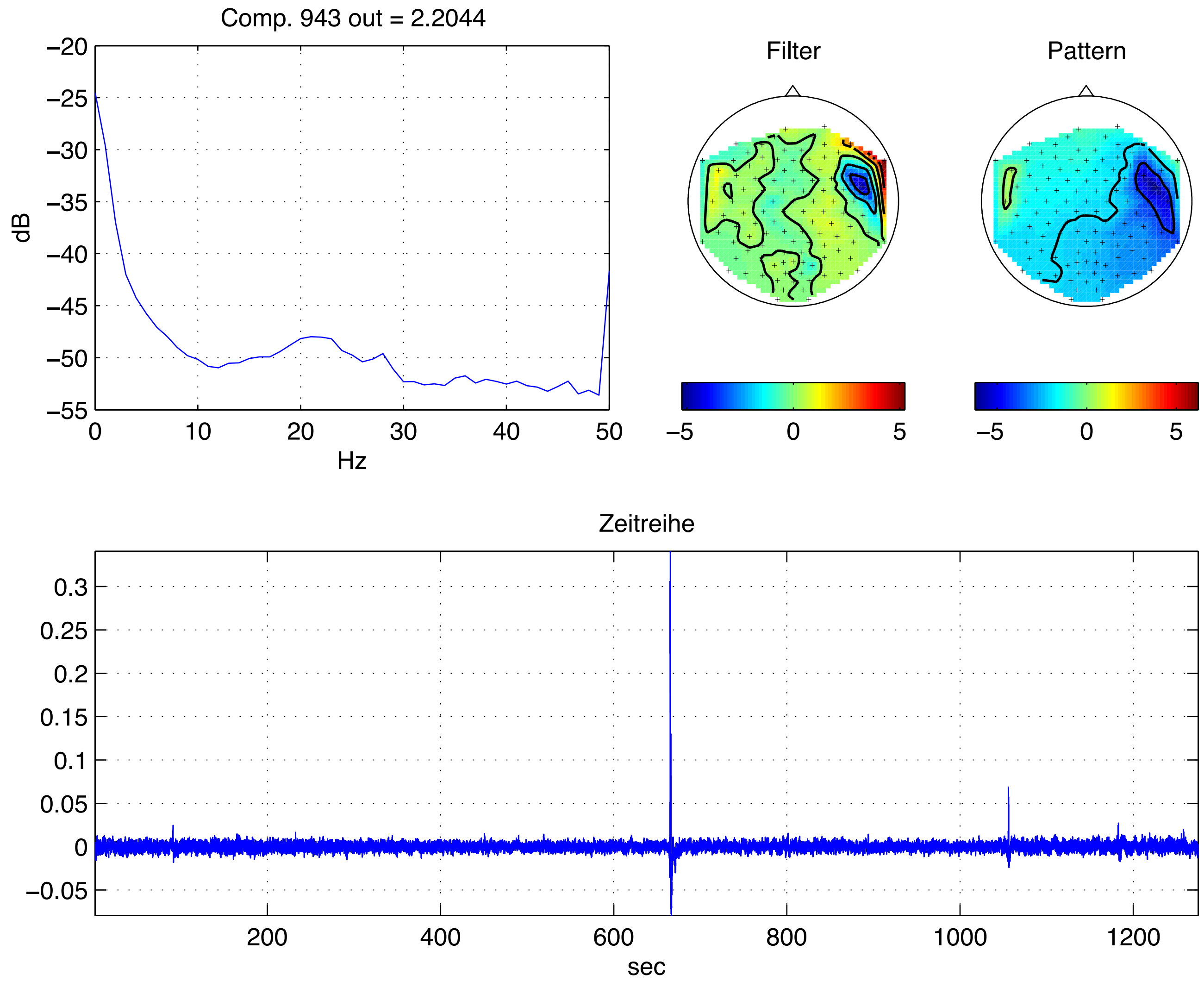

Comp. 943 out = 2.2044
dB
Hz
Filter
Pattern
Zeitreihe
sec

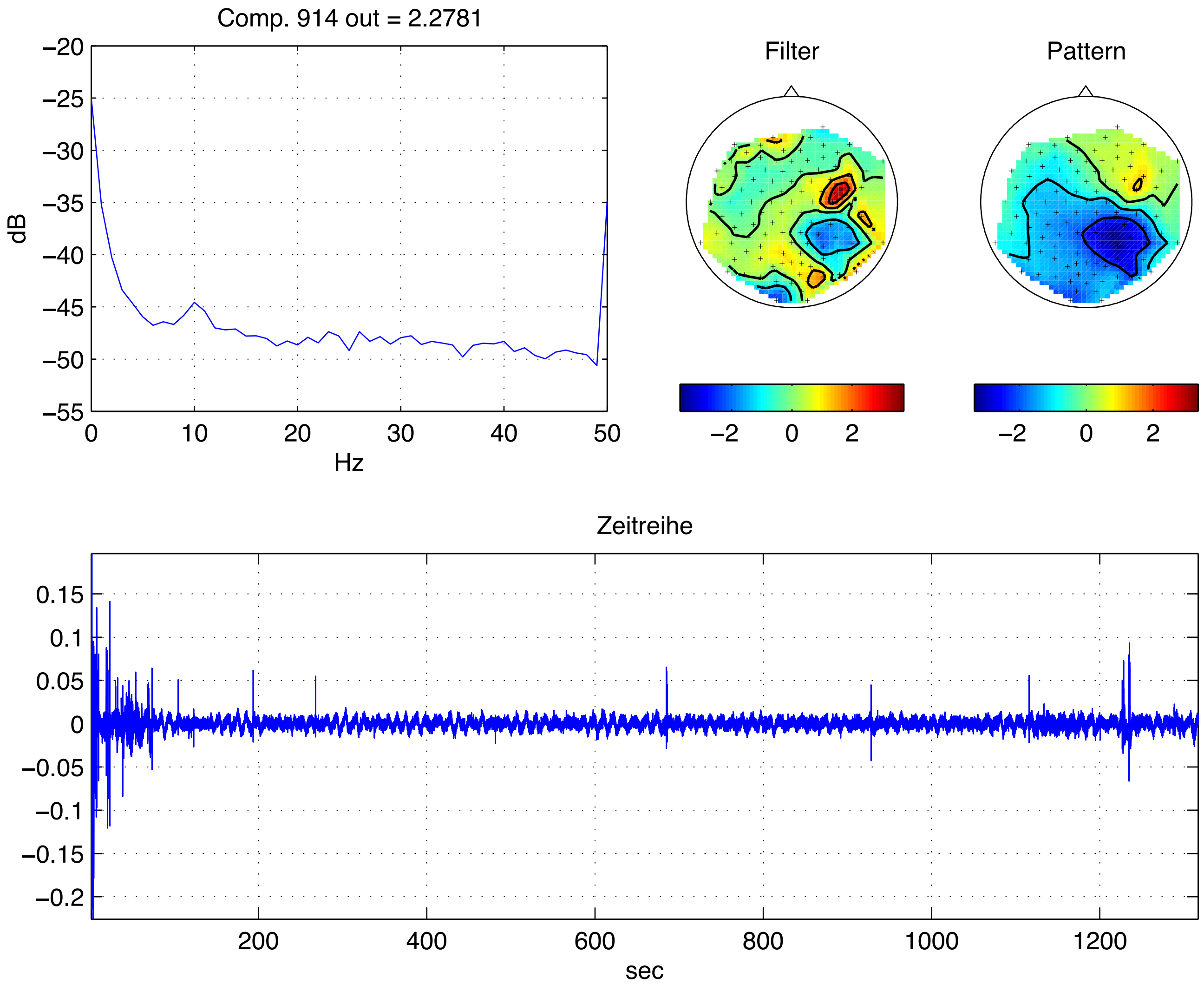

Comp. 914 out = 2.2781
dB
Hz
Filter
Pattern
Zeitreihe
sec

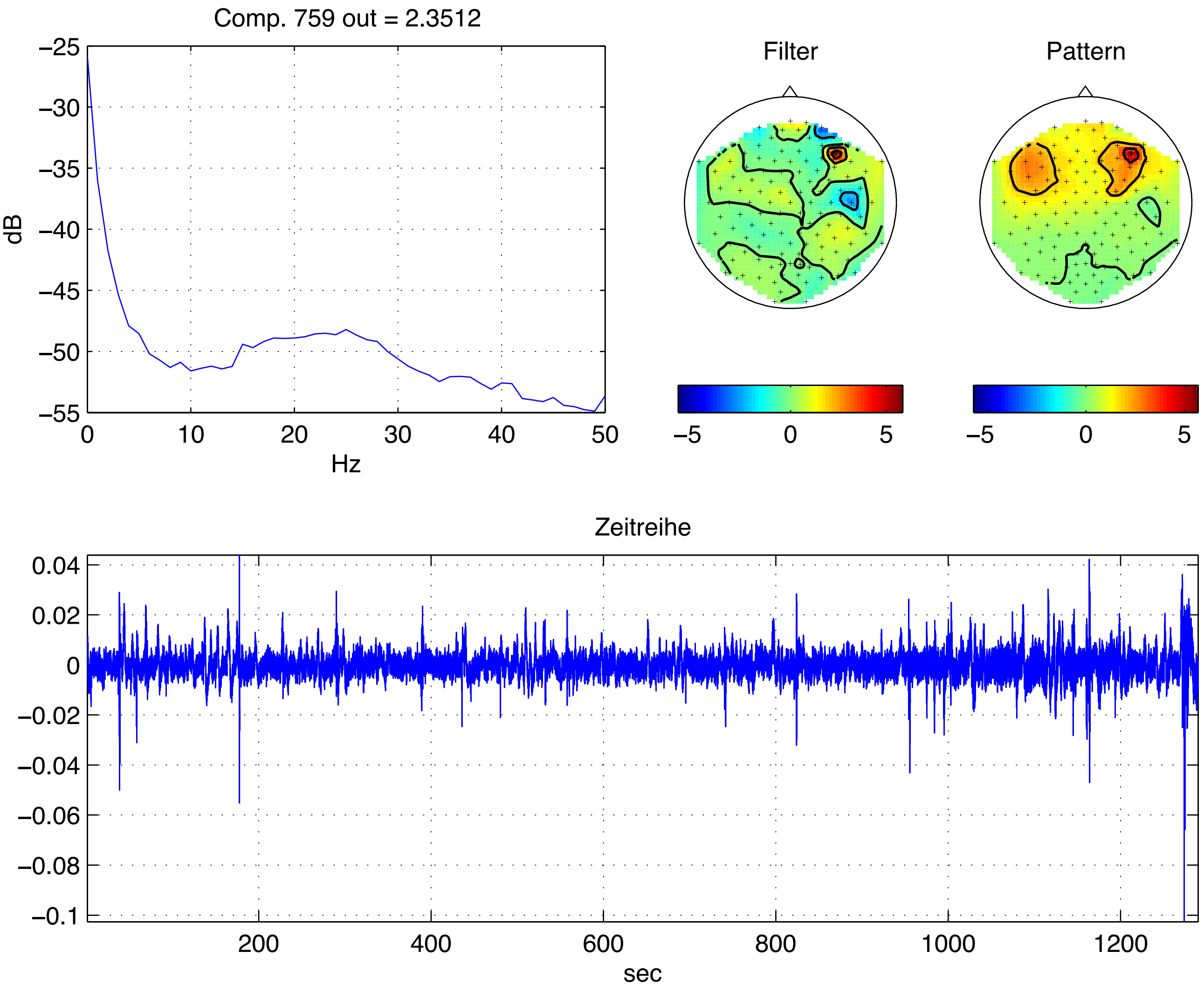
Comp. 759 out = 2.3512
dB
Hz
Filter
Pattern
Zeitreihe
sec

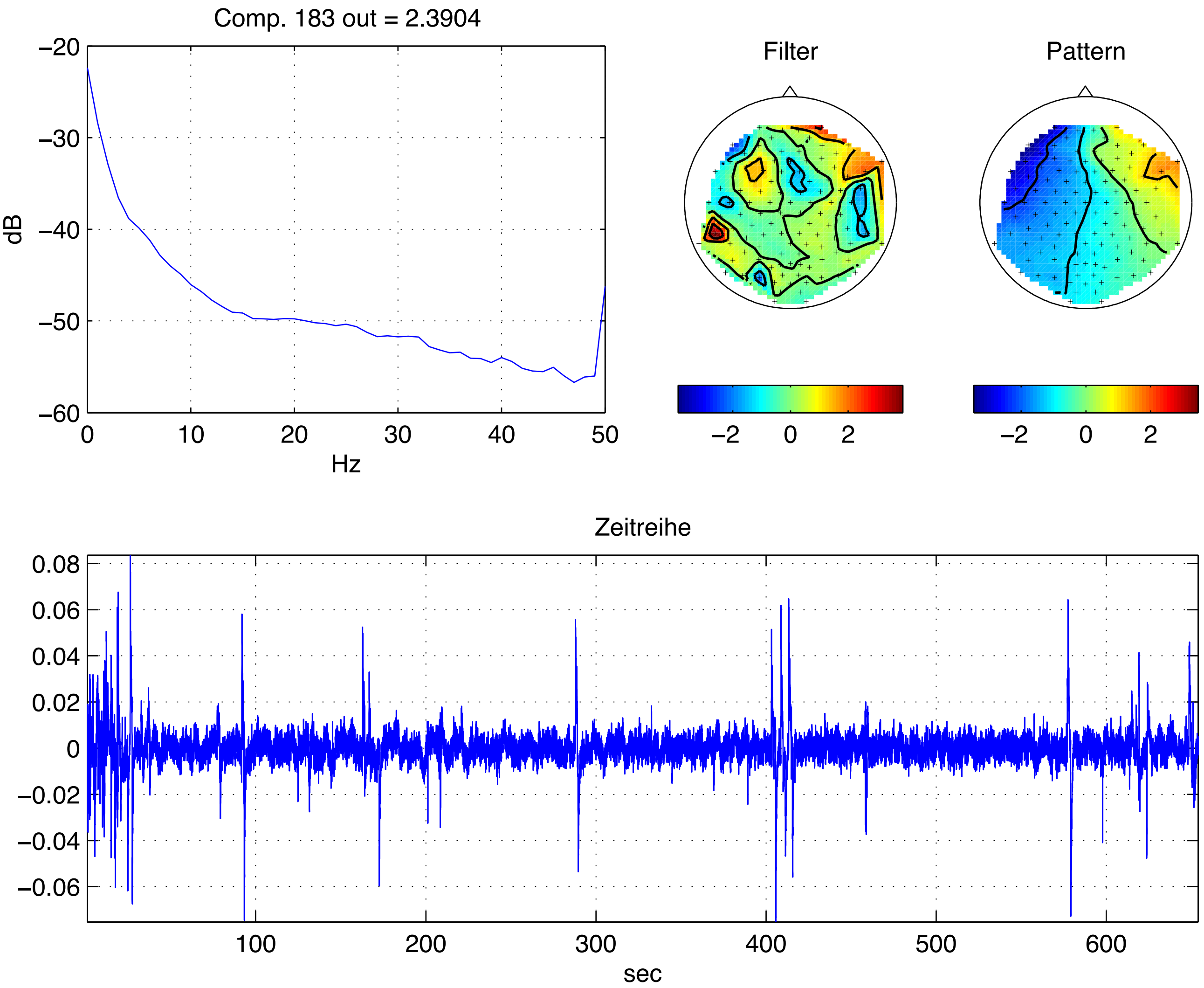

Comp. 183 out = 2.3904
dB
Hz
Filter
Pattern
Zeitreihe
sec

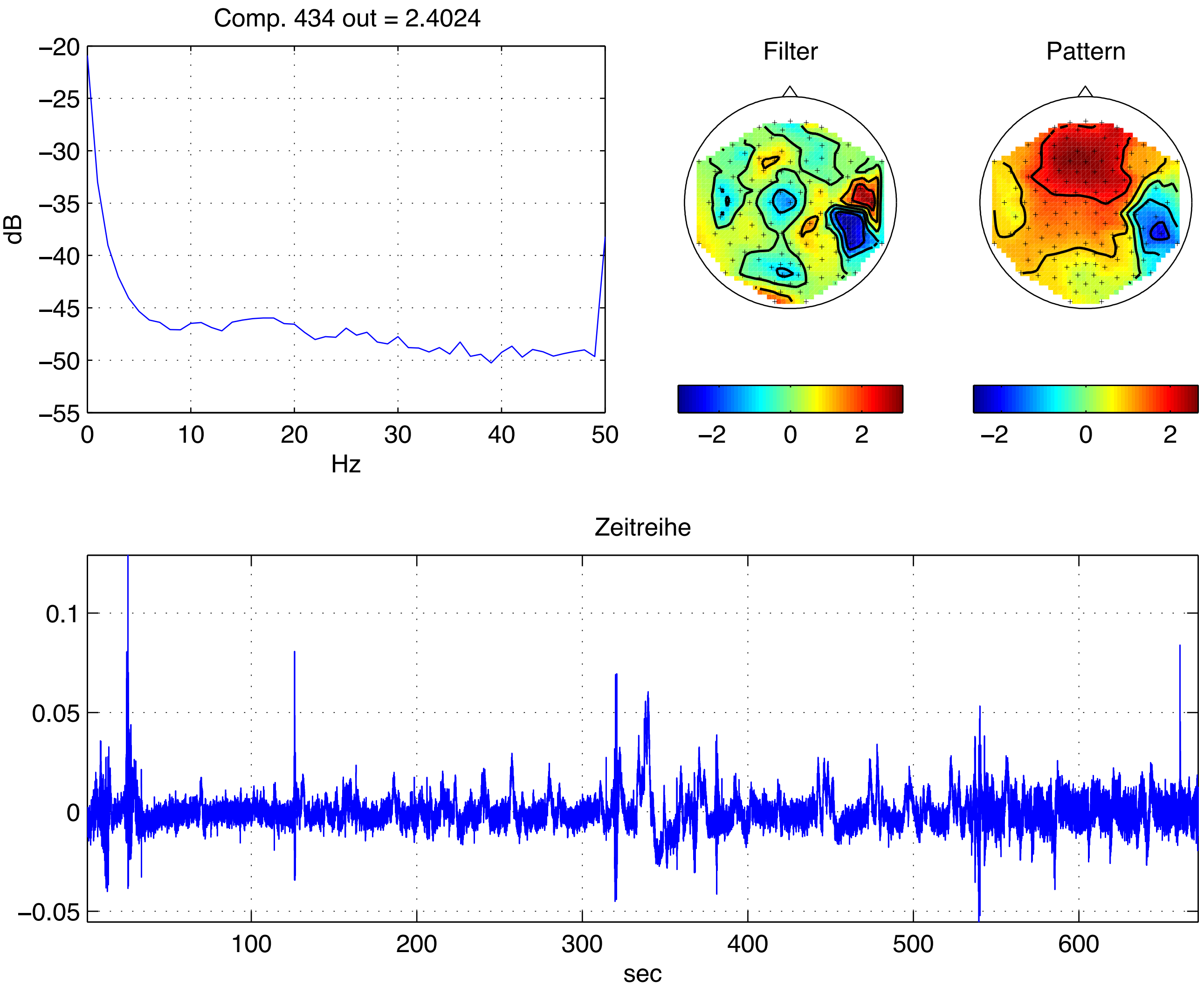

Comp. 434 out = 2.4024
dB
−20
−25
−30
−35
−40
−45
−50
−55
0
10
20
30
40
50
Hz
Filter
Pattern
−2
0
2
−2
0
2
Zeitreihe
0.1
0.05
0
−0.05
100
200
300
400
500
600
sec

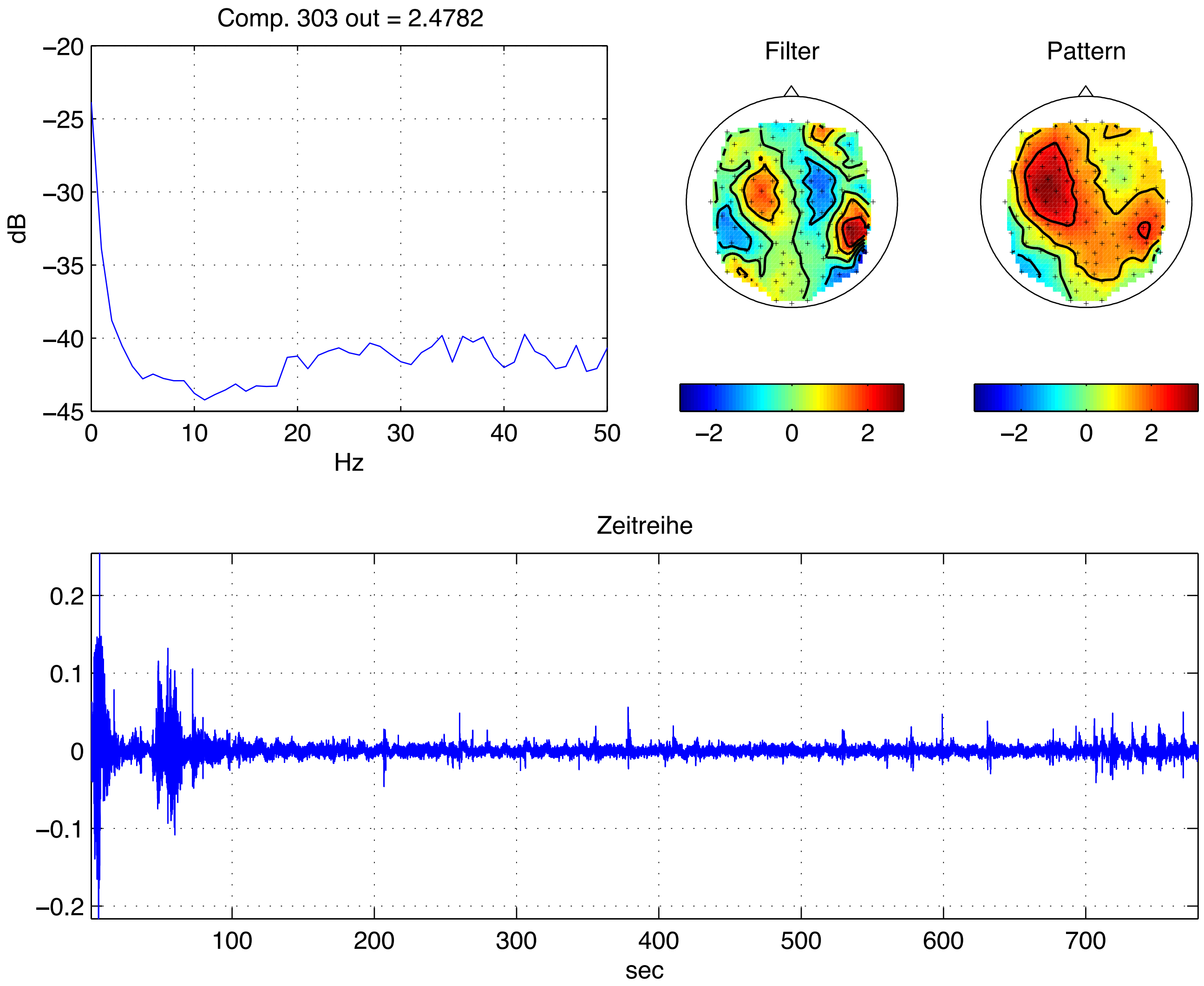
Comp. 303 out = 2.4782
dB
Hz
Filter
Pattern
Zeitreihe
sec

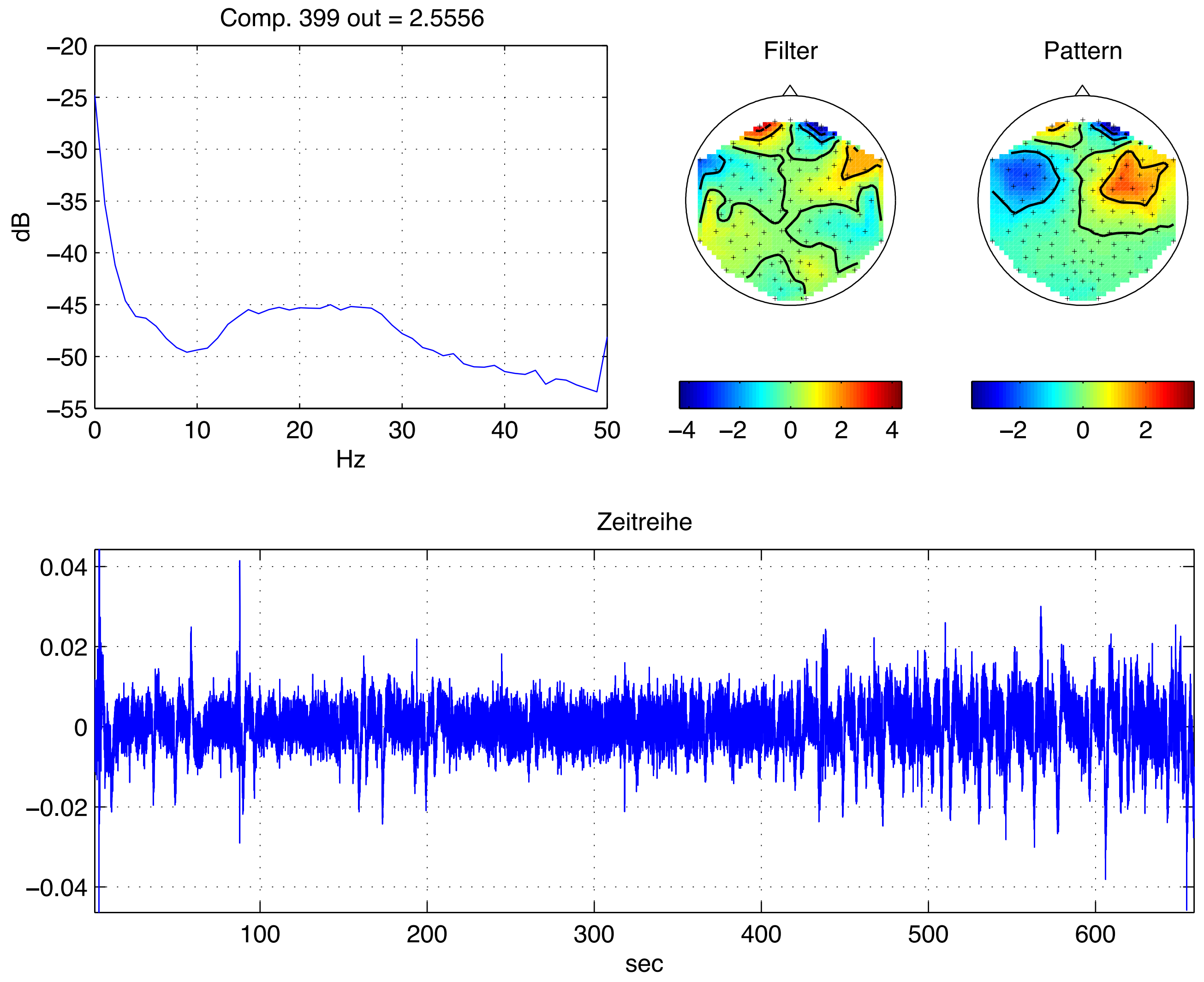
Comp. 399 out = 2.5556
dB
Hz
Filter
Pattern
Zeitreihe
sec

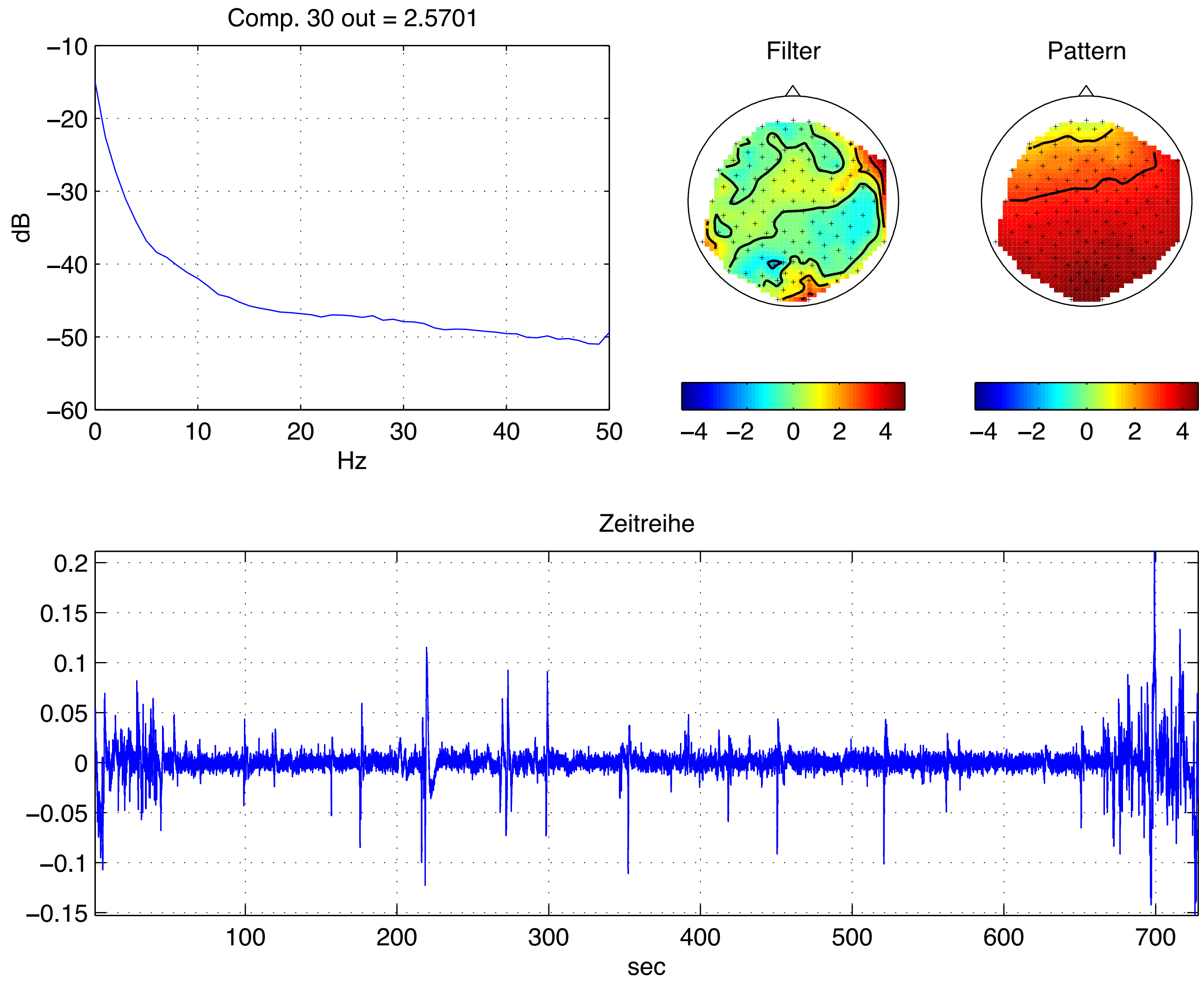

Comp. 30 out = 2.5701
dB
Hz
−10
−20
−30
−40
−50
−60
0
10
20
30
40
50
Filter
Pattern
−4
−2
0
2
4
Zeitreihe
0.2
0.15
0.1
0.05
0
−0.05
−0.1
−0.15
100
200
300
400
500
600
700
sec

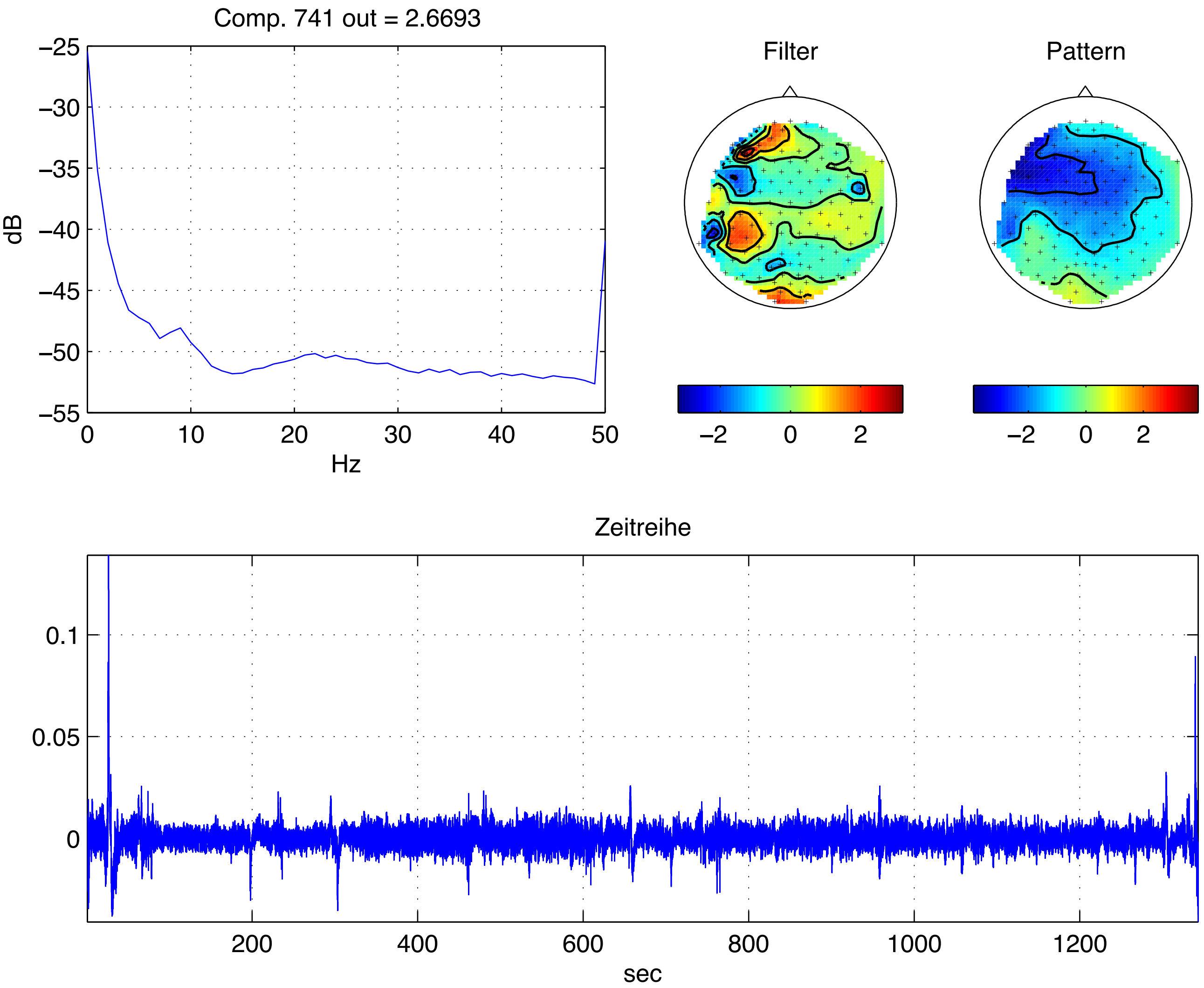
Comp. 741 out = 2.6693
dB
Hz
Filter
Pattern
Zeitreihe
sec

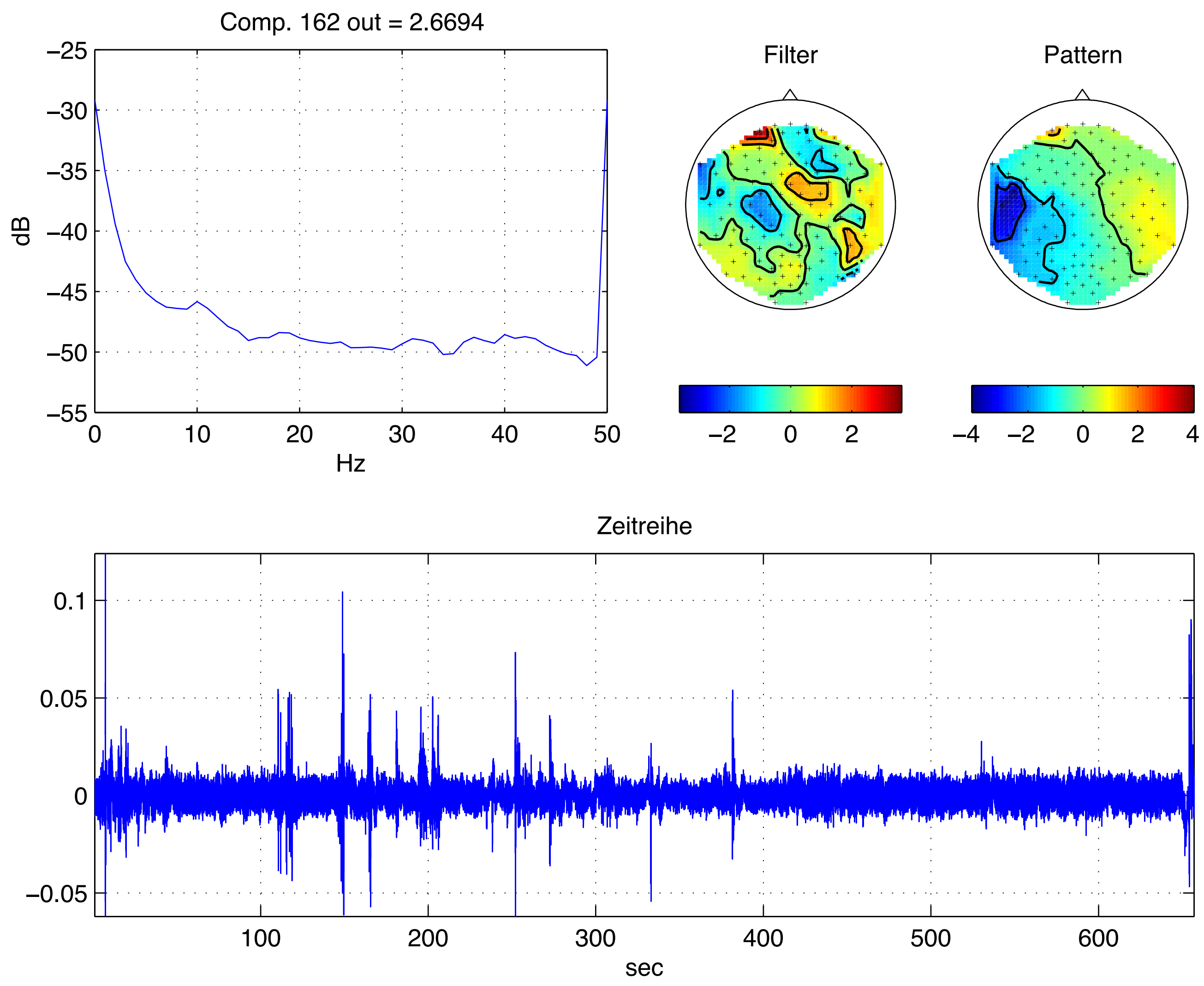

Comp. 162 out = 2.6694
dB
Hz
Filter
Pattern
Zeitreihe
sec

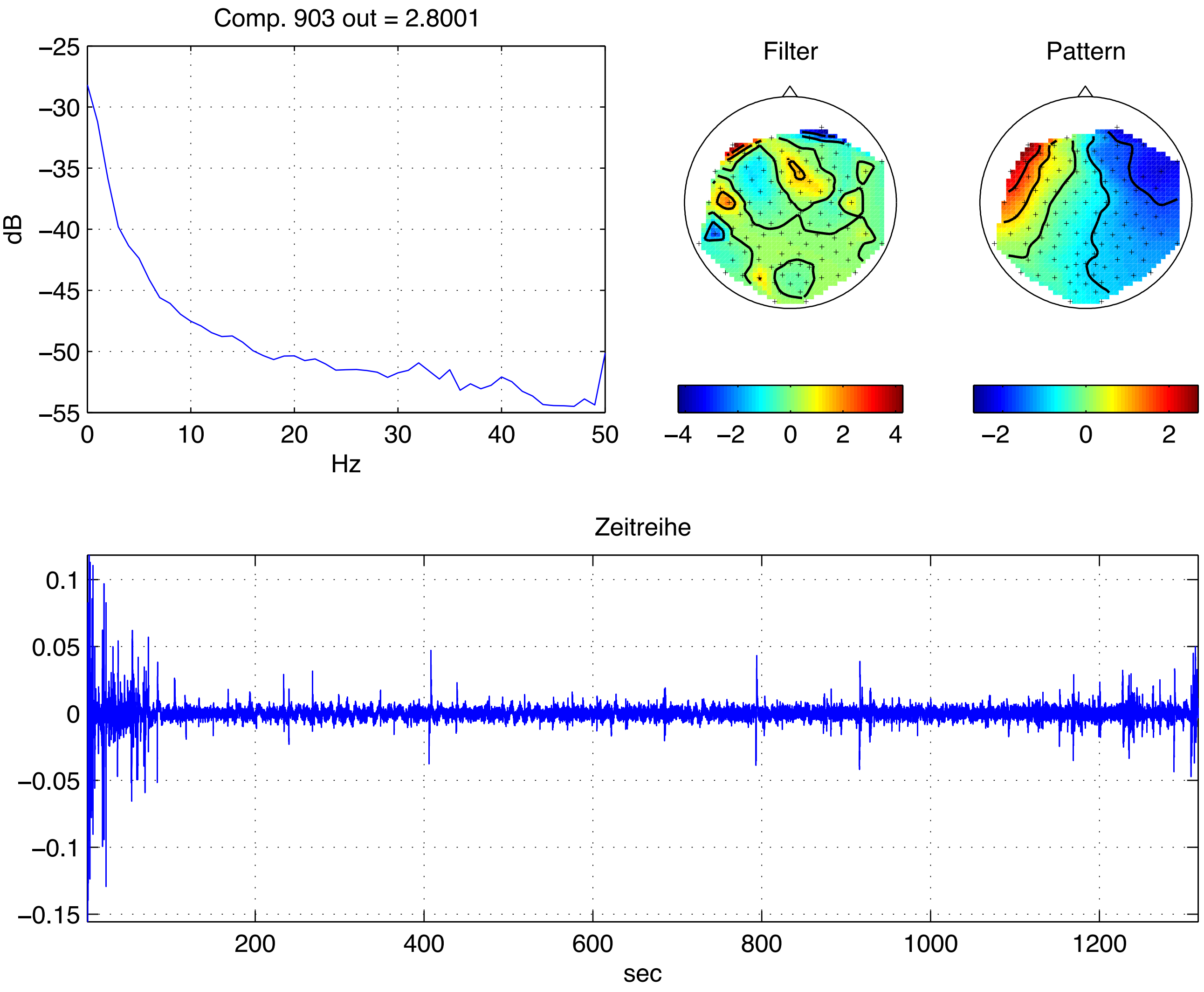

Comp. 903 out = 2.8001
dB
Hz
Filter
Pattern
Zeitreihe
sec

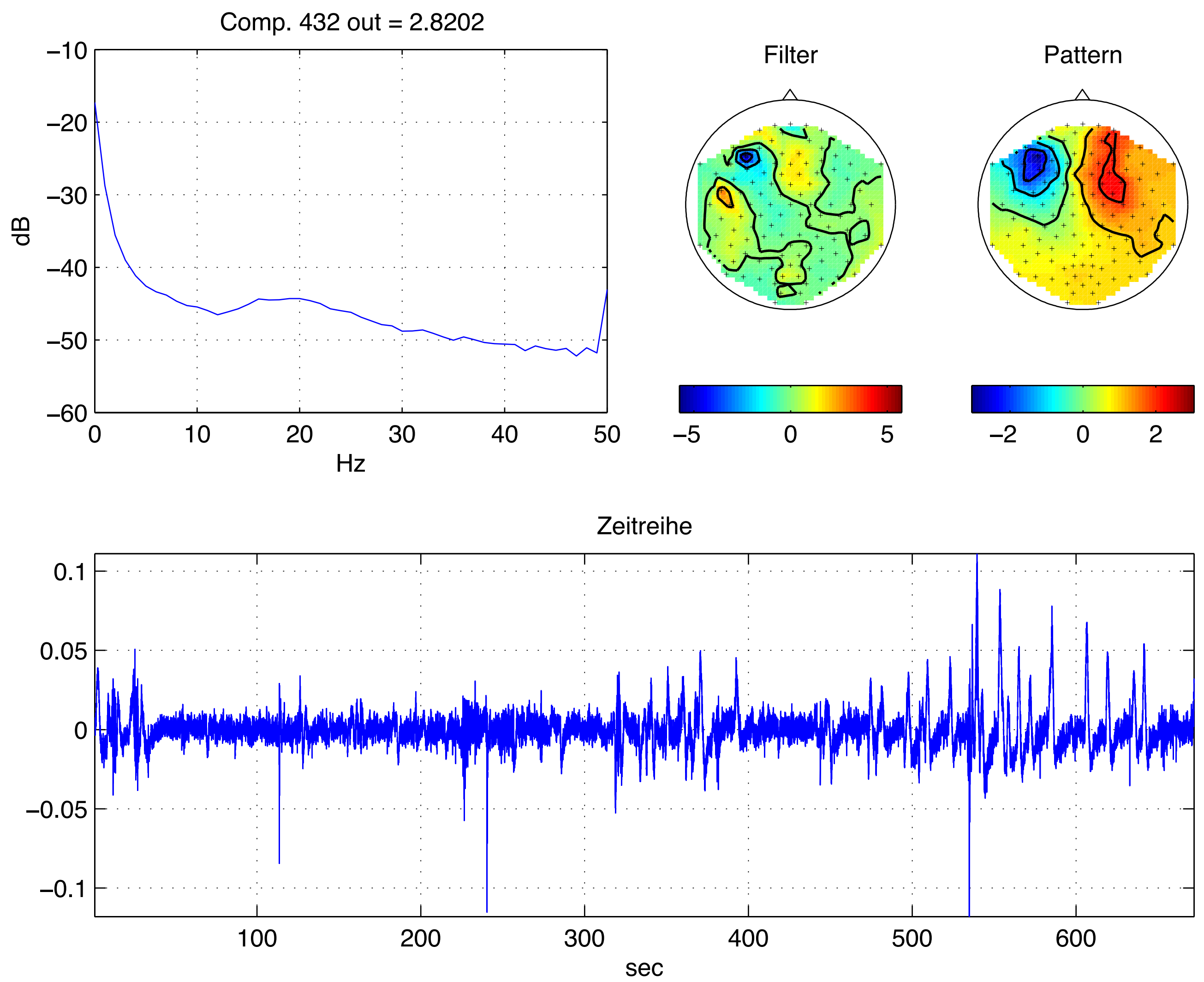

Comp. 432 out = 2.8202
dB
Hz
Filter
Pattern
Zeitreihe
sec

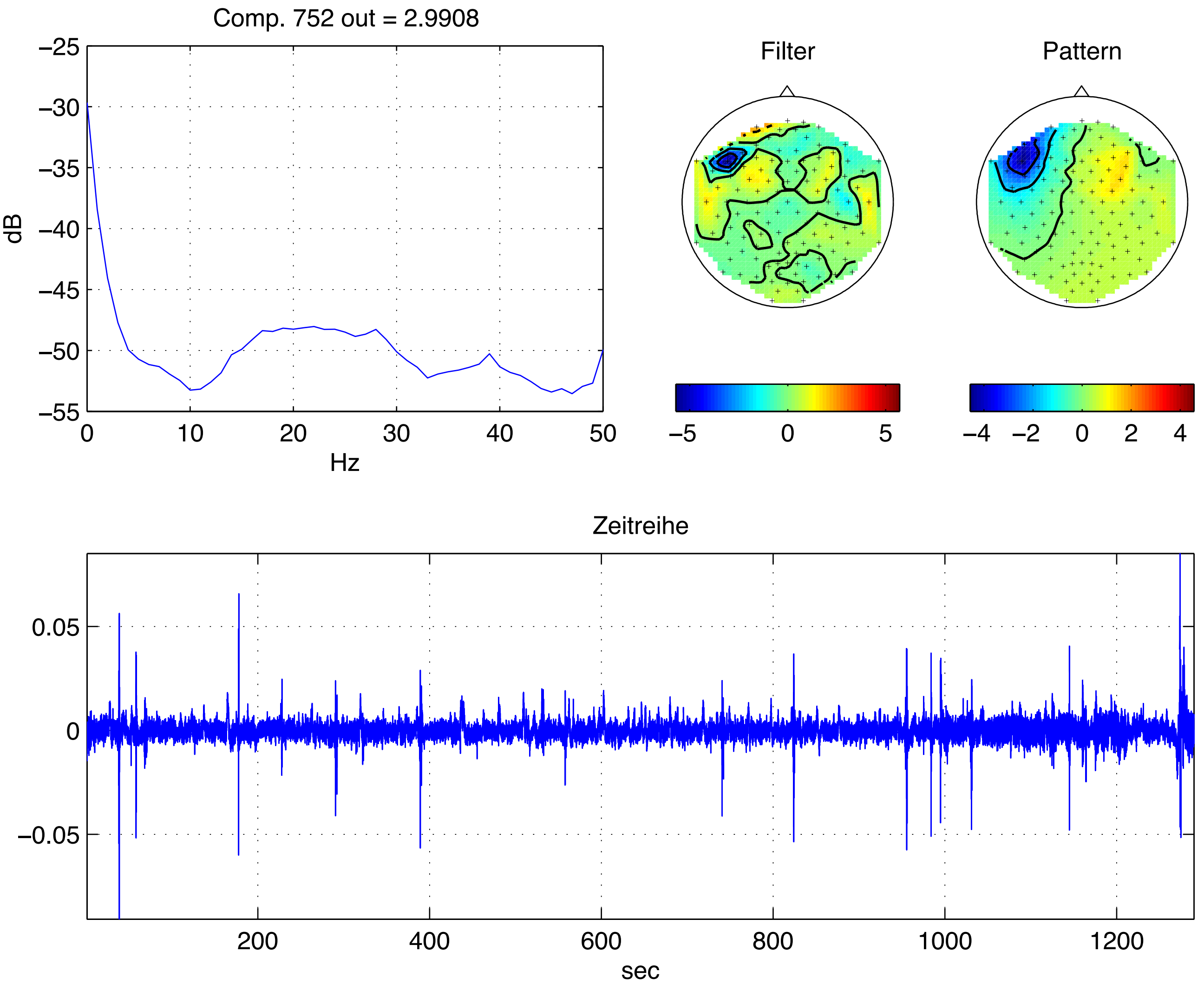

Comp. 752 out = 2.9908
dB
Hz
Filter
Pattern
Zeitreihe
sec

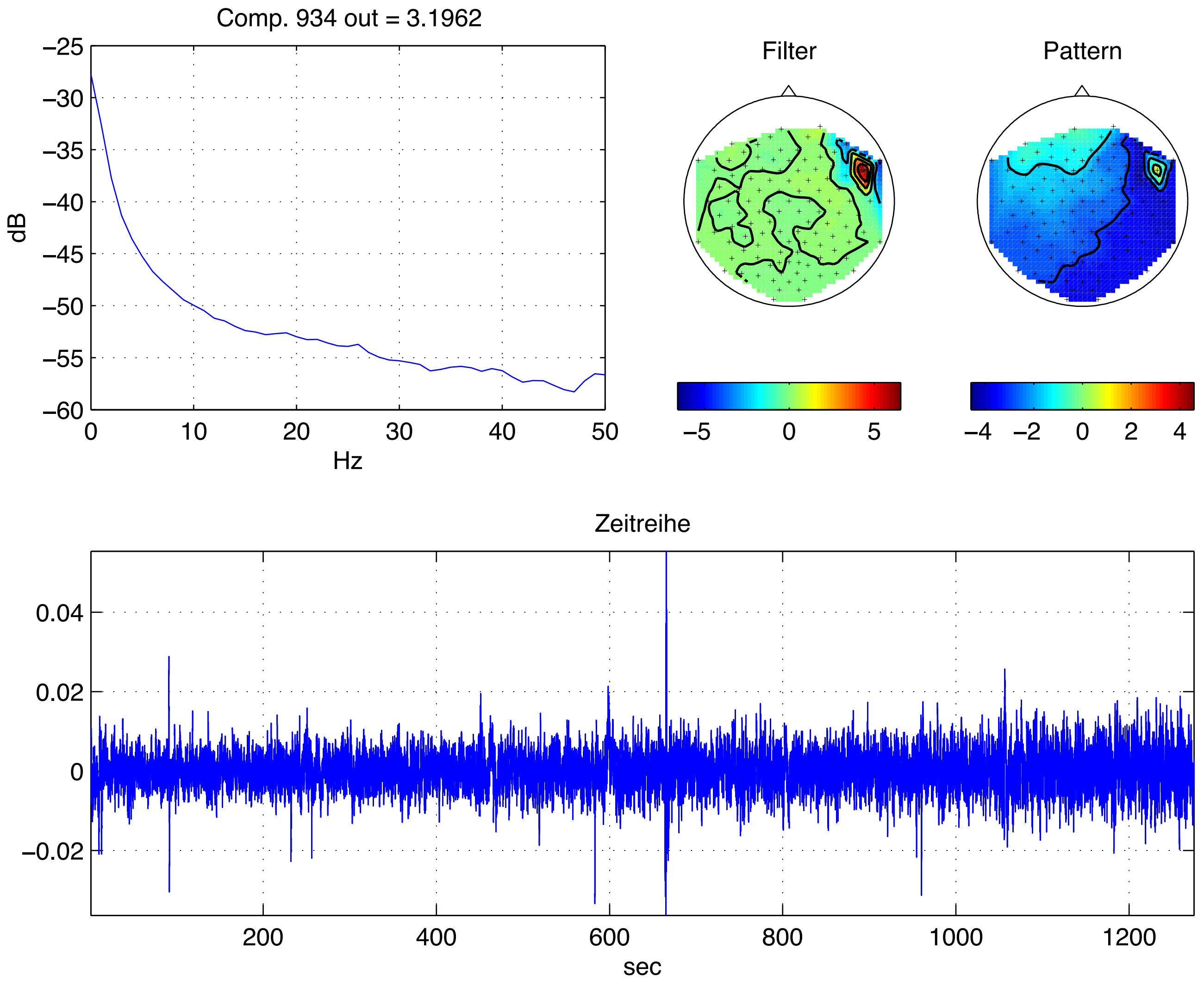

Comp. 934 out = 3.1962
dB
Hz
Filter
Pattern
Zeitreihe
sec

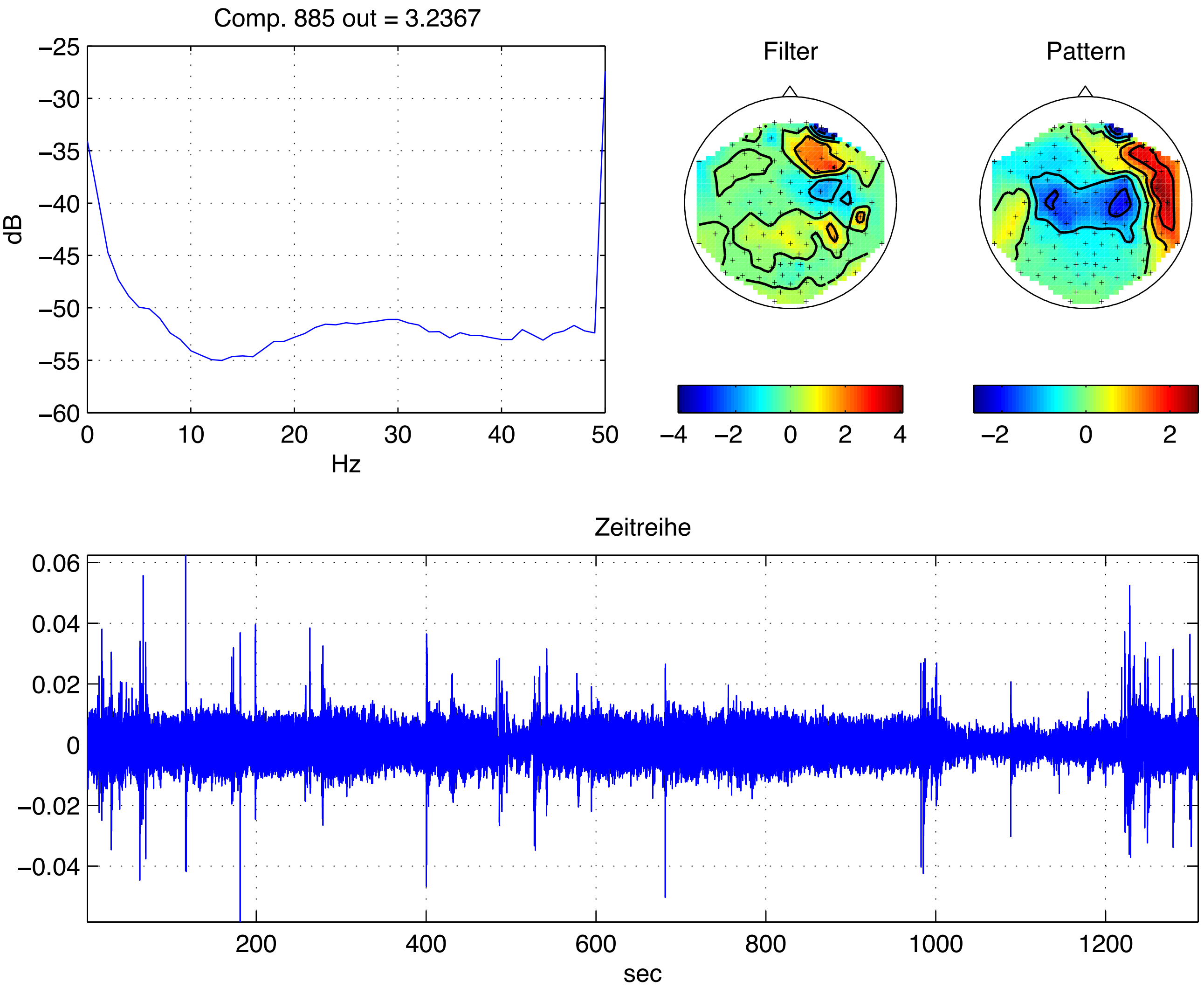

Comp. 885 out = 3.2367
dB
Hz
Filter
Pattern
Zeitreihe
sec

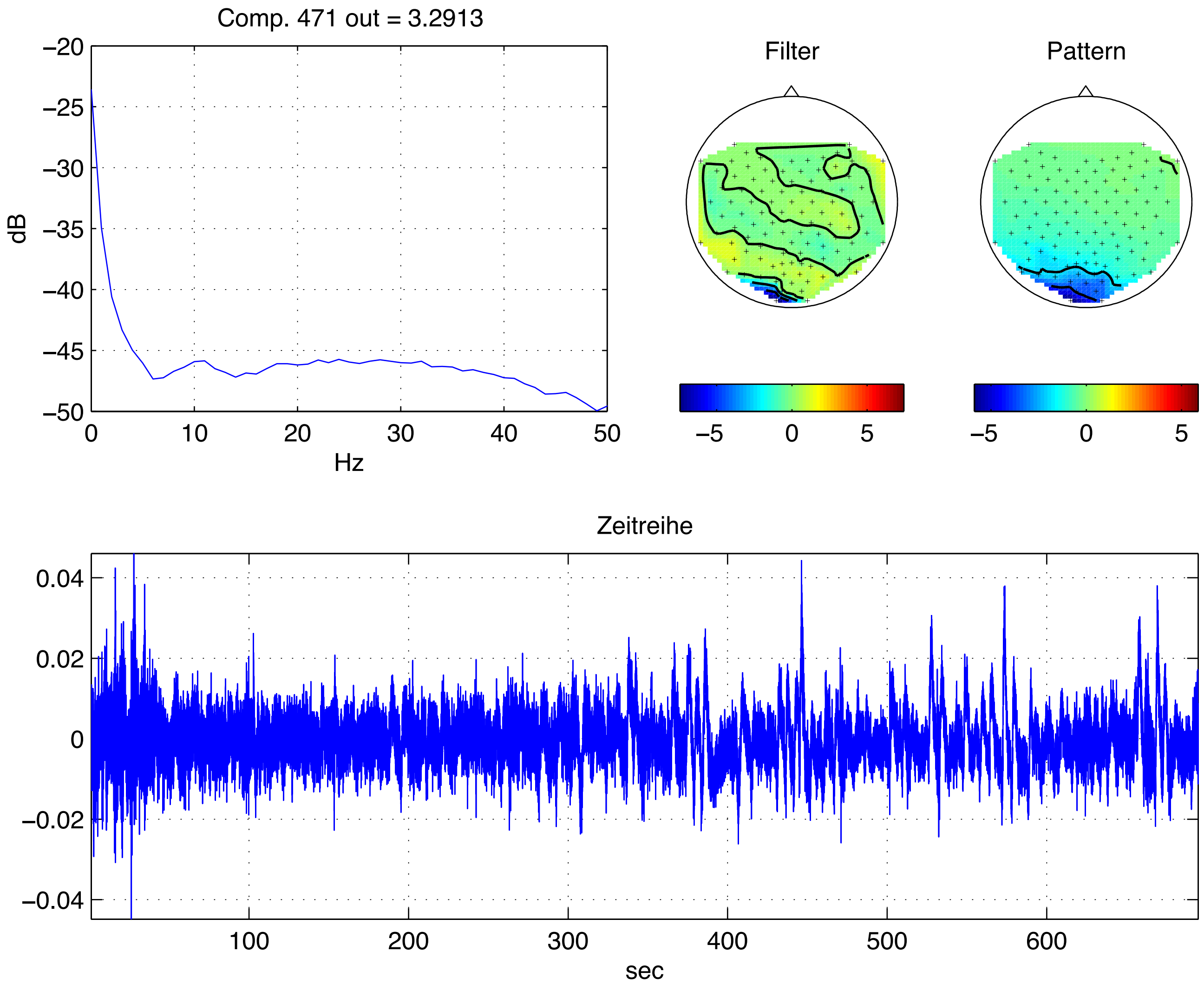

Comp. 471 out = 3.2913
dB
Hz
Filter
Pattern
Zeitreihe
sec

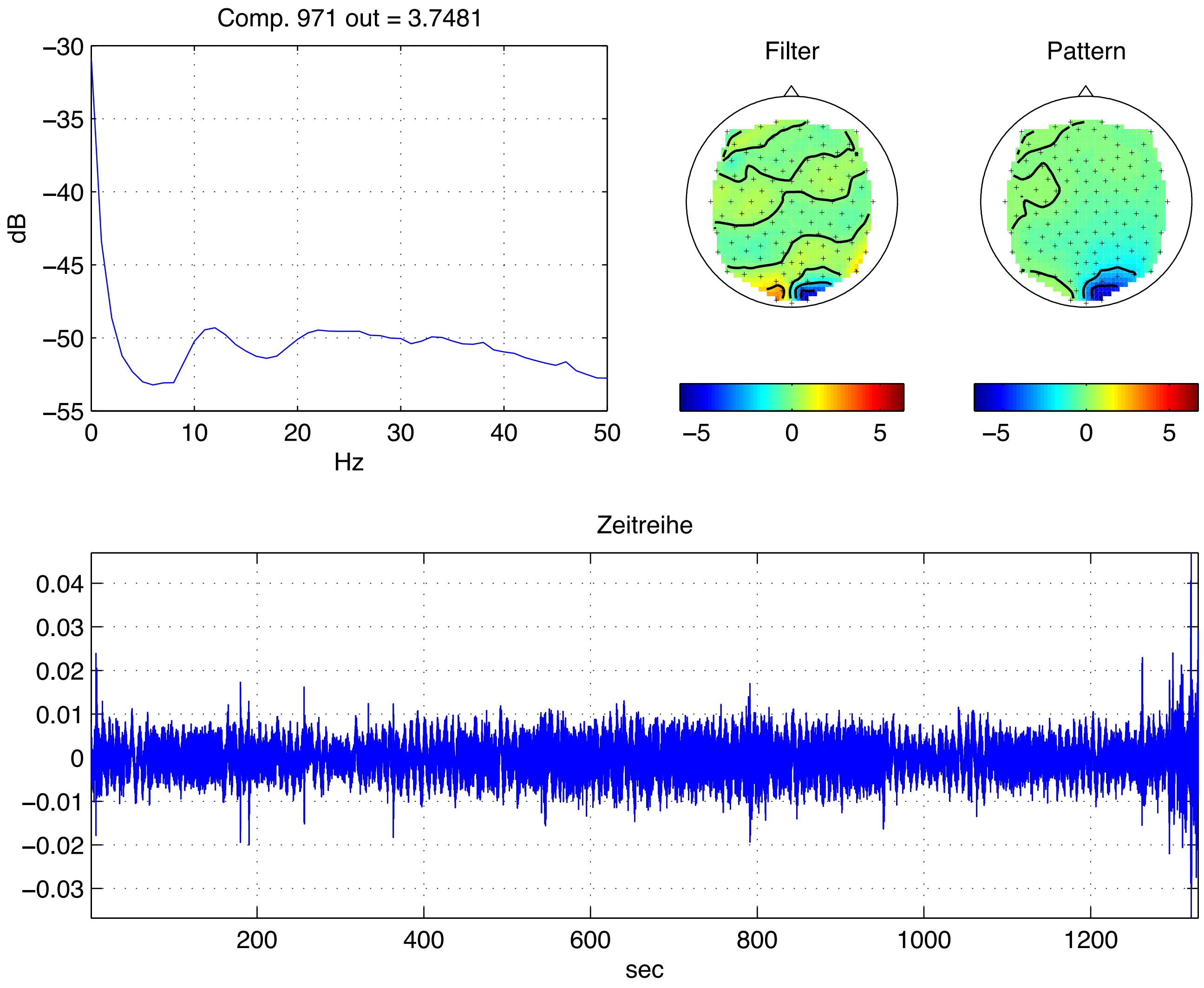

Comp. 971 out = 3.7481
dB
Hz
Filter
Pattern
Zeitreihe
sec

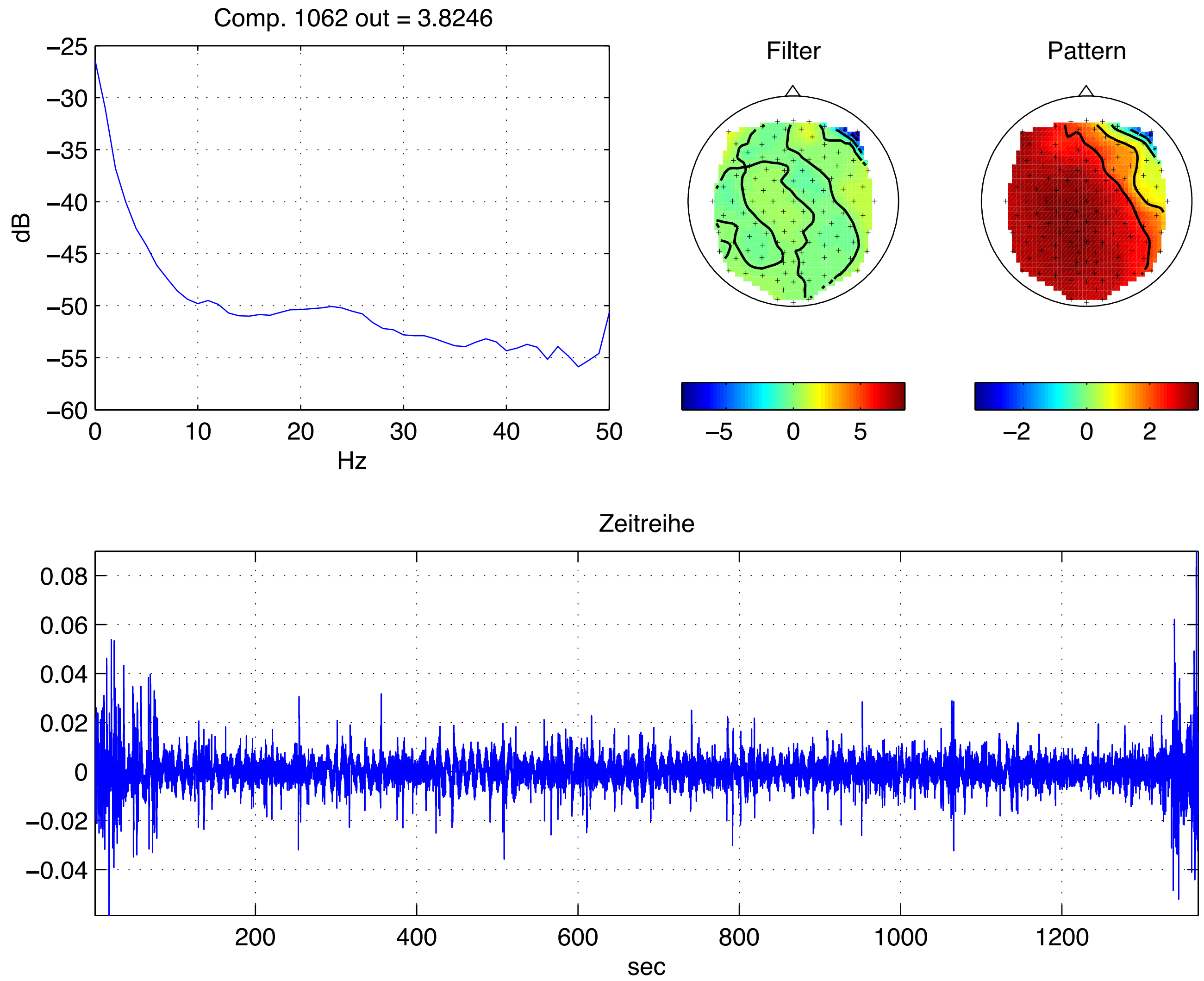

Comp. 1062 out = 3.8246
dB
Hz
Filter
Pattern
Zeitreihe
sec

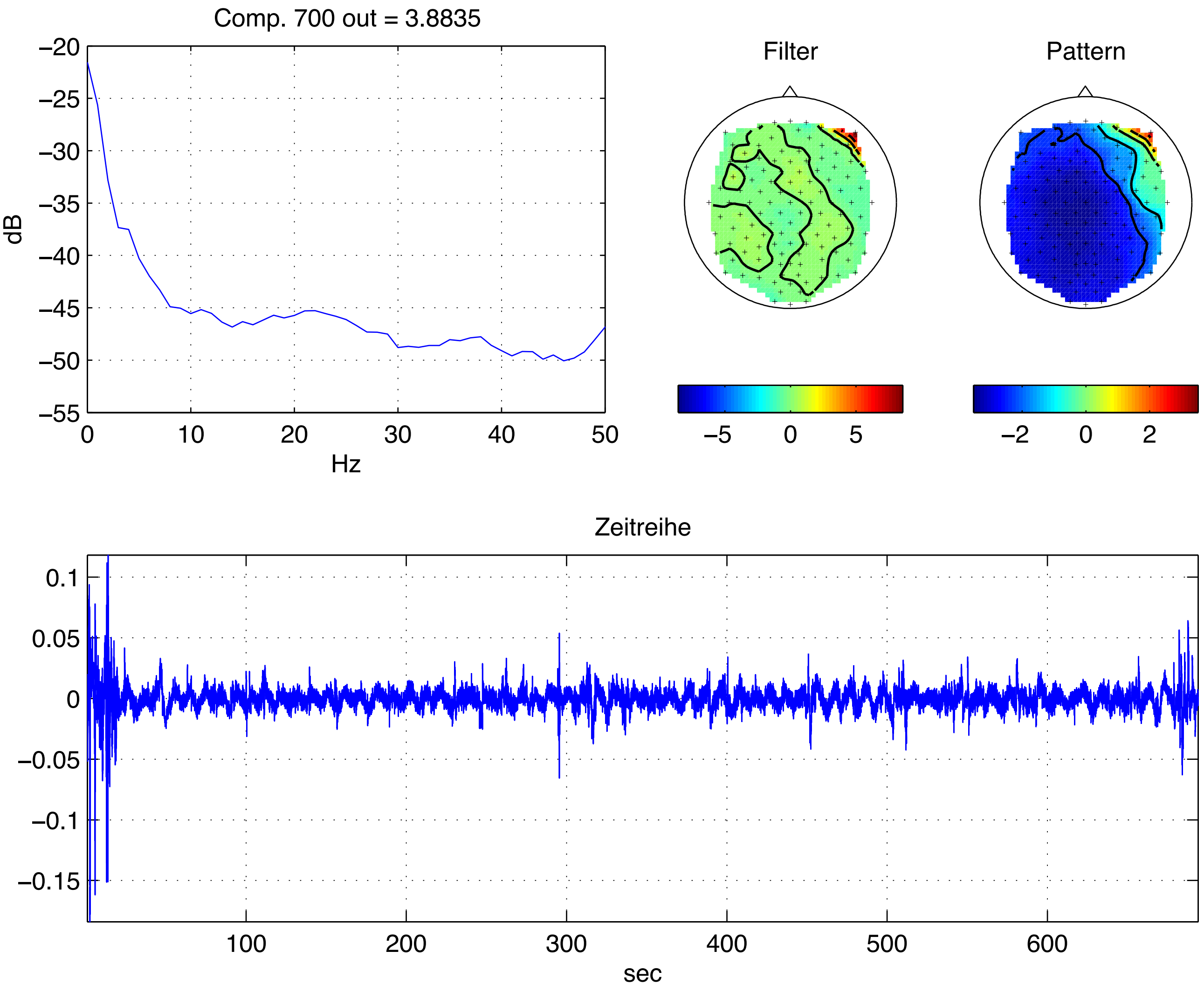

Comp. 700 out = 3.8835
dB
Hz
−20
−25
−30
−35
−40
−45
−50
−55
0
10
20
30
40
50
Filter
Pattern
−5
0
5
−2
0
2
Zeitreihe
0.1
0.05
0
−0.05
−0.1
−0.15
100
200
300
400
500
600
sec

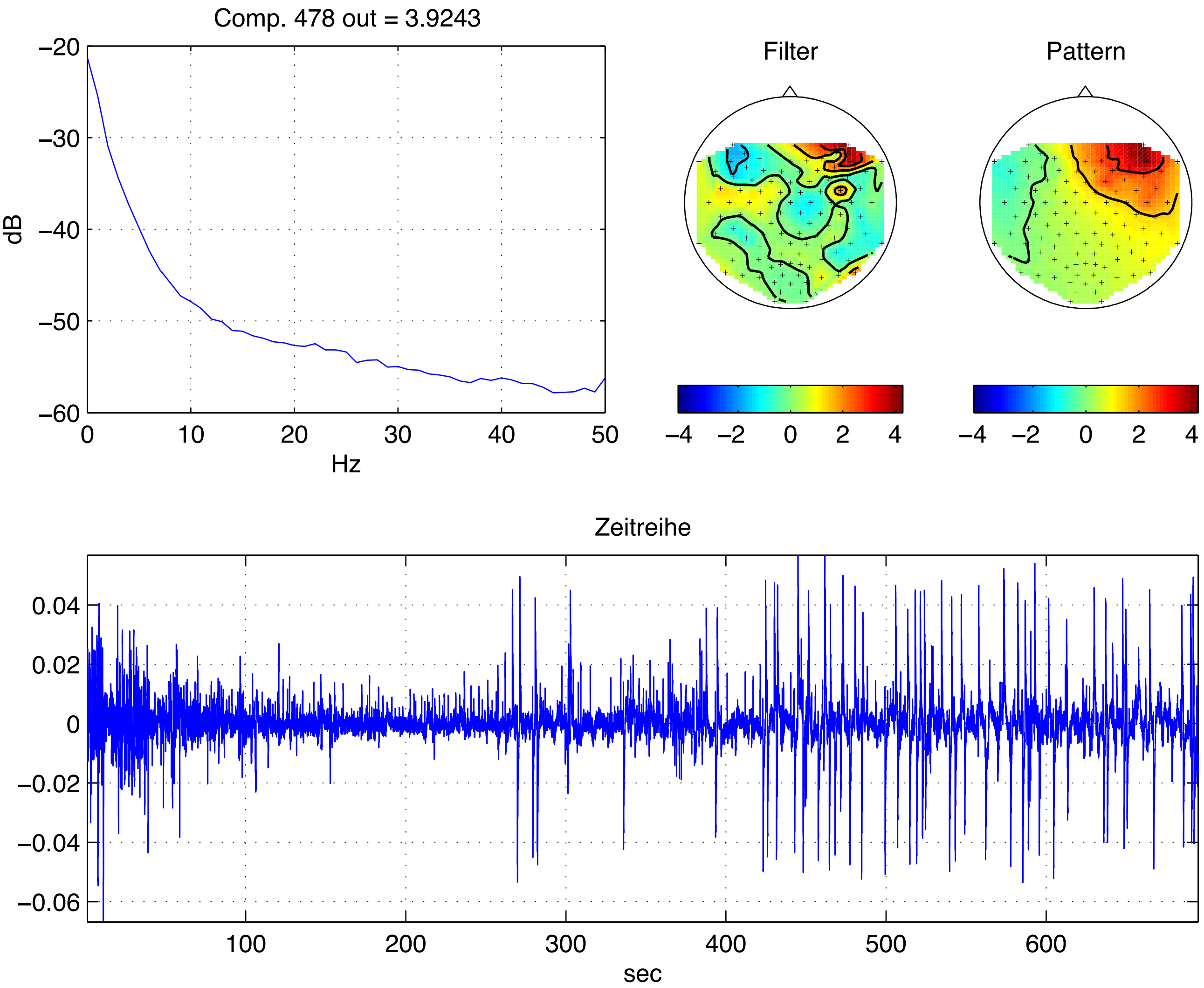

Comp. 478 out = 3.9243
dB
Hz
Filter
Pattern
Zeitreihe
sec

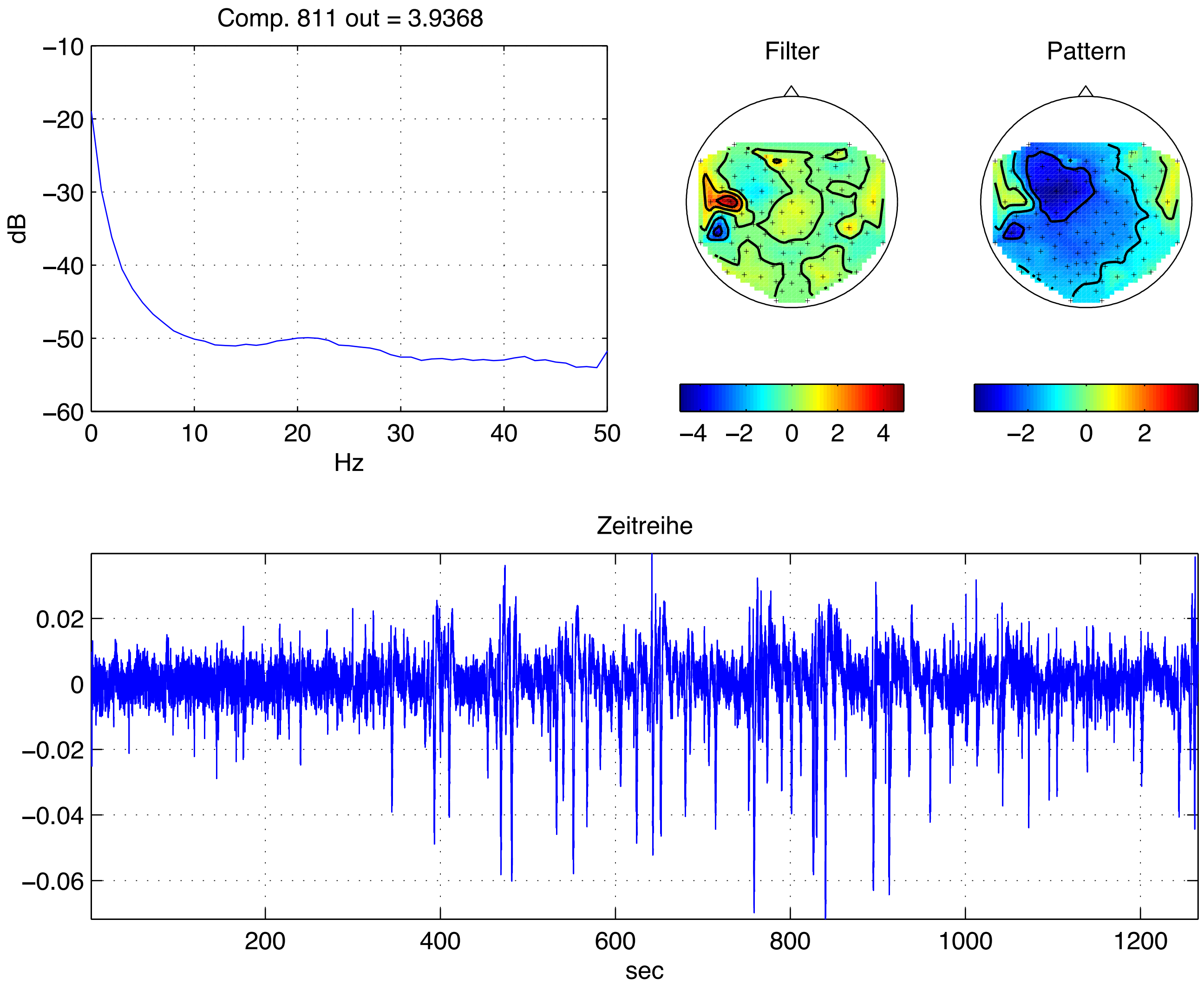

Comp. 811 out = 3.9368
dB
Hz
Filter
Pattern
Zeitreihe
sec

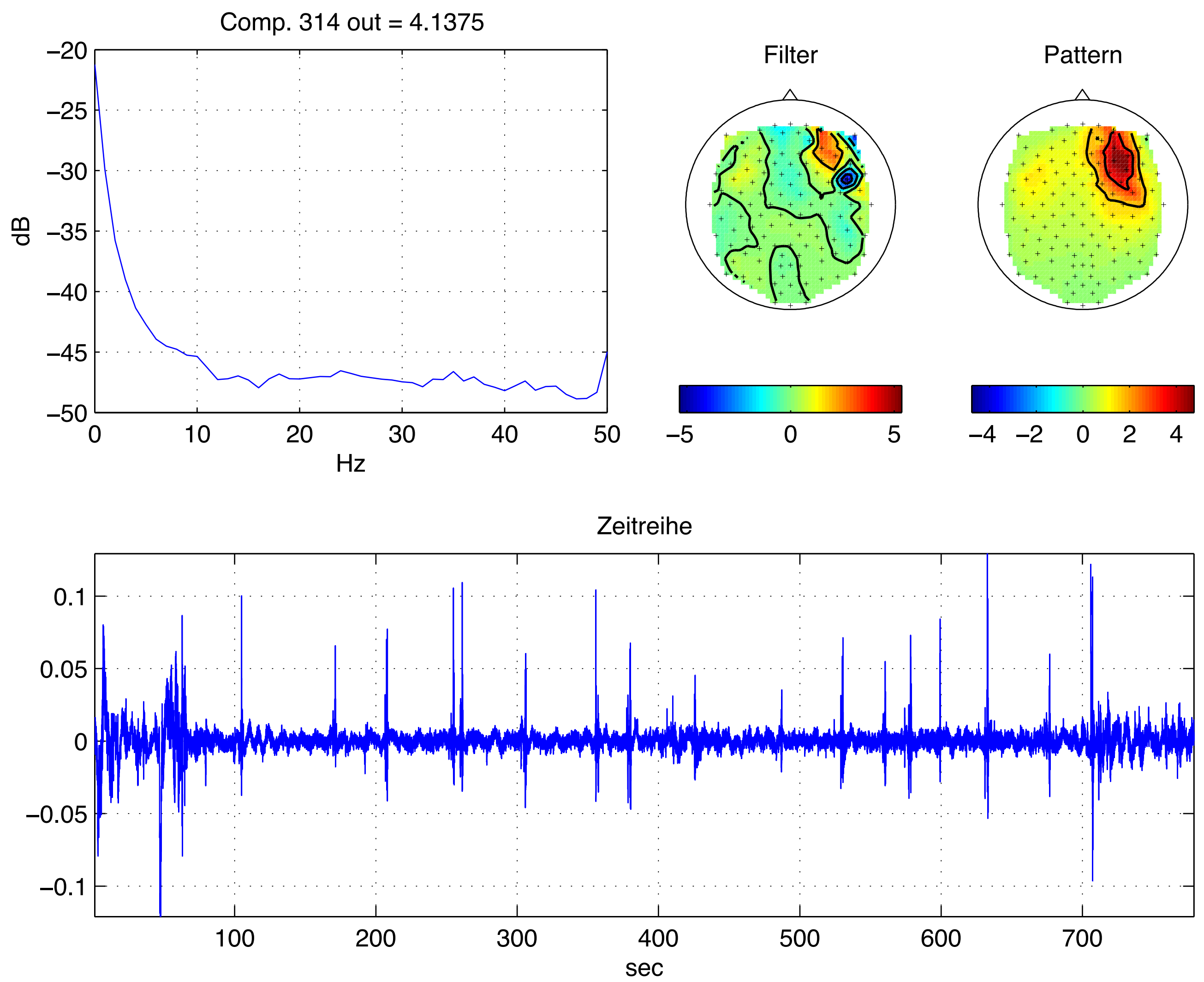

Comp. 314 out = 4.1375
dB
Hz
Filter
Pattern
Zeitreihe
sec

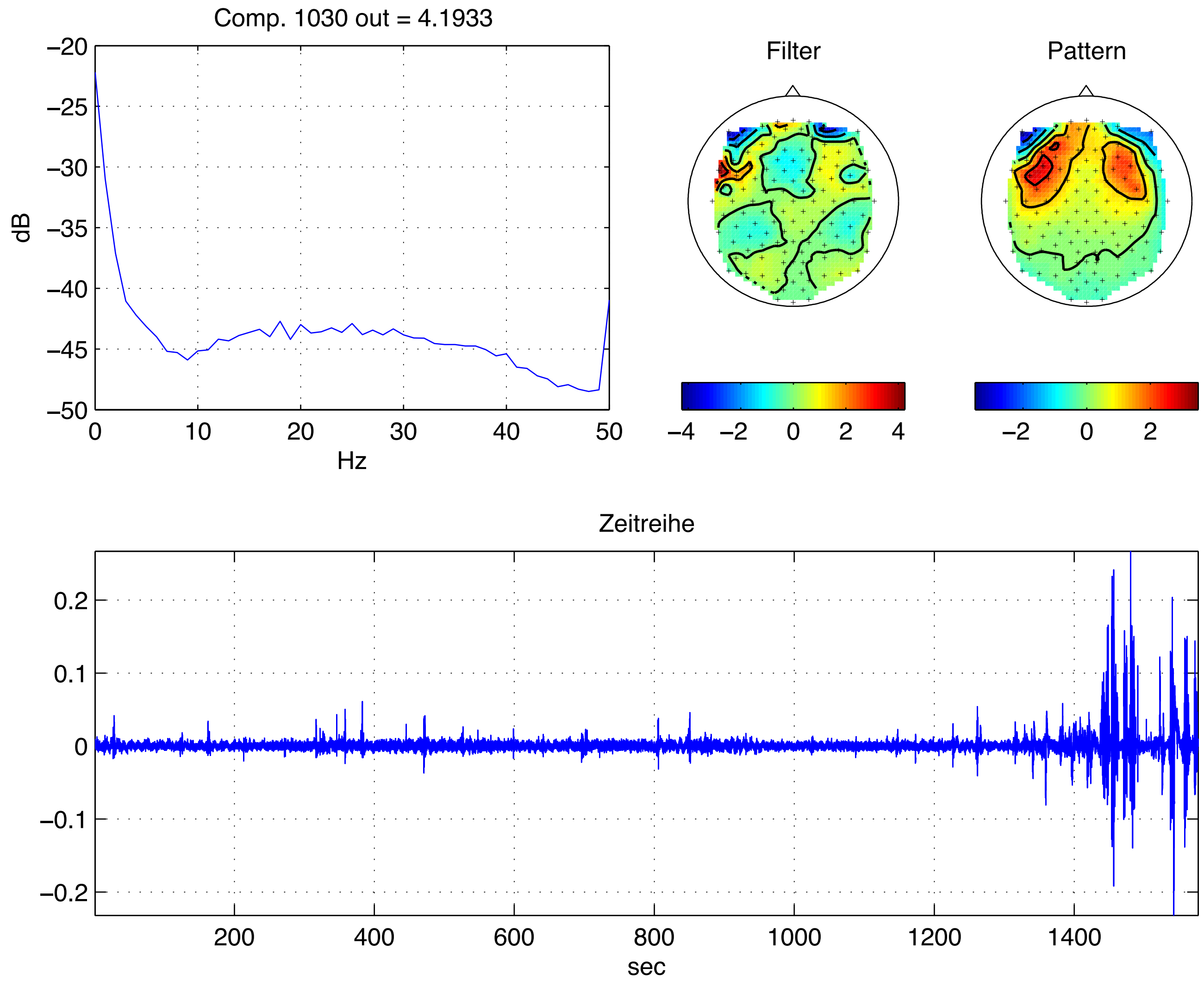

Comp. 1030 out = 4.1933
dB
Hz
Filter
Pattern
Zeitreihe
sec

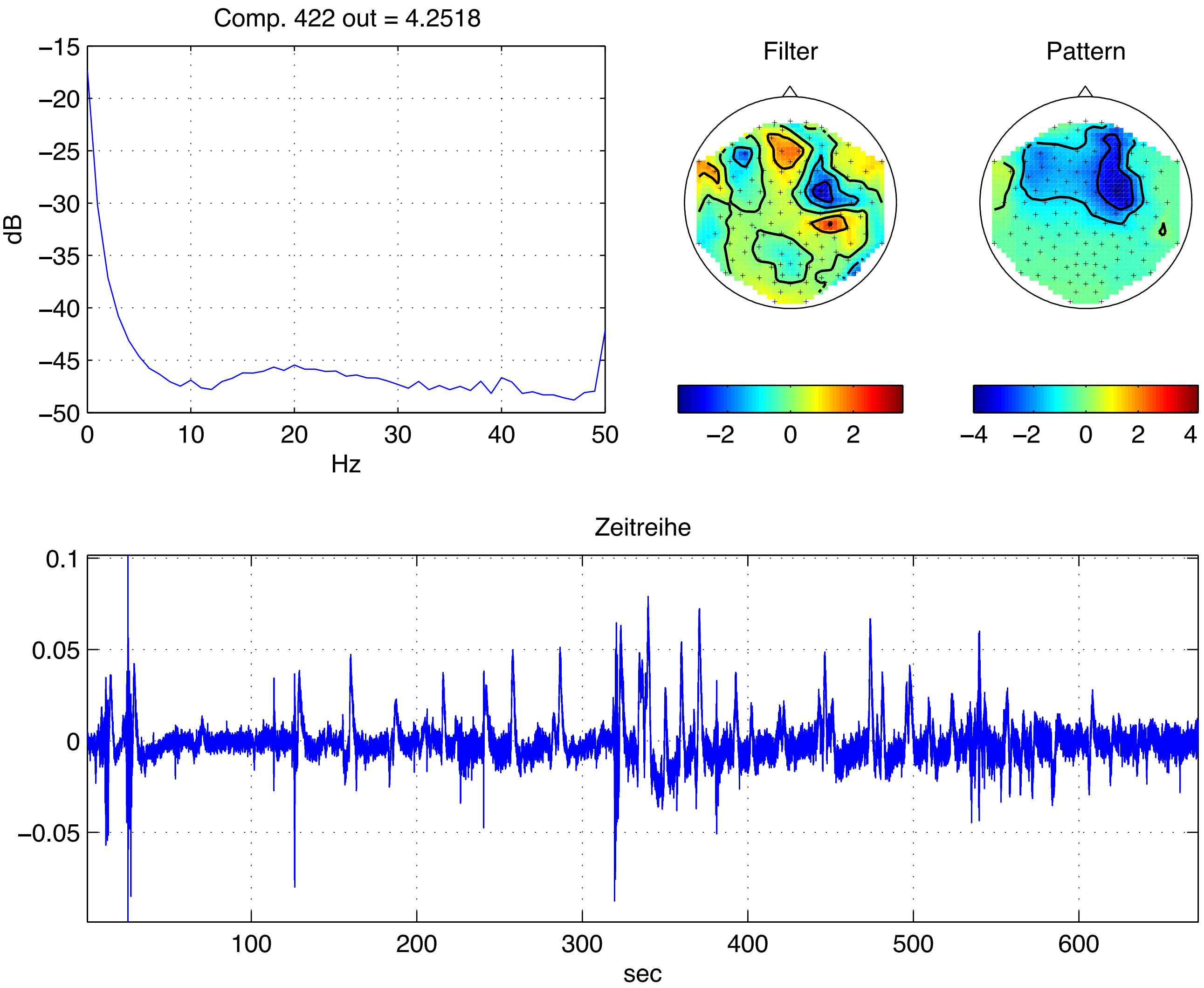

Comp. 422 out = 4.2518
dB
Hz
−15
−20
−25
−30
−35
−40
−45
−50
0
10
20
30
40
50
Filter
Pattern
−2
0
2
−4
−2
0
2
4
Zeitreihe
0.1
0.05
0
−0.05
100
200
300
400
500
600
sec

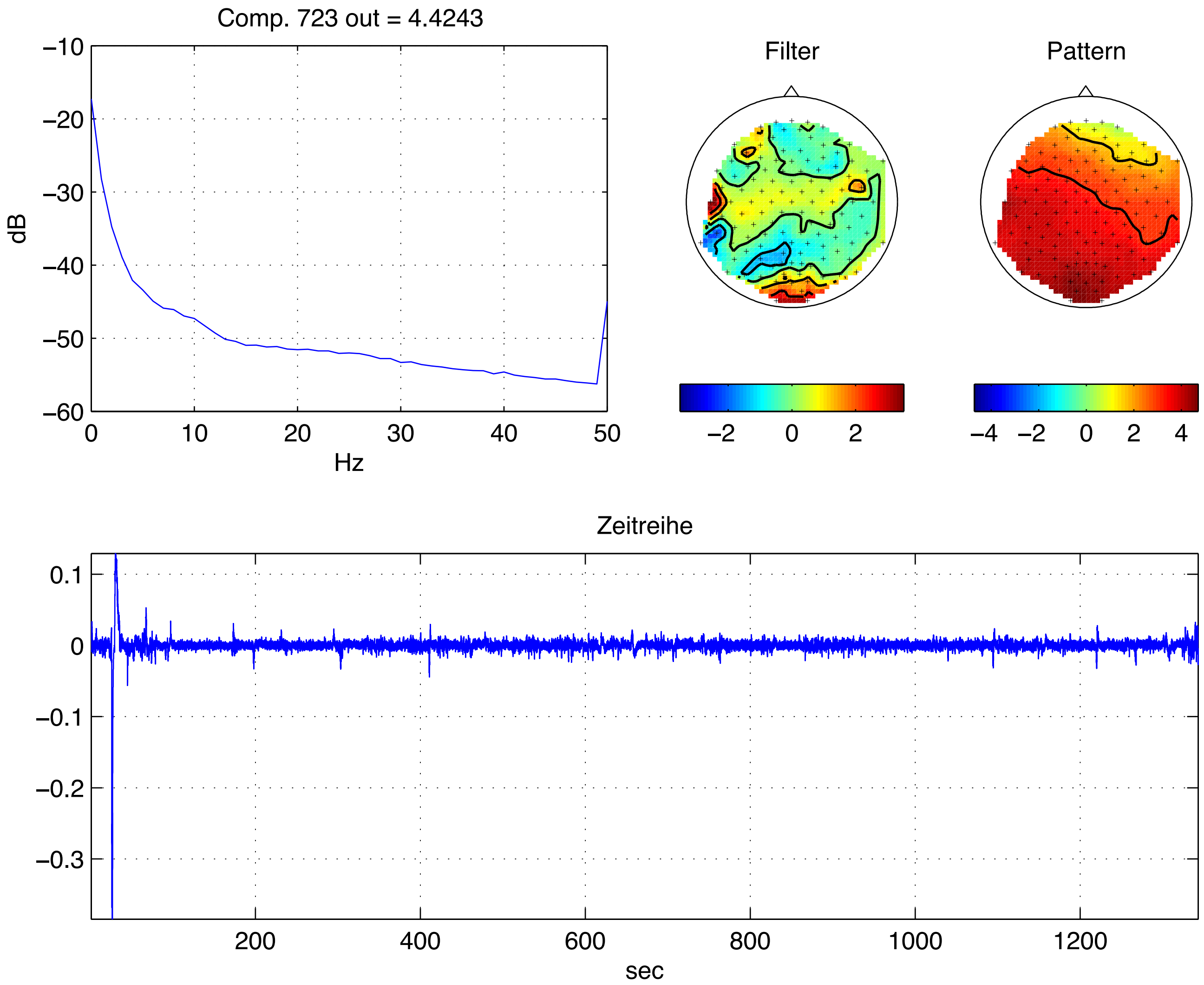

Comp. 723 out = 4.4243
dB
Hz
Filter
Pattern
Zeitreihe
sec

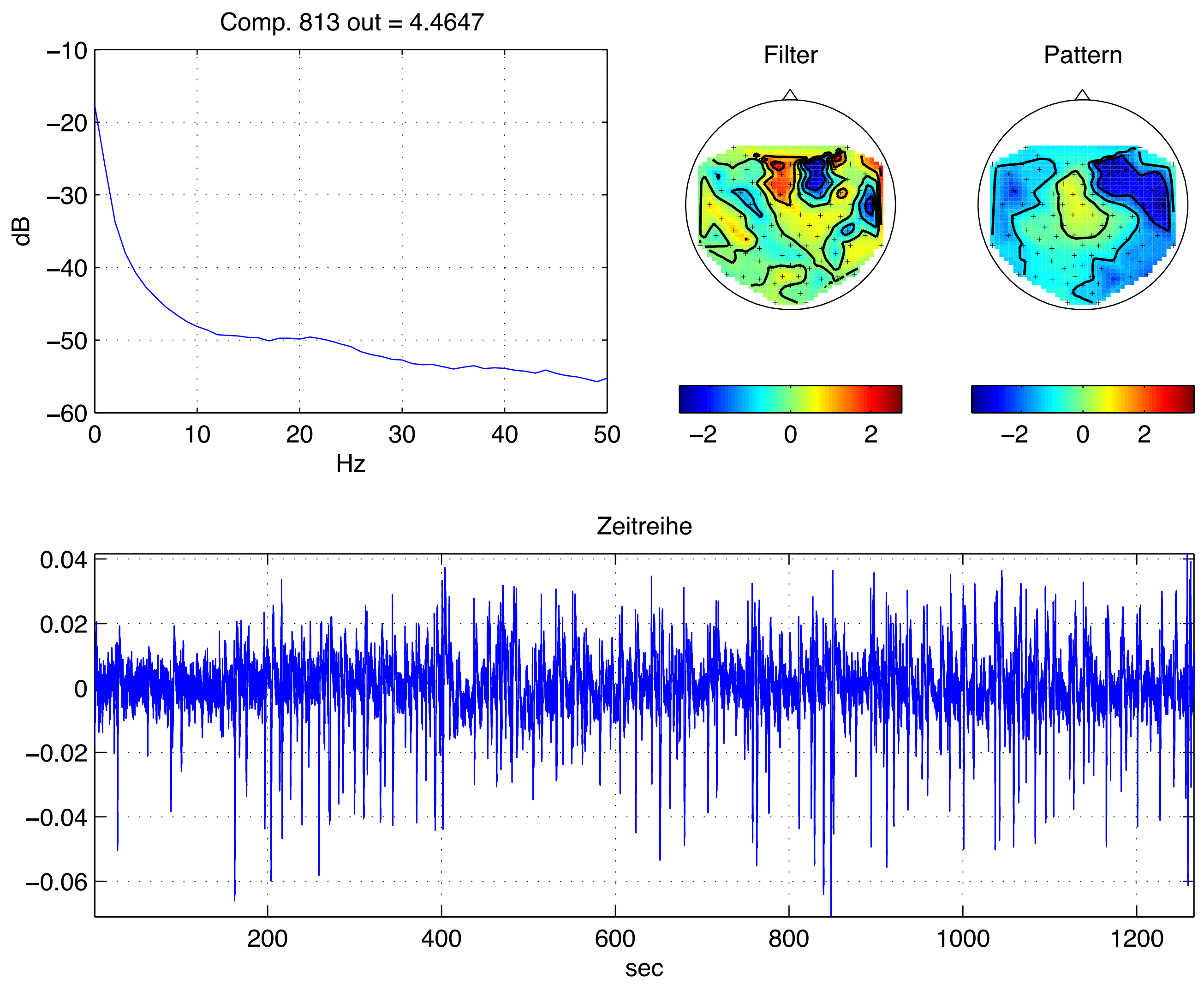

Comp. 813 out = 4.4647
dB
Hz
Filter
Pattern
Zeitreihe
sec

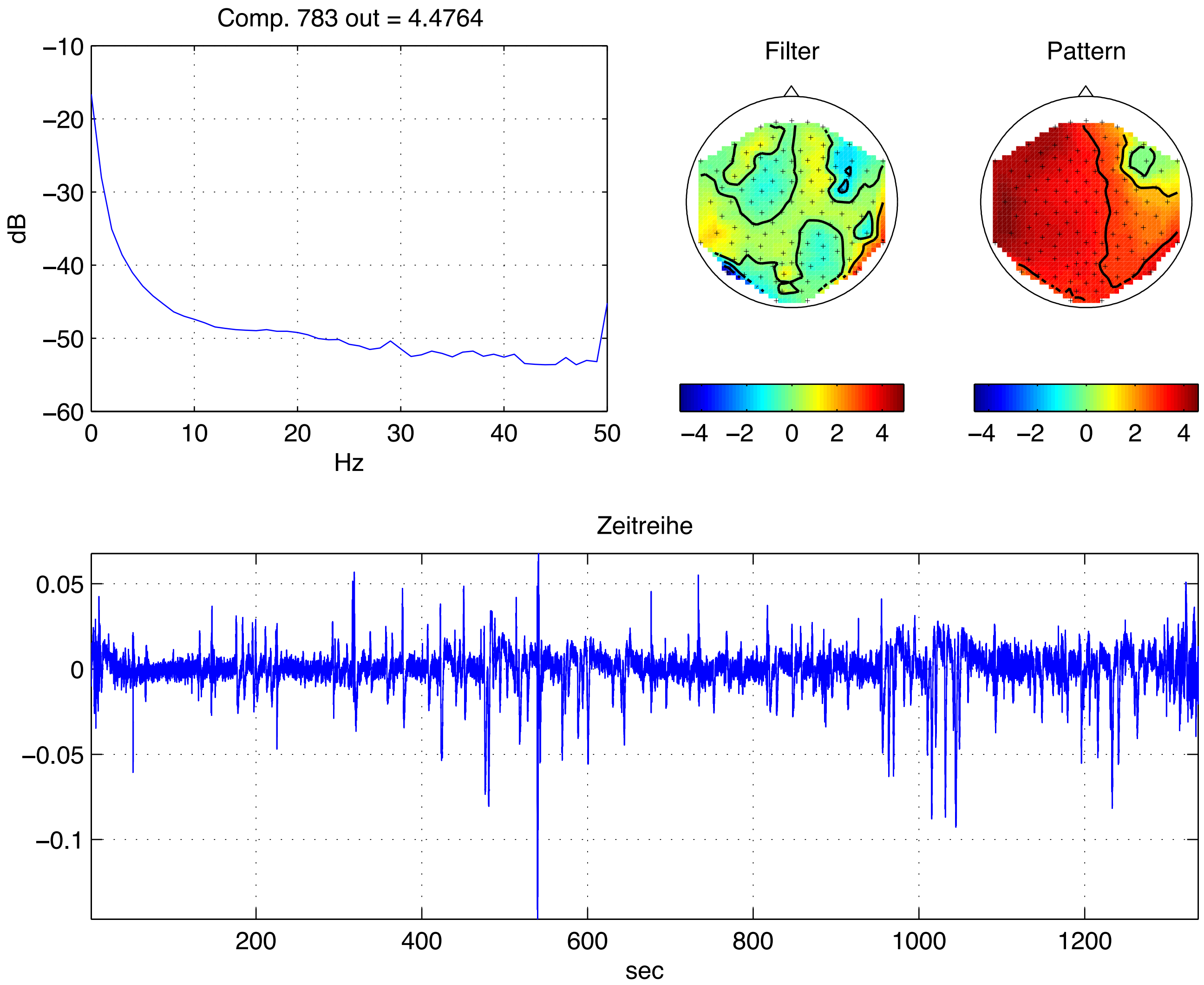

Comp. 783 out = 4.4764
dB
Hz
Filter
Pattern
Zeitreihe
sec

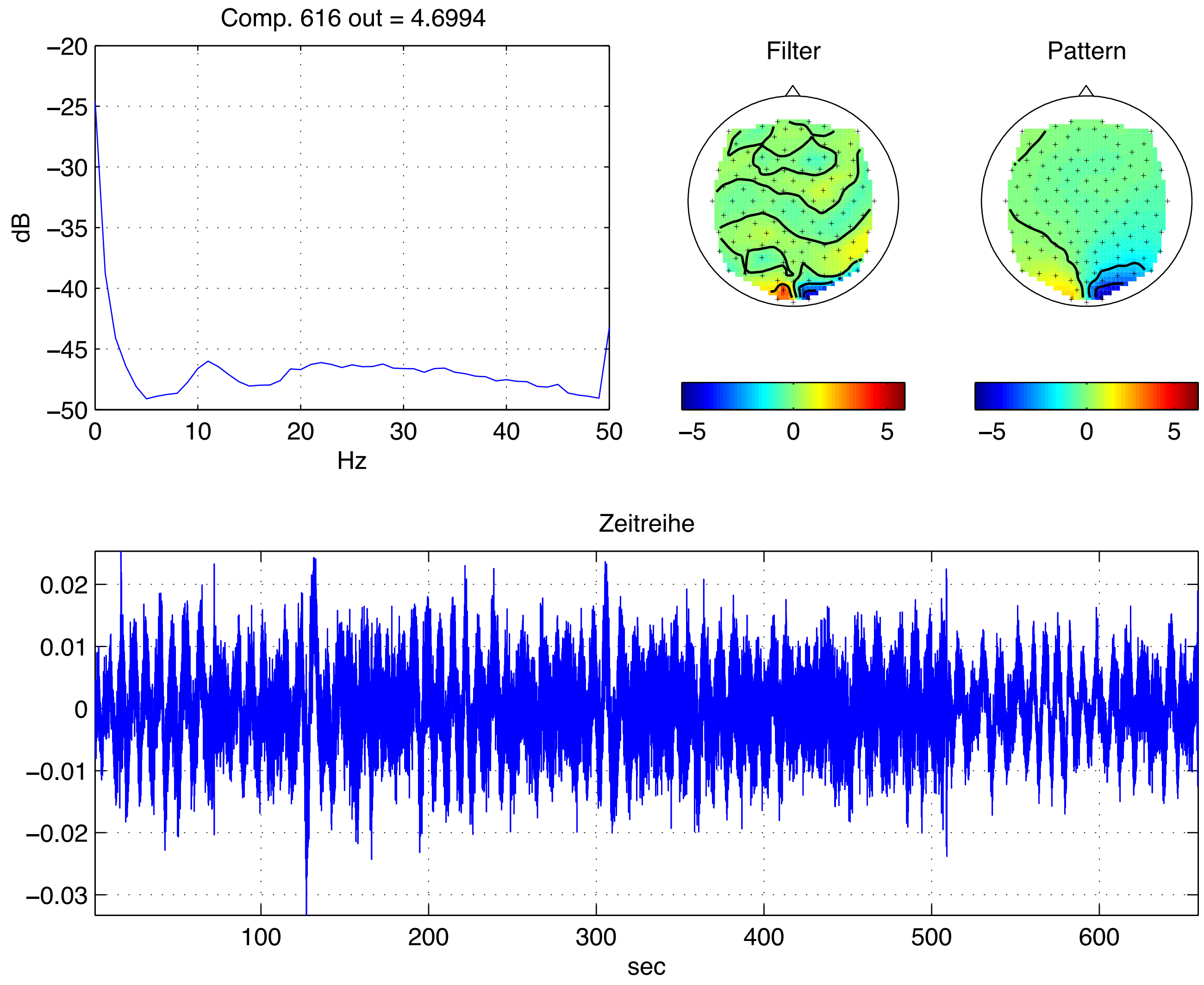
Comp. 616 out = 4.6994
dB
Hz
Filter
Pattern
Zeitreihe
sec

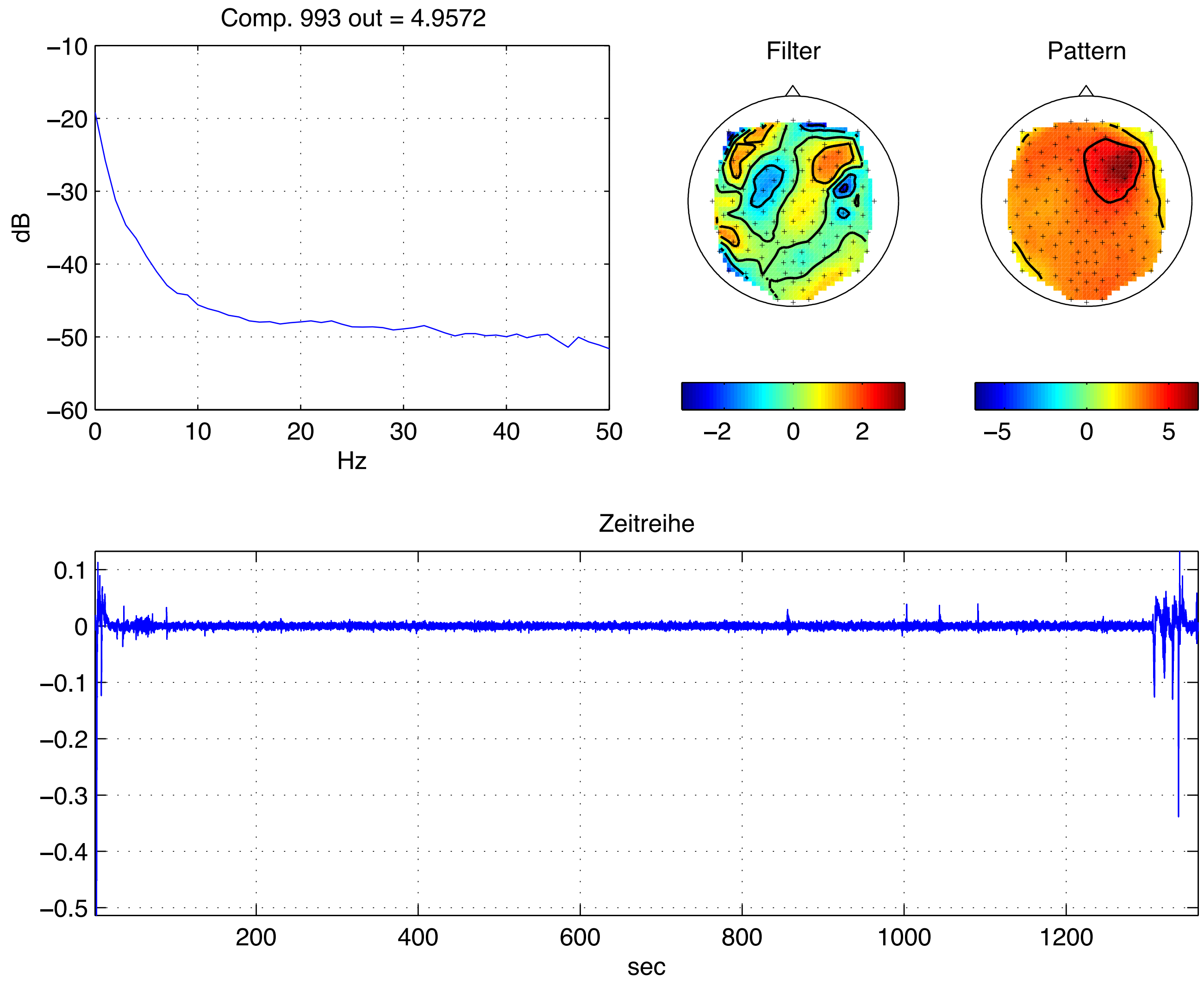
Comp. 993 out = 4.9572
dB
Hz
Filter
Pattern
Zeitreihe
sec

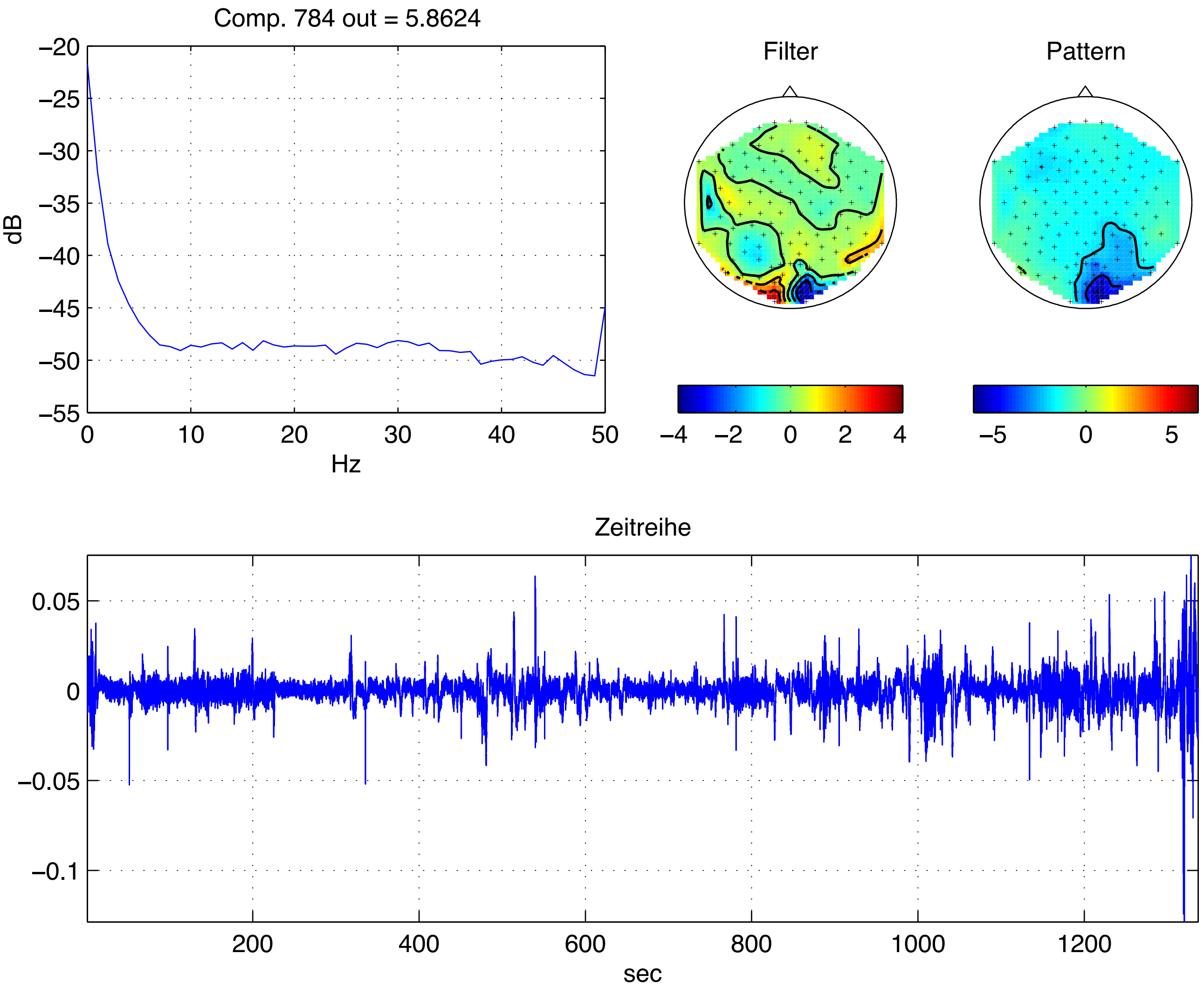

Comp. 784 out = 5.8624
dB
Hz
Filter
Pattern
Zeitreihe
sec

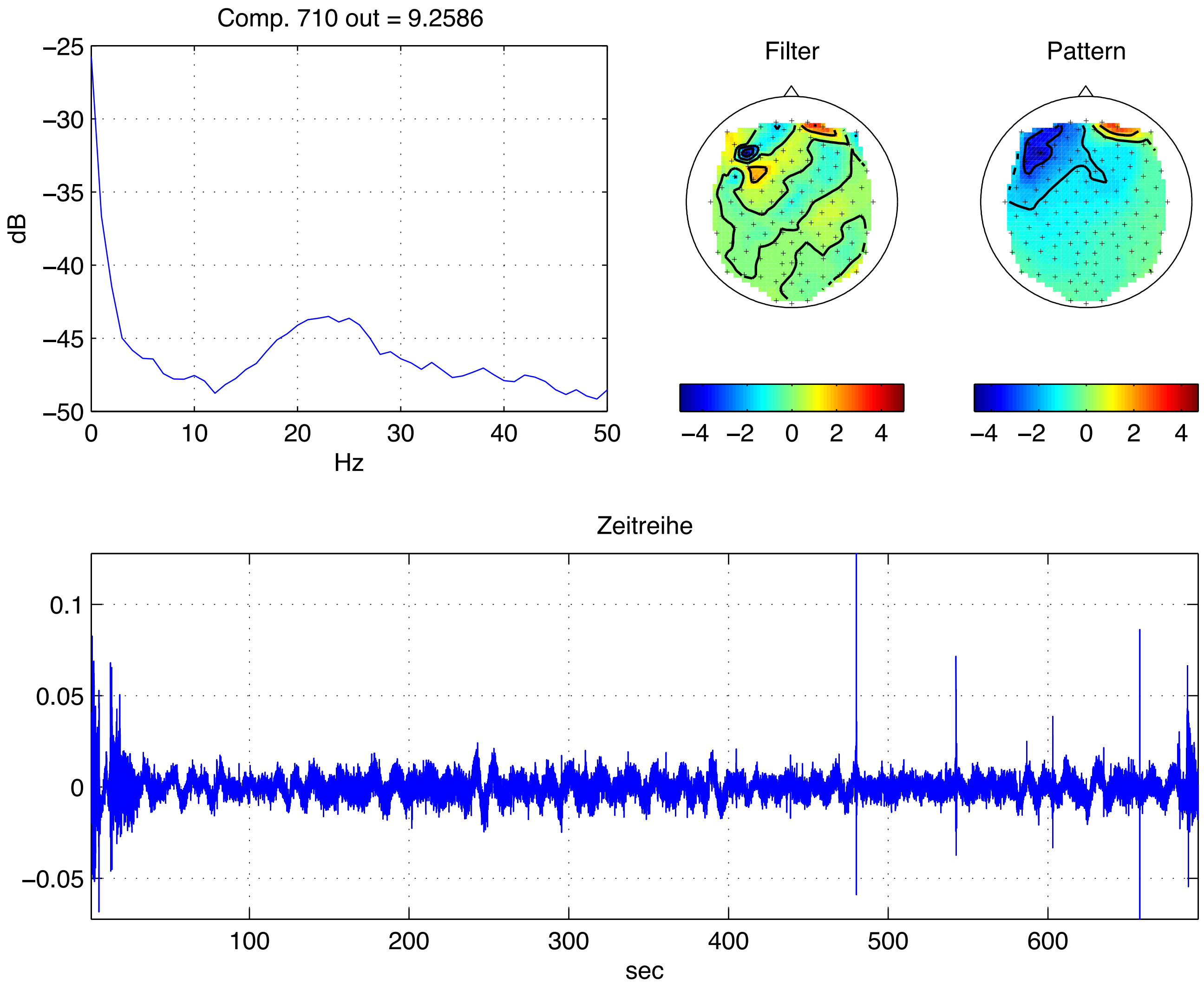
Comp. 710 out = 9.2586
dB
Hz
−25
−30
−35
−40
−45
−50
0
10
20
30
40
50
Filter
Pattern
−4
−2
0
2
4
Zeitreihe
0.1
0.05
0
−0.05
100
200
300
400
500
600
sec
