# Supplementary figures and images for: Automatic Classification of Artifactual ICA-Components for Artifact Removal in EEG Signals (part 5 of 5)
Source: Behav Brain Funct. 2011 Aug 2;7:30. doi: 10.1186/1744-9081-7-30 (PMC3175453; doi:10.1186/1744-9081-7-30)

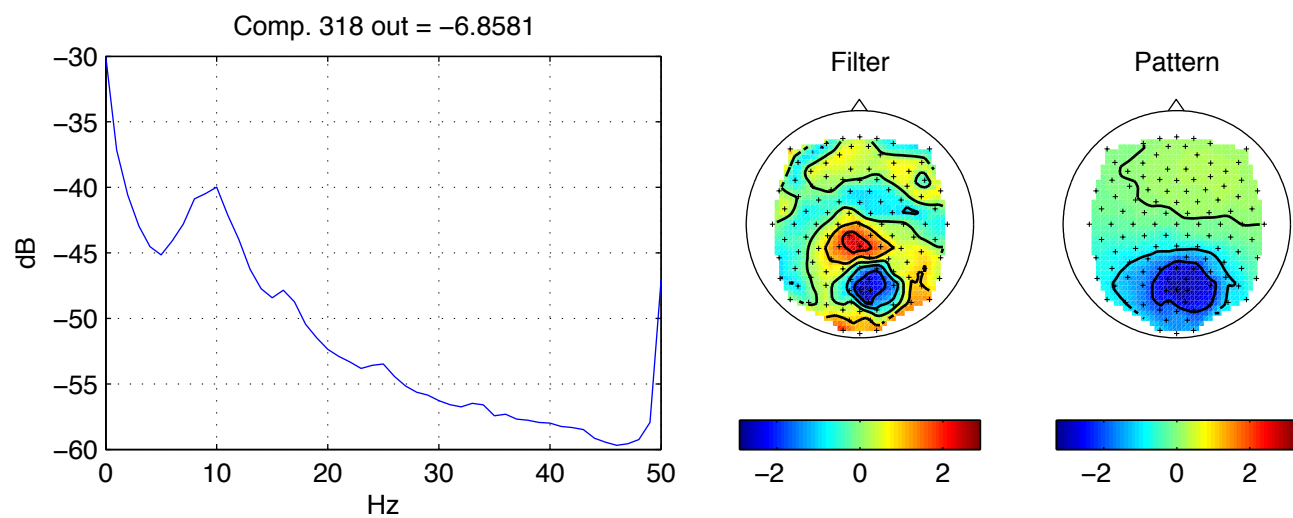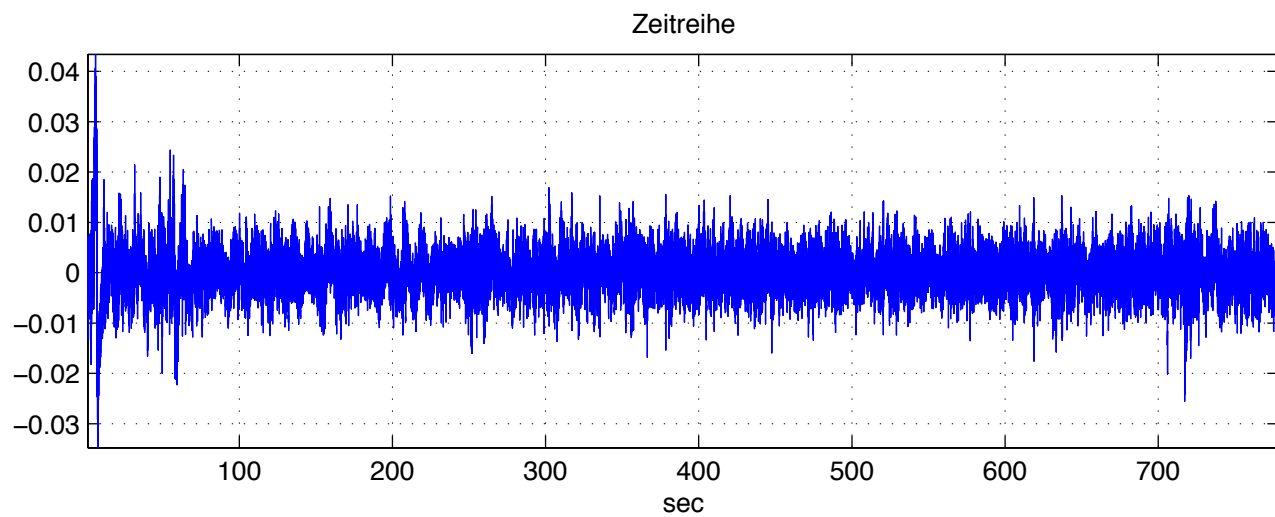

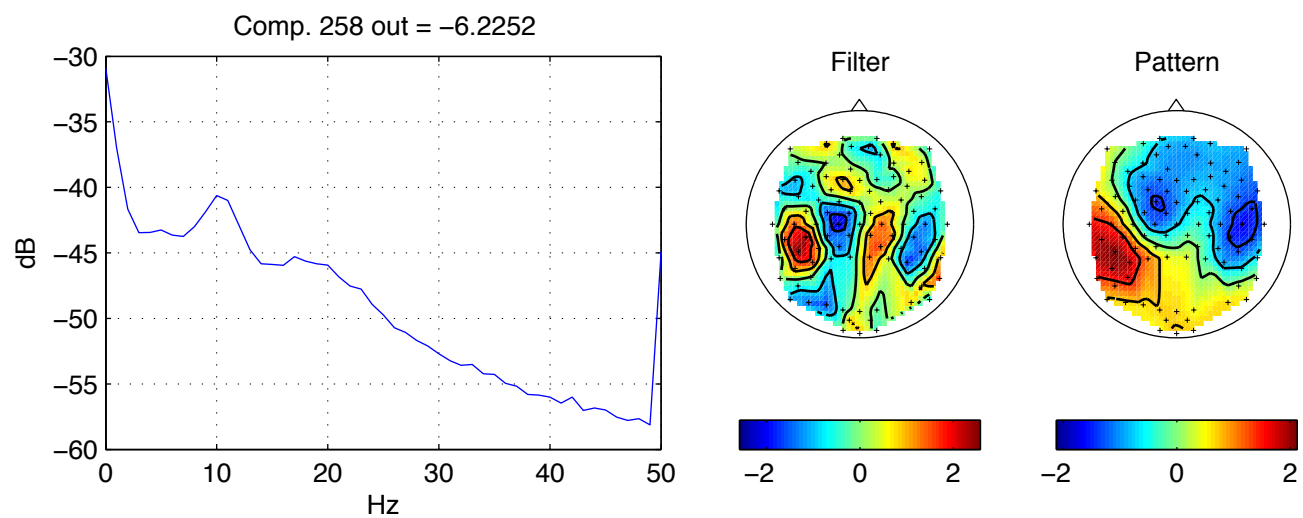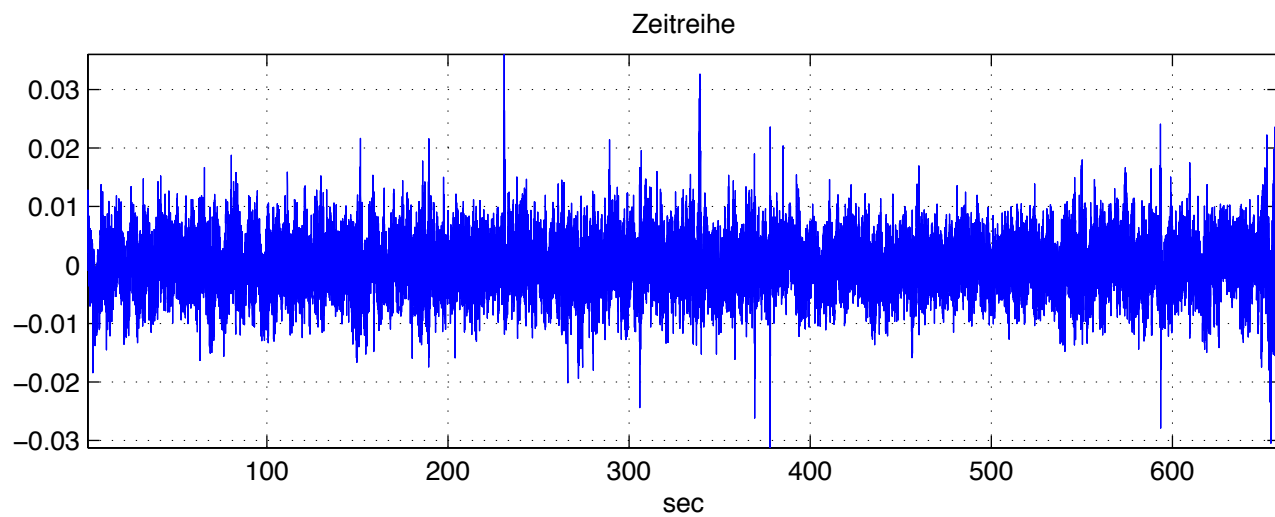

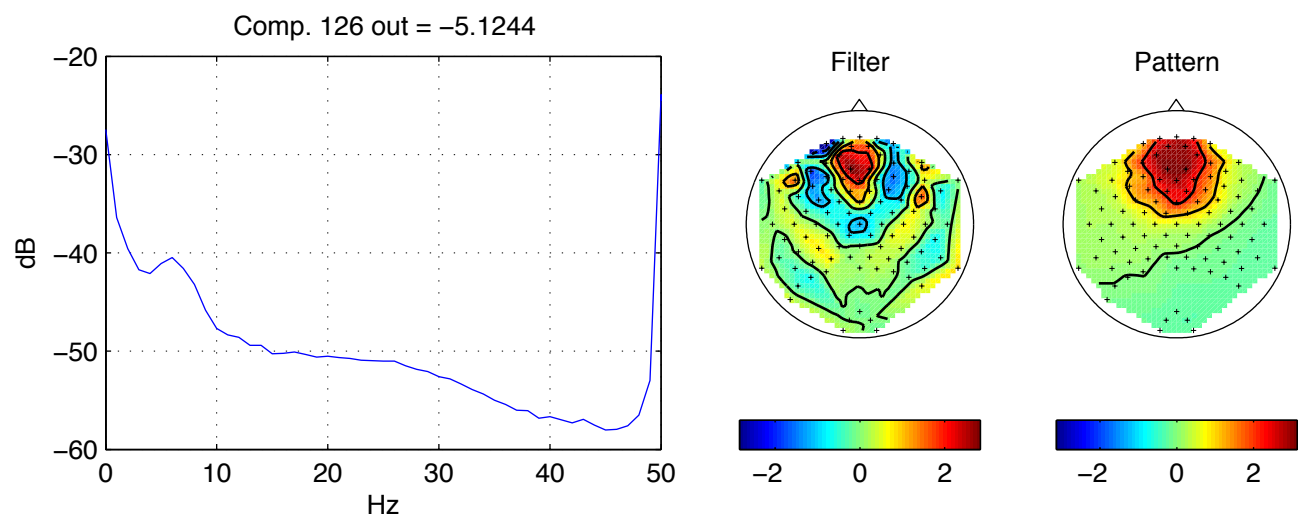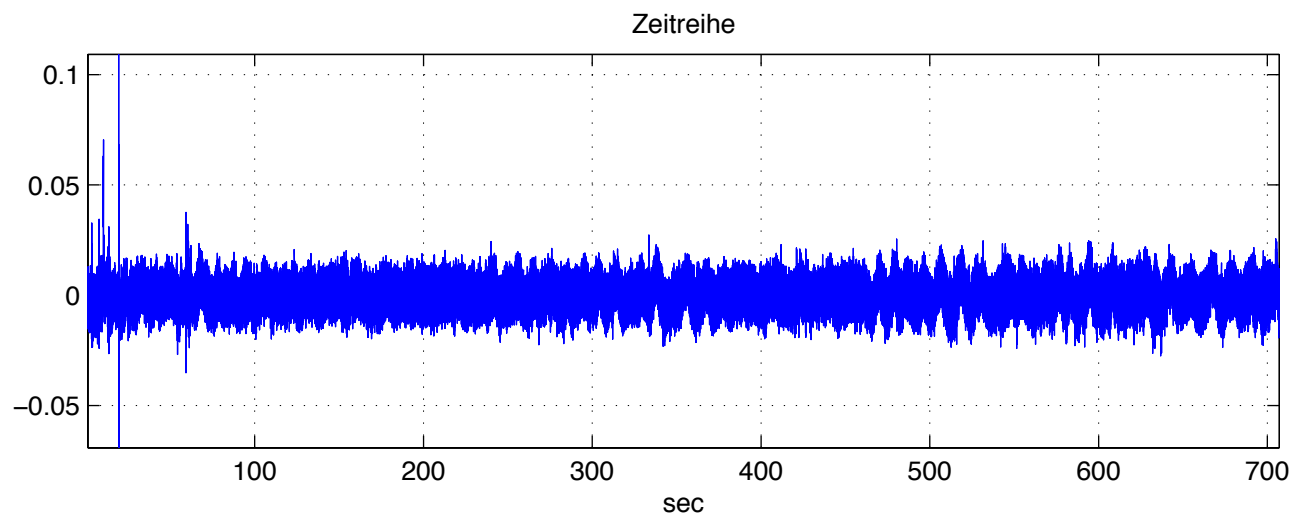

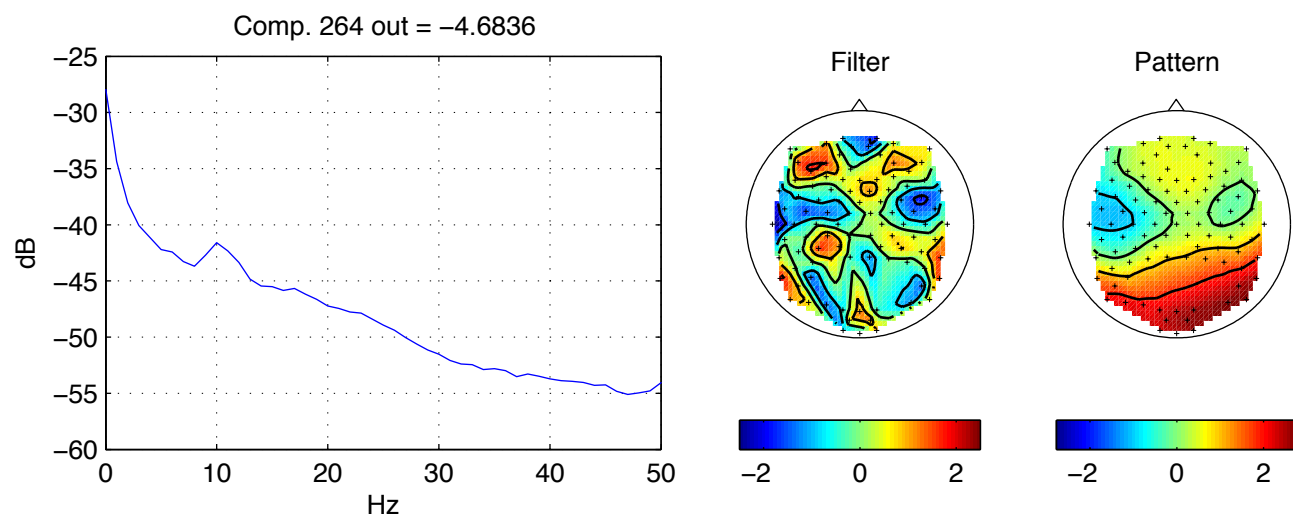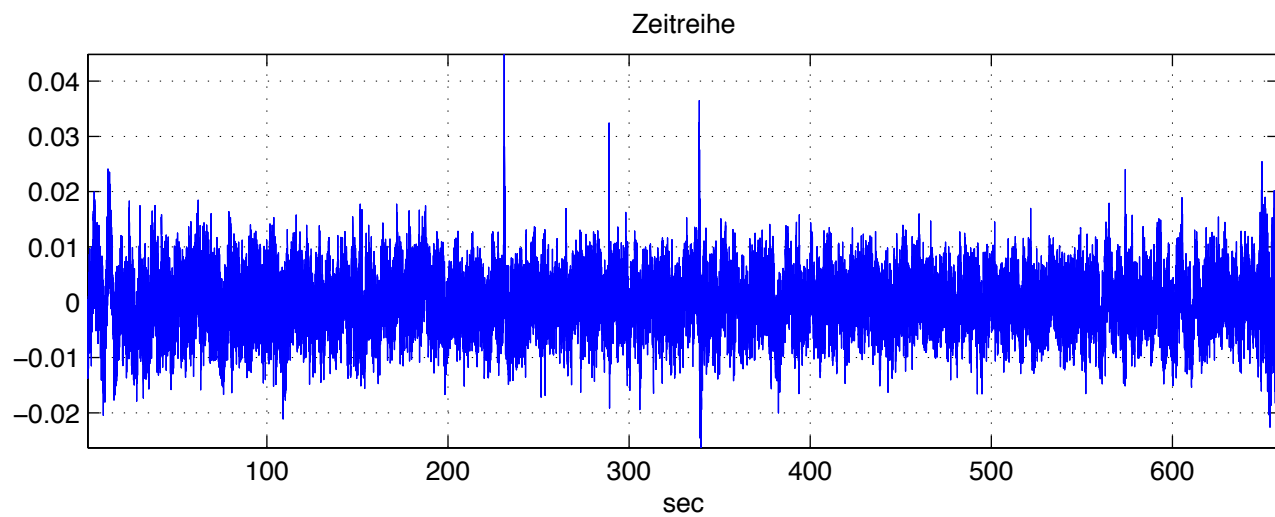

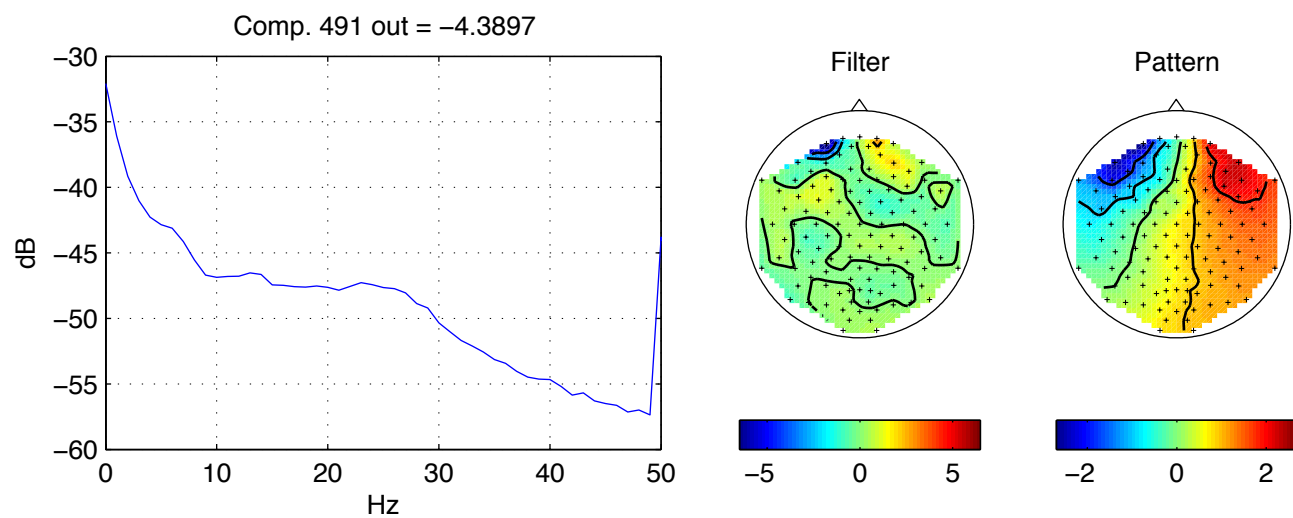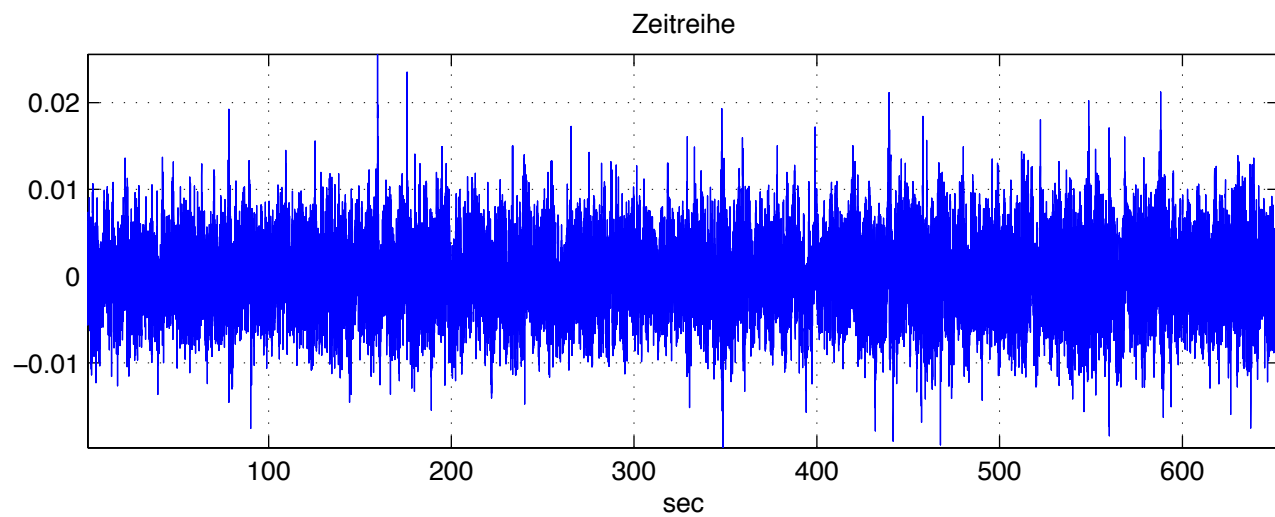

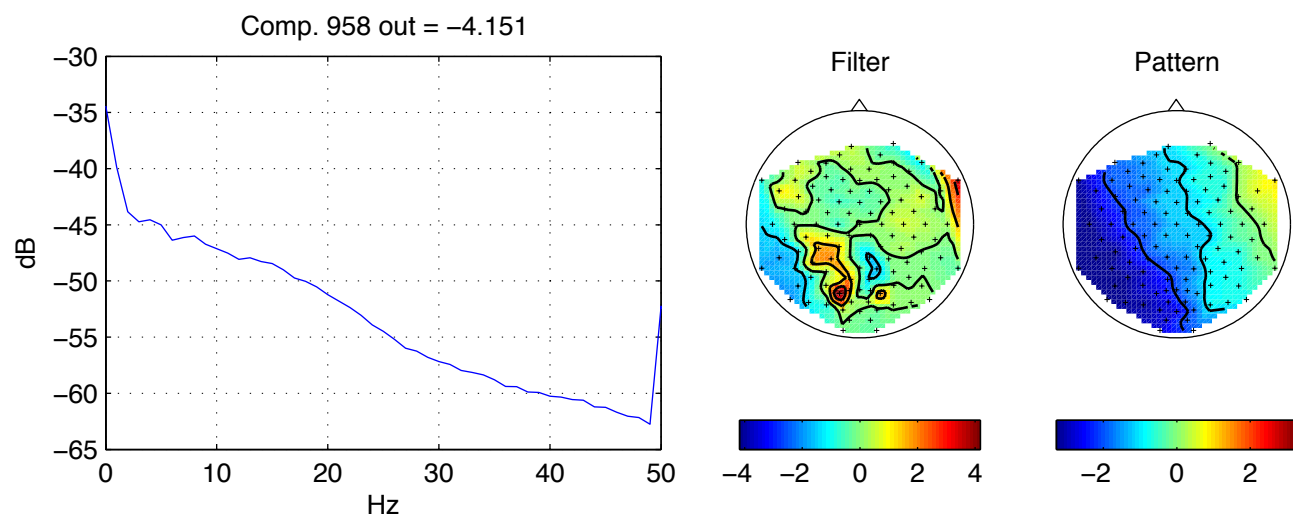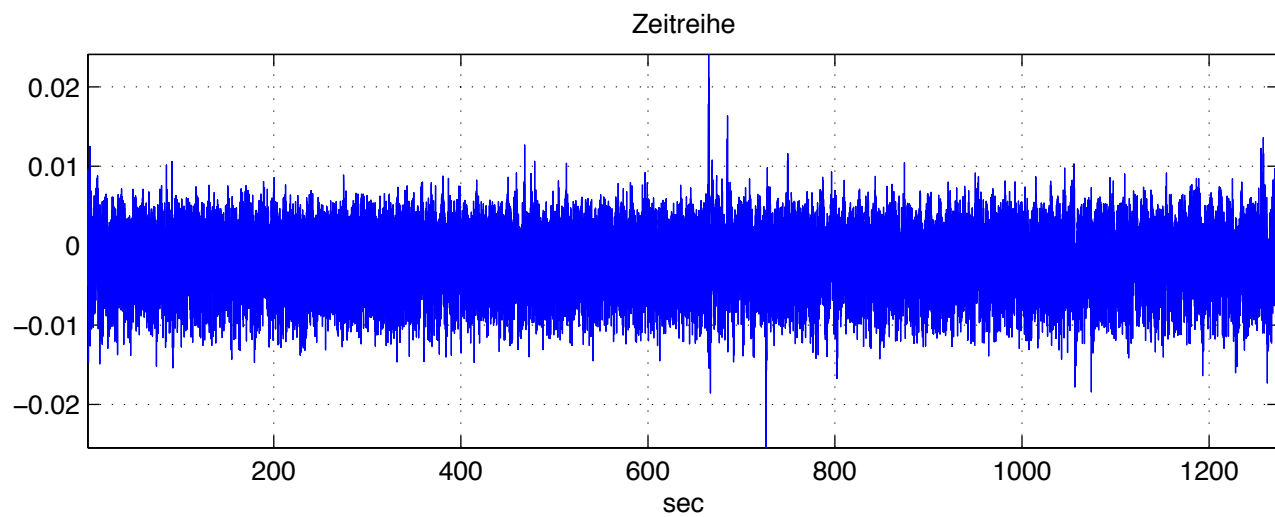

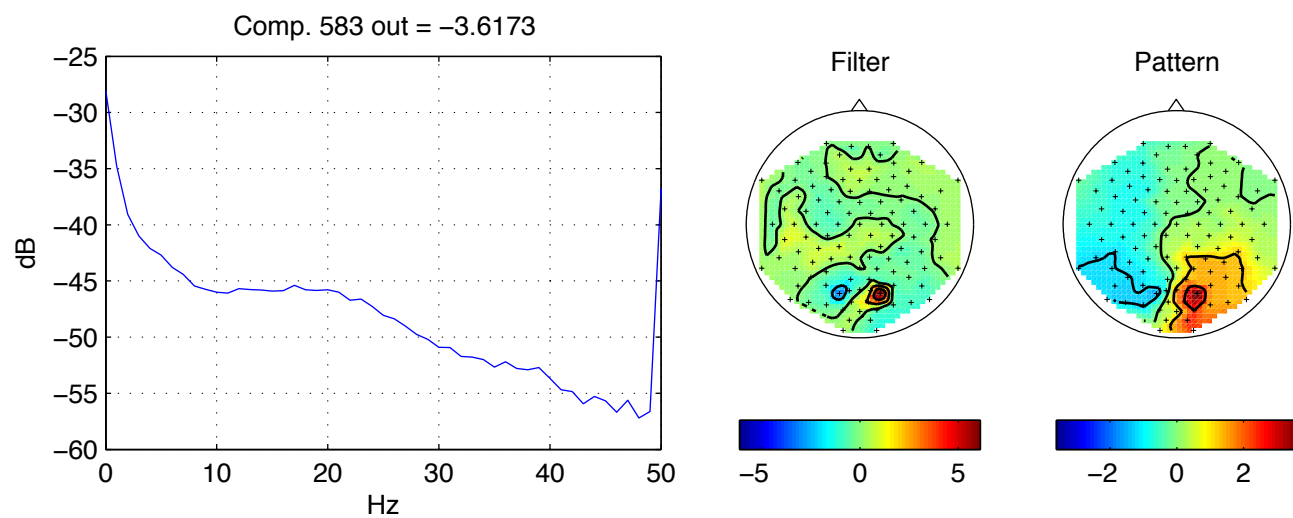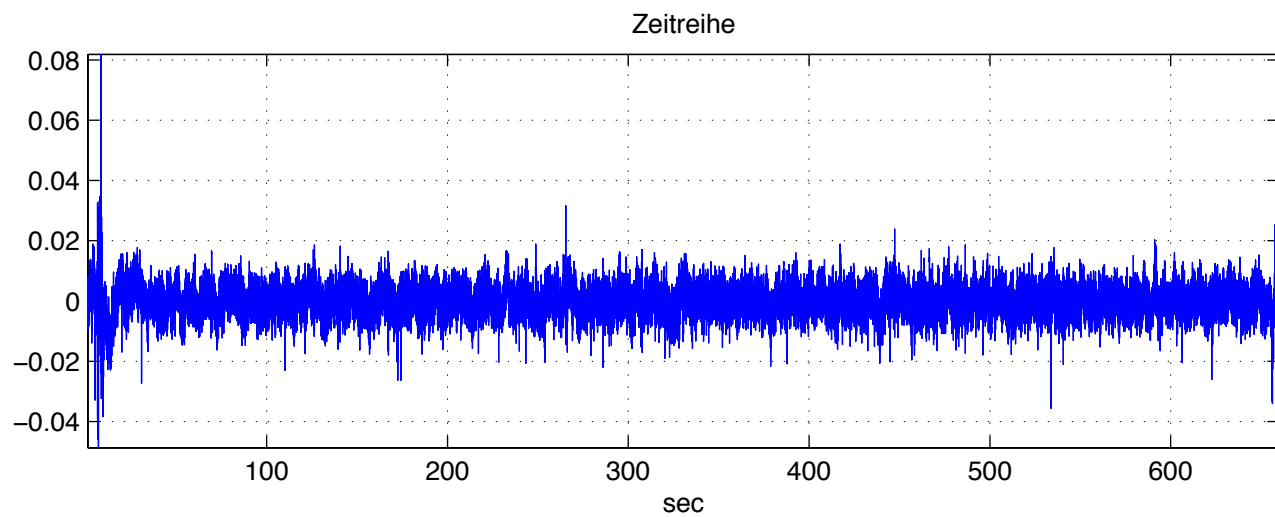

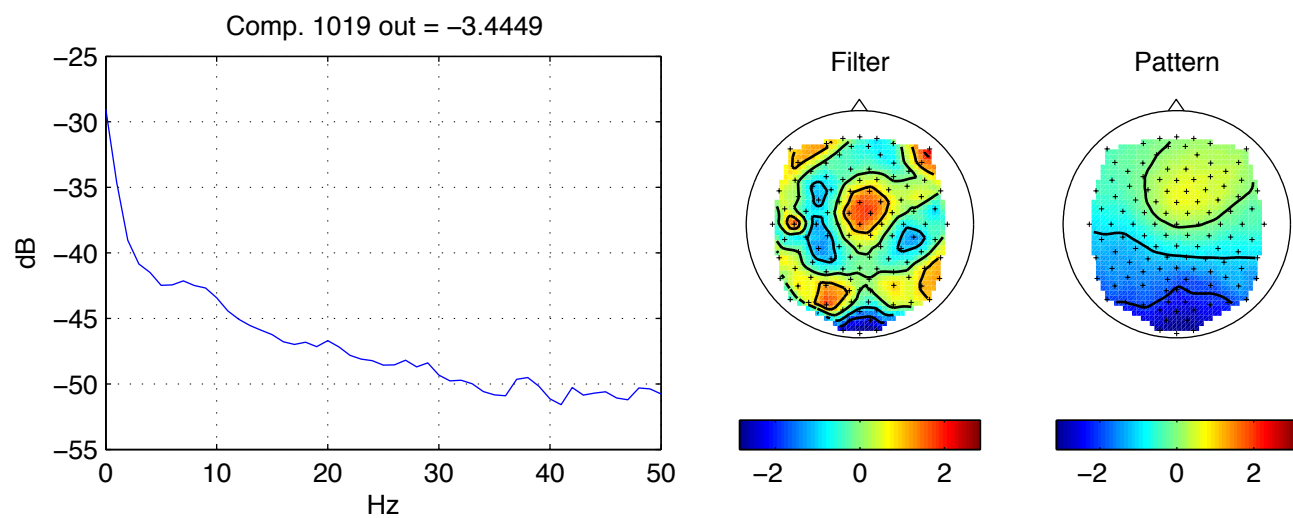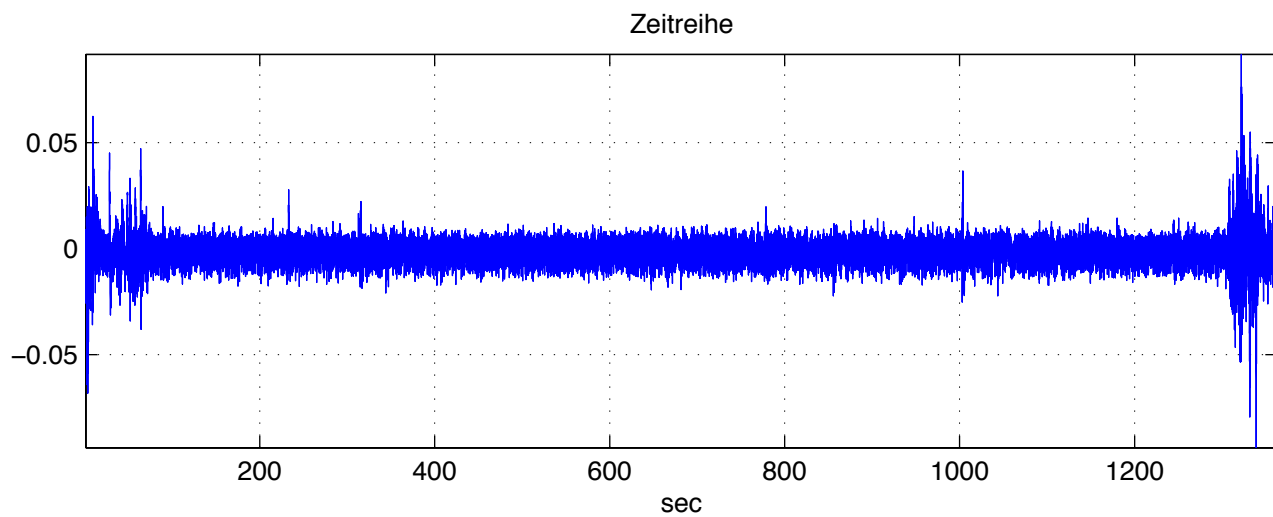

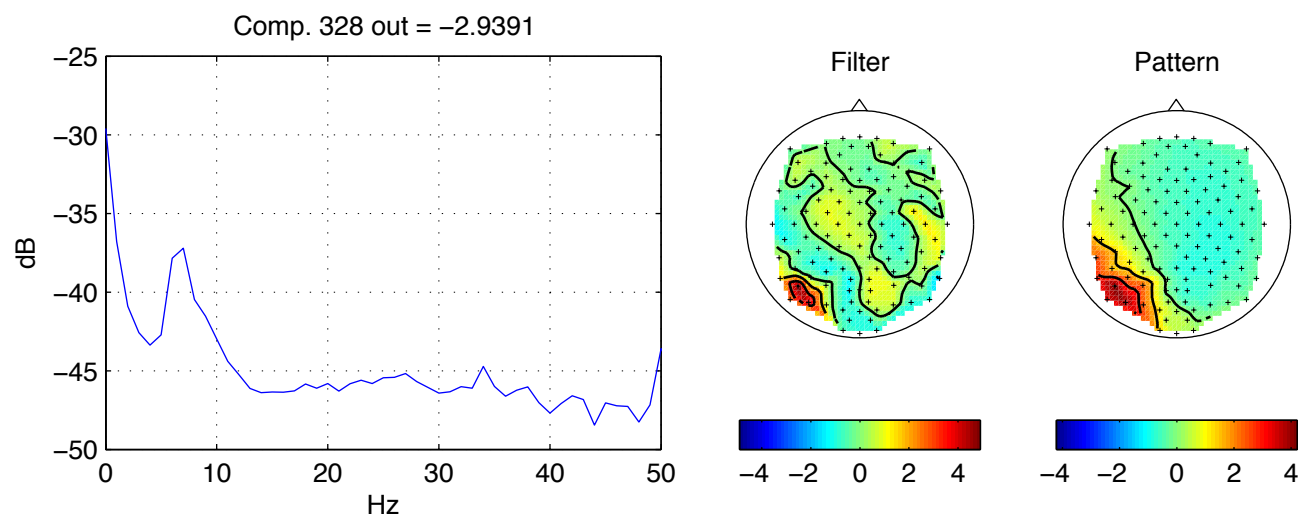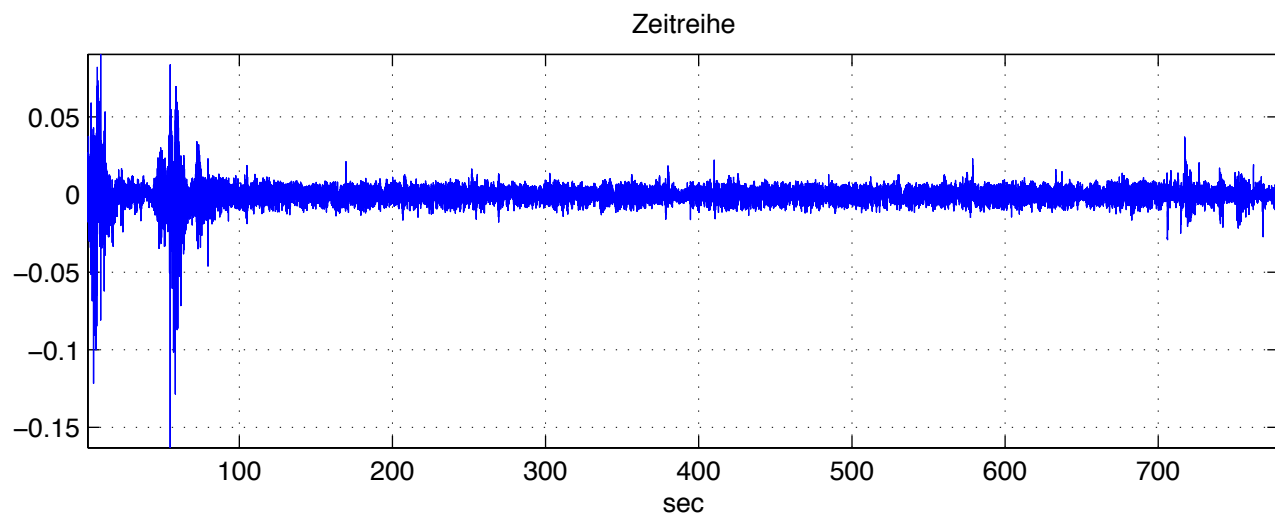

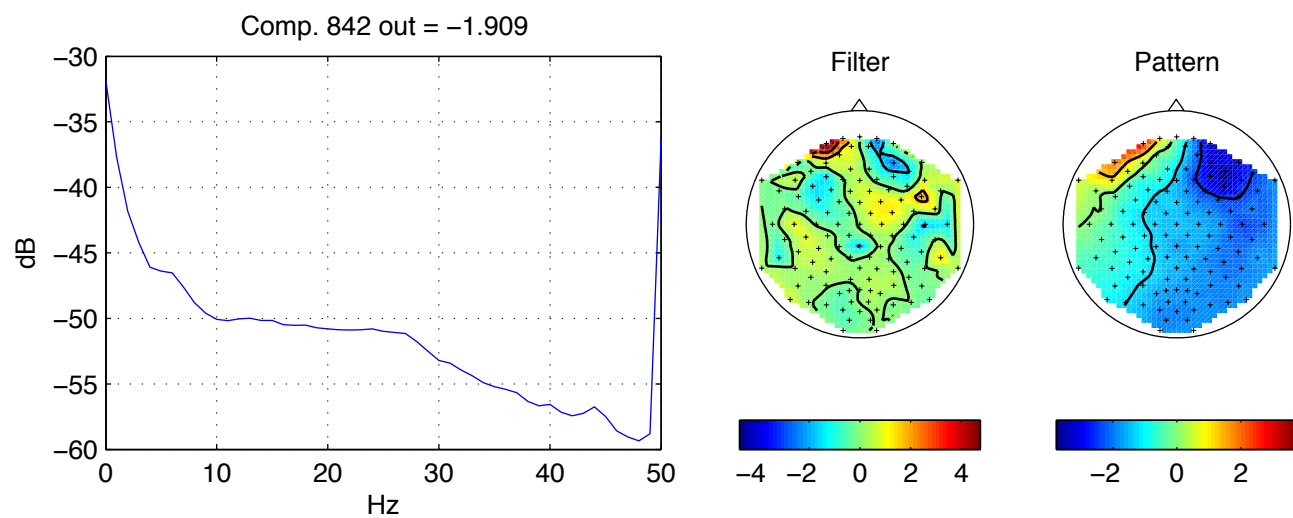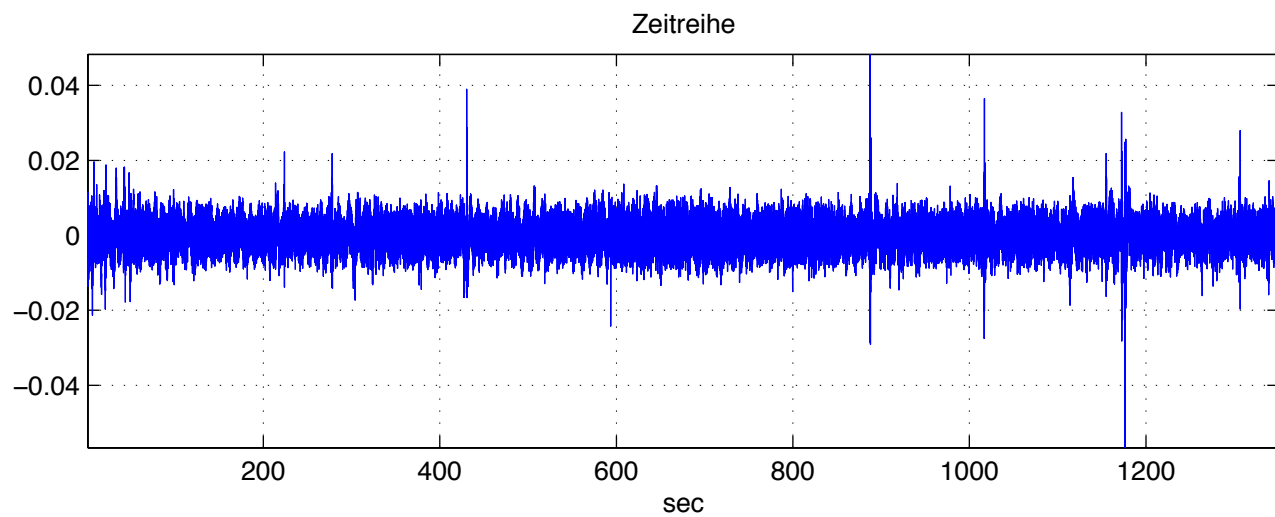

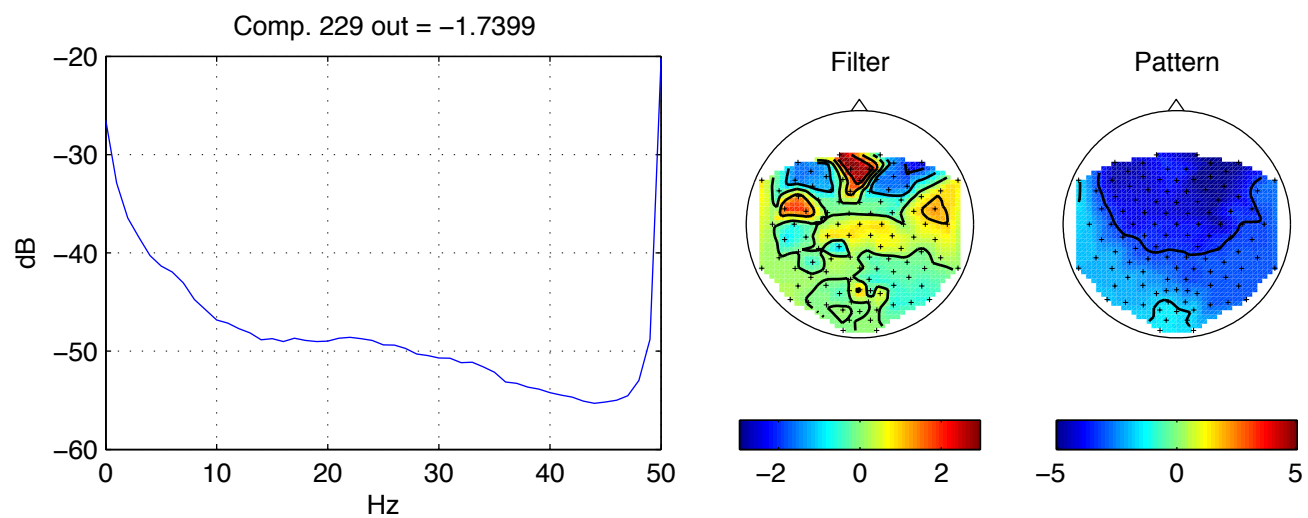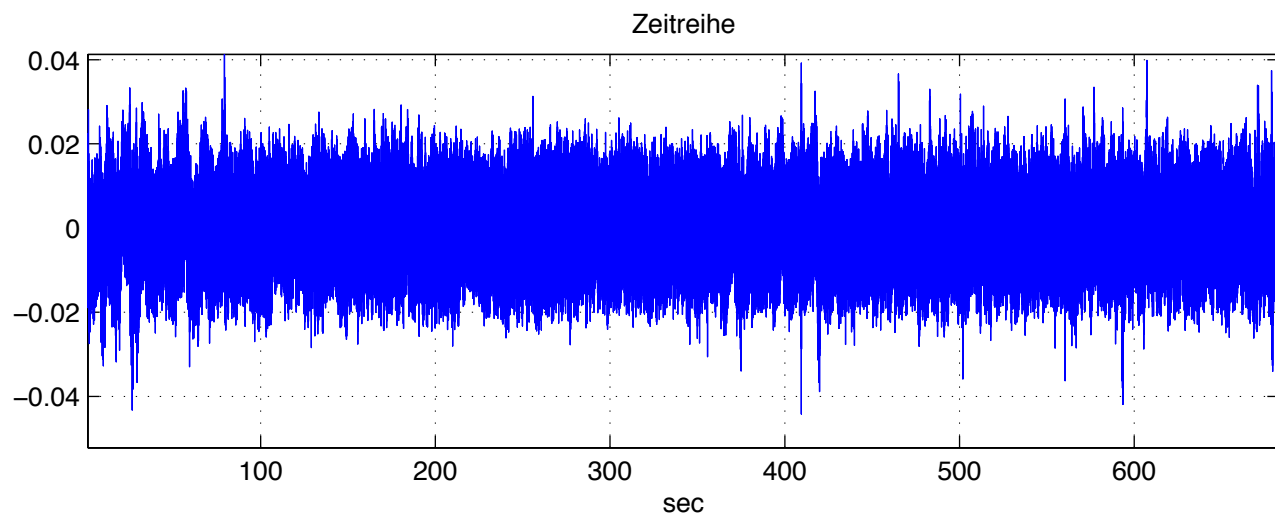

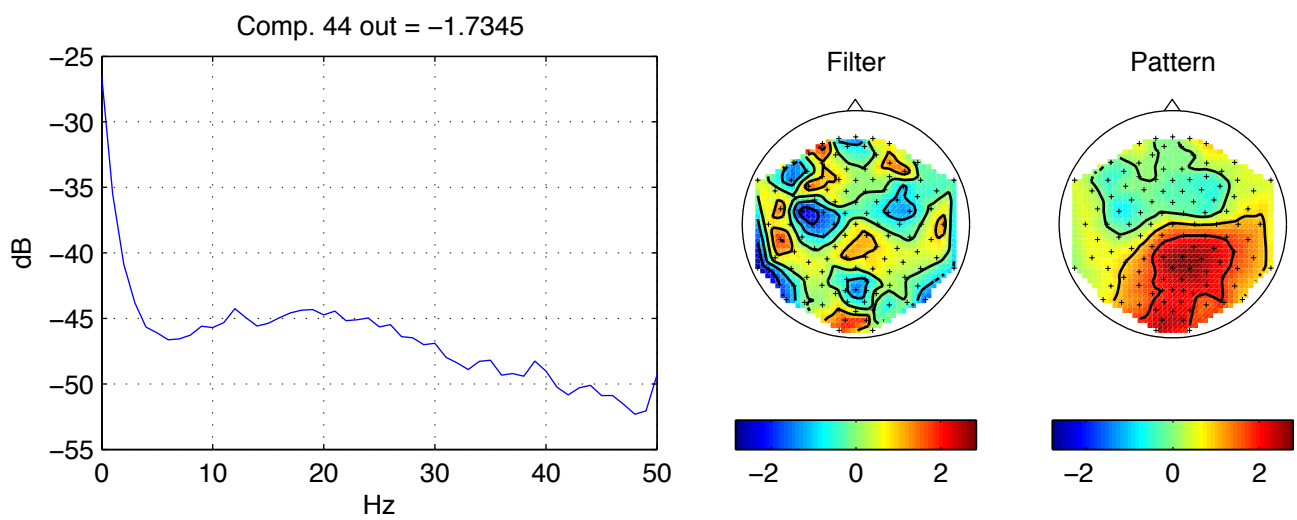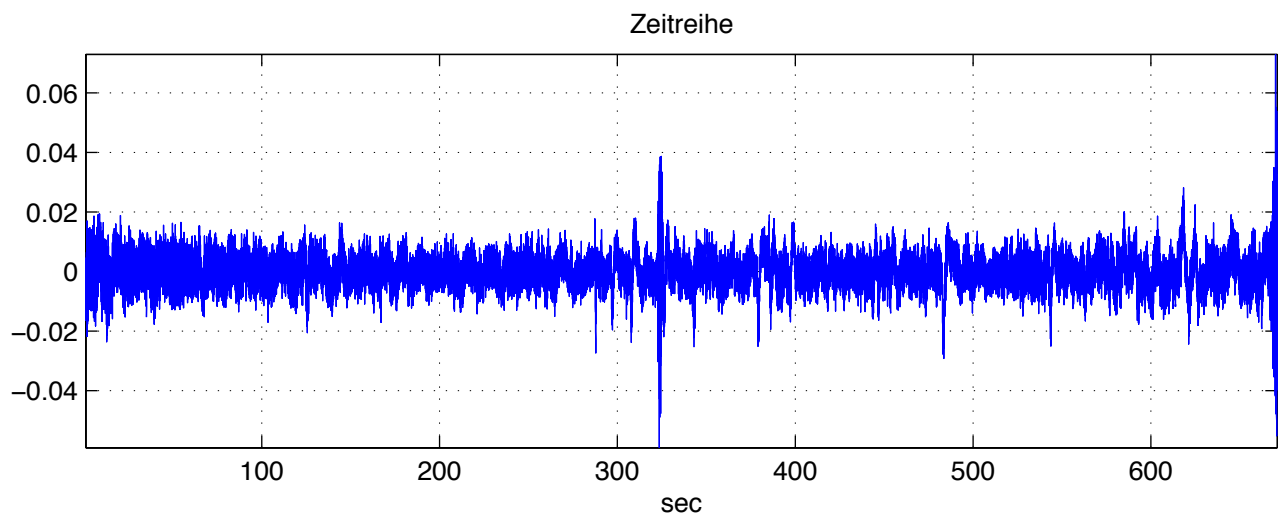

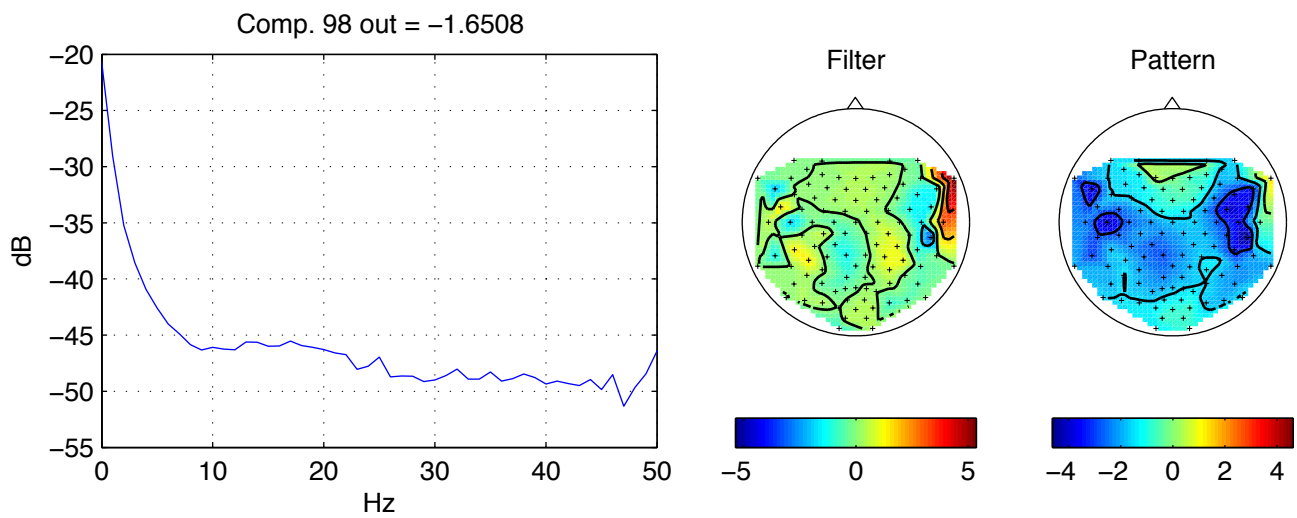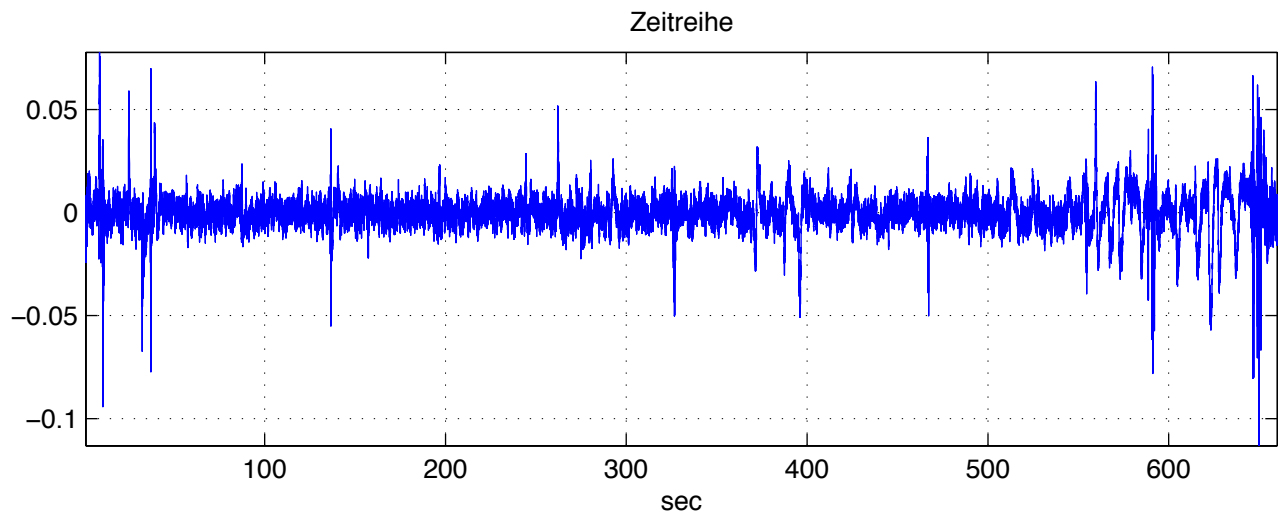

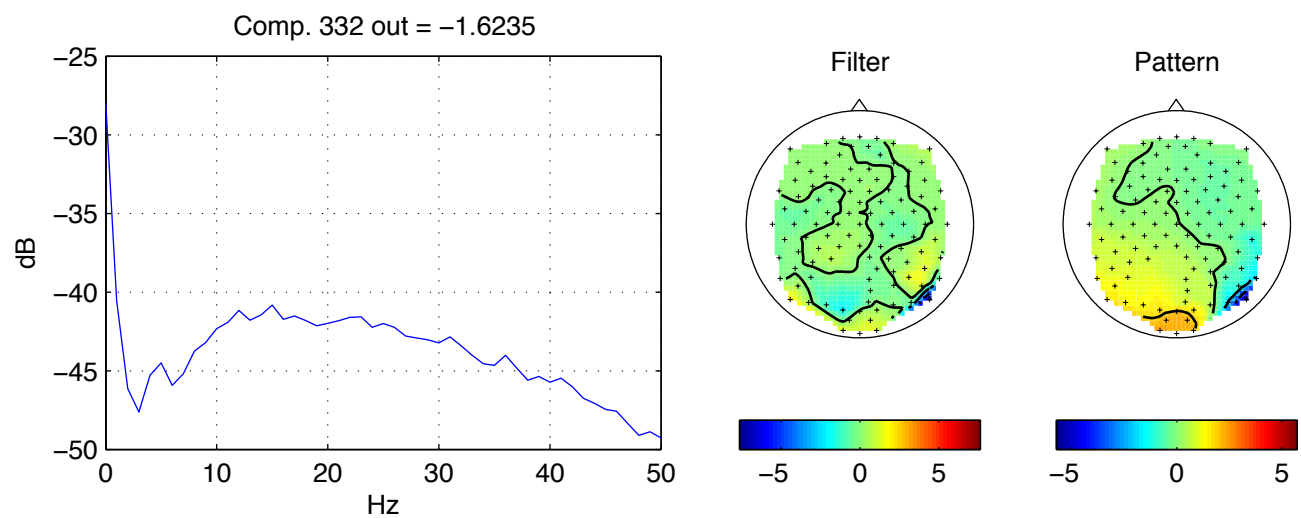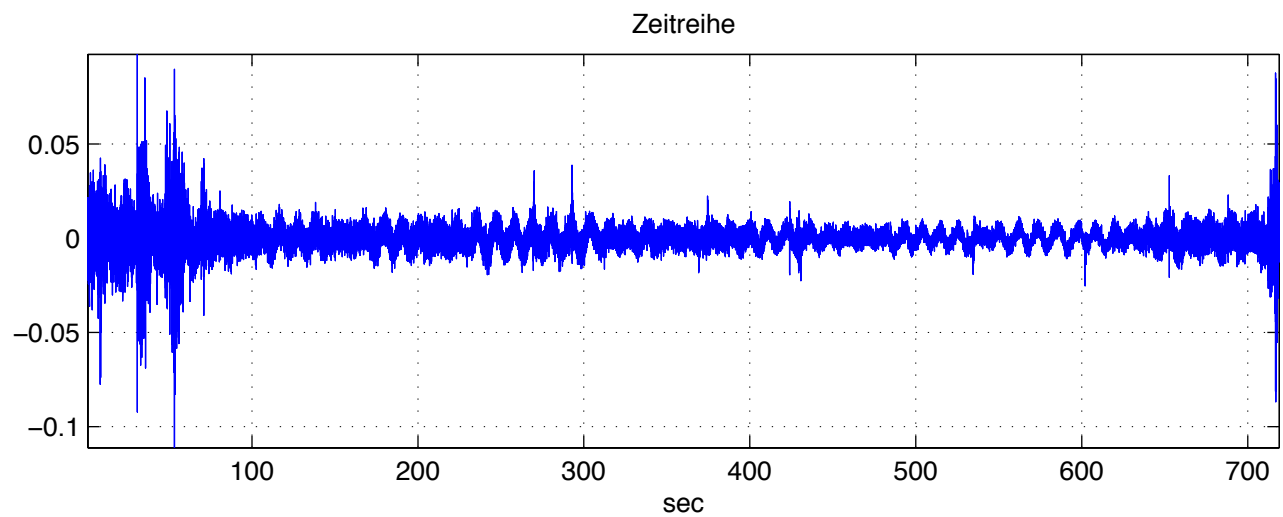

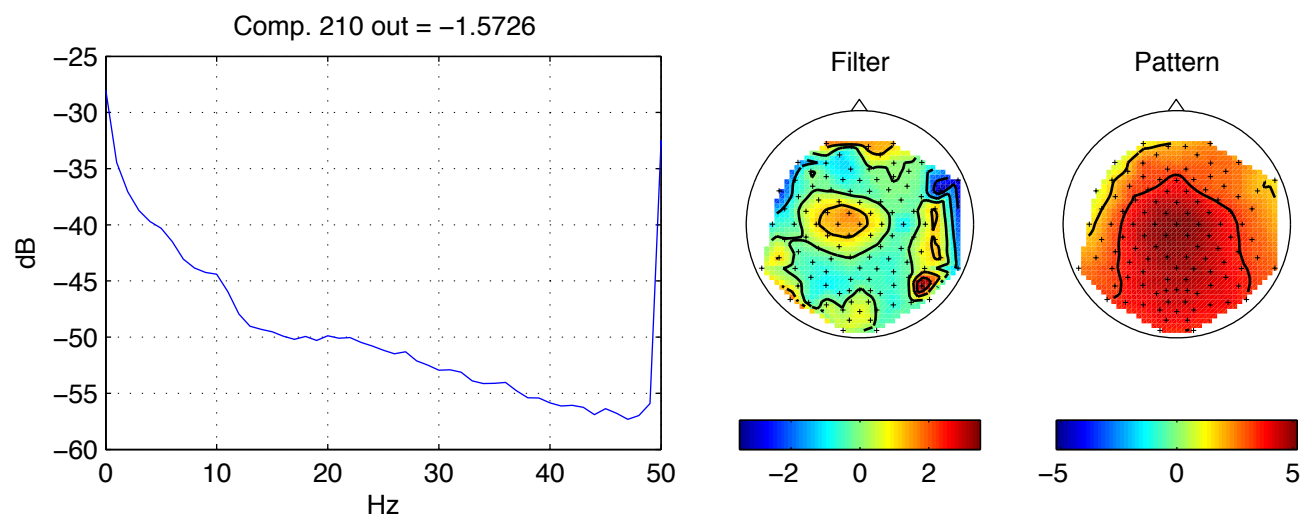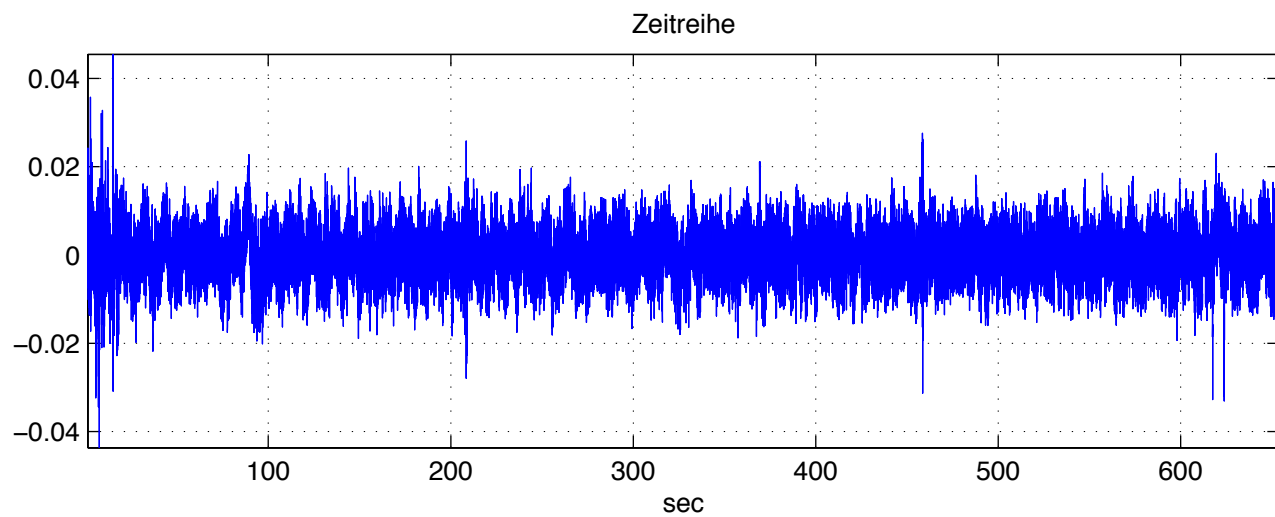

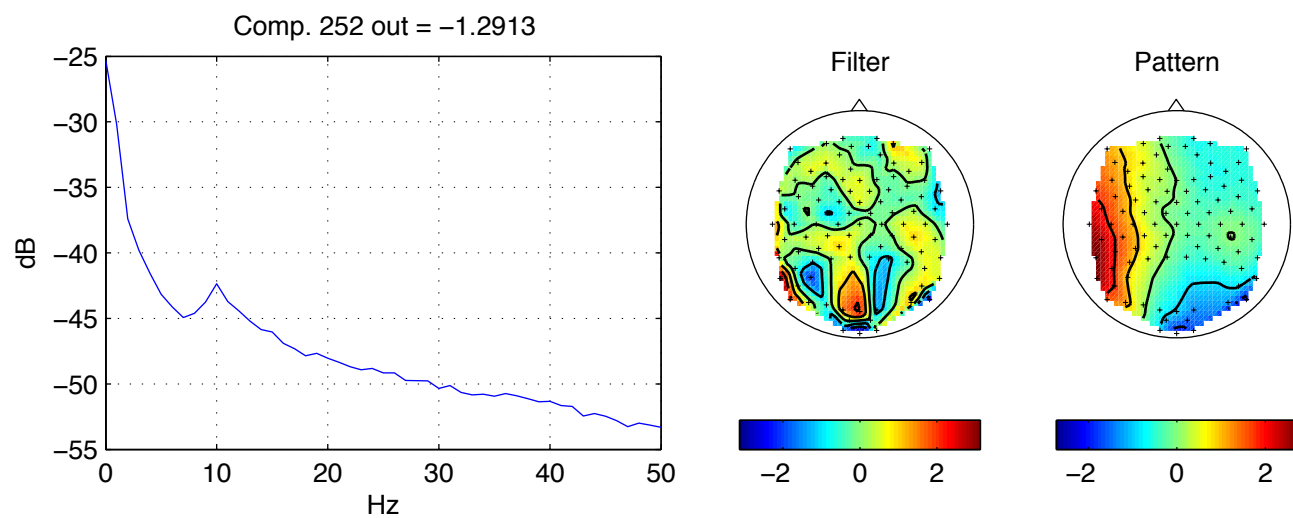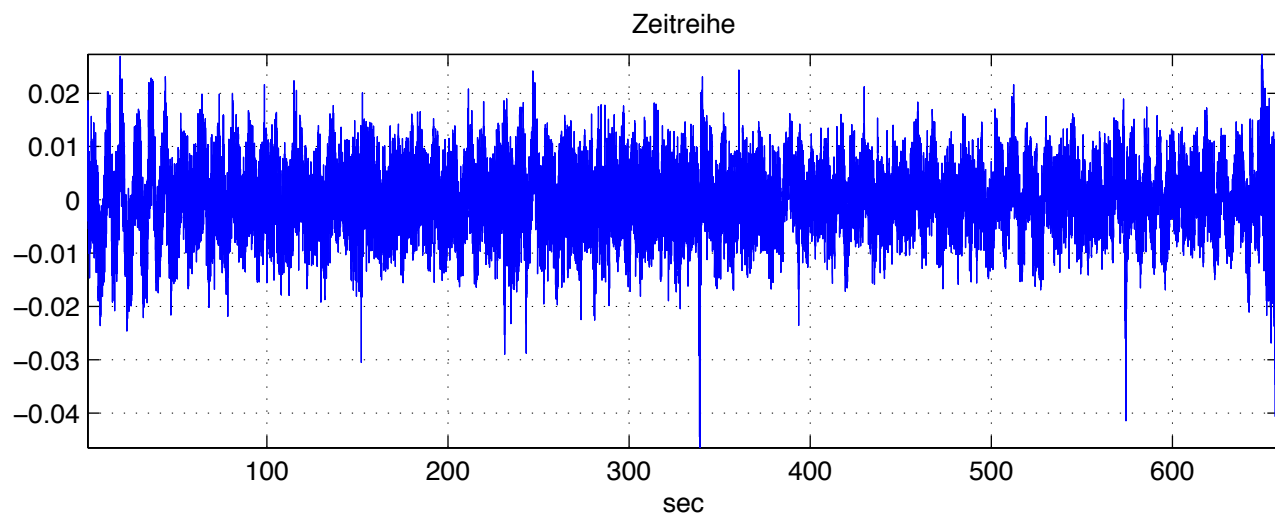

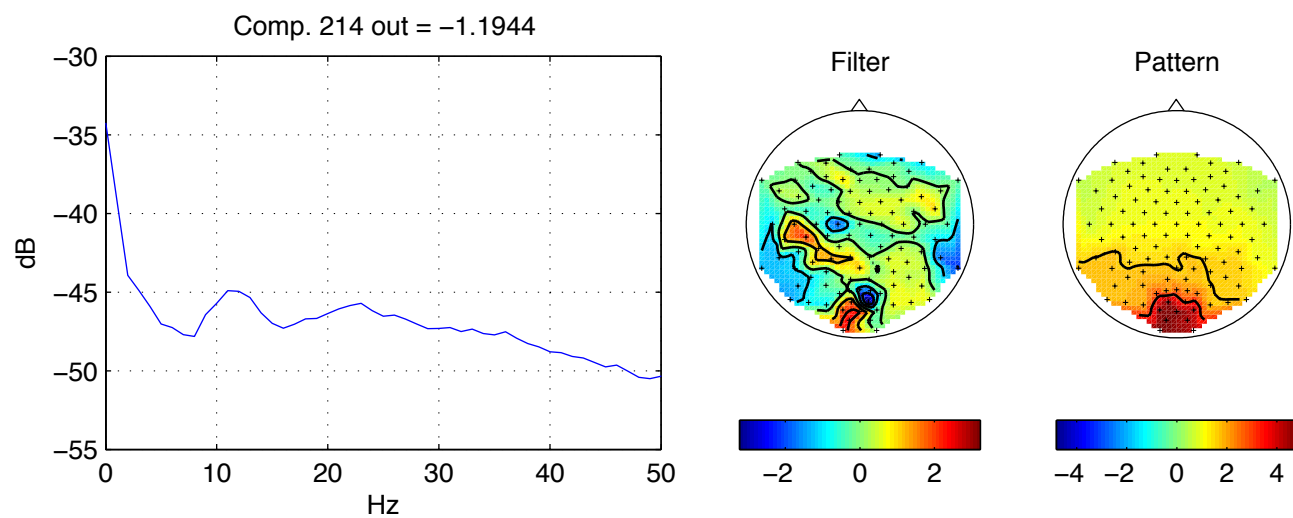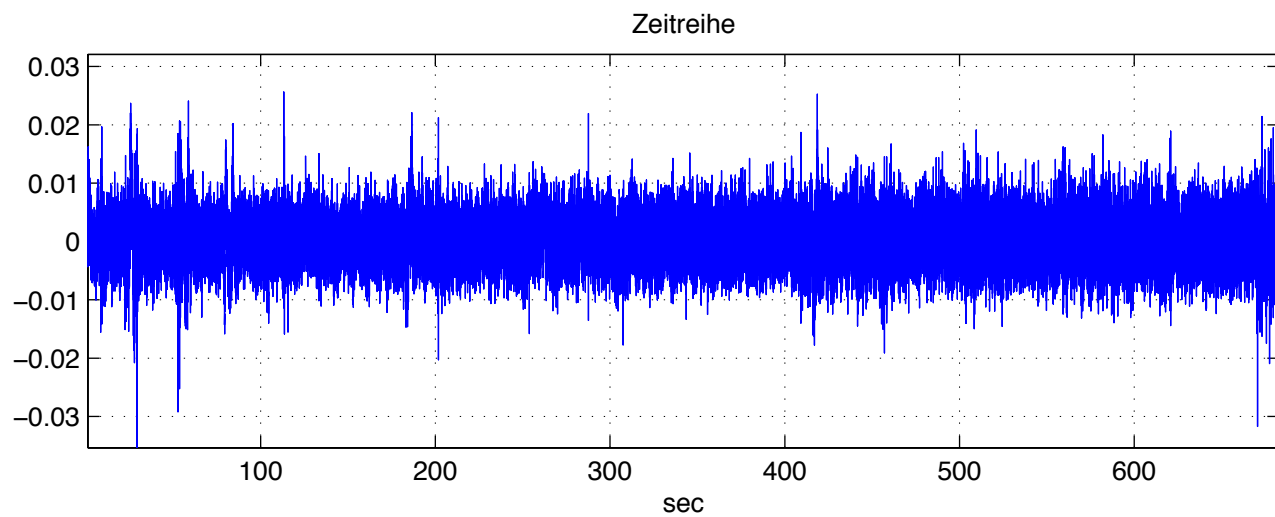

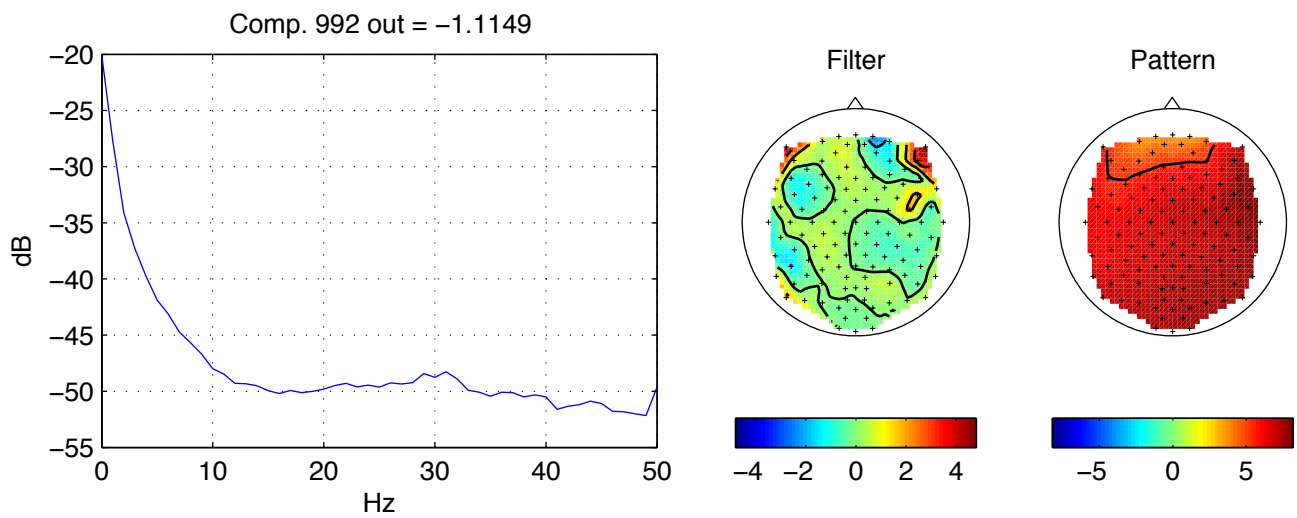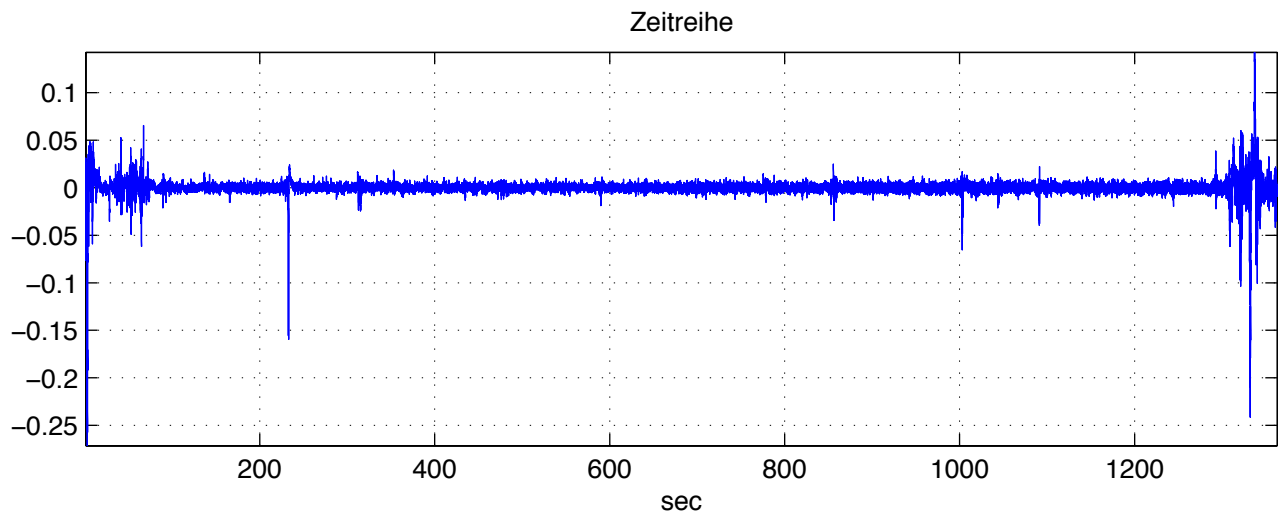

Comp. 515 out = -0.9033

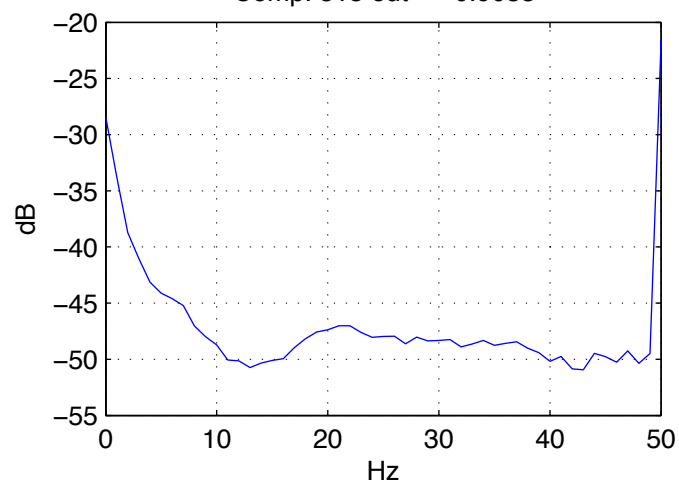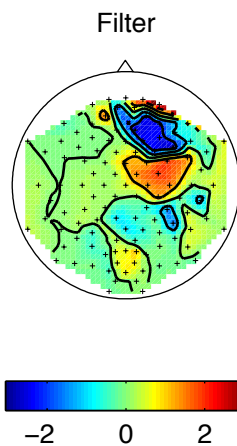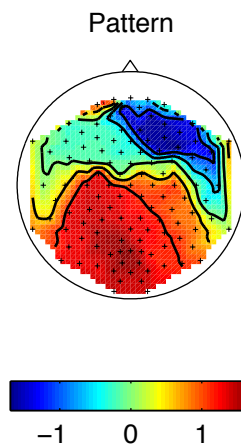

Zeitreihe

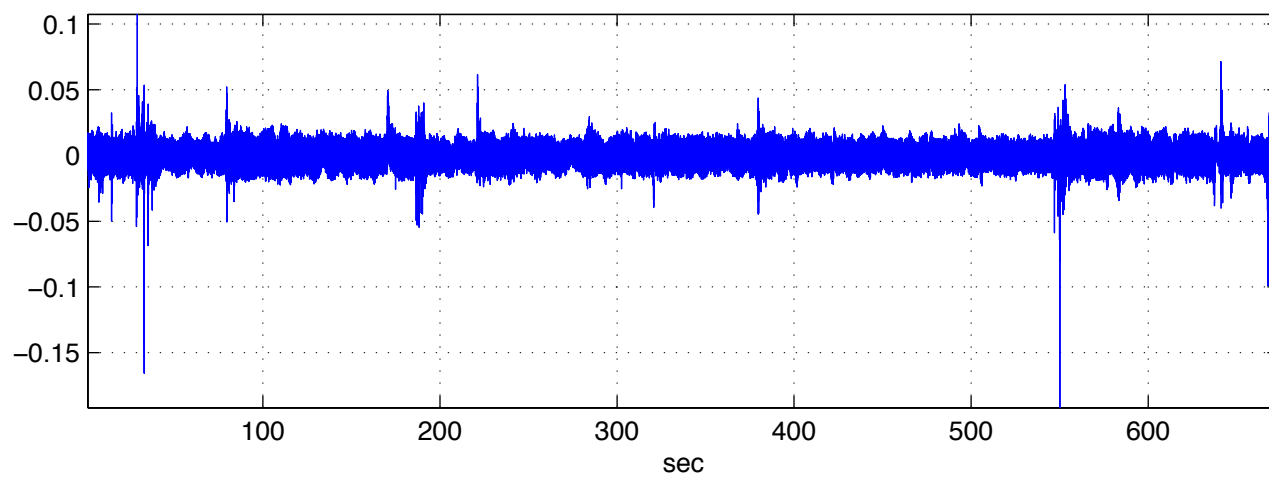

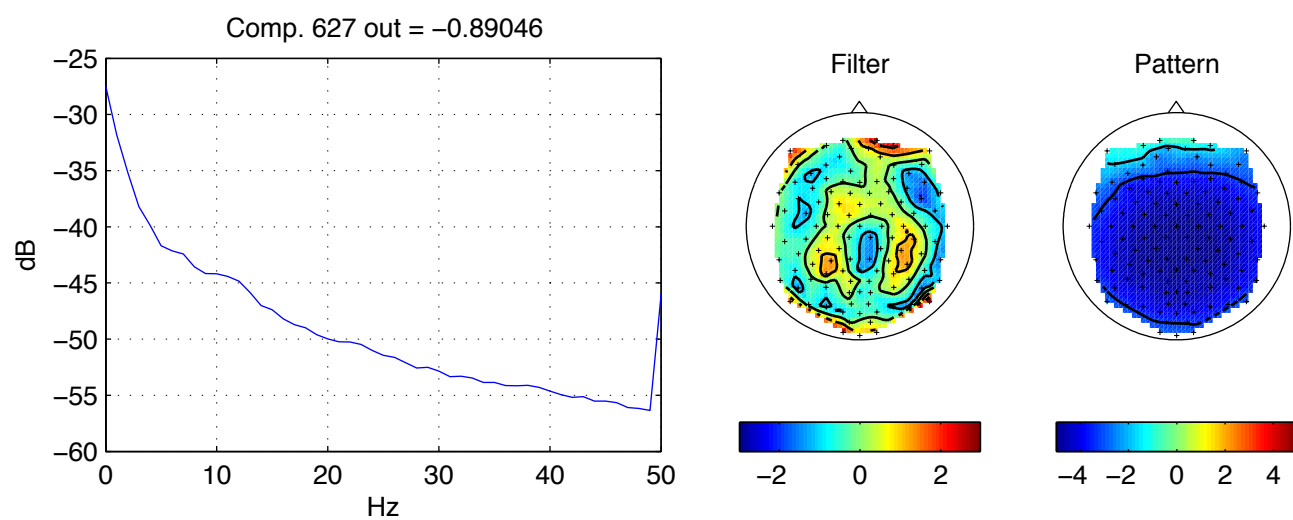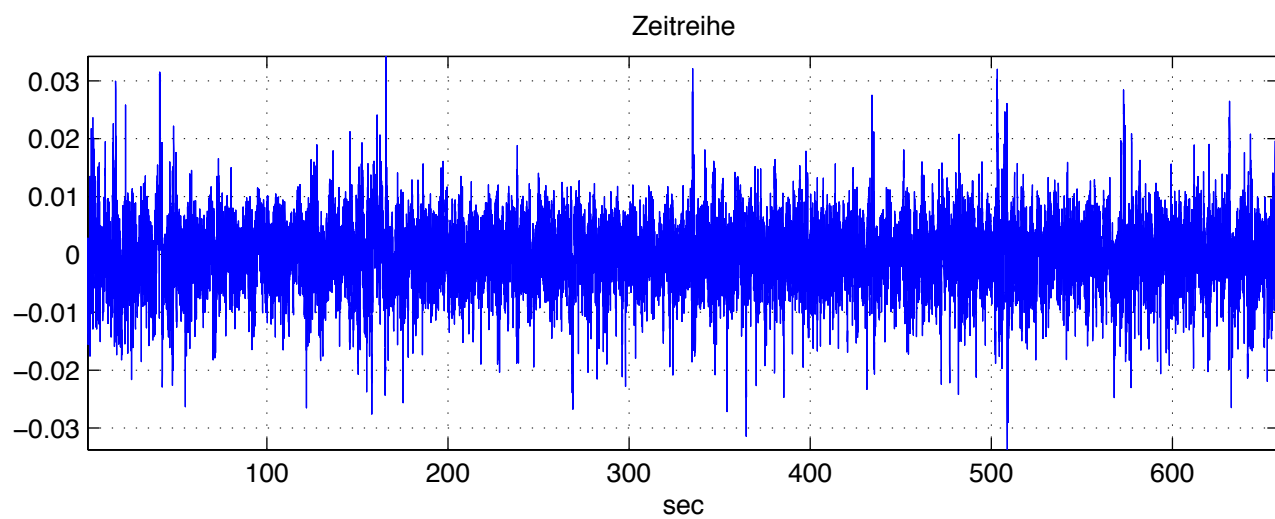

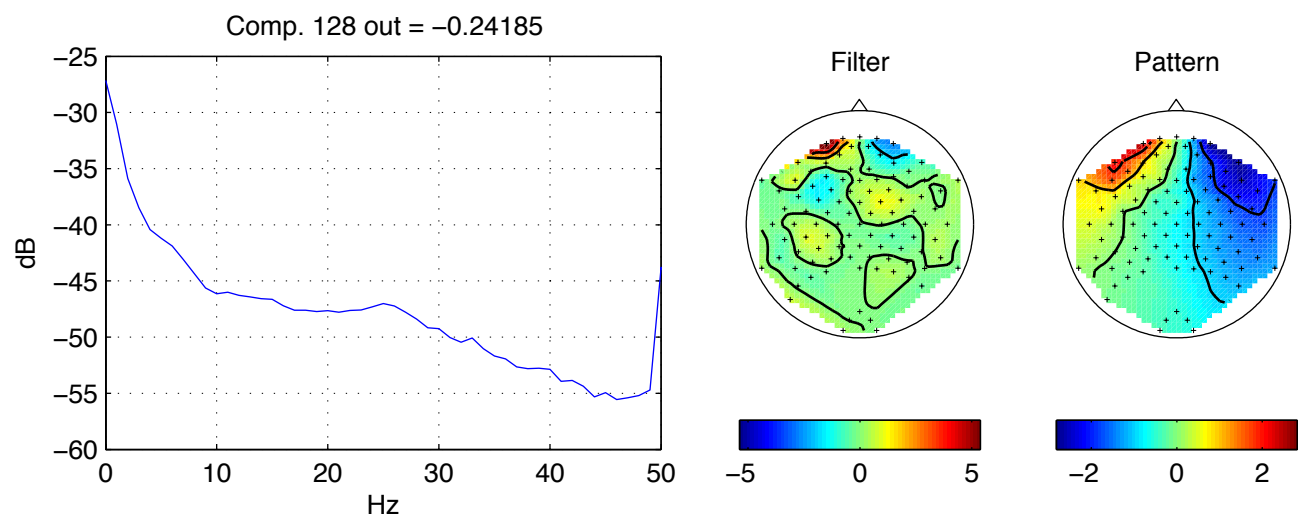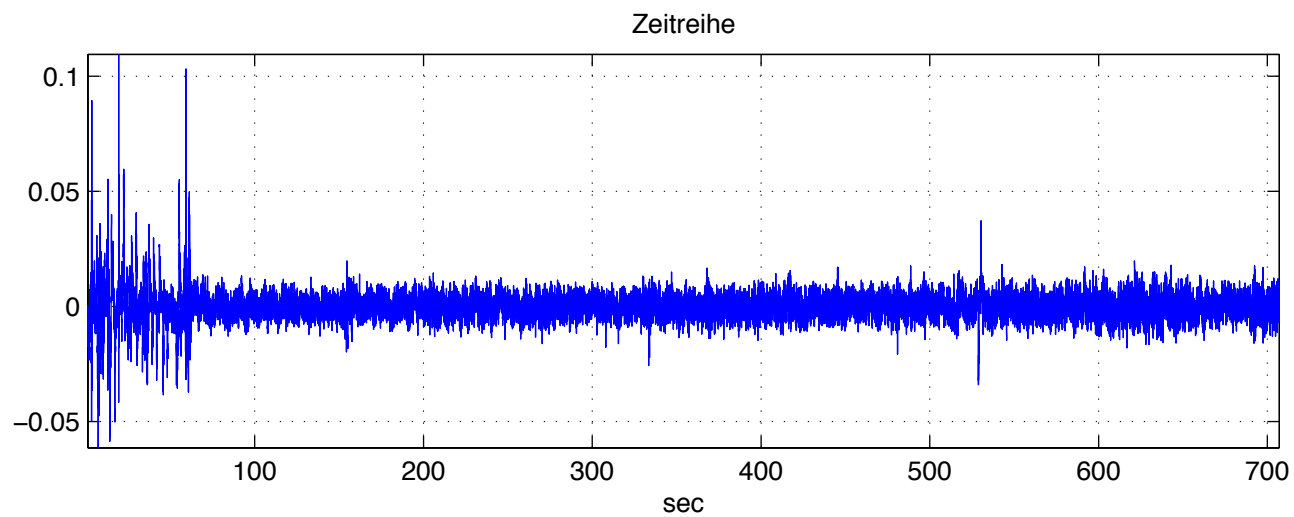

Supplement: Additional file 4 — Misclassifications. Visualization of the 75 + 21 misclassified components of the RT test data [file 1744-9081-7-30-S4.GZ › misclass/RLDA_says_neural_activity.pdf]
